# Pharmacological interventions for the prevention of renal injury in surgical patients: a systematic literature review and meta-analysis

**DOI:** 10.1016/j.bja.2020.06.064

**Published:** 2020-08-20

**Authors:** Suraj Pathak, Guido Olivieri, Walid Mohamed, Riccardo Abbasciano, Marius Roman, Sara Tomassini, Florence Lai, Marcin Wozniak, Gavin J. Murphy

**Affiliations:** Department of Cardiovascular Sciences, National Institute for Health Research Leicester Biomedical Research Unit in Cardiovascular Medicine, University of Leicester, Leicester, UK

**Keywords:** clinical outcome, kidney injury, pharmacological interventions, renal protection, surgery, systematic review

## Abstract

**Background:**

The aim of this systematic review was to summarise the results of randomised controlled trials (RCTs) that have evaluated pharmacological interventions for renoprotection in people undergoing surgery.

**Methods:**

Searches were conducted to update a previous review using the Cochrane Central Register of Controlled Trials, MEDLINE, and EMBASE to August 23, 2019. RCTs evaluating the use of pharmacological interventions for renal protection in the perioperative period were included. The co-primary outcome measures were 30-day mortality and acute kidney injury (AKI). Pooled effect estimates were expressed as risk ratios (RRs) (95% confidence intervals).

**Results:**

We included 228 trials enrolling 56 047 patients. Twenty-three trials were considered to be at low risk of bias across all domains. Atrial natriuretic peptides (14 trials; *n*=2207) reduced 30-day mortality (RR: 0.63 [0.41, 0.97]) and AKI events (RR: 0.43 [0.33, 0.56]) without heterogeneity. These effects were consistent across cardiac surgery and vascular surgery subgroups, and in sensitivity analyses restricted to studies at low risk of bias. Inodilators (13 trials; *n*=2941) reduced mortality (RR: 0.71 [0.53, 0.94]) and AKI events (RR: 0.65 [0.50, 0.85]) in the primary analysis and in cardiac surgery cohorts. Vasopressors (4 trials; *n*=1047) reduced AKI (RR: 0.56 [0.36, 0.86]). Nitric oxide donors, alpha-2-agonists, and calcium channel blockers reduced AKI in primary analyses, but not after exclusion of studies at risk of bias. Overall, assessment of the certainty of the effect estimates was low.

**Conclusions:**

There are multiple effective pharmacological renoprotective interventions for people undergoing surgery.

**Editor's key points**

• The authors performed a systematic review and meta-analysis of 228 studies evaluating pharmacological perioperative renoprotection.
• Interventions improving mortality and acute kidney injury included atrial natriuretic peptides, alpha-2-agonists, inodilators, vasopressors, calcium channel blockers, and nitric oxide donors. Statins, spironolactone, and restrictive *vs* liberal fluid therapy were associated with worsened mortality and acute kidney injury.
• This review identifies new therapeutic approaches for renoprotection in surgical patients, and provides a useful framework for future research.

Acute kidney injury (AKI) is a common and serious complication of surgery that is associated with increased morbidity, mortality, and use of healthcare resources.^1^, ^2^, ^3^ Given the growing number of people referred for surgery who are elderly or who have multiple chronic conditions, including diabetes and chronic kidney disease, the clinical burden of AKI is likely to increase.^4^ Our current understanding of the underlying pathology remains poor, and existing evidence reviews have concluded that there are no effective treatments.^1^^,^^5^ A 2013 Cochrane systematic review by Zacharias and colleagues^1^ did not identify any effective renoprotective pharmacological interventions in 72 trials of eight interventions (dopamine and analogues, diuretics, calcium channel blockers, angiotensin-converting enzyme (ACE) inhibitors, atrial natriuretic peptide (ANP), *N*-acetylcysteine, erythropoietin, and intravenous fluid).^5^ There has been a significant increase in research activity in this area in recent years. The aim of this systematic review was to update the review by Zacharias and colleagues^1^ of renoprotection interventions in people undergoing surgery, highlight knowledge gaps, and identify potential areas for research.

## Methods

A systematic review of RCTs was performed using the methods described in *Cochrane Handbook for Systematic Reviews of Interventions*.^6^ A protocol was registered prospectively on PROSPERO (CRD42018115851).^7^ The study adhered to the Preferred Reporting Items for Systematic Reviews and Meta-Analyses guidelines.^8^

### Study eligibility

Inclusion criteria were RCTs comparing any pharmacological intervention against controls (placebo or no intervention) in adult patients undergoing surgery for cardiovascular disease, thoracic disease, neoplastic disease, orthopaedic disease/injury, or gastrointestinal disease, irrespective of blinding, language, publication status, date of publication, and sample size.

Exclusion criteria were non-RCT studies; trials evaluating multiple interventions concomitantly; trials evaluating non-pharmacological interventions; paediatric trials (age <18 yr); or where patients underwent transplant surgery, surgery of the kidneys and urinary tract, resuscitation for trauma, or treatment for burns injuries, gastrointestinal haemorrhage, intracranial haemorrhage, obstetric conditions, or critical care patients.

### Data sources and search strategy

Electronic searches were conducted to update the Cochrane review conducted by Zacharias and colleagues^1^ using the Cochrane Central Register of Controlled Trials, MEDLINE, and EMBASE from the date of the final search recorded in the review by Zacharias and colleagues (August 2012) to August 23, 2019. A full description of the search terms is listed in Supplementary material 1.

### Study selection

Two reviewers screened the results of the literature search independently of each other and identified studies for inclusion. Full texts of shortlisted studies were retrieved and further assessed for inclusion. Excluded studies and the reason for exclusion were recorded. Disagreements were resolved by discussion, or where this was not possible, by the senior author.

### Assessment of methodological quality

Included trials were appraised using the Cochrane risk-of-bias tool.^9^ Two authors assessed each outcome of interest as being at either low, high, or unclear risk of bias for each domain. Disagreements were resolved as mentioned previously. Studies at low risk of bias in all domains are referred to in the text as ‘high-quality’ studies.

### Data extraction

Data were extracted by two reviewers using a specifically designed *pro forma*. This included year, study type, setting, sample size, participant characteristics, baseline characteristics, type of surgery, details of interventions, outcomes, and risk-of-bias assessments. Standard errors of the mean and inter-quartile ranges (IQRs) were converted to standard deviations using appropriate formulae.^9^

The co-primary outcomes included 30-day mortality or hospital all-cause mortality, and AKI, as defined by the study authors.

We considered renal replacement therapy (RRT) as a secondary outcome, as this is often not specific to renal injury, particularly in cardiac surgery, where it is commonly used for management of acidosis in low-cardiac-output states. Other secondary outcome measures included urine output at 24 h; creatinine clearance at 24 h, 2–4 days, and 5–7 days post-surgery; perioperative blood loss; myocardial infarction; acute brain injury/stroke; sepsis/infection; hospital length of stay; ICU length of stay; low cardiac output; and adverse events, as defined by the study authors.

### Missing data

For dichotomous data presented only as percentages, frequencies were estimated from reported sample sizes. For continuous outcomes, the mean and the standard deviation were estimated from median (IQR) values using standard formulae, if not available from the paper or study authors.^9^

### Data analysis

Risk ratios (RRs) with 95% confidence interval (CI) were estimated for dichotomous outcomes using the Mantel–Haenszel random-effects method. Mean difference (MD) with 95% CI was estimated for continuous outcomes using inverse-variance random-effects method. The *I*^2^ statistic was used to estimate the percentage of total variation across studies attributed to heterogeneity rather than chance. All analyses were carried out using Review Manager (RevMan) version 5.3 (Nordic Cochrane Centre, Copenhagen, Denmark) and the R software package (University of Auckland, New Zealand).

### Subgroup analysis

A subgroup analysis assessed whether treatment effects interacted with clinical setting (i.e. vascular surgery, cardiac surgery, and general surgery).

### Sensitivity analysis

Sensitivity analyses excluded studies with high or uncertain risk of bias in any one of the domains of the Cochrane risk-of-bias tool. Additional sensitivity analyses excluded only studies at high or uncertain risk of allocation concealment bias.

### Reporting bias

Publication bias for the co-primary outcomes was assessed using funnel plots and Egger's intercept test, where 10 or more studies contributed to an outcome.

### Data summary

The quality of evidence was assessed using Grading of Recommendations, Assessment, Development and Evaluation (GRADE) methodology using GRADEpro GDT software (gdt.gradepro.org).^10^^,^^11^

## Results

### Characteristics of studies

**Fig 1:**
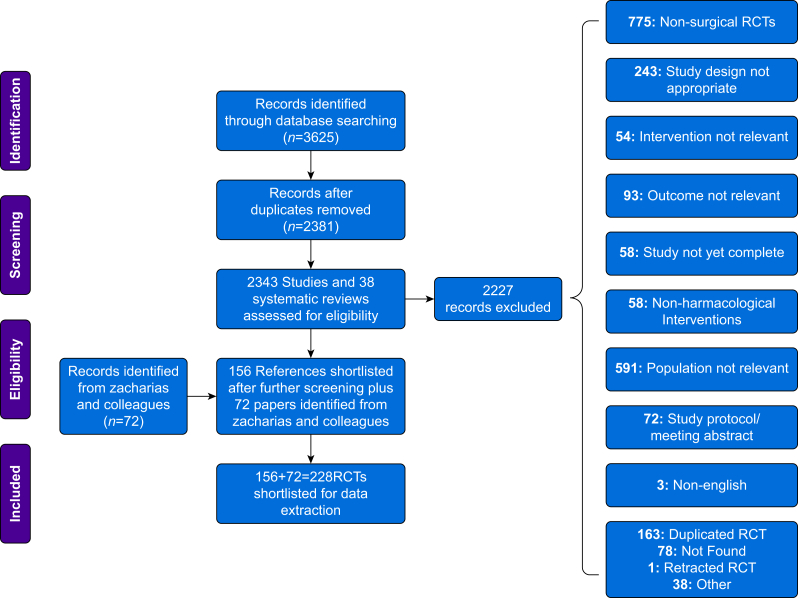
Preferred Reporting Items for Systematic Reviews and Meta-Analyses (PRISMA) diagram.

A flow diagram showing the results of the searches and exclusions is shown in Figure 1. In total, 228 RCTs, including 72 trials from the review by Zacharias and colleagues,^1^ were included in the data synthesis (Supplementary Table 1). A summary of the characteristics of each trial is listed in Supplementary Table 2.

### Assessment of bias

The results of the risk-of-bias assessments are shown in Supplementary Figs 1 and 2. A total of 105 trials were at low risk of allocation concealment and 23 trials were considered to be at low risk of bias in all bias domains.

### Data synthesis

For the primary analysis, the included studies were analysed by the type of intervention and were reported as trials of interventions that demonstrate renoprotective effects, harmful effects, or neither renoprotective effects nor harm, on the co-primary outcome measures (Table 1). Results from our primary analysis and subgroup analyses are presented in Supplementary Tables 3–6.

**Table 1:**
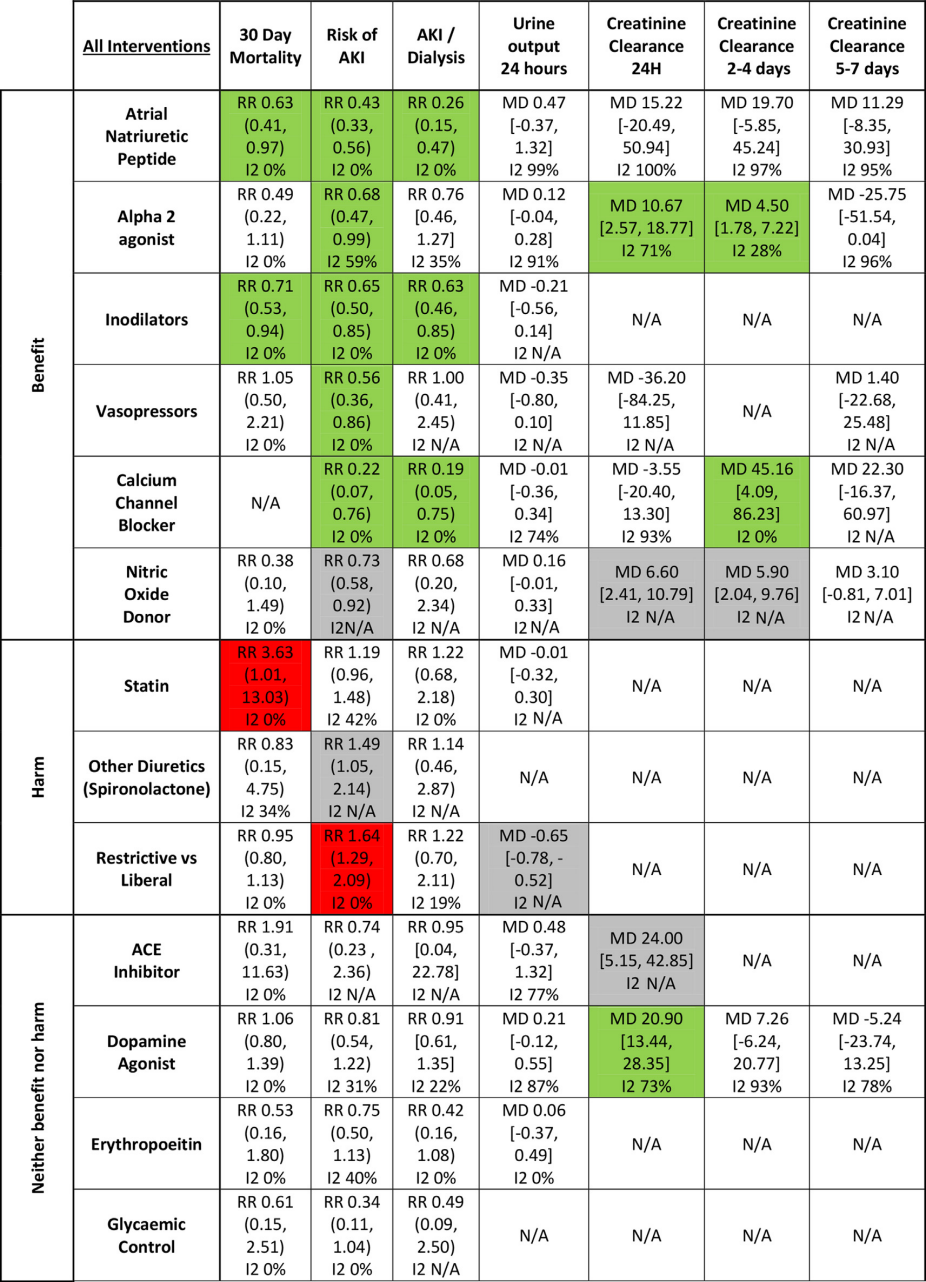

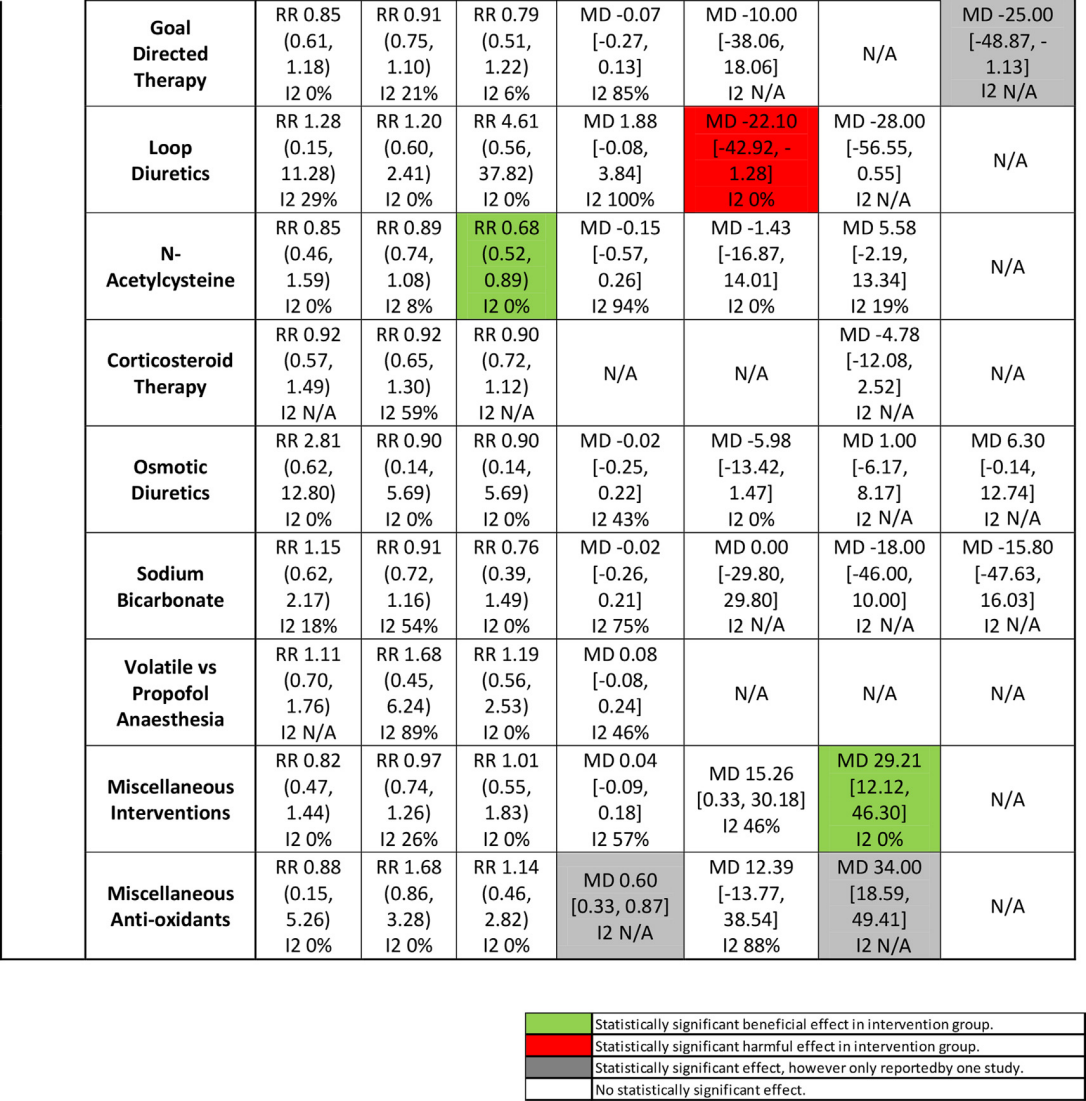
Key outcomes for all interventions. Colour codes indicate direction of treatment effect on that outcome. Red indicates a harmful effect and green indicates a beneficial effect. Green, statistically significant beneficial effect in intervention group; red, statistically significant harmful effect in intervention group; grey, statistically significant effect, but reported only by one study; white, no statistically significant effect. ACE, angiotensin-converting enzyme; AKI, acute kidney injury; MD, mean difference; N/A, not applicable; RR, risk ratio.

### Interventions with renoprotective effects

*Atrial natriuretic peptides* were evaluated in 14 trials with 2207 participants. Three RCTs evaluated nesiritide, one evaluated carperitide, and 10 evaluated synthetic ANP. In the primary analysis, ANP significantly reduced 30-day mortality (RR: 0.63 [0.41–0.97]; *P*=0.04; *I*^2^=0%) and AKI (RR: 0.43 [0.33–0.56]; *P*<0.001; *I*^2^=0%). ANP therapy also reduced RRT (RR: 0.26 [0.15–0.47]; *P*<0.001; *I*^2^=0%), with no significant treatment effects on other secondary outcomes. Funnel plots and Egger's test did not indicate likely publication bias (Supplementary Fig. 3 and Table 7).

Sensitivity analysis restricted to studies at low risk of allocation concealment bias (Supplementary Table 8; four RCTs with 942 participants) demonstrated no significant treatment effect on 30-day mortality, although significant treatment effects for AKI (RR: 0.40 [0.30–0.54]; *P*<0.001; *I*^2^=0%) and RRT (RR: 0.28 [0.15–0.52]; *P*<0.001; *I*^2^=0%) persisted. Sensitivity analysis restricted to high-quality trials only (Supplementary Table 9; one RCT with 103 participants) demonstrated a significant treatment effect on the risk of AKI (RR: 0.24 [0.07–0.77]; *P*=0.02; *I*^2^=not applicable [N/A]).

Subgroup analyses demonstrated a significant treatment effect of ANP on AKI, mortality, and RRT in cardiac surgery cohorts and on AKI in the vascular surgery cohort (Supplementary Tables 4 and 6).

The GRADE assessment judged the certainty of the effect estimates for 30-day mortality as low, AKI as moderate, and RRT as high (Supplementary Table 10).

*Alpha*=*2*=*agonists* were evaluated in 17 trials with 9167 participants; 15 evaluated dexmedetomidine and two evaluated clonidine. In the primary analysis, alpha-2-agonists significantly reduced AKI (RR: 0.68 [0.47–0.99]; *P*=0.04; *I*^2^=59%), with no treatment effect on mortality. Alpha-2-agonists also increased creatinine clearance at 24 h (MD: 10.67 [2.57–18.77]; *P*=0.010; *I*^2^=71%) and at 2–4 days post-surgery (MD: 4.50 [1.78–7.22]; *P*=0.001; *I*^2^=28%), and reduced acute brain injury/stroke (RR: 0.44 [0.21–0.93]; *P*=0.03; *I*^2^=0%). Funnel plots and Egger's test did not indicate likely publication bias (Supplementary Fig. 4 and Table 7).

No significant treatment effects on the risk of AKI and 2–4 days creatinine clearance were observed in sensitivity analyses, although treatment effects on brain injury and creatinine clearance at 24 h were statistically significant.

Significant treatment effects of alpha-2-agonists on AKI were not observed in any subgroup, although a significant increase in 24 h creatinine clearance was observed in cardiac surgery cohorts.

The GRADE assessment judged the certainty of the effect estimates for 30-day mortality and AKI as high and RRT as moderate (Supplementary Table 11).

*Inodilators* were evaluated in 13 trials with 2941 participants, all of which used levosimendan in patients undergoing cardiac surgery. No studies were considered to be high quality. The primary analysis showed a significant reduction in 30-day mortality (RR: 0.71 [0.53–0.94]; *P*=0.02; *I*^2^=0%) and AKI (RR: 0.65 [0.50–0.85]; *P*=0.002; *I*^2^=0%). Inodilators also reduced the frequency of RRT (RR: 0.63 [0.46–0.85]; *P*=0.003; *I*^2^=0%). Funnel plots and Egger's test suggested significant publication bias for 30-day mortality (*P*=0.001; Supplementary Fig. 5 and Table 7).

Exclusion of studies at high or uncertain risk of allocation concealment eliminated any significant treatment effect on 30-day mortality and AKI, although a significant treatment effect on RRT persisted.

The GRADE assessment judged the certainty of the effect estimates for 30-day mortality, AKI, and RRT as moderate (Supplementary Table 12).

*Vasopressors* were evaluated in four trials with 1047 participants, and included terlipressin, norepinephrine, and phenylephrine. No trials were considered to be high quality. The primary analysis demonstrated a significant reduction in the risk of AKI (RR: 0.56 [0.36–0.86]; *P*=0.009; *I*^2^=0%) with vasopressor treatment, with no significant treatment effect on other outcomes. The significant treatment effect on AKI was not observed in sensitivity analyses.

Subgroup analysis demonstrated a reduction in the risk of AKI in general surgery cohorts, but not in other surgical settings. Vasopressors also reduced perioperative blood loss and ICU length of stay in general surgery cohorts.

The GRADE assessment judged the certainty of the effect estimates for both 30-day mortality and AKI as moderate and RRT as low (Supplementary Table 13).

*Calcium channel blockers* were evaluated in 13 trials with 544 participants; four evaluated diltiazem, two evaluated nifedipine, five evaluated nicardipine, one evaluated felodipine, one evaluated verapamil, and one evaluated nimodipine. No studies were considered to be high quality. No study reported 30-day mortality or all-cause mortality. In the primary analysis, calcium channel blockers caused a significant reduction in the risk of AKI (RR: 0.22 [0.07–0.76]; *P*=0.02; *I*^2^=0%). Analyses of secondary outcomes demonstrated a reduction in the need for RRT and a significant increase in creatinine clearance at 2–4 days post-surgery (MD: 45.16 [4.09–86.23]; *P*=0.03; *I*^2^=0%). Fewer than 10 studies reported frequencies of AKI; therefore, Egger's test was not performed.

Significant treatment effects for calcium channel blockers on AKI, RRT, or 2–4 days creatinine clearance were not observed in any individual subgroup.

The GRADE assessment judged the certainty of the effect estimates for AKI as low and RRT as moderate (Supplementary Table 14).

*Nitric oxide (NO) donors* were evaluated in two RCTs with 484 participants, and included inhaled NO and i.v. sodium nitroprusside. Neither trial was considered to be high quality. In the primary analysis, there was a significant reduction in AKI (RR: 0.73 [0.58–0.92]; *P*=0.008; *I*^2^=N/A) with NO donors, with no treatment effect on mortality. Secondary outcomes described improvements in 24 h and 2–4 days creatinine clearance, with only one study reporting each outcome. The trial of inhaled NO^12^ was found to be at high risk of allocation concealment. The trial of sodium nitroprusside showed no treatment effect on AKI.

The GRADE assessment judged the certainty of the evidence of the effect estimate for AKI and RRT as low and 30-day mortality as moderate (Supplementary Table 15).

### Interventions with evidence of adverse effects

*Statins* were evaluated in two trials with 2839 participants. No trial was considered high quality. Statin therapy significantly increased mortality (RR: 3.63 [1.01; 13.03]; *P*=0.05; *I*^2^=0%). There was no effect on secondary outcomes. All the trials were performed in cardiac surgery cohorts. One trial at low risk of allocation concealment bias demonstrated no treatment effect on primary or secondary outcomes.

*Spironolactone* (other diuretics intervention group) was evaluated in two trials with 706 participants. Neither study was considered to be high quality. The only trial to report AKI events demonstrated a statistically significant increase in the risk of AKI (RR: 1.49 [1.05–2.14]; *P*=0.03; *I*^2^=N/A). There was no significant treatment effect on mortality. The GRADE assessment judged the certainty of the evidence of the effect estimate for AKI and RRT as very low and 30-day mortality as low (Supplementary Table 16).

*Restrictive vs liberal fluid therapy* was evaluated in 16 RCTs with 10 430 recipients. No study was found to be of high quality. Restrictive compared with liberal fluid therapy resulted in a significant increase in the risk of AKI (RR: 1.64 [1.29–2.09]; *P*<0.001; *I*^2^=0%), but not mortality. Meta-analysis of secondary endpoints demonstrated a significant reduction in 24 h urine output (MD: –0.65 [–0.78 to –0.52]; *P*<0.001; *I*^2^=N/A).

Excluding studies at high risk of allocation concealment eliminated any statistically significant treatment effect on AKI, but did not change the effect estimate for 24 h urine output.

In the general surgery subgroup, restrictive fluid strategies increased the frequency of AKI and sepsis, and reduction in urine output and ICU length of stay. There was no significant treatment effect on mortality. The GRADE assessment judged the certainty of the evidence of the effect estimate for AKI and RRT as moderate and 30-day mortality as low (Supplementary Table 17).

### Interventions with evidence of neither significant renoprotective effects nor harm

In the primary analysis, there were no significant treatment effects on mortality or risk of AKI for the following interventions: ACE inhibitors, dopamine agonists, erythropoietin, glycaemic control, goal-directed fluid therapy, loop diuretics, *N*-acetylcysteine, and corticosteroid therapy (Supplementary Table 3).

With respect to secondary outcomes, *ACE inhibitors* increased 24 h creatinine clearance and perioperative bleeding. *Dopamine agonists* also increased 24 h creatinine clearance. Conversely, *goal-directed fluid therapy* and *loop diuretics* reduced postoperative creatinine clearance. *N-acetylcysteine* reduced RRT and increased perioperative blood loss. *Osmotic diuretics*, *sodium bicarbonate*, *statins*, *volatile anaesthesia vs propofol anaesthesia*, miscellaneous interventions, and miscellaneous antioxidants did not demonstrate significant treatment effects on any of the outcomes.

## Discussion

### Main findings

An updated systematic review of pharmacological strategies to prevent AKI in people undergoing surgery demonstrated:

(i) ANP analogues are renoprotective and result in important reductions in mortality and frequency of RRT. These effects were consistent across the cardiac surgery and vascular surgery subgroups, and in sensitivity analyses restricted to studies at low risk of bias.
(ii) Levosimendan was shown to reduce mortality, AKI, and RRT in both the primary and analysis and in cardiac surgery cohorts.
(iii) The use of vasopressors reduced AKI in the primary analysis, with reductions in AKI, bleeding, and ICU stay in general surgery cohorts.
(iv) Nitric oxide donors, alpha-2-agonists, and calcium channel blockers reduced AKI in primary analyses, but these effects were not observed after exclusion of studies at low or uncertain risk of bias.
(v) Restrictive fluid therapy and the aldosterone agonist spironolactone increased the frequency of AKI.
(vi) Despite significant research activity in this field with 156 new trials identified since the original Cochrane review was published in 2013, overall, only 10% of the trials were considered to be of high quality. These results demonstrate clinical progress towards improved renoprotection in these vulnerable cohorts, but underscore the need for high-quality trials to address remaining areas of uncertainty.

### Clinical importance

This review identified pharmacological interventions that are renoprotective in surgical patients. First, ANP appears to have generalisable renoprotective effects in people undergoing surgery. Most of the trials identified in the current review were undertaken in Japan. Whether these findings will translate into other ethnicities is unclear. This is an important question to be addressed by future research. Currently, ANPs for I.V. use are not licenced or manufactured in Europe. Second, both vasopressors and the inodilator levosimendan reduced AKI and RRT. The comparative risks and benefits of these approaches are unclear. The evidence for levosimendan was derived from cardiac surgery cohorts, whereas the benefits of vasopressors were most evident in general surgery cohorts, where they also reduced bleeding. Anecdotal evidence of variation in care for the ‘pressure *vs* flow’ approaches to perioperative care and the low-to-moderate certainty of evidence for both strategies identified in this review argue for a trial of the two approaches. Third, the study confirmed again that pharmacological strategies that remain in common clinical use for renoprotection have no demonstrable clinical benefits. These include dopamine agonists, loop diuretics, osmotic diuretics, goal-directed fluid therapy, and corticosteroids. In addition, these analyses highlight a disconnection between interventions that are effective at changing creatinine clearance and those with consistent treatment effects on AKI, mortality, or RRT (ANPs, vasopressors, and inodilators). A research question that arises is whether evaluating the effects of renoprotective interventions on measures of renal function, particularly in early phase trials, is the correct approach. This is an important consideration in future trial design.

Novel AKI biomarkers have the potential to overcome this limitation; however, we did not identify many trials in searches that used these, perhaps a reflection of uncertainty as to their specificity and relatively high cost.

### Strengths and weaknesses

The review updated an existing Cochrane report, used Cochrane methodology, followed a pre-specified protocol that was published in advance, and summarised the quality of the evidence using the GRADE approach. The broad scope of the review provides useful mechanistic insights, for example by contrasting the effects of pressure *vs* flow strategies, or the absence of clinical effectiveness for interventions that target urine output, or renal function, as opposed to injury. The review was limited by the quality of the studies and by the relatively small numbers of patients included in important subgroups and in sensitivity analyses. These limitations, as highlighted by the GRADE assessment, preclude firm recommendations as to effective treatments, and importantly, as to whether the positive treatment effects we observed are truly generalisable or limited to specific patient cohorts.

## Conclusions

The limitations of the analyses notwithstanding, this comprehensive review has identified new therapeutic approaches to renoprotection in people undergoing surgery, has identified new knowledge gaps, and provides a useful framework for future research.

## Authors' contributions

Study conception and protocol design: GJM, SP

Data acquisition: SP, GO, WM, MR, RA, MW, ST

Data analysis/interpretation: SP, FL, GJM

Drafting of paper: SP, GJM, FL

Study supervision: GJM

All authors had full access to all of the data in the study and take responsibility for the integrity of the data and the accuracy of the data analysis.

## Declarations of interest

GJM is supported by the 10.13039/501100000274British Heart Foundation (RG/13/6/29947, CH/12/1/29419, and AA/18/3/34220). MR is a 10.13039/501100000272National Institute for Health Research clinical lecturer. RA and SP are supported by the Leicester NIHR Biomedical Research Centre.

## Funding

Leicester National Institute for Health Research Biomedical Research Centre; 10.13039/501100000274British Heart Foundation (CH/12/1/29419 and AA18/3/34220).

## References

[1] Zacharias M., Mugawar M., Herbison G.P. (2013). Interventions for protecting renal function in the perioperative period. Cochrane Database Syst Rev.

[2] Adabag A.S., Ishani A., Bloomfield H.E., Ngo A.K., Wilt T.J. (2009). Efficacy of N-acetylcysteine in preventing renal injury after heart surgery: a systematic review of randomized trials. Eur Heart J.

[3] Ring A., Morris T., Wozniak M. (2017). A phase I study to determine the pharmacokinetic profile, safety and tolerability of sildenafil (Revatio®) in cardiac surgery: the REVAKI-1 study. Br J Clin Pharmacol.

[4] Kidney Disease: Improving Global Outcomes (2012). KDIGO clinical practice guideline for acute kidney injury. Kidney Int Supple.

[5] Patel N.N., Rogers C.A., Angelini G.D., Murphy G.J. (2011). Pharmacological therapies for the prevention of acute kidney injury following cardiac surgery: a systematic review. Heart Fail Rev.

[6] Higgins J., Green Se (2011). Cochrane Handbook for systematic reviews of interventions. https://handbook-5-1.cochrane.org/.

[7] Suraj P., Roman M., Abbasciano R., Murphy G. (2019). Interventions for the prevention of renal injury in surgical patients. A systematic literature review and meta-analysis. PROSPERO 2018 CRD42018115851. PROSPERO: international prospective register of systematic reviews. https://www.crd.york.ac.uk/prospero/display_record.php?RecordID=115851.

[8] Moher D., Liberati A., Tetzlaff J., Altman D.G. (2009). Preferred reporting Items for systematic reviews and meta-analyses: the PRISMA statement. BMJ.

[9] Higgins J.P.T., Thomas J., Chandler J. Cochrane Handbook for systematic reviews of interventions. http://www.training.cochrane.org/handbook.

[10] Guyatt G.H., Oxman A.D., Kunz R. (2008). Going from evidence to recommendations. BMJ.

[11] Guyatt G.H., Oxman A.D., Vist G.E. (2008). GRADE: an emerging consensus on rating quality of evidence and strength of recommendations. BMJ.

[12] Lei C., Berra L., Rezoagli E. (2018). Nitric oxide decreases acute kidney injury and stage 3 chronic kidney disease after cardiac surgery. Am J Respir Crit Care Med.

