# Supplementary material for: Pharmacological interventions for the prevention of renal injury in surgical patients: a systematic literature review and meta-analysis
Source: Br J Anaesth. 2020 Aug 20;126(1):131–8. doi: 10.1016/j.bja.2020.06.064 (PMC7844346; doi:10.1016/j.bja.2020.06.064)
Supplement: Multimedia component 1 [file mmc1.pdf]

Interventions for the Prevention of Renal Injury in Surgical Patients. A Systematic Literature Review and Meta-analysis.

Suraj Pathak MBChB[1], Guido Olivieri MD[1], Walid Mohamed MBBCh[1], Riccardo Abbasciano MD[1], Marius Roman MD[1], Sara Tomassini MBBS[1], Florence Lai MPhil[1], Marcin Wozniak [1] and Gavin J Murphy MD[1].

1. Department of Cardiovascular Sciences and National Institute for Health Research Leicester Biomedical Research Unit in Cardiovascular Medicine, University of Leicester, Leicester, LE3 9QP, United Kingdom.

Supplementary Information

Search Strategies Employed

Search strategy for MEDLINE (2012 to 23rd Aug 2019)

1 exp Kidney Failure/ or exp Kidney Failure Acute/ or exp Kidney Failure Chronic/ or exp Kidney Function Tests/ or exp Glomerular Filtration Rate/ or exp Renal Circulation/ or exp Renal Plasma Flow/ or exp Renal Insufficiency/ or kidney.ti,ab. or (glomerul* adj3 filtration).mp. or (renal adj3 (failure or protect* or function*)).mp. or kidney function test*.mp. or renal function test*.mp. or free water clearance.mp. or fractional excretion of sodium.mp. or (urine adj3 (output or flow)).mp.

2 exp Angiotensin Converting Enzyme Inhibitors/ or exp Fluid Therapy/ or exp Infusions Intravenous/ or exp Angiotensin Converting Enzyme Inhibitors/ or exp diuretics/ or exp mannitol/ or exp Furosemide/ or exp Dopamine/ or exp Dopamine Agonists/ or (diuretic* or mannitol or frusemide or furosemide).mp. or (fluid* adj3 therap*).mp. or (intravenous adj3 fluid*).mp. or hydration.ti,ab. or angiotensin converting enzyme inhibitor*.mp. or ACE inhibitor*.mp. or dopamin*.ti,ab. or exp Vasodilator Agents/ or exp Nitrates/ or exp Nitroglycerin/ or (Nitroglycerin* or NTG or (glyceryl adj3 trinitrate*) or Trinitrin or Anginine or Isosorbide dinitrate or Isosorbide mononitrate or (isosorbide adj3 (dinitrate or mononitrate)) or Isoket or GTN).mp. or exp Phosphodiesterase Inhibitors/ or exp VASODILATION/de or (Sildenafil or sildenafil* or sildefil or sildefil* or Phosphodiesterase Inhibitors or Phosphodiesterase 5 Inhibitors or phophodiesterases inhibitors or PDE5 or desmethyl sildenafil or desmethylsildenafil or acetildenafil or homosildenafil or Viagra or viagra* or vigain or vizarsin or revatio or zwagra) .mp. [mp=title, original title, abstract, name of substance, mesh subject heading]

3 exp Perioperative Care/ or exp Intraoperative Period/ or exp Intraoperative Care/ or exp Intraoperative Complications/ or (peri? operativ* or intra?operativ*).ti,ab.

4 1 and 2 and 3

5 reno?protect*.af.

6 4 or 5 (2513)

7 ((randomized controlled trial or controlled clinical trial).pt. or randomized.ab. or placebo.ab. or clinical trials as topic.sh. or randomly.ab. or trial.ti.) not (animals not (humans and animals)).sh.

8 6 and 7

Search strategy for EMBASE (2012 to23rd Aug 2019)

1 exp kidney failure/ or exp kidney failure/ or exp kidney function test/ or exp glomerulus filtration rate/ or exp kidney circulation/ or exp kidney clearance/ or exp kidney plasma flow/ or exp urine flow rate/ or exp urine volume/ or kidney.ti,ab. or (glomerul* adj3 filtration).mp. or (renal adj3 (failure or protect* or function*)).mp. or kidney function test*.mp. or renal function test*.mp. or free water clearance.mp. or fractional excretion of sodium.mp. or (urine adj3 (output or flow)).mp.

2 exp dipeptidyl carboxypeptidase inhibitor/ or exp fluid therapy/ or intravenous drug administration/ or exp diuretic agent/ or exp diuretic agent/ or exp mannitol/ or exp furosemide/ or exp dopamine/ or exp dopamine receptor stimulating agent/ or (diuretic* or mannitol or frusemide or furosemide).mp. or (fluid* adj3 therap*).mp. or (intravenous adj3 fluid*).mp. or hydration.ti,ab. or angiotensin converting enzyme inhibitor*.mp. or ACE inhibitor*.mp. or dopamin*.ti,ab. or nitrate/ or glyceryl trinitrate/ or (nitroglycerin* or NTG or (glyceryl adj3 trinitrate*) or Trinitrin or Anginine or

Isosorbide dinitrate or Isosorbide mononitrate or (isosorbide adj3 (dinitrate or mononitrate)) or Isoket or GTN).ti,ab. or exp Vasodilator Agents/ or exp Phosphodiesterase Inhibitors/ or exp VASODILATION/de or (Sildenafil or sildenafil* or sildefil or sildefil* or Phosphodiesterase Inhibitors or Phosphodiesterase 5 Inhibitors or phophodiesterases inhibitors or PDE5 or desmethyl sildenafil or desmethylsildenafil or acetildenafil or homosildenafil or Viagra or viagra* or vigain or vizarsin or revatio or zwagra) .mp. [mp=title, original title, abstract, name of substance, mesh subject heading]

3 exp perioperative period/ or exp intraoperative period/ or exp peroperative care/ or (peri?operativ* or intra?operativ*).ti,ab.

4 1 and 2 and 3

5 reno?protect*.ti,ab.

6 4 or 5

7 (placebo.sh. or controlled study.ab. or random*.ti,ab. or trial*.ti,ab. or ((singl* or doubl* or trebl* or tripl*) adj3 (blind* or mask*)).ti,ab.) not (animals not (humans and animals)).sh.

8 6 and 7

Search strategy for CENTRAL (2012 to 23rd Aug 2019)

#1 MeSH descriptor Acute Kidney Injury explode all trees

#2 MeSH descriptor Kidney Failure, Chronic explode all trees

#3 MeSH descriptor Kidney Function Tests explode all trees

#4 MeSH descriptor Glomerular Filtration Rate explode all trees

#5 MeSH descriptor Renal Circulation explode all trees

#6 MeSH descriptor Renal Plasma Flow, Effective explode all trees

#7 MeSH descriptor Renal Insufficiency explode all trees

#8 kidney

#9 glomerul* near filtration

#10 renal near (failure or protect* or function*)

#11 kidney function test*

#12 renal function test*

#13 free water clearance

#14 (fractional excretion) of sodium

#15 urine near (output or flow)

#16 (#1 OR #2 OR #3 OR #4 OR #5 OR #6 OR #7 OR #8 OR #9 OR #10 OR #11 OR #12 OR #13 OR #14 OR #15)

#17 MeSH descriptor Angiotensin-Converting Enzyme Inhibitors explode all trees

#18 diuretic* or mannitol or frusemide or furosemide

#19 MeSH descriptor Fluid Therapy explode all trees

#20 fluid* near therap*

#21 MeSH descriptor Infusions, Intravenous explode all trees

#22 (intravenous near fluid*) or hydration

#23 angiotensin converting enzyme inhibitor*

#24 ACE inhibitor*

#25 MeSH descriptor Diuretics explode all trees

#26 MeSH descriptor Mannitol explode all trees

#27 MeSH descriptor Furosemide explode all trees

#28 MeSH descriptor Dopamine explode all trees

#29 MeSH descriptor Dopamine Agonists explode all trees

#30 dopamin*

#31 MeSH descriptor: [Nitrates] explode all trees

#32 MeSH descriptor: [Nitroglycerin] explode all trees

#33 (Nitroglycerin* or NTG or (glyceryl near trinitrate*) or Trinitrin or Anginine or Isosorbide dinitrate or Isosorbide mononitrate or(isosorbide near (dinitrate or mononitrate)) or Isoket or GTN

#34 MeSH descriptor sildenafil Citrate explode all trees

#35 Sildenafil or sildenafil* or sildefil or sildefil* or Phosphodiesterase Inhibitors or Phosphodiesterase 5 Inhibitors or phophodiesterases inhibitors or PDE5 or desmethyl sildenafil or desmethylsildenafil or acetildenafil or homosildenafil or Viagra or viagra* or vigain or vizarsin or revatio or zwagra

#36 (#16 OR #17 OR #18 OR #19 OR #20 OR #21 OR #22 OR #23 OR #24 OR #25 OR #26 OR #27 OR

#28 OR #29 OR #30 OR #31 OR #32 OR #33 OR #34 OR #35)

#37 MeSH descriptor Perioperative Care explode all trees

#38 MeSH descriptor Intraoperative Care explode all trees

#39 MeSH descriptor Intraoperative Complications explode all trees

#40 MeSH descriptor Intraoperative Period explode all trees

#41 perioperativ* or intraoperativ*

#42 (#37 OR #38 OR #39 OR #40 OR #41)

#43 (#16 AND #36 AND #42)

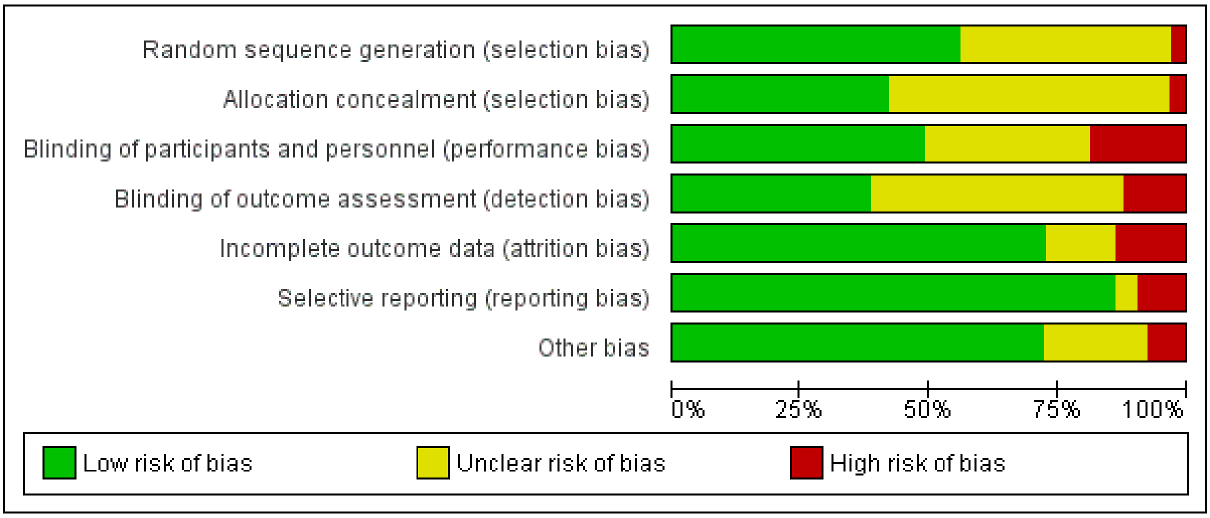

eFigure 1: Risk of Bias Summary

eFigure 2: Risk of Bias graph for included studies

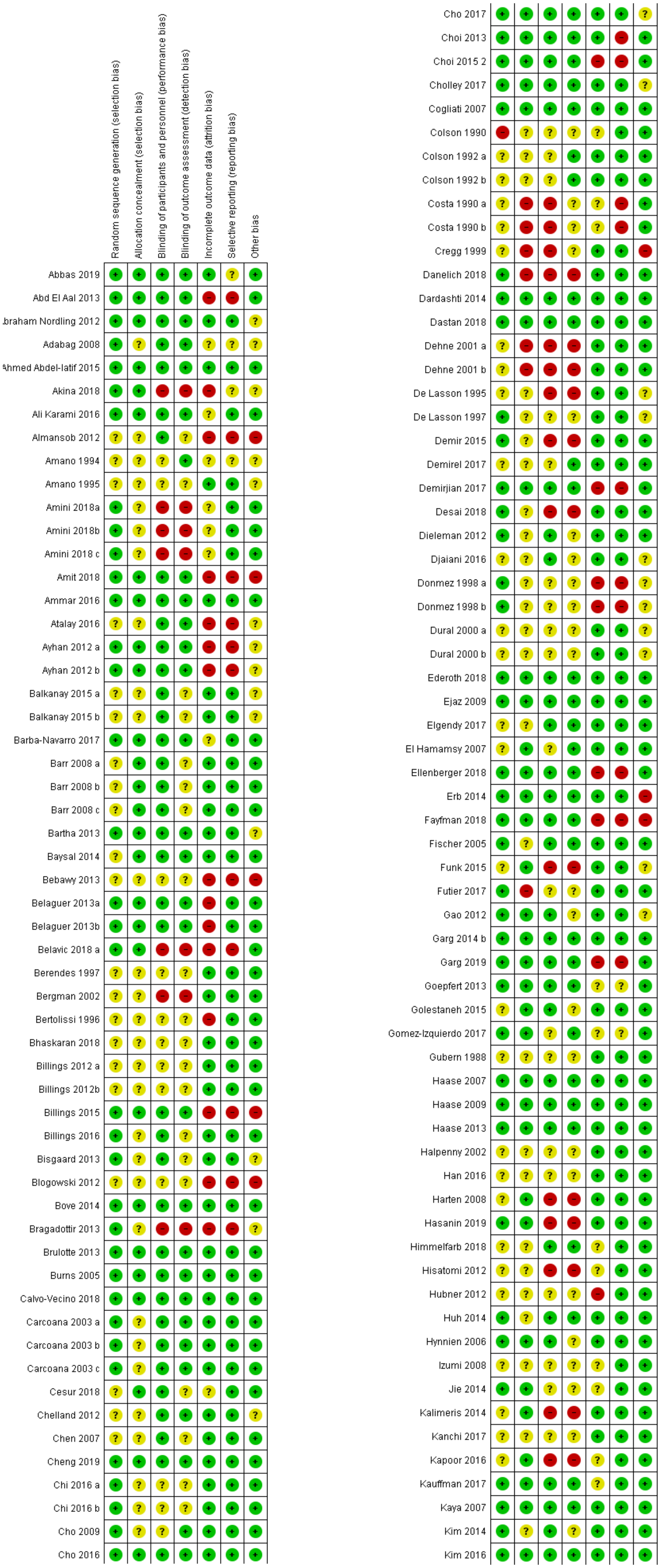

Kishimoto 2018	?	?	−	−	+	+	+
Kramer 2002	+	+	+	+	+	+	−
Kristeller 2013	+	?	+	?	+	+	+
Kulka 1996	?	+	+	+	+	+	?
Kumar 2015	+	?	?	?	−	−	?
Landoni 2017	+	+	+	+	+	+	?
Landoni 2019	+	+	−	?	+	+	+
Lassnigg 2000 a	+	+	+	?	−	+	+
Lassnigg 2000 b	+	+	+	?	−	+	+
Lei 2018	?	?	+	+	?	+	+
Levin 2012	+	?	?	?	+	+	?
Li 2017	+	+	+	?	+	+	−
Licker 1996	?	+	+	?	+	+	?
Liu 2016	+	?	−	−	+	+	+
Loef 2004	?	?	+	+	+	−	?
Luo 2013 a	?	?	?	?	+	+	?
Luo 2013 b	?	?	?	?	+	+	?
Luo 2017	+	+	?	?	+	+	+
Mahesh 2008	?	?	+	?	−	+	+
Mansourian 2015	+	?	+	?	+	+	+
Marathias 2006	?	−	?	?	+	+	?
Matot 2012	+	?	−	+	+	+	+
Matot 2013	+	?	−	+	−	+	+
Mazer 2018	+	?	−	−	−	+	+
McGuiness 2013	+	+	+	+	?	+	+
Mehta 2017	+	+	+	?	+	+	+
Mentzer 2007	?	?	+	?	+	+	+
Mikor 2015	?	+	−	?	?	+	+
Mitaka 2008	−	+	+	?	+	+	?
Mitaka 2017	+	?	+	+	+	+	+
Moppett 2015	+	+	−	+	+	+	+
Morariu 2005	?	?	+	+	+	+	+
Morgera 2002	?	?	?	?	+	+	?
Mori 2014	+	+	+	+	−	+	+
Muralidhar 2018	?	?	?	?	+	+	+
Myles 1993	?	?	?	?	+	?	?
Myles 2018	+	?	−	+	+	+	+
Nan Hai 2013	+	+	?	?	+	+	+
Nicholson 1996	+	?	+	?	+	+	?
Nouri-Majalan 2009	?	?	+	?	+	+	+
Osawa 2016	+	+	−	+	+	+	+
Park 2014	?	?	?	?	+	+	+
Park 2016	+	?	+	+	+	+	+
Park 2017	+	?	?	?	+	+	+
Parke 2015	+	+	−	−	+	+	+
Parks 1994	−	?	−	?	+	+	?
Peng 2013	+	+	?	?	?	+	+
Peng 2014	+	+	?	?	+	+	+
Perez 2002	?	?	+	?	+	+	?
Pestana 2014	+	+	?	?	?	+	+
Phan 2014	+	+	?	+	+	+	+
Piljic 2015	?	?	?	?	?	?	?
Prasad 2010	+	?	?	?	+	+	?
Pretorius 2012 a	?	?	+	?	+	+	+
Pretorius 2012 b	?	?	+	?	+	+	+
Prowle 2012	+	+	+	+	?	+	?
Puckett 2017	+	?	−	−	+	+	−
Pull Ter Gunne 1990	?	?	?	?	−	−	−
Raghava 2015	+	?	?	?	+	+	+
Ristikankare 2006	?	?	+	?	+	+	?
Ristikankare 2012	?	?	+	?	+	+	+
Russo 2014	+	+	?	+	+	+	+
Ryckwaert 2001	?	?	+	?	+	+	?
Salah 2013	?	+	+	+	?	+	+
Santana-Santos 2014	+	?	+	+	+	+	+
Saratzis 2018	+	?	−	+	+	+	+
Schmid 2016	+	?	?	?	?	+	+
Schmidt 2017	+	?	+	+	+	+	+
Sezai 2000	?	?	?	?	+	+	?
Sezai 2006	?	?	?	?	+	+	+
Sezai 2007	?	?	?	?	+	+	+
Sezai 2009	+	+	+	?	?	+	+
Sezai 2011	+	+	?	+	+	+	+
Sezai 2013	?	?	?	+	?	+	+
Shah 2014	−	?	+	?	+	+	+
Shahbazi 2017	+	?	+	?	+	+	+
Sharma 2014	−	?	+	?	+	+	+
Shehata 2012	?	+	−	−	+	+	+
Shim 2007	+	?	?	?	+	+	+
Sisillo 2008	+	?	+	+	+	+	+
Soh 2016	+	+	+	+	+	+	?
Soliman 2016	?	?	+	?	+	+	+
Soliman 2017	+	?	+	?	+	+	+
Soliman 2019	+	?	+	?	+	+	+
Song 2009	+	+	+	+	+	+	−
Song 2013 1	−	?	+	+	+	+	+
Song 2013 2	+	?	?	?	−	+	+
Song 2015	+	+	+	+	+	+	+
Song 2018	+	+	+	+	+	+	+
Stoppe 2013	?	?	−	?	+	+	+
Tang 1999	?	?	?	?	+	+	+
Tasanarong 2013	?	?	?	?	?	?	?
Thompson 1986	?	?	?	?	+	+	+
Turner 2008	?	+	−	?	+	+	+
Turner 2014	?	?	−	−	+	+	?
Urzua 1992	−	?	?	?	+	+	+
Van Samkar 2015	+	?	+	?	?	+	+
Wahbah 2000 a	+	+	?	?	+	+	+
Wahbah 2000 b	+	+	?	?	+	+	−
Wahbah 2000 c	+	+	?	?	+	+	−
Wahby 2016	+	?	?	?	+	+	+
Wang 2013	?	?	?	?	?	?	?
Wang 2014	+	+	+	+	+	+	+
Wasowicz 2018	+	+	−	?	−	+	+
Weinberg 2017	?	+	?	?	+	+	+
Weinberg 2019	+	+	+	+	?	+	+
Welch 1995	?	?	−	?	+	+	−
Wijeysundera 2007	+	+	+	+	+	+	+
Wijnen 2002	?	?	?	?	+	+	+
Witczak 2008	?	?	+	?	+	+	−
Woo 2002	?	?	?	?	+	?	−
Wu 2017 1a	?	?	−	−	+	+	+
Wu 2017 1b	?	?	−	−	+	+	+
Wu 2017 2	?	?	?	?	+	+	+
Xu 2017	+	+	?	+	+	+	+
Yavuz 2002 1	?	?	?	?	+	+	?
Yavuz 2002 2a	?	?	?	?	+	+	+
Yavuz 2002 2b	?	?	?	?	+	+	+
Yin 2018	+	+	?	?	?	+	+
Yoo 2014	+	+	−	+	+	+	+
Yu 2018	?	?	?	?	+	+	+
Zadeh 2016	?	?	−	−	+	+	+
Zakhaleva 2012	+	?	−	−	−	+	+
Zanardo 1993 a	?	?	?	?	+	+	+
Zanardo 1993 b	?	?	?	?	+	+	+
Zangrillo 2018	+	+	+	+	−	+	−
Zhai 2017	+	?	+	?	+	+	+
Zhao 2017	+	?	−	−	+	+	+
Zhao 2018	?	?	+	?	+	+	+
Zheng 2013	+	+	?	+	+	+	+
Zheng 2016	?	?	+	?	+	+	+

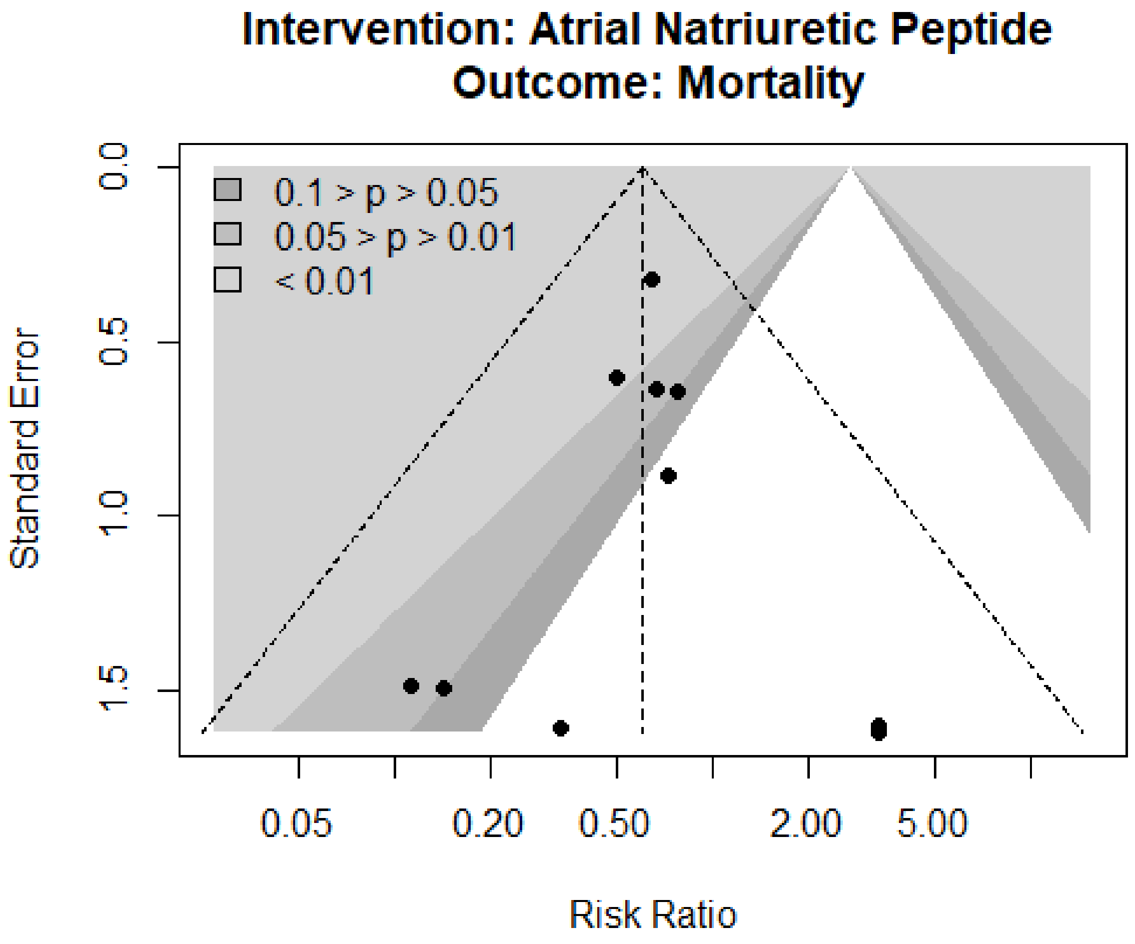

eFigure3: Funnel plot for studies investigating Atrial Natriuretic Peptide reporting Mortality.

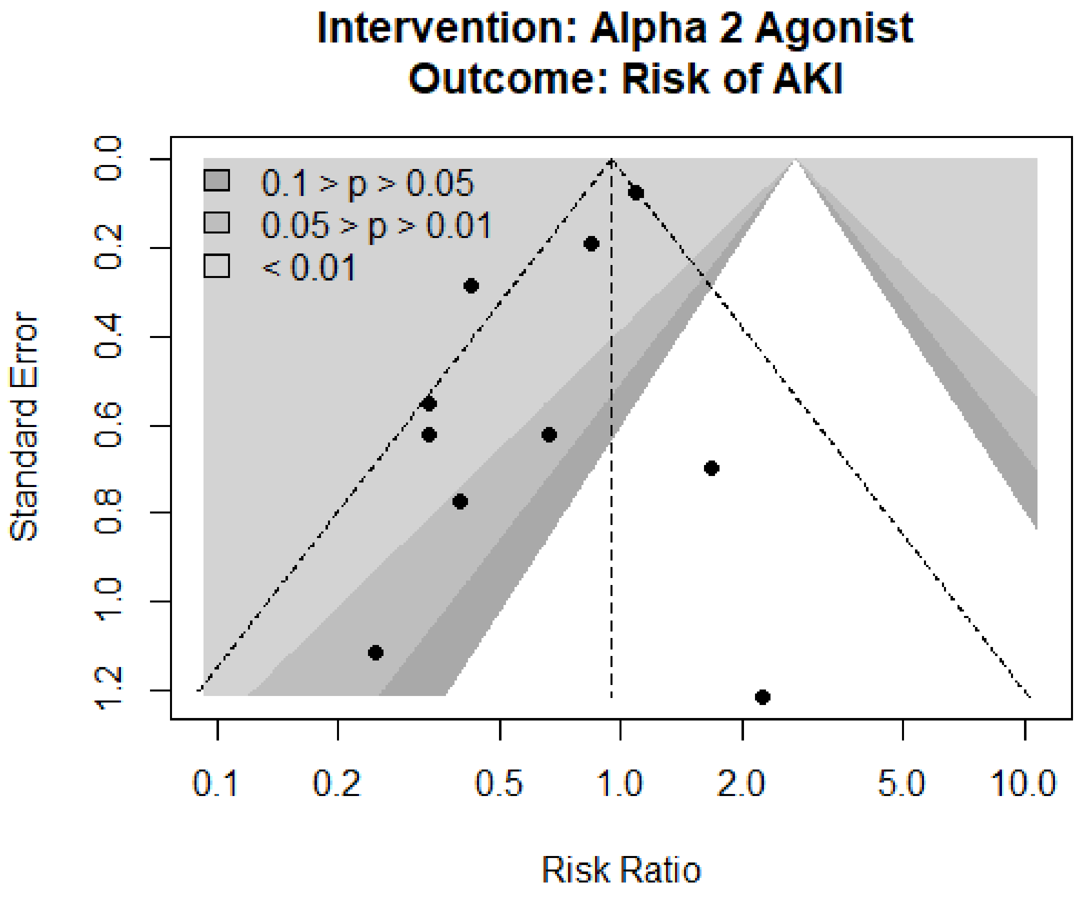

eFigure4: Funnel plot for studies investigating alpha 2 agonists reporting risk of AKI.

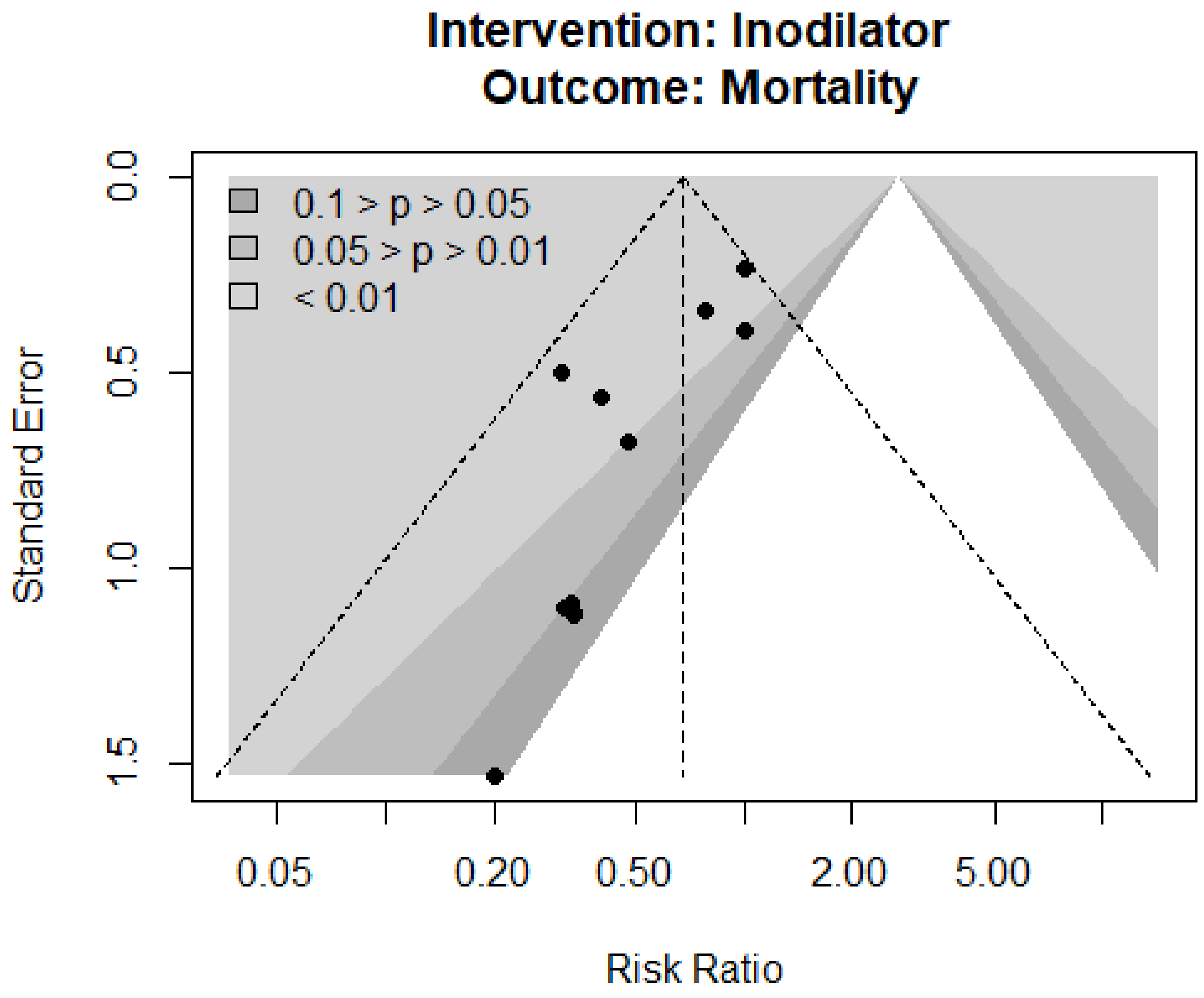

eFigure5: Funnel plot for studies investigating Inodilators reporting Mortality.

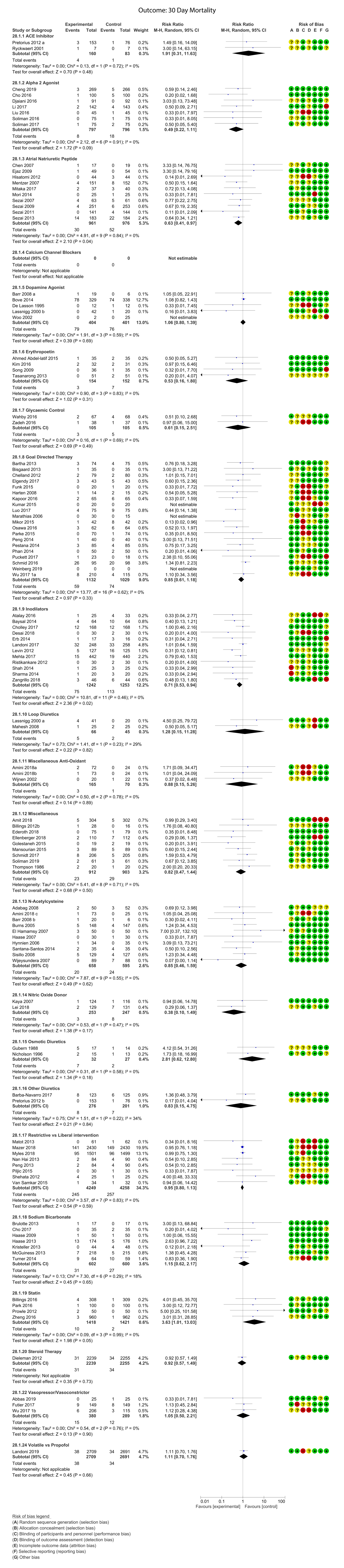

eFigure 6: Forest plot of all interventions for 30 day mortality.

Outcome: Risk of AKI

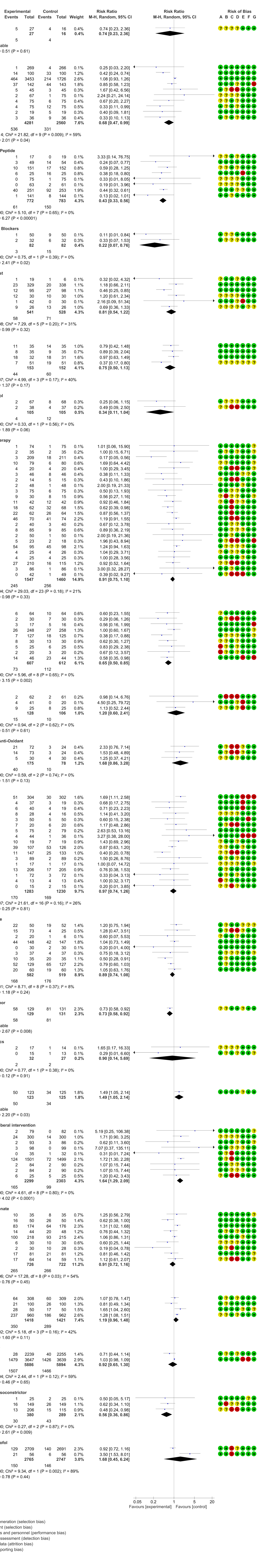

eFigure 7: Forest plot of all interventions for risk of Acute Kidney Injury (AKI)

Outcome: Risk of Dialysis / RRT

Study or Subgroup	Experimental Events	Experimental Total	Control Events	Control Total	Weight	Risk Ratio M-H, Random, 95% CI
**28.14.1 ACE Inhibitor**						
Billings 2012 a	1	87	0	27	0.2%	0.95 [0.04, 22.78]
**Subtotal (95% CI)**		**87**		**27**	**0.2%**	**0.95 [0.04, 22.78]**
Total events	1		0			
Heterogeneity: Not applicable						
Test for overall effect: Z = 0.03 (P = 0.98)						
**28.14.2 Alpha 2 Agonist**						
Balkanay 2015 a	1	31	1	14	0.2%	0.45 [0.03, 6.71]
Balkanay 2015 b	1	29	0	14	0.2%	1.50 [0.06, 34.66]
Cho 2016	1	100	5	100	0.3%	0.20 [0.02, 1.68]
Djaiani 2016	0	91	2	92	0.2%	0.20 [0.01, 4.15]
Garg 2014 b	47	3453	17	1726	3.4%	1.38 [0.80, 2.40]
Li 2017	37	142	44	143	5.3%	0.85 [0.58, 1.23]
Soliman 2017	3	75	11	75	0.9%	0.27 [0.08, 0.94]
**Subtotal (95% CI)**		**3921**		**2164**	**10.6%**	**0.76 [0.46, 1.27]**
Total events	90		80			
Heterogeneity: Tau² = 0.13; Chi² = 9.19, df = 6 (P = 0.16); I² = 35%						
Test for overall effect: Z = 1.05 (P = 0.29)						
**28.14.3 Atrial Natriuretic Peptide**						
Ejaz 2009	2	49	3	54	0.5%	0.73 [0.13, 4.21]
Mitaka 2017	1	37	3	40	0.3%	0.36 [0.04, 3.31]
Sezai 2007	0	63	2	61	0.2%	0.19 [0.01, 3.96]
Sezai 2009	9	251	35	253	2.4%	0.26 [0.13, 0.53]
Sezai 2011	1	141	8	144	0.4%	0.13 [0.02, 1.01]
Sezai 2013	0	183	7	184	0.2%	0.07 [0.00, 1.17]
**Subtotal (95% CI)**		**724**		**736**	**3.9%**	**0.26 [0.15, 0.47]**
Total events	13		58			
Heterogeneity: Tau² = 0.00; Chi² = 2.88, df = 5 (P = 0.72); I² = 0%						
Test for overall effect: Z = 4.51 (P < 0.00001)						
**28.14.4 Calcium Channel Blockers**						
Cho 2009	1	50	9	50	0.4%	0.11 [0.01, 0.84]
Park 2017	1	32	2	32	0.3%	0.50 [0.05, 5.24]
Witczak 2008	0	10	3	10	0.2%	0.14 [0.01, 2.45]
**Subtotal (95% CI)**		**92**		**92**	**0.8%**	**0.19 [0.05, 0.75]**
Total events	2		14			
Heterogeneity: Tau² = 0.00; Chi² = 0.99, df = 2 (P = 0.61); I² = 0%						
Test for overall effect: Z = 2.38 (P = 0.02)						
**28.14.5 Dopamine Agonist**						
Barr 2008 a	1	19	1	6	0.2%	0.32 [0.02, 4.32]
Bove 2014	241	329	218	338	9.2%	1.14 [1.03, 1.26]
Kanchi 2017	1	30	1	30	0.2%	1.00 [0.07, 15.26]
Lassnigg 2000 b	1	42	0	20	0.2%	1.47 [0.06, 34.46]
Myles 1993	9	26	12	26	2.6%	0.75 [0.38, 1.47]
Welch 1995	2	15	8	17	0.8%	0.28 [0.07, 1.13]
**Subtotal (95% CI)**		**461**		**437**	**13.2%**	**0.91 [0.61, 1.35]**
Total events	255		240			
Heterogeneity: Tau² = 0.06; Chi² = 6.44, df = 5 (P = 0.27); I² = 22%						
Test for overall effect: Z = 0.48 (P = 0.63)						
**28.14.6 Erythropoetin**						
Ahmed Abdel-latif 2015	1	35	1	35	0.2%	1.00 [0.07, 15.36]
Dardashti 2014	1	35	0	35	0.2%	3.00 [0.13, 71.22]
Kim 2016	1	32	3	31	0.3%	0.32 [0.04, 2.94]
Song 2009	3	36	10	35	1.0%	0.29 [0.09, 0.97]
**Subtotal (95% CI)**		**138**		**136**	**1.7%**	**0.42 [0.16, 1.08]**
Total events	6		14			
Heterogeneity: Tau² = 0.00; Chi² = 2.28, df = 3 (P = 0.52); I² = 0%						
Test for overall effect: Z = 1.79 (P = 0.07)						
**28.14.7 Glycaemic Control**						
Zadeh 2016	2	38	4	37	0.6%	0.49 [0.09, 2.50]
**Subtotal (95% CI)**		**38**		**37**	**0.6%**	**0.49 [0.09, 2.50]**
Total events	2		4			
Heterogeneity: Not applicable						
Test for overall effect: Z = 0.86 (P = 0.39)						
**28.14.8 Goal Directed Therapy**						
Bartha 2013	1	74	1	75	0.2%	1.01 [0.06, 15.90]
Bisgaard 2013	2	35	2	35	0.4%	1.00 [0.15, 6.71]
Calvo-Vecino 2018	3	209	18	211	1.0%	0.17 [0.05, 0.56]
Chelland 2012	10	79	6	80	1.5%	1.69 [0.64, 4.42]
Elgendy 2017	2	43	4	43	0.6%	0.50 [0.10, 2.59]
Funk 2015	4	20	4	20	0.9%	1.00 [0.29, 3.45]
Kauffman 2017	2	48	1	48	0.3%	2.00 [0.19, 21.33]
Kumar 2015	0	20	0	20		Not estimable
Luo 2017	0	75	1	75	0.2%	0.33 [0.01, 8.05]
Marathias 2006	9	30	8	15	2.3%	0.56 [0.27, 1.16]
Mikor 2015	1	42	1	42	0.2%	1.00 [0.06, 15.47]
Osawa 2016	2	62	0	64	0.2%	5.16 [0.25, 105.34]
Parke 2015	2	70	1	74	0.3%	2.11 [0.20, 22.80]
Peng 2014	1	40	0	40	0.2%	3.00 [0.13, 71.51]
Pull Ter Gunne 1990	0	13	0	13		Not estimable
Schmid 2016	2	95	2	98	0.4%	1.03 [0.15, 7.18]
**Subtotal (95% CI)**		**955**		**953**	**8.6%**	**0.79 [0.51, 1.22]**
Total events	41		49			
Heterogeneity: Tau² = 0.04; Chi² = 13.88, df = 13 (P = 0.38); I² = 6%						
Test for overall effect: Z = 1.06 (P = 0.29)						
**28.14.9 Inodilators**						
Baysal 2014	6	64	10	64	1.5%	0.60 [0.23, 1.55]
Desai 2018	2	30	0	30	0.2%	5.00 [0.25, 99.95]
Erb 2014	3	17	5	16	0.9%	0.56 [0.16, 1.99]
Landoni 2017	24	248	33	258	3.9%	0.76 [0.46, 1.24]
Levin 2012	7	127	18	125	1.8%	0.38 [0.17, 0.88]
Mehta 2017	9	442	16	440	2.0%	0.56 [0.25, 1.25]
Shah 2014	4	25	4	25	0.9%	1.00 [0.28, 3.56]
Sharma 2014	2	20	3	20	0.5%	0.67 [0.12, 3.57]
Zangrillo 2018	4	46	9	44	1.2%	0.43 [0.14, 1.28]
**Subtotal (95% CI)**		**1019**		**1022**	**12.9%**	**0.63 [0.46, 0.85]**
Total events	61		98			
Heterogeneity: Tau² = 0.00; Chi² = 4.84, df = 8 (P = 0.77); I² = 0%						
Test for overall effect: Z = 2.99 (P = 0.003)						
**28.14.10 Loop Diuretics**						
Lassnigg 2000 a	6	41	0	20	0.2%	6.50 [0.38, 109.96]
Mahesh 2008	1	25	0	25	0.2%	3.00 [0.13, 70.30]
**Subtotal (95% CI)**		**66**		**45**	**0.4%**	**4.61 [0.56, 37.82]**
Total events	7		0			
Heterogeneity: Tau² = 0.00; Chi² = 0.13, df = 1 (P = 0.71); I² = 0%						
Test for overall effect: Z = 1.42 (P = 0.16)						
**28.14.11 Miscellaneous Anti-Oxidant**						
Amini 2018a	1	72	0	24	0.2%	1.03 [0.04, 24.42]
Billings 2015	3	30	3	30	0.6%	1.00 [0.22, 4.56]
Nouri-Majalan 2009	5	30	4	30	1.0%	1.25 [0.37, 4.21]
**Subtotal (95% CI)**		**132**		**84**	**1.8%**	**1.14 [0.46, 2.82]**
Total events	9		7			
Heterogeneity: Tau² = 0.00; Chi² = 0.05, df = 2 (P = 0.97); I² = 0%						
Test for overall effect: Z = 0.27 (P = 0.78)						
**28.14.12 Miscellaneous**						
Belaguer 2013a	4	37	3	19	0.8%	0.68 [0.17, 2.75]
Belaguer 2013b	6	40	4	19	1.1%	0.71 [0.23, 2.23]
Billings 2012b	1	28	0	16	0.2%	1.76 [0.08, 40.80]
Ederoth 2018	3	75	2	79	0.5%	1.58 [0.27, 9.19]
Golestaneh 2015	1	19	1	19	0.2%	1.00 [0.07, 14.85]
Himmelfarb 2018	2	107	2	126	0.4%	1.18 [0.17, 8.22]
Kishimoto 2018	1	147	1	133	0.2%	0.90 [0.06, 14.32]
Mansourian 2015	3	89	2	89	0.5%	1.50 [0.26, 8.76]
Morgera 2002	1	17	1	17	0.2%	1.00 [0.07, 14.72]
Thompson 1986	1	20	0	20	0.2%	3.00 [0.13, 69.52]
**Subtotal (95% CI)**		**579**		**537**	**4.2%**	**1.01 [0.55, 1.83]**
Total events	23		16			
Heterogeneity: Tau² = 0.00; Chi² = 1.73, df = 9 (P = 1.00); I² = 0%						
Test for overall effect: Z = 0.02 (P = 0.98)						
**28.14.13 N-Acetylcysteine**						
Adabag 2008	3	50	2	52	0.5%	1.56 [0.27, 8.95]
Ali Karami 2016	16	26	30	30	6.3%	0.62 [0.46, 0.84]
Amini 2018 c	0	73	1	25	0.2%	0.12 [0.00, 2.79]
Barr 2008 a	2	20	1	6	0.3%	0.60 [0.07, 5.53]
Brulotte 2013	0	0	0	0		Not estimable
Burns 2005	1	148	3	147	0.3%	0.33 [0.03, 3.15]
Haase 2007	0	30	2	30	0.2%	0.20 [0.01, 4.00]
Prasad 2010	3	37	4	37	0.7%	0.75 [0.18, 3.12]
Ristikankare 2006	1	40	0	40	0.2%	3.00 [0.13, 71.51]
Sisillo 2008	10	129	6	127	1.4%	1.64 [0.61, 4.38]
Song 2015	3	60	2	60	0.5%	1.50 [0.26, 8.66]
Wijeysundera 2007	1	89	3	88	0.3%	0.33 [0.03, 3.11]
**Subtotal (95% CI)**		**702**		**642**	**10.8%**	**0.68 [0.52, 0.89]**
Total events	40		54			
Heterogeneity: Tau² = 0.00; Chi² = 9.16, df = 10 (P = 0.52); I² = 0%						
Test for overall effect: Z = 2.80 (P = 0.005)						
**28.14.14 Nitric Oxide Donor**						
Lei 2018	4	129	6	131	0.9%	0.68 [0.20, 2.34]
**Subtotal (95% CI)**		**129**		**131**	**0.9%**	**0.68 [0.20, 2.34]**
Total events	4		6			
Heterogeneity: Not applicable						
Test for overall effect: Z = 0.62 (P = 0.54)						
**28.14.15 Osmotic Diuretics**						
Gubern 1988	2	17	1	14	0.3%	1.65 [0.17, 16.33]
Nicholson 1996	0	15	1	13	0.2%	0.29 [0.01, 6.60]
**Subtotal (95% CI)**		**32**		**27**	**0.5%**	**0.90 [0.14, 5.69]**
Total events	2		2			
Heterogeneity: Tau² = 0.00; Chi² = 0.77, df = 1 (P = 0.38); I² = 0%						
Test for overall effect: Z = 0.12 (P = 0.91)						
**28.14.16 Other Diuretics**						
Barba-Navarro 2017	9	123	8	125	1.6%	1.14 [0.46, 2.87]
**Subtotal (95% CI)**		**123**		**125**	**1.6%**	**1.14 [0.46, 2.87]**
Total events	9		8			
Heterogeneity: Not applicable						
Test for overall effect: Z = 0.29 (P = 0.78)						
**28.14.17 Restrictive vs Liberal intervention**						
Abraham Nordling 2012	2	79	0	82	0.2%	5.19 [0.25, 106.38]
Mazer 2018	87	2430	94	2430	6.5%	0.93 [0.70, 1.23]
Myles 2018	13	1501	4	1499	1.1%	3.25 [1.06, 9.93]
Nan Hai 2013	2	84	2	90	0.4%	1.07 [0.15, 7.44]
Peng 2013	2	84	2	90	0.4%	1.07 [0.15, 7.44]
Shehata 2012	0	25	1	25	0.2%	0.33 [0.01, 7.81]
**Subtotal (95% CI)**		**4203**		**4216**	**8.8%**	**1.22 [0.70, 2.11]**
Total events	106		103			
Heterogeneity: Tau² = 0.11; Chi² = 6.18, df = 5 (P = 0.29); I² = 19%						
Test for overall effect: Z = 0.70 (P = 0.48)						
**28.14.18 Sodium Bicarbonate**						
Brulotte 2013	1	17	0	17	0.2%	3.00 [0.13, 68.84]
Cho 2017	0	35	1	35	0.2%	0.33 [0.01, 7.91]
Haase 2009	2	50	2	50	0.4%	1.00 [0.15, 6.82]
McGuiness 2013	7	218	10	215	1.5%	0.69 [0.27, 1.78]
Soh 2016	0	81	0	81		Not estimable
Turner 2014	4	64	5	59	0.9%	0.74 [0.21, 2.62]
**Subtotal (95% CI)**		**465**		**457**	**3.1%**	**0.76 [0.39, 1.49]**
Total events	14		18			
Heterogeneity: Tau² = 0.00; Chi² = 1.12, df = 4 (P = 0.89); I² = 0%						
Test for overall effect: Z = 0.79 (P = 0.43)						
**28.14.19 Statin**						
Billings 2016	5	308	3	309	0.7%	1.67 [0.40, 6.94]
Park 2016	1	100	1	100	0.2%	1.00 [0.06, 15.77]
Prowle 2012	6	50	8	50	1.4%	0.75 [0.28, 2.00]
Zheng 2016	13	960	8	962	1.7%	1.63 [0.68, 3.91]
**Subtotal (95% CI)**		**1418**		**1421**	**4.1%**	**1.22 [0.68, 2.18]**
Total events	25		20			
Heterogeneity: Tau² = 0.00; Chi² = 1.57, df = 3 (P = 0.67); I² = 0%						
Test for overall effect: Z = 0.67 (P = 0.50)						
**28.14.20 Steroid Therapy**						
Garg 2019	145	3647	161	3639	7.6%	0.90 [0.72, 1.12]
**Subtotal (95% CI)**		**3647**		**3639**	**7.6%**	**0.90 [0.72, 1.12]**
Total events	145		161			
Heterogeneity: Not applicable						
Test for overall effect: Z = 0.95 (P = 0.34)						
**28.14.22 Vasopressor/Vasoconstrictor**						
Futier 2017	9	149	9	149	1.7%	1.00 [0.41, 2.45]
**Subtotal (95% CI)**		**149**		**149**	**1.7%**	**1.00 [0.41, 2.45]**
Total events	9		9			
Heterogeneity: Not applicable						
Test for overall effect: Z = 0.00 (P = 1.00)						
**28.14.24 Volatile vs Propofol**						
Landoni 2019	13	2709	12	2691	2.1%	1.08 [0.49, 2.35]
Yoo 2014	2	56	0	56	0.2%	5.00 [0.25, 101.85]
**Subtotal (95% CI)**		**2765**		**2747**	**2.2%**	**1.19 [0.56, 2.53]**
Total events	15		12			
Heterogeneity: Tau² = 0.00; Chi² = 0.94, df = 1 (P = 0.33); I² = 0%						
Test for overall effect: Z = 0.44 (P = 0.66)						

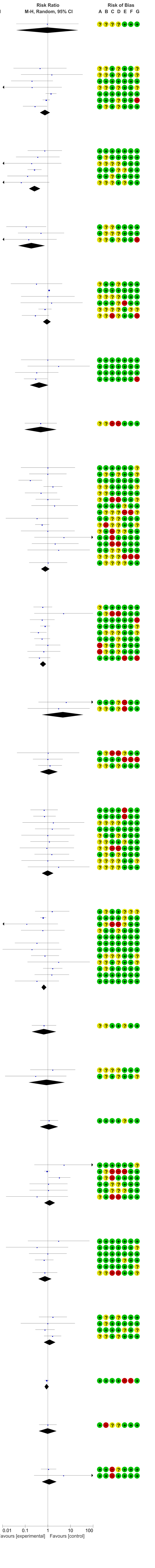

Risk of bias legend
(**A**) Random sequence generation (selection bias)
(**B**) Allocation concealment (selection bias)
(**C**) Blinding of participants and personnel (performance bias)
(**D**) Blinding of outcome assessment (detection bias)
(**E**) Incomplete outcome data (attrition bias)
(**F**) Selective reporting (reporting bias)
(**G**) Other bias

eFigure 8: Forest plot of all interventions for risk of stage 3 AKI / Dialysis.

Study *(Author, Year)*	• Country<br>• Language<br>• Year of the trial completion<br>• Study population size (n)<br>• Inclusion criteria (descriptive)	Exclusion criteria (descriptive)	Demographics (Age between groups)	Comorbidities (CV disease, Cancer, Renal Disease, Anaemia)	Type of Surgery	• Type of Intervention (subtype if available)<br>• Type of Control	Primary Outcomes (list)	Secondary Actual Outcomes (list)
**ACE Inhibitor**								
Billings 2012 a[(1)]	• USA<br>• English<br>• 2012<br>• 87 Patients<br>• Adult patients undergoing cardiac surgery with the use of CPB	left ventricular ejection fraction less than 30%, serum potassium greater than 5.0 mEq L-1, serum creatinine greater than 1.6 mg dL-1, and inability to discontinue current ACE inhibitor or ARB.	Candesartan: 67.0(1.7)<br>Ramipril: 64.4(2.1)<br>Placebo: 66.1(2.1)	CV disease<br>Hypertension	Cardiac Surgery	Candesartan<br>Ramipril<br>Placebo	- plasma t-PA and PAI-1 antigen concentrations	- IL-6, IL-8 and IL-10 concentrations.<br>- postoperative blood loss, transfusion requirements, re-exploration for bleeding, inotropic and vasopressor use, new onset atrial fibrillation, and changes in serum creatinine
Colson 1990[(2)]	• France<br>• English<br>• 1990<br>• 18 Patients<br>• Adult patients undergoing coronary artery bypass surgery	Prior renal dysfunction or cardiac failure	Captopril 60(2)<br>Placebo 56(3)	CAD	Cardiac Surgery	Captopril<br>Placebo	Blood pressure and renal function	- FeNA, FeK, Hemoglobin
Colson 1992 b [(3)]	• France<br>• English<br>• 1992<br>• 24 Patients<br>• Adult patients undergoing abdominal aortic disease	Prior renal dysfunction, cardiac failure, long-term treatment with Ca-channel blockers or CEIs or emergency surgery	Enalapril 58 (4)<br>Control 63 (1)	AAA	Vascular Surgery	Enalapril<br>Control	Blood pressure and renal function	- FeNA, FeK, Hemoglobin

Licker 1996[4]	• Switzerland<br>• English<br>• 1996<br>• 22 Patients<br>• Adult patient undergoing abdominal aortic surgery	Preoperative renal dysfunction, cardiac insufficiency, myocardial infarction within the last 6 months, renal artery stenosis detected on the angiogram or those receiving ACEI therapy	Enalapril 69 (55-81)<br>Placebo 68 (53-79)	AAA	Vascular Surgery	Enalapril<br>Placebo	Renal function and hemodynamic function	Renal vascular flow, creatinine clearance, fractional sodium excretion and protein excretion
Pretorius 2012 a[5]	• USA<br>• English<br>• 2012<br>• 432 Patients<br>• Adult patients undergoing cardiac surgery with the use of CPB	Chronic or paroxysmal (>6 m) AF, LVEF < 30%, emergency surgery, renal failure, coagulopathy and hyperkalemia	Ramipril 58.7 (12.3)<br>Placebo 60.0 (12.0)	Cardiac Disease	Cardiac Surgery	Ramipril<br>Placebo	Incidence of post-operative AF	Hemodynamic function, use of vasopressor, renal failure, post-operative complications, inflammatory biomarkers
Ryckwaert 2001[6]	• France<br>• English<br>• 2001<br>• 14 Patients<br>• Adult patients undergoing coronary artery bypass surgery	ejection fraction < 40% after myocardial infarction, prior renal dysfunction, unstable congestive heart failure, with valvular pathology, or chronically treated with ACEI	IV enalaprilat 60.1 (3.6)<br>Control 66.3 (4.2)	CAD	Cardiac Surgery	IV enalaprilat<br>Control	Hemodynamic function, use of vasopressor and renal failure	Plasma renin activity, arginine vasopressin concentration
**Alpha 2 agonist**								

Ammar 2016[7]	• Egypt<br>• English<br>• 2016<br>• 50 Patients<br>• Adult patients undergoing cardiac surgery	age >75 y, LVEF <55%, preexisting severe LV hypertrophy, cardiomyopathies, Grade II (pseudonormal filling) and Grade III (restrictive filling) diastolic dysfunction, preoperative AF, pericardial disease, drug dependence, cerebrovascular diseases, use of alpha-2 agonists, type I diabetes mellitus, renal disease, significant pulmonary disease, and hepatic insufficiency.	Dexmedetomidine 55.4 (7.1)<br>Control 59.1 (6.2)	Cardiac Disease	Cardiac Surgery	Dexmedetomidin e<br>Control	Cardiac and kidney markers	Inflammatory markers, post-operative complications
Balkanay 2015a [8]	• Turkey<br>• English<br>• 2014<br>• 90 Patients<br>• Adult patients undergoing coronary artery bypass graft surgery	concomitant operation, preoperative renal impairment and diuretic use and no patients with emergency operations	Mean age of patients 60.5 (8.6)	CAD	Cardiac Surgery	Dexmedetomidin e (low dose < 8 mcg kg-1)<br>Placebo	NGAL	Renal function, cardio-respiratory post-operative parameters, post-operative AF, poster operative need of diuretics
Balkanay 2015 b[8]	• Turkey<br>• English<br>• 2014<br>• 90 Patients<br>• Adult patients undergoing coronary artery bypass graft surgery	concomitant operation, preoperative renal impairment and diuretic use and no patients with emergency operations	Mean age of patients 60.5 (8.6)	CAD	Cardiac Surgery	Dexmedetomidin e (high dose > 8 mcg kg-1)<br>Placebo	NGAL	Renal function, cardio-respiratory post-operative parameters, post-operative AF, poster operative need of diuretics
Cheng 2019[9]	• China<br>• English<br>• 2019<br>• 535 Patients<br>• Inclusion Criteria: 65 years or more undergoing scheduled gastro-intestinal laparotomy	Exclusion Criteria: mini-mental state examination scores < 20 or those unable to complete baseline cognitive assessments: factors that might affect	Dexmedetomidine: 71 (67—75<br>Placebo: 70 (68—74<br>Median (IQR)	Corona ry artery disease , hypert ension,	General Surgery	Dexmedetomidine (0.125 ml.kg-1 bolus of study solution<br>15 min before induction of anaesthesia,	cognition on the seventh postoperative day compared with pre-operative cognition	Post-operative complication neuropsychological scores brain-derived neurotrophic factor

		cognitive assessment, such as language, visual and auditory dysfunction, an unstable mental status or mental illness; grade 2–3 atrioventricular heart block or heart rate < 50 min^-1; and known or suspected abuse of an analgesic drug.		COPD, Previous Stroke, Diabetes, Cancer,		followed by a maintenance infusion of 0.1 ml.kg-1.hr-1 Placebo (Saline)		
Chi 2016 a[10]	• China<br>• English<br>• 2016<br>• 116 Patients<br>• Adult patients undergoing off pump coronary artery bypass surgery	left ventricular ejection fraction o40%, left ventricular aneurysm ,acute myocardial infarction in the 2 weeks before OPCAB surgery, atrial fibrillation, need for cardiac valve replacement, associated vascular diseases, severe systemic diseases involving the renal and hepatic systems, respiratory disease (forced vital capacity <50% of predicted values),and preoperative left bundle-branch block	Dexmedetomidine 56.1 (7.2)<br>Control 56.0 (7.7)	CAD	Cardiac Surgery	Dexmedetomidine (high dose 0.6 - 1 mcg kg-1)<br>Control	Cardiac markers and function	Hemodynamic and renal function, post-operative complications

Chi 2016 b[10]	• China<br>• English<br>• 2016<br>• 116 Patients<br>• Adult patients undergoing off pump coronary artery bypass surgery	left ventricular ejection fraction o40%, left ventricular aneurysm ,acute myocardial infarction in the 2 weeks before OPCAB surgery, atrial fibrillation, need for cardiac valve replacement, associated vascular diseases, severe systemic diseases involving the renal and hepatic systems, respiratory disease (forced vital capacity <50% of predicted values),and preoperative left bundle-branch block	Dexmedetomidine 54.0 (7.1)<br>Control 56.0 (7.7)	CAD	Cardiac Surgery	Dexmedetomidine (low dose 0.3 - 0.6 mcg/kg)<br>Control	Cardiac markers and function	Hemodynamic and renal function, post-operative complications
Cho 2016[11]	• Republic of Korea<br>• English<br>• 2016<br>• 200 Patients<br>• Adult patients undergoing valvular heart surgery	left main coronary artery occlusion >50%, hemodynamically significant arrhythmia, left ventricular ejection fraction <30%, intra-aortic balloon pump or ventricular assist device, estimated GFR <15 ml.min-1 per 1.73 m2, use of an a2 agonist to treat hypertension, untreated hypertension, previous exposure to dexmedetomidine, or a history of severe allergy to drugs	Dexmedetomidine 64 (12)<br>Control 62 (13)	HVD	Cardiac Surgery	Dexmedetomidine<br>Control	Incidence of AKI	Hemodynamic and renal function, use of vasopressor, renal failure, post-operative complications, need of transfusion, need of inotropes

Djaiani 2016[12]	• Canada<br>• English<br>• 2015<br>• 183 Patients<br>• Adult patients undergoing older than 60 year undergoing elective complex cardiac surgery and older than 70 year undergoing either isolated coronary revascularization or single-valve repair or replacement surgery with the use of cardiopulmonary bypass (CPB)	history of serious mental illness, delirium, severe dementia,<br>or undergoing emergency procedures	Dexmedetomidine 72.2 (6.4)<br>Control 72.4 (6.2)	Cardiac Disease	Cardiac Surgery	Dexmedetomidin e<br>Control (Propofol)	Delirium onset (CAM)	Blood product transfusion rates, requirement for inotropic<br>and/or vasoconstrictor support, permanent pacemaker<br>insertion, major end-organ dysfunction, extubation times,<br>and ICU and hospital length of stay
Garg 2014 b[13]	• Canada<br>• English<br>• 2014<br>• 6905 Patients<br>• Adult patients undergoing non cardiac surgery	Prior end-stage renal failure	Clonidine 69.1 (10.0)<br>Placebo 69.2 (9.9)	Non-Cardiac Disease	General Surgery	Clonidine<br>Placebo	AKI	Renal function, bleeding, hypotension
Kulka 1996[14]	• Germany<br>• English<br>• 1996<br>• 50 Patients<br>• Adult patients undergoing coronary artery bypass surgery	Prior renal dysfunction and emergency surgery	Clonidine 58 (7)<br>Placebo 57 (2)	CAD	Cardiac Surgery	Clonidine<br>Placebo	Renal function	Hemodynamic function, ADH levels
Li 2017[15]	• China<br>• English<br>• 2017<br>• 285 Patients<br>• Adult patients undergoing cardiac surgery	Younger than 60 y, emergency surgery, severe renal and/or hepatic failure, pre operative bradycardia arrhythmia, previous neurological and psychiatric condition	Dexmedetomidine 66.4 (5.4)<br>Control 67.5 (5.3)	Cardiac Disease	Cardiac Surgery	Dexmedetomidin e<br>Control (Propofol)	Delirium onset	Post-operative complications

Liu 2016[16]	• China<br>• English<br>• 2016<br>• 88 Patients<br>• Adult patients undergoing cardiac surgery	Prior AF, bradycardia, AV block, acute neurological disorder, pre op hemodynamic instability, drug allergy	Dexmedetomidine 53.0 (46.0 - 63.0)<br>Control 56.5 (49.3 - 62.0)	Cardiac Disease	Cardiac Surgery	Dexmedetomidin e<br>Control (Propofol)	AF onset	Post operative complications (AKI, ICU stay, IOT time, ect)
Park 2014[17]	• Republic of Korea<br>• English<br>• 2014<br>• 142 Patients<br>• Adult patients undergoing cardiac surgery	Younger than 60 y, emergency surgery, severe renal or hepatic failure, pre-operative bradycardia arrhythmia, previous neurological and psychiatric condition	Dexmedetomidine 51.09 (16.10)<br>Control 54.35 (13.97)	Cardiac Disease	Cardiac Surgery	Dexmedetomidin e<br>Control (Remifentanil)	Delirium onset	Post-operative complications (AKI, ICU stay, IOT time, ect)
Soliman 2016[18]	• Egypt<br>• English<br>• 2016<br>• 150 Patients<br>• Adult patients undergoing aortic surgery	Recent MI, CHF, heart block, obese, emergency surgery	Dexmedetomidine 58.37 (7.32)<br>Control 57.82 (7.65)	Aortic Disease	Cardiac Surgery	Dexmedetomidin e<br>Control (Remifentanil)	Hemodynamic function	Post-operative complications
Soliman 2017[19]	• Saudi Arabia<br>• English<br>• 2017<br>• 150 Patients<br>• Adult patients undergoing cardiac surgery (CABG or valve procedure) on CPB	emergent CABG, cardiac transplantation, known allergy<br>to study medication, acute renal failure, chronic renal replacement therapy, prior renal transplantation, or aortic aneurysm surgery	Dexmedetomidine 53.54 (10.25)<br>Control 52.62 (9.70)	Cardiac Disease	Cardiac Surgery	Dexmedetomidin e<br>Control (Dopamine)	Renal function	Hemodynamic function, adverse event
Song 2018[20]	• Republic of Korea<br>• English<br>• 2018<br>• 38 Patients<br>• Adult patients undergoing oncological surgery	Prior renal failure, recent MI, AV block, CHF, pregnancy	Dexmedetomidine 51 (10)<br>Control 56 (15)	Oncolo gy disease	General Surgery	Dexmedetomidin e<br>Control	Renal function (creatinine clearance - CrCl)	AKI, renal replacement therapy (RRT), NGAL, SrCr, cardiovascular and other post-operative complications, ITU stay

Wang 2014[21]	• China<br>• English<br>• 2014<br>• 44 Patients<br>• Adult patients undergoing hepatic surgery	operation history of cryosurgery or radio frequency ablation, scheduled resection not requiring hepatic portal occlusion, cardiac ejection fraction < 40%, myocardial infarction within 3 months, or any angina pain within 48 h, pulmonary dysfunction or chronic obstructive pulmonary disease, hypertension, diabetes mellitus, and history of inflammatory bowel disease or diarrhoea within 1 week of surgery.	Dexmedetomidine 27-68<br>Control 30-69	Hepatic Disease	General Surgery	Dexmedetomidin e<br>Control	Intestinal injury, hepatic function	Vasoconstrictor requirement, ITU stay, Cardiovascular function, renal function
Zhai 2017[22]	• China<br>• English<br>• 2017<br>• 72 Patients<br>• Adult patients undergoing cardiac valve surgery	atrioventricular block, heart block, severe left ventricular dysfunction with left ventricular ejection fraction (LVEF) ≤ 40% and those treated with α2-adrenoceptor agonist within two weeks before surgery, kidney dysfunction, hypertension III, and diabetes	Dexmedetomidine 45 (10)<br>Placebo 47 (11)	Valve Disease	Cardiac Surgery	Dexmedetomidin e<br>Placebo	Renal function	ITU stay, post-operative complication
Salah 2013[23]	• Egypt<br>• English<br>• 2013<br>• 80 Patients<br>• Inclusion Criteria: patients with mild to moderate renal impairment and scheduled for elective CABG surgery with CPB. Adult patients scheduled for elective CABG with serum creatinine level between 1.5	Exclusion Criteria: urgent surgery, combined CABG and valve surgery, with ejection fraction less than 45%, hemodynamic instability, using mechanical support devices, with uncontrolled arrhythmias, under treatment with a2 blockers, with uncontrolled type 1 diabetes, morbid obesity, a history of severe drug allergy, liver	Dexmedetomidine: 61.4(6.5)<br>0.9% saline: 58.3 (8)	Cardiac disease , Hypert ension, Diabete s	Cardiac surgery	Dexmedetomidine<br>Placebo (0.9% saline)	creatinine clearance	serum creatinine 24 h before the study and 24, 48, and 72 h after skin closure. Mean arterial pressure. Heart rate. Fluid requirements. Blood loss.

	and 2.0 mg/dl and creatinine clearance less than 60 ml/min despite maximal medical treatment	disease, and diuretic therapy were excluded						

Alpha Natriuretic Peptide

Chen 2007[24]	• USA<br>• English<br>• 2007<br>• 40 Patients<br>• Adult patients undergoing cardiac surgery on CPB	cardiogenic shock or hypotension with systolic BP 90 mm Hg,<br>patients with acute or chronic aortic dissection	Nesiritide 77 (10)<br>Placebo 78 (7)	Cardiac Disease	Cardiac Surgery	Nesiritide<br>Placebo	Renal function	Hemodynamic function, post-operative fluid balance
Ejaz 2009[25]	• USA<br>• English<br>• 2009<br>• 94 Patients<br>• Adult patients undergoing valve surgery on CPB	adverse reaction to nesiritide, organ transplant, preoperative intra-aortic balloon pump, or symptomatic, acute decompensated congestive heart failure	Nesiritide 64.1 (13.31)<br>Placebo 65.91 (10.76)	Valve Disease	Cardiac Surgery	Nesiritide<br>Placebo	AKI and mortality	Renal function
Hisatomi 2012[26]	• Japan<br>• English<br>• 2011<br>• 70 Patients<br>• Adult patients undergoing cardiac surgery on CPB	Emergency procedure, chronic dialysis, angiotensin-converting enzyme (ACE) inhibitor or angiotensin II receptor blocker (ARB) therapy within the previous 2 weeks, received contrast medium within 3 days before surgery or left ventricular ejection fraction <35%	Carperitide 70.9 ± 7.4<br>Control 71.5 ± 7.8	Cardiac Disease	Cardiac Surgery	Carperitide<br>Control	Serum Creatinine	Renal and cardiac function

Izumi 2008[27]	• Japan<br>• English<br>• 2007<br>• 18 Patients<br>• Adult patients undergoing cardiac surgery on CPB	Emergency procedure, chronic dialysis, angiotensin-converting enzyme (ACE) inhibitor therapy within the previous week, received contrast medium within 3 days<br>before surgery or left ventricular ejection fraction <35%	Carperitide 74.7 (7.5)<br>Control 70.5 (12.1)	Cardiac Disease	Cardiac Surgery	Carperitide<br>Control	Serum Creatinine and Creatinine clearance	Renal function, inflammatory, markers, need of inotropes or diuretics, renal replacement therapy (RRT)
Mentzer 2007 [28]	• USA<br>• English<br>• 2007<br>• 272 Patients<br>• Adult patients undergoing coronary artery bypass surgery on CPB	ongoing or chronic dialysis; presence of restrictive or obstructive cardiomyopathy, pericarditis, or pericardial tamponade; documented low cardiac filling pressures; known congenital heart disease; evidence of ongoing infection; and pulmonary disease, including chronic obstructive pulmonary disease or asthma,<br>requiring hospital stay within 60 days	Nesiritide 63.6 (10.5)<br>Placebo 64.1 (11.3)	CAD	Cardiac Surgery	Nesiritide<br>Placebo	Serum Creatinine and Creatinine clearance	Renal function, cardiac function, inflammatory, markers, need of inotropes or diuretics, renal replacement therapy (RRT), post-operative complications, mortality
Mitaka 2008[29]	• Japan<br>• English<br>• 2016<br>• 77 Patients<br>• Adult patients undergoing cardiac surgery on CPB	Emergency procedure, RV infarction, chronic kidney failure, ECMO, iACE, FANS or ARBs therapy within the previous 24 h, received contrast medium within 3 days before surgery, dehydration	ANP 72 (66-78)<br>Placebo 74 (69-78)	Cardiac Disease	Cardiac Surgery	ANP<br>Placebo	Renal function	Renal Replacement Therapy (RRT), ITU stay, post- operative complications, costs

Mitaka 2017[30]	• Japan<br>• English<br>• 2008<br>• 40 Patients<br>• Adult patients undergoing abdominal aorta aneurysm surgery	chronic dialysis or preoperative serum creatinine concentration 3 mg dL-1	ANP 69.4 (7.7)<br>Placebo 73.3 (8.6)	Aortic Aneury sm	Vascular Surgery	ANP<br>Placebo	Renal function	Fluid therapy, inotrope and diuretics dosage
Mori 2014[31]	• Japan<br>• English<br>• 2014<br>• 42 Patients<br>• Adult patients undergoing aortic arch surgery on CPB	Emergency procedure, pre-operative heart failure, prior reaction to ANP	ANP 75.5 (36-87)<br>Placebo 73.0 (53-86)	Aortic Disease	Cardiac Surgery	ANP<br>Placebo	AKI	Dialysis and all cause mortality
Sezai 2000[32]	• Japan<br>• English<br>• 2000<br>• 40 Patients<br>• Adult patients undergoing coronary artery bypass graft surgery on CPB	Emergency procedure	ANP 62.1 (7.9)<br>Placebo 64.8 (5.2)	CAD	Cardiac Surgery	ANP<br>Placebo	Hemodynamic function	Renal function, Angiotensin, Aldosterone, renin GMP and ANP activity
Sezai 2006[33]	• Japan<br>• English<br>• 2006<br>• 150 Patients<br>• Adult patients undergoing coronary artery bypass graft surgery on CPB	Emergency procedure, prior real dysfunction, prior CABG or PCI, LVEF < 50% or ischemia in LAD territory	ANP 63.7 (8.6)<br>Placebo 64.9 (7.3)	CAD	Cardiac Surgery	ANP<br>Placebo	Hemodynamic function	Renal function, Angiotensin, Aldosterone, renin, ANP and BNP levels
Sezai 2007[34]	• Japan<br>• English<br>• 2007<br>• 124 Patients<br>• Adult patients undergoing emergency coronary artery bypass graft surgery on CPB	Prior real dysfunction	ANP 68.7 (9.0)<br>Placebo 65.8 (10.6)	CAD	Cardiac Surgery	ANP<br>Placebo	Hemodynamic function and mortality	Renal function, Angiotensin, Aldosterone, renin, ANP and BNP levels, need of furosemide and KCL

Sezai 2009[35]	• Japan<br>• English<br>• 2009<br>• 504 Patients<br>• Adult patients undergoing coronary artery bypass graft surgery on CPB	Prior real dysfunction	ANP 65.6 (0.6)<br>Placebo 66.3 (0.6)	CAD	Cardiac Surgery	ANP<br>Placebo	Post-operative complications and renal dysfunction	Angiotensin, Aldosterone, renin and ANP levels
Sezai 2011[36]	• Japan<br>• English<br>• 2011<br>• 303 Patients<br>• Adult patients with CKD undergoing coronary artery bypass graft surgery on CPB	Normal renal function	Carperitide 68.8 (6.7)<br>Placebo 68.8(7.8)	CAD	Cardiac Surgery	Carperitide<br>Placebo	Dialysis and renal function	Post-operative outcome after surgery and at 1 year, maximum serum creatinine and ANP/GMP levels
Sezai 2013[37]	• Japan<br>• English<br>• 2006<br>• 367 Patients<br>• High risk adult patients undergoing coronary artery bypass graft surgery on CPB	Euroscore < 6, OPCABG	Carperitide 70.1 (8.4)<br>Placebo 70.8 (8.8)	CAD	Cardiac Surgery	Carperitide<br>Placebo	MACCE	Mortality, BNP and Serum creatinine levels, renal function.

Calcium Channel Blocker

Amano 1995 [38]	• Japan<br>• English<br>• 1994<br>• 19 Patients<br>• Adult patients undergoing coronary artery bypass graft surgery on CPB	Prior real dysfunction, heart failure, use of furosemide and/or ACE inhibitor	Glutathione 58.2 (2.5)<br>Control 56.8 (2.3)	CAD	Cardiac Surgery	Glutathione<br>Control	Hemodynamic and renal function	Hormonal profiles
Bergman 2002 [39]	• Sweden<br>• English<br>• 2002<br>• 24 Patients<br>• Adult patients undergoing cardiac surgery on CPB with	Severely impaired LV, recent radiocontrast investigations or ongoing Ca-Channel blockers	Diltiazem 72 (69-76)<br>Placebo 73 (69-74)	Cardiac Disease	Cardiac Surgery	Diltiazem<br>Placebo	Hemodynamic and renal function	Post-operative complications, need of inotropes, need of diuretics, blood losses and fluid balance

	pre-operative renal failure							
Bertolissi 1996[40]	• Italy<br>• English<br>• 1996<br>• 20 Patients<br>• Adult patients undergoing coronary artery bypass graft surgery on CPB	Emergency, prior renal failure, heart failure and pre-operative diuretics	Nifedipine 63 (3)<br>Placebo 65 (5)	CAD	Cardiac Surgery	Nifedipine<br>Placebo	Hemodynamic and renal function	Fluid balance
Cho 2009[41]	• Republic of Korea<br>• English<br>• 2008<br>• 100 Patients<br>• Adult patients undergoing robot-assisted laparoscopic radical prostatectomy	Prior real dysfunction	Nicardipine 67 (6)<br>Control 68 (4)	BPH	General Surgery	ANP<br>Placebo	Hemodynamic and renal function	Anemia, hospital stay
Colson 1992 a[42]	• France<br>• English<br>• 1992<br>• 24 Patients<br>• Adult patients undergoing abdominal aortic disease	Prior renal dysfunction, cardiac failure, long-term treatment with Ca-channel blockers or CEIs or emergency surgery	Enalapril 58 (4)<br>Nicardipine: 63 (3)<br>Control 63 (1)	AAA	Vascular Surgery	Enalapril<br>Nicardipine<br>Control	Blood pressure and renal function	FeNA, FeK, Hemoglobin
De Lasson 1997[43]	• Denmark<br>• English<br>• 1997<br>• 29 Patients<br>• Inclusion criteria: patients with aorto-iliac occlusive arterial disease undergoing elective aorto-biliac or aorto-bifemoral graft implantation. kidney function was normal after diethyltriamine-<br>• pentaacetic acid (DTPA) renography.	Exclusion Criteria: Exclusion criteria were: age more than 70 years; myocardial infarction within the past year; cardiovascular symptoms or receiving medications; unstable angina or stable angina with congestive heart failure; symptomatic cerebrovascular disease; diabetes mellitus; diastolic arterial pressure ≥100 mm Hg; cardiac valvular stenosis with haemodynamic implications; systemic therapy with bronchodilators or steroids;	Felodipine: 65 (46–69)<br>Placebo: 60 (47–68)<br>Median (range)	NA	Vascular Surgery	felodipine 5 mg orally, given daily for 5 days before operation	(Primary outcomes not defined)	Effective renal plasma flow (ERPF) and glomerular filtration rate (GFR), Angiotensin II levels, Arginine vasopressin, Plasma felodipine. Peroperative heart rate (HR), mean arterial pressure (MAP), and cardiac index (CI). Central venous pressure (CVP), pulmonary artery mean pressure (PAMP) or pulmonary artery wedge pressure (PAW)

		or treatment with a calcium antagonist or an angiotensin converting enzyme inhibitor.						
Donmez 1998[44]	• Turkey<br>• English<br>• 1998<br>• 75 Patients<br>• Inclusion criteria: CABG Patients	Exclusion Criteria: NA	Group (1) verapamil 5mg added to prime solution: n = 25; age, mean = 58.3, SE = 1.9; group (2), nimodipine 1-15mcg.kg-1.min-1 during bypass; n = 25; age, mean = 56.1, SE = 2.6; group (3), control group; normal saline infusion only; n = 25; age, mean = 56.5, SE = 2.0	NA	Cardiac surgery	Verapamil 5 mg in prime in group (1); group (2) received infusion of nimodipine 1-15 mcg/kg/min during bypass; group (3), control group received normal saline only	Creatinine clearance	(Secondary outcomes not defined)
Huh 2014[45]	• Korea<br>• English<br>• 2014<br>• 100 patients<br>• Inclusion Criteria: male patients with preoperative renal insufficiency (eGFR <60 ml/min per 1.73m2) who were scheduled to undergo robot-assisted laparoscopic radical prostatectomy (RALRP).	Exclusion Criteria: Patients with eGFR ≥60 ml.min-1 per 1.73m^2	Control: 68(6)<br>Nicardipine: 68(6)	Diabetes, Hypertension, Ischaemic Heart Disease	Prostate Surgery	Control (0.9% saline)<br>Nicardipine (0.5 mg.kg-1 per Min)	(Primary outcomes not defined)	Serum creatinine and haemoglobin. Postoperative urine output. Haemodynamic parameters (MAP, HR, CVP)
Kim 2014[46]	• Korea<br>• English<br>• 2014<br>• 46 patients<br>• Inclusion Criteria: patients presenting for preanesthetic evaluation for orthognathic surgery. American Society of Anesthesiologists Physical Status had to be 1 or 2	Exclusion Criteria: Patients with known diabetes mellitus, subalimentation, anemia, cerebrovascular accidents, coronary artery diseases, heart failure, peripheral vascular diseases, respiratory insufficiency, anemia, abnormal liver or renal function, hormonal or hypertensive diseases, and previous treatment with	Remifentanil: 21 (19-26)<br>Nicardipine: 24 (20-29)	NA	MaxFax Surgery	Remifentanil<br>Nicardipine	renal function markers: estimated creatinine clearance (CrCl), fractional excretion of sodium (FENa), urinary N-acetyl-1-b-D-glucosaminidase (NAG), and	hemodynamic data: MAP, heart rate (HR), and cardiac index (CI),

	and the orthognathic surgery must have included mandibular osteotomy and Le Fort I osteotomy.	diuretics or antihypertensive drugs					serum cystatin C	
Park 2017[47]	• Korea<br>• English<br>• 2017<br>• 64 patients<br>• Inclusion Criteria: Patients undergoing anterior or posterior spinal interbody fusion.	Exclusion Criteria: NA	Nicardipine: NA<br>Placebo (Normal Saline): NA	NA	Spinal Surgery	Nicardipine<br>Placebo (Normal Saline)	(Primary outcomes not defined)	Creatinine clearance<br>serum cystatin C<br>Fractional excretion of NA
Witczak 2008[48]	• Norway<br>• English<br>• 2008<br>• 20 patients<br>• Inclusion: patients with impaired renal function who were scheduled for elective CBP surgery. Included Males and females with serum creatinine >150mmol L21 and >130mmol L21, respectively,	Exclusion Criteria: unstable angina pectoris, ejection fraction <35% and renal replacement therapy (haemodialysis, peritoneal dialysis or renal transplantation).	Nifedipine: 67.7(9.0)<br>Controls: 65.8(10.6)	Hypertension, Diabetes, Insulin, Peripheral vascular disease, History of acute myocardial infarction.	Cardiac Surgery	Nifedipine<br>Controls (No treatment)	(Primary outcomes not defined)	Renal function (eGFR, Cr-EDTA, creatinine. Albumin (mg mmol-1 creatinine), N-acetyl-b-D-glucosaminidase (NAG) (U mmol21 creatinine) and alkaline phosphatase (ALP) (U mmol-1 creatinine) were measured in all urinary samples. Creatinine (mmol L-1), urea (mmol L-1), sodium (mmol L-1), potassium (mmol L-1), haemoglobin (g dL-1) and albumin were measured in all plasma samples.
Yavuz 2002 2b[49]	• Turkey<br>• English<br>• 2002<br>• 60 patients	Exclusion Criteria: Patients with poor preoperative renal function were excluded from the study	Control: 61.3 (8.3)<br>dopamine only: 58.4(5.3)<br>diltiazem-only: 60.3(7.1)<br>combined dopamine and	N/A	Cardiac Surgery	control (Group 1, n=15), dopamine only (Group 2, n=15)	(Primary outcomes not defined)	Creatinine clearance<br>Osmotic Clearance<br>Microalbuminuria<br>β2-Microglobulin

	• Inclusion Criteria: consecutive patients undergoing elective first-time CABG. Functional capacities of Class I and II according to the New York Heart Association; ejection fraction of 50% and over; normal renal functions (defined as urea less than 50 mg.dl-1, creatinine less than 1.4 mg/dl); no diagnosis of metabolic diseases, such as diabetes mellitus or malignant hypertension (over 140/90 mm Hg); discontinued 10 days prior to surgery from receiving drugs such as angiotensin-converting enzyme inhibitors, diuretics and beta blocking agents.		diltiazem: 58.7(7.1)			diltiazem-only (Group 3, n=15), combined dopamine and diltiazem (Group 4, n=15).		
Zanardo 1993a[50]	• Italy<br>• English<br>• 1993<br>• 35 patients<br>• Inclusion Criteria: patients scheduled for coronary artery surgery	Exclusion Criteria: NA	Control: 57.8 (10.1)<br>Diltiazem - Dl (1 ug.kg-1.min-1): 58.1 (10.7)<br>Diltiazem - D2 (2 ug.kg-1.min-1): 58.3 (5.8)	Hypertension, Diabetes, Prostatic hypertrophy	Cardiac surgery	Control<br>Diltiazem - Dl (1 ug.kg-1.min-1)<br>Diltiazem - D2 (2 ug.kg-1.min-1)	(Primary outcomes not defined)	Renal function (eGFR), creatinine clearance, urine-serum ratio of creatinine, osmolality, fractional excretion of sodium
Zanardo 1993b[50]	• Italy<br>• English<br>• 1993<br>• 35 patients<br>• Inclusion Criteria: patients scheduled for coronary artery surgery	Exclusion Criteria: NA	Control: 57.8 (10.1)<br>Diltiazem - Dl (1 ug.kg-1.min-1): 58.1 (10.7)<br>Diltiazem - D2 (2 ug.kg-1.min-1): 58.3 (5.8)	Hypertension, Diabetes, Prostatic hypertrophy	Cardiac surgery	Control<br>Diltiazem - Dl (1 ug.kg-1.min-1)<br>Diltiazem - D2 (2 ug.kg-1.min-1)	(Primary outcomes not defined)	Renal function (eGFR), creatinine clearance, urine-serum ratio of creatinine, osmolality, fractional excretion of sodium

Dopamine Agonist								
Barr 2008 a[51]	• USA<br>• English<br>• 2008<br>• 79 patients<br>• Inclusion Criteria: ≥18 years old; who were undergoing elective, urgent, or emergency cardiac bypass and/or valve surgery; and who had a preoperative creatinine clearance of ≤40 mL/min. This group comprises 16% of cardiac surgery patients in this hospital	Exclusion Criteria: active hemodialysis, uncontrolled glaucoma (because of possible exacerbation by fenoldopam), pregnancy, nausea and vomiting, or sensitivity to metabisulfite (the preservative for fenoldopam). Patients with preoperative creatinine clearance ≤40 mL/min but with creatinine ≤1.1 (generally thin, elderly females) were excluded because of concern that very small changes in creatinine would have an exaggerated effect on creatinine clearance.	Fenoldopam: 77.2 (1.2)<br>N-Acetylcysteine: 73.8 (2.2)<br>Fenoldopam +N-Acetylcysteine: 73.5 (2.0)<br>Control: 72.4 (2.0)	NA	Cardiac Surgery	Fenoldopam<br>N-Acetylcysteine<br>Fenoldopam +N-Acetylcysteine<br>Control	the difference between the preoperative and postoperative day 3 creatinine clearances.	change in weight between preoperative and postoperative day 3, the percent change in creatinine clearance postoperative day 14, the length of postoperative critical care unit stay, the length of postoperative total hospital stay, the need for renal replacement therapy any time during the postoperative hospitalization, mortality, and hospital costs.
Berendes 1997[52]	• Germany<br>• English<br>• 1997<br>• 44 patients<br>• Inclusion Criteria: Patients with left ventricular ejection fraction ≥0.5 scheduled for CABG	Exclusion Criteria: NA	Placebo: 62 (6.3)<br>Dopexamine (0.5 ug/kg/min): 60 (7.1)<br>Dopexamine (1.0 ug/kg/min): 62 (8.2)<br>Dopexamine (2.0 ug/kg/min): 62 (10.2)	NA	Cardiac Surgery	Placebo<br>Dopexamine (0.5 ug.kg-1.min-1)<br>Dopexamine (1.0 ug.kg-1.min-1)<br>Dopexamine (2.0 ug.kg-1.min-1)	(Primary outcomes not defined)	Haemodynamic parameters, systemic oxygen delivery, hepatic venous oxygen saturation, glucose, lactate, endotoxin, IL-6, SAA, CRP, Creatinine clearance,
Bove 2014[53]	• Italy<br>• English<br>• 2014<br>• 667 patients<br>• Inclusion Criteria: Patients admitted to ICU after cardiac surgery with early acute kidney injury (≥50% increase of serum creatinine level from baseline or oliguria≥6	Exclusion Criteria: previous allergy to fenoldopam, glaucoma, fenoldopam administration within the previous 30 days, use of preoperative renal replacement therapy (RRT) (for these patients we did not request preoperative consent), expected ICU stay less than 24 hours after	Placebo: 70 (9.4)<br>Fenoldopam: 70 (8.2)	Endocarditis, Myocardial infarction, Hypotension, hypertension, atrial	Cardiac Surgery	Placebo (saline)<br>Fenoldopam (starting dose of 0.1 µg.kg-1.min-1 (range, 0.025-0.3 µg.kg-1.min-1).	rate of renal replacement (RRT) administration in the ICU.	mortality (ICU mortality and mortality 30 days after surgery), time receiving mechanical ventilation (hours), length of ICU and hospital stay (days), peak serum creatinine level (mg.dL-1), and the incidence of AKI (According to the

	hours)	randomization, renal replacement therapy (RRT) already started or about to start, do-not-resuscitate orders, and participation in other randomized studies within the previous 30 days		fibrillation, chronic lung disease, Diabetes, Peripheral vascular disease				RIFLE score and definitions)
Carcoana 2003 b[54]	• USA<br>• English<br>• 2003<br>• 100 patients<br>• Inclusion Criteria: Male and nonpregnant female patients aged 21 to 79 year, with a preoperative serum creatinine level of ≤ 1.5 mg.dL-1, who were scheduled for elective, primary coronary artery bypass graft surgery requiring CPB,	Exclusion Criteria: Patients who had cardiac catheterization within 5 days of surgery; preoperative hypotension, defined as a systolic blood pressure <90 mm Hg, at any time; use of an intraaortic balloon pump at any time during the current hospitalization; the administration of dopaminergic or antidopaminergic drugs; a contraindication to the use of DA; and chronic inflammatory disease states, lymphoproliferative disorders, or carcinoma, which are associated with increased serum levels of 2M. Because of the diurnal variation in 2M excretion, patients undergoing surgery in the late afternoon or evening were also excluded.	Placebo: 63.3 (8.8)<br>Mannitol: 64.3 (8.9)<br>Dopamine: 63.8 (9.4)<br>Mannitol and dopamine: 63.4 (7.8)	Hypertension, Myocardial infarction, Pulmonary disease ,Cerebrovascular disease , NIDDM ,IDDM.	Cardiac Surgery	Placebo<br>Mannitol<br>Dopamine<br>Mannitol and dopamine	Beta-2 M excretion rate at one hour post-CPB	Beta-2 M excretion rate at 6 and 24 h post-CPB; urinary flow rate and creatinine clearance at 1, 6, and 24 h post-CPB; and the highest postoperative serum creatinine level. Length of intensive care stay and hospitalization, as well as adverse events.

Cogliati 2007[55]	• Italy<br>• English<br>• 2007<br>• 193 Patients<br>• Inclusion Criteria: Patients undergoing elective cardiac surgery were included if at least 1 of the following risk factors was present: preoperative serum creatinine >1.5 mg/dL, age >70 years, diabetes mellitus on insulin treatment, or prior cardiac surgery.	Exclusion Criteria: Exclusion criteria were defined by age <18 years, preoperative use of inotropes, preoperative dialysis, and known allergy to fenoldopam mesylate.	Fenoldopam: 70.3 (7.6)<br>Placebo: 69.6 (10.4)	Type 1 Diabetes Previous cardiac surgery	Cardiac Surgery	Fenoldopam<br>Placebo	AKI defined as a postoperative serum creatinine level of ≥2 mg.dL-1 with an increase in serum creatinine level of 0.7 mg.dL-1 or greater from preoperative to maximum postoperative values, limited to day 1 and day 2.	Anaesthetic and Surgical Duration, Perioperative Blood Loss, and Fluid Administration. Renal Function, Urine Output, and Fluid Intake
Costa 1990 a[56]	• Italy<br>• English<br>• 1990<br>• 36 Patients<br>• Inclusion Criteria: patients with preoperative renal dysfunction (creatinine clearance 550 mL.min-1) undergoing cardiac surgery.	N/A	C, controls: 61.3 (6.9)<br>D, dopamine: 60.3 (12.3)<br>DN, dopamine and nitroprusside: 54.2 (6.7)	Renal Dysfunction	Cardiac Surgery	controls:<br>dopamine<br>dopamine and nitroprusside	(Primary outcomes not defined)	creatinine clearance; osmolar clearance; free water clearance; fractional excretion of sodium, Lysozyme, cu-Glycosidase/creatinine ratio
Cregg 1999[57]	• Ireland<br>• English<br>• 1999<br>• 30 patients<br>• Inclusion Criteria: ASA I or II, aged 6–18 years, scheduled to undergo corrective spinal surgery for idiopathic scoliosis	Exclusion criteria: history of pulmonary, cardiac, renal, adrenal or hepatic disease, known sensitivity to any of the anaesthetic agents or other drugs to be administered.	Dopamine: 14.6 (3.6)<br>Control: 12.1 (2.8)	NA	Spinal Surgery	Dopamine (3 μg·kg -1.min-1)<br>Control (dextrose)	(Primary outcomes not defined)	Urine output. serum and urinary sodium, urea, creatinine, osmolality. Serum ADH.

De Lasson 1995[58]	• Denmark<br>• English<br>• 1995<br>• 30 Patients<br>• Inclusion Criteria: consecutive patients scheduled for elective vascular surgery with implantation of an aorto-bi-femoral or an aorto-bi-iliac graft due to aortoiliac occlusive arteriosclerosis	Exclusion Criteria: Age more than 70 years; myocardial infarction within the last 3 years, the presence of cardiovascular symptoms or currently taking medication; unstable angina and stable angina pectoris with congestive heart failure; cerebrovascular disease; diabetes mellitus; hypertension (diastolic blood pressure _> 100 mmHg); medication with bronchodilator therapy or steroid.	Dopamine: 63 (50-69)<br>Placebo: 60 (49-68)<br>Median (Range)	NA	Vascular Surgery	dopamine 3 ug.kg-1.min-1 or placebo	(Primary outcomes not defined)	Duration of the operation, aortic cross-clamping time and blood loss. Peri-operative mean arterial blood pressure, pulmonary artery mean blood pressure and central venous blood pressure. Effective renal plasma flow and glomerular filtration rate. Fractional excretion of sodium, fractional excretion of potassium, osmolar clearance and free water clearance. Atrial natriuretic peptide and arginine vasopressin. Aldosterone, angiotensin II and endothelin.
Dehne 2001 a[59]	• Germany<br>• English<br>• 2001<br>• 24 patients<br>• Inclusion Criteria: planned aorto-coronary bypass operation as well as the absence of hepatic dysfunction	Exclusion Criteria: Contrast media given during the previous 2 weeks.<br>.	Dopexamine (DX): 64.0 (7.5)<br>Control: 62.6 (8.0)<br>Control/ Dysfunction: 62.4 (7.5)<br>Dopexamine/ Dysfunction: 65.4(8.1)	NA	Cardiac Surgery	Dopexamine (DX)<br>Control<br>Control/ Dysfunction<br>Dopexamine/ Dysfunction	(Primary outcomes not defined)	Angiotensinase A (ATA) N-Acetyl-b-D-glucosaminidase (NAG), a-1- Microglobulin (a-1-MG), Tamm–Horsfall-Protein (THp), and Immunoglobulin G (IgG), Creatinine (Serum and Urine) and Urine Osmolality
Dehne 2001 b[59]	• Germany<br>• English<br>• 2001<br>• 24 patients<br>• Inclusion Criteria: planned aorto-coronary bypass operation as well as the	Exclusion Criteria: Contrast media given during the previous 2 weeks.<br>.	Dopexamine (DX): 64.0 (7.5)<br>Control: 62.6 (8.0)<br>Control/ Dysfunction: 62.4 (7.5)<br>Dopexamine/ Dysfunction: 65.4 (8.1)	NA	Cardiac Surgery	Dopexamine (DX)<br>Control<br>Control/ Dysfunction<br>Dopexamine/ Dysfunction	(Primary outcomes not defined)	Angiotensinase A (ATA) N-Acetyl-b-D-glucosaminidase (NAG), a-1- Microglobulin (a-1-MG), Tamm–Horsfall-Protein (THp), and Immunoglobulin G

	absence of hepatic dysfunction							(IgG), Creatinine (Serum and Urine) and Urine Osmolality
Dural 2000 a[60]	• Turkey<br>• English<br>• 2000<br>• 36 patients<br>• Inclusion Criteria: elective coronary artery surgery	Exclusion Criteria: (i) serum creatinine levels greater than 1.3 mg.dl-1, (ii) BUN greater than 60 mg.dl-1, (iii) severe hypertension, (iv) presence of any carotid or peripheral artery disease, (v) left ventricular ejection fraction lower than 50%, and (vi) contact with any radiocontrast agent (as in a diagnostic procedure) within 72 hours of surgical procedure.	Control: 53.7 (8.3)<br>Mannitol: 55.4(8.4)<br>Dopamine: 53.2 (10.9)	NA	Cardiac Surgery	Patients received a continuous infusion of dopamine, 3 µg.kg-1.min-1 (Group I), mannitol, 1 Mg.kg-1.h-1 (Group II), no medication (Group III) before the induction of anaesthesia.	(Primary outcomes not defined)	Serum BUN (mg.dl-1) and creatinine (CRE), Urine output (mg.kg-1.h-1), urine log (NAG) activity
Halpenny 2002[61]	• Ireland<br>• English<br>• 2002<br>• 28 patients<br>• Inclusion Criteria: ASA II-III patients undergoing elective aortic surgery requiring infrarenal aortic cross-clamping	Exclusion criteria: concurrent administration of nephrotoxic drugs or diuretics, unstable angina, myocardial infarction within the previous 6 months and diabetes mellitus.	Fenoldopam: 70 (5)<br>Placebo: 69 (6)	NA	Aortic Surgery	Fenoldopam (0.1 µg.kg-1. min-1)<br>Placebo (0.9% Saline)	(Primary outcomes not defined)	Plasma creatinine<br>Creatinine Clearance<br>Haemodynamic Parameters (MAP, HR, CVP)<br>Urine Output, fractional excretion of sodium (FENa), free water clearance (FWC).
Kanchi 2017[62]	• India<br>• English<br>• 2017<br>• 60 Patients<br>• Inclusion Criteria: presence of stable CKD (not on dialysis) and estimated GFR (eGFR) ≤60 ml.min-1 per 1.73 m-2 or creatinine ≥1.4 mg%	Exclusion Criteria: Patients scheduled for on-pump CABG, emergency surgery, redo operations, end-stage renal disease, chronic inflammatory disease/immunosuppression, corticosteroid therapy, age <18 years, enrolled in a conflicting research study, patients on renal	Group A (Control): 57.1(9.5)<br>Group P (Placebo): 61.1(7.6)<br>Group D (Dopamine): 60.7(5.9)	NA	Cardiac Surgery	Group D: received dopamine 2 µg.kg-1.min-1 following anesthesia induction till the end of the surgery and standard care.<br>Group P: Placebo group that received 0.9%	(Primary outcomes not defined)	Serum creatinine, neutrophil gelatinase-associated lipocalin, troponin, and brain natriuretic peptide levels<br>Urine Output<br>Fluid balance

	before the surgery	replacement therapy (RRT) and renal transplanted patients				sodium chloride infusion and standard care. In addition control group: had no renal dysfunction (preoperative serum creatinine of ≤1.4 mg.dl-1 and eGFR ≥60 ml.min-1 per 1.73 m2).		
Lassnigg 2000 b[63]	• Austria<br>• English<br>• 2000<br>• 132 Patients<br>• Inclusion Criteria: adult patients who underwent elective cardiac surgery were	Exclusion Criteria: reoperation because of bleeding, urinary output exceeded 2000 ml within the first 4 of furosemide administration,	Dopamine group<br>Furosemide group:<br>Control group:	Diabetes Hypertension	Cardiac surgery	Dopamine group: 63 (10)<br>Furosemide group: 63 (10)<br>Control group: 65 (10)	change in serum creatinine	occurrence of acute renal injury (ARI), changes in creatinine clearance over time, urine output per hour, volume intake per hour, serum sodium, serum potassium, other parameters of renal function (see below), necessity for hemodialysis or hemofiltration, and hospital mortality rate.
Myles 1993[64]	• Australia<br>• English<br>• 1993<br>• 52 Patients<br>• Inclusion Criteria: elective patients booked for coronary artery bypass surgery, who gave written, informed consent.	Exclusion criteria: pre-existing renal failure (defined as serum creatinine> 300 JlmollL), had infective endocarditis, or where dopamine was contraindicated: phaeochromocytoma, hyperthyroidism, tachyarrhythmias.	Dopamine: 61.0 (10)<br>Control: 62.2 (8.0)	Diabetes, Hypertension,	Cardiac Surgery	1. control group-5% dextrose, 50 ml at 3 ml.l.hr-1;<br>2. dopamine group-dopamine 200 mg in 5% dextrose, 50 ml also at 3 ml.hr-1 (i.e. 200 ug.min-1, or 3 ug.kg-1.min-1 for a patient weighing 66.7 kg). The infusion was commenced at induction and ran	(Primary outcomes not defined)	Fluid Input<br>Fluid Output<br>Perfusion pressure<br>Renal function: urine output, daily serum creatinine, repeated creatinine and free-water clearance

						for 24 hours.		
Parks 1994[65]	• Ireland<br>• English<br>• 1994<br>• 23 Patients<br>• Inclusion Criteria: Adults undergoing elective surgery for obstructive jaundice (serum bilirubin level above 100 pmol l-')	NA	Dopamine Group: NA<br>Control Group: NA	NA	General Surgery	Control group had pre-op IV fluids and frusemide on induction; dopamine group had the above + infusion of dopamine 3 mcg/kg/min for 48 hours	(Primary outcomes not defined)	Urine output, creatinine clearance
Perez 2002[66]	• Spain<br>• English<br>• 2002<br>• 40 Patients<br>• Inclusion Criteria: American Society of Anesthesiology (ASA) 2 and 3 physical status, undergoing colorectal laparoscopic surgery because of a malignant process	Exclusion Criteria: known renal, hepatic, hormonal, and hypertension disease, malnutrition status and previous treatment with diuretic or antihypertensive drugs.	Dopamine group: 64.3 (9.4)<br>Control group: 61.3 (16.7)	Cancer	General Surgery	Dopamine group (continuous intravenous infusion of dopamine at 2 gg/kg/min)<br>Control group (same volume of saline)	(Primary outcomes not defined)	Hemodynamic variables (MAP, CVP, End tidal CO2), Urine output, Creatinine clearance.
Russo 2014[67]	• Italy<br>• English<br>• 2014<br>• 60 Patients<br>• Inclusion Criteria: Patients undergoing gynecological laparoscopic surgery for deep endometriosis.	Exclusion Criteria: diseases (echocardiographic findings suggesting diastolic and/or systolic dysfunction [i.e., ejection fraction <50%]), renal and endocrine disorders and obesity (body mass index (BMI)>30 kg.m-2).	Group A: 33.6(5)<br>Group B: 32(4)<br>Group c: 33(6)	NA	General Surgery	Group A was given saline solution at 5 mL.kg-1.h-1.<br>Group B received saline solution at 5 mL.kg-1.h-1 and dopamine 3 mg.kg-1.min-1.<br>Group C received saline solution at 10 mL/kg/h.	estimated glomerular filtration rate (eGFR) of the intraoperative urinary output and of the ADH serum levels.	cardiac filling parameters by use of tissue doppler imaging (TDI).

Tang 1999[68]	• UK<br>• English<br>• 1999<br>• 40 Patients<br>• Inclusion Criteria: Consecutive patients on the elective coronary artery bypass grafting (CABG) waiting list	Exclusion Criteria: Pre-existing renal disease; Preoperative serum creatinine above 120 mmol.l-1; Preoperative blood urea above 6 mmol.l-1; Hypertension; Diabetes mellitus; Impaired left ventricular function as assessed by contrast ventriculography; Unstable angina; Age more than 70 years	Dopamine: 61.0 (10.3)<br>Control: 56.3 (8.7)	NA	Cardiac Surgery	Dopamine: 2.5–4.0 mg.kg-1 per min starting from induction of general anaesthesia for 48 h,<br>Control: No treatment	(Primary outcomes not defined)	Creatinine clearance<br>Serum Urea<br>Fluid balance<br>Post-operative complications
Wahbah 2000 a[69]	• Egypt<br>• English<br>• 2000<br>• 40 patients<br>• Inclusion Criteria: patients with patients having obstructive jaundice	Exclusion Criteria: NA	Dopamine group n = 10, age, median = 50, range = 37-60; control group n = 10, age, median = 44.5, range = 36-60; dopamine + mannitol group n = 10,<br>age, median = 51, range = 44-58; dopamine + frusemide (furosemide) group n = 10, age,<br>median = 61, range = 55-71	NA	GI Surgery	Group 1: Control<br>Group 2: Dopamine<br>Group 3: Dopamine and Mannitol<br>Group 4: Furosemide	(Primary outcomes not defined)	creatinine clearance<br>serum creatinine
Welch 1995[70]	• UK<br>• English<br>• 1995<br>• 32 patients<br>• Inclusion Criteria: patients undergoing elective infrarenal aortic surgery	Exclusion Criteria: Serum creatinine levels > 150 mmol/L; a history of ventricular or supraventricular arrhythmias; recent or concomitant administration of the following drugs: monoamine oxidase inhibitors, antiemetic agents (except metoclopramide), nonselective beta blockers, mannitol, dopamine, and positive inotropes<br>such as noradrenaline and dobutamine.	Dopexamine: 63.5 (9.8)<br>Placebo: 62.1 (7.7)	NA	Aortic Surgery	Dopexamine<br>Placebo (0.9% Saline)	(Primary outcomes not defined)	Blood product requirement, diuretic administration, post-op complications, renal function.

Woo 2002[71]	• UK<br>• English<br>• 2002<br>• 50 patients<br>• Inclusion Criteria: Consecutive patients undergoing cardiac surgery under two surgeons at Wythenshawe Hospital	Exclusion Criteria: intraoperative circulatory arrest, re-sternotomy for hemostasis, insertion of an intra-aortic balloon pump, major hemodynamic instability that might affect renal perfusion.	Group 1: a 'renal-dose' (3 mg .kg-1.min-1) dopamine: 64.5 (58–82)<br>Group 2: saline infusion acted as placebo: 66.5 (48–84) Mean(range)	Hypertension<br>Diabetes<br>LVEF<40%<br>Unstable angina	Cardiac Surgery	Group 1 received a 'renal-dose' (3 mg .kg-1.min-1) dopamine infusion starting at anaesthetic induction for 48 h saline infusion acted as placebo in Group 2.	Daily Urinary excretion of retinol binding protein (RBP) indexed to Cr	Daily fluid balance, blood urea and serum Cr.
Yavuz 2002 1[72]	• Turkey<br>• English<br>• 2002<br>• 22 patients<br>• Inclusion Criteria: patients with normal preoperative renal function who had CABG.	Exclusion Criteria: NA	Dopamine group, n = 11, age, mean = 55.7, SD = 5.2; control group, n = 11, age, mean = 56.4, SD = 9.5	NA	Cardiac Surgery	Dopamine<br>Control: No treatment	(Primary outcomes not defined)	Renal function: clearances of creatinine<br>Urinary excretion of b2-Microglobulin (b2-M)<br>Urine microalbumin levels
Yavuz 2002 2a[49]	• Turkey<br>• English<br>• 2002<br>• 60 patients<br>• Inclusion Criteria: consecutive patients undergoing elective first-time CABG. Functional capacities of Class I and II according to the New York Heart Association; ejection fraction of 50% and over; normal renal functions (defined as urea less than 50 mg.dl-1, creatinine less than 1.4 mg.dl-1); no diagnosis of metabolic diseases, such as diabetes mellitus or malignant hypertension (over 140/90 mm Hg);	Exclusion Criteria: Patients with poor preoperative renal function were excluded from the study	Control: 61.3(8.3)<br>dopamine only: 58.4(5.3)<br>diltiazem-only: 60.3(7.1)<br>combined dopamine and diltiazem: 58.7(7.1)	N/A	Cardiac Surgery	control (Group 1, n=15), dopamine only (Group 2, n=15)<br>diltiazem-only (Group 3, n=15), combined dopamine and diltiazem (Group 4, n=15).	(Primary outcomes not defined)	Creatinine clearance<br>Osmotic Clearance<br>Microalbuminuria<br>β2-Microglobulin

	discontinued 10 days prior to surgery from receiving drugs such as angiotensin-converting enzyme inhibitors, diuretics and beta blocking agents.							
**Erythropoeitin**								
Ahmed Abdel-latif 2015[(73)] Dardashti 2014[(74)]	• Sweden<br>• English<br>• 2014<br>• 75 patients<br>• Inclusion Criteria: nonemergent CABG, preoperative estimated GFR (eGFR) less than 60 and greater than 15 ml.min-1 (based on p-cystatin C), and written and oral consent.	Exclusion criteria: were uncontrolled hypertension (defined as previously undetected hypertension with no antihypertensive therapy), hypersensitivity to the active drug, pregnancy, fertile women (<50 year old), treatment with erythropoietin up to 4 weeks before the surgery ongoing dialysis, planned off-pump CABG surgery, known malignancy, inclusion in other ongoing clinical trial, or clinical judgment by the investigators that the patient could not participate in the study due to inability to assimilate information such as linguistic barriers.	single high-dose erythropoietin (400 IU.kg-1) IV: 72.4 (8.1)<br>Placebo: 0.9% saline IV: 72.5 (10.5)	Diabetes, Previous PCI, Peripheral vascular disease, Thyroid disease, paroxysmal atrial fibrillation	Cardiac surgery	single high-dose erythropoietin (400 IU.kg-1) IV<br>Placebo: 0.9% saline IV	p-cystatin C levels preoperatively compared to the third postoperative day	ICU LOS, Ventilator time, fluid balance, Bleeding, Diuresis, Post-operative complications, renal replacement therapy (RRT) /Dialysis, Transfusion requirement.

Kim 2016[75]	• Korea<br>• English<br>• 2015<br>• 66 Patients<br>• Inclusion Criteria: patients undergoing thoracic aortic surgery with moderate Hypothermic circulatory arrest (HCA) were enrolled	Exclusion Criteria: cerebrovascular or pulmonary thrombosis, liver dysfunction, chronic kidney disease, hypersensitivity to erythropoietin, malignancy, or prior aortic surgery	erythropoietin 500 IU.kg-1 IV: 64 (37–80)<br>same amount of normal saline placebo IV: 65 (35–92)<br>median (range)	Diabetes, Hypertension, Previous myocardial infarction, Cerebrovascular accident, prior cardiac surgery.	Aortic surgery	erythropoietin 500 IU.kg-1 IV<br>normal saline placebo	incidence of AKI	Hemodynamics, fluid balances including packed red blood cells transfusion, and relevant laboratory values, including troponin-T, were recorded both during and after surgery. In addition, the durations of mechanical ventilation, intensive care unit stay, and hospital stay were recorded.
Song 2009[76]	• Korea<br>• English<br>• 2009<br>• 71 patients<br>• Inclusion Criteria: Patients scheduled for elective CABG	Exclusion Criteria: NA	300 U.kg-1 of EPO: NA<br>Saline Placebo: NA	NA	Cardiac Surgery	300 U.kg-1 of EPO: NA<br>Saline Placebo: NA	(Primary outcomes not defined)	Incidence of AKI, Egfr, Post-operative complications,

Tasanarong 2013[77]	• Thailand<br>• English<br>• 2013<br>• 100 Patients<br>• Inclusion Criteria: aged at least 18 years who were scheduled for elective CABG using the CPB technique at Thammasat Chalerm Prakiat Hospital during the period from January 2010 to March 2011	Exclusion Criteria: Patients with AKI before randomization, CKD stage 5 or unstable renal function (as evidenced by a change in Serum Creatinine of ≥ 0.3 mg.dL-1, or ≥ 50%, within 14 days prior to the study), using the nephrotoxic drugs and/or contrast media administration within two weeks before operation and using recombinant human erythropoietin (rHuEPO) prior to CABG	RHuEPO: 63 (16)<br>Placebo: 60 (16)	Diabetes, Hypertension, Dyslipidaemia	Cardiac surgery	RHuEPO<br>Placebo	incidence of CSA-AKI	comparative changes in Serum Creatinine, eGFR and urine NGAL during the first three postoperative days, postoperative complications, length of stay in the intensive care unit (ICU) and hospital, a requirement for renal replacement therapy (RRT) and all causes hospital mortality between rHuEPO and placebo groups
**Glycaemic Control**								

Wahby 2016[78]	• Egypt<br>• English<br>• 2016<br>• 135 patients<br>• Inclusion Criteria: diabetic patients planned for CABG surgery during the period from January 2013 till January 2015	Exclusion Criteria: emergency CABG, off pump surgery and combined valve and CABG surgery.	Tight Glycaemic Control: 54.99 (6.49)<br>Conventional glycaemic control: 56.40 (7.79)	Hypertension Cerebrovascular accident, Myocardial Infarction	Cardiac Surgery	group A subjected to tight glycemic control during operation to maintain blood glucose level between 110 and 149 mg.dl-1<br><br>group B subjected to conventional moderate glycemic control to achieve blood glucose level between 150 and 180 mg.dl-1 using continuous insulin infusion	(Primary outcomes not defined)	operative mortality (defined as mortality within 30 days of operation or during hospitalization due to cause related to operation), renal dysfunction (elevated serum creatinine above 2 mg.dl-1 postoperative or more than 25% of preoperative level), acute renal failure required postoperative dialysis, postoperative permanent neurological deficit, sternal wound infection, leg infection and need for postoperative inotropic support that was defined as the use of dopamine 5 mg.kg-1.min-1; any dose of epinephrine, norepinephrine, dobutamine, or milrinone.

Zadeh 2016[79]	• Iran<br>• English<br>• 2016<br>• 75 Patients<br>• Inclusion Criteria: Diabetic patients scheduled for non-emergency open cardiac surgery and aged between 18 to 70 years, were included.	Exclusion Criteria: Exclusion criteria:<br>Patients with ketoacidosis or hyperosmolar coma, Redo surgery,<br>patients with a history of cerebrovascular accident (CVA) or transient ischaemic attacks, patients with ejection fraction<30% and patients with a history of liver or kidney disease.	Modified tight control: 58.18(10.77)<br>Conventional control: 59.18(8.91)	Hypertension, myocardial infarction, arrythmia, atrial fibrillation, smoking	Cardiac surgery	modified tight control (Blood Sugar maintained between 100-120 mg.dl-1)<br><br>conventional method (Blood Sugar < 200 mg.dl-1).	mortality, sternal wound infection, duration of mechanical ventilation, cardiac arrhythmias (Atrial fibrillation, Cardiac arrest, Heart block which require pacemaker), cerebrovascular attack and acute renal failure (two-fold increase in baseline creatinine)	length of ICU stay

Akina 2018[80]	• Japan<br>• English<br>• 2018<br>• 30 Patients<br>• Inclusion Criteria: patients diagnosed with oral malignant tumors who were aged ≥ 60 years and scheduled for radical operation with tissue reconstruction (scheduled time required ≥ 8 h) from February 2013 to May 2016	Exclusion: short operation time less than 8 h (3 patients in the GI group), hypoglycemia during GI infusion (1 patient in the GI group), and diabetes mellitus (3 patients in the control group and 2 patients in the GI group).	intraoperative glycaemic control: 77.0 (8.2)<br>control group: 72.7 (8.4)	NA	maxillofacial surgery	intraoperative glycaemic control by glucose-insulin (GI) infusion<br><br>control group: combination of acetate Ringer's solution which contains 1% (W/V) glucose and lactate Ringer's solution	(Primary outcomes not defined)	Pre- and postoperative C-reactive protein (CRP), body temperature measured in the armpit, total protein (TP) and serum albumin concentration, days until discharge, and incidence of postoperative hypoalbuminemia and complications
**Goal Directed Therapy**								

Bartha 2013[81]	• Sweden<br>• English<br>• 2013<br>• 150 patients<br>• Inclusion Criteria: patients aged ≥70 year and weight ≥40 kg who were undergoing PFF surgery during regular operating hours	Exclusion Criteria: (i) patients who could be harmed due to the treatment (ongoing myocardial infarction, chronic dialysis), (ii) concomitant medication with lithium, (iii) known allergy to lithium or medical device components, (iv) weight ≤40 kg, (v) life expectancy ,6 months, (vi) pathological fractures and conditions, (vii) inability to give informed consent, (viii) anticipated difficulties obtaining data during the first postoperative year (as judged by a research team member), and (ix) operations scheduled during hours when research team was unavailable.	Standard of Care: 85 (70–101)<br>GDHT: 86 (71–101) median and range.	Cardiovascular Disease, Respiratory disease, Hypertension, Diabetes, Kidney disease, Rheumatoid arthritis, Dementia	Orthopaedic Surgery	Routine fluid treatment<br>Goal-directed haemodynamic treatment	difference between the absolute risks of postoperative complications in survivors at hospital discharge	volume of administered fluids, proportion of patients with intraoperative hypotension, intraoperative haemodynamic responses, mortality (within 30 days of surgery), aggregated healthcare costs, use of social services, and postoperative quality of life at 12 months.
Bisgaard 2013[82]	• Denmark<br>• English<br>• 2012<br>• 70 Patients<br>• Inclusion Criteria: Adult patients scheduled for OEAAS who can provide written informed consent	Exclusion Criteria: end-stage renal failure, lithium therapy, body weight < 40 kg or intervention period not completed.	Individual goal directed therapy: 68 (6.6)<br>Conventional Therapy: 68 (9.4)	N/A	Vascular surgery	Individual goal directed therapy<br>Conventional Therapy	The primary end point was the number of post-operative complications	Secondary end points were the intensive care unit (ICU) and hospital length of stay.

Calvo-Vecino 2018[83]	• Spain & London<br>• English<br>• 2018<br>• 450 Patients<br>• Inclusion Criteria: aged 18 year or older and scheduled for major abdominal, urological, gynaecological, or orthopaedic surgery under general anaesthesia using laparoscopic or open approaches. Surgery was considered major if it fulfilled at least one of the following criteria: expected duration >2 h, estimated blood loss >15% of blood volume, or transfusion requirements of at least two packs of red blood cells.	Exclusion criteria: emergency surgery, ASA physical status31 >3, contraindications for ODM, or aortic pathology that could lead to misinterpretation of haemodynamic variables (e.g. intraaortic balloon pump, thoracic aorta aneurysm).	Control Group: 64.2 (67)<br>GDHT group: 66.3 (71)	Obesity, Smoking, Hypertension Coronary artery disease, Congestive heart failure, diabetes mellitus, chronic obstructive pulmonary disease, or chronic alcohol consumption	Mixed Surgery	Control: continuous infusion of balanced crystalloid fluids (Ringer's lactate) IV fluids, vasopressors, and inotropes according to a haemodynamic algorithm	percentage of patients who developed pre-defined moderate or severe postoperative complications within 180 days of surgery, including complications that occurred before or after discharge from hospital and required outpatient or inpatient care.	length of hospital stay (LOS) defined as the number of days spent in the hospital from the day of surgery to hospital discharge or death), length of stay in the intensive care unit, re-interventions, time to onset of oral tolerance and time to ambulation, and all-cause mortality at 180 days after surgery.

Cesur 2018[84]	• Turkey<br>• English<br>• 2018<br>• 70 patients<br>• Inclusion Criteria: ASA (American Society of Anesthesiology) I–II patients over the age of 18 years, who would undergo elective open colorectal tumor surgery.	Exclusion Criteria: serious cardiac arrhythmia and peripheral artery disease, an ejection fraction below 30%, a pulmonary pathology preventing inhalation with a volume more than 6 ml.kg-1 via mechanical ventilation and the presence of liver and renal dysfunction.	conventional fluid management: 62.31 (10.52)<br><br>PVI-based goal directed fluid management: 58.68 (14.41)	N/A	GI Surgery	conventional fluid management: 62.31 (10.52) PVI-based goal directed fluid management: 58.68 (14.41)	Amount of crystalloids administered, blood lactate, and serum creatinine levels during the intraoperative period.	Length of Hospital Stay
Challand 2012[85]	• UK<br>• English<br>• 2011<br>• 179 patients<br>• Inclusion Criteria: NA	Exclusion Criteria: All patients undergoing major colorectal surgery underwent CPET on a stationary bicycle (Zan, nSpire, CO, USA) as part of their routine preoperative assessment. Anaerobic threshold (AT), determined by V slope and ventilatory equivalents, was used as the marker of aerobic fitness. Individuals whose oxygen consumption at AT was undetectable or measured ,8.0 ml O2 kg min (considered too unfit to randomize) were excluded,	Control – Standard fluid therapy 65.9 (14.1)<br>GDT - 66.0 (15.6)	N/A	GI Surgery	Standard fluid regimen<br>GDFT	Readiness for discharge - based on predefined criteria, that is, tolerance of oral diet, mobilization and self-support at an appropriate level, adequate pain control with simple oral analgesics, return of adequate lower gastrointestinal function, and adequate stoma care, where applicable.	actual length of stay (LOS), critical care admission, 30 and 90 day mortality, post-operative complications and 30 day hospital readmission rates.

Demirel 2017[86]	• Turkey<br>• English<br>• 2017<br>• 60 patients<br>• Inclusion Criteria: adult Patients undergoing elective laparoscopic RYGB surge	Exclusion Criteria: Patients below 18 years of age, having cardiac<br>arrhythmia, cardiac ejection fraction of ≤ 30%, pulmonary disease interfering with mechanical ventilation by ≥ 8 ml.kg-1<br>tidal volume, or chronic renal failure	Standard fluid regimen: 40.07 (11.92)<br>GDFT protocol via Pleth Variability Index (PVI): 36.33 (10.80)	Obesity	GI Surgery	Standard fluid regimen<br>GDFT protocol via Pleth Variability Index (PVI)	(Primary outcomes not defined)	Lactate, creatinine, blood pressure
Elgendy 2017[87]	• Egypt<br>• English<br>• 2017<br>• 43 patients<br>• Inclusion Criteria: included high-risk patients scheduled for major abdominal surgeries with anticipated operative time of >120 min or blood loss of >20% of blood volume.	Exclusion Criteria N/A	Conventional Fluid Therapy: 57 (8.5)<br>Goal directed fluid therapy group: 58 (6.6)	Diabetes, Hypertension, Ischaemic heart disease.	GI Surgery	Goal directed fluid therapy group:<br>Conventional Fluid Therapy:	changes of SVV and CI using the Vigileo-FloTrac system on the frequency of PO complications encountered during ICU stay, ICU and hospital length of stay (LOS) in high risk patients	ICU mortality, the frequency of ICU re-admission and total hospital morbidity and mortality.

Funk 2015[88]	• Canada<br>• English<br>• 2015<br>• 40 patients<br>• Inclusion Criteria: over the age of 18 years presenting for elective open repair of their AAA.	Exclusion Criteria: age over 80 years, weight greater than 120 kg, known or suspected aortic insufficiency, renal dysfunction (serum creatinine >150 µmol.l-1), active congestive heart failure, or atrial fibrillation.	GDT: 70 (9)<br>Control: 67 (8)	diabetes, hypertension, hyperlipidemia, and ischemic heart disease	Vascular Surgery	GDT targeting stroke volume variation with an arterial pulse contour cardiac output monitor. Control, where fluid therapy was administered at the discretion of the attending anaesthesiologist	hospital LOS	composite outcome of perioperative complications. Traditional hemodynamic parameters of heart rate, MAP, and CVP. Surgical duration and aortic cross clamp times.

Goepfert 2013[89]	• Germany<br>• English<br>• 2013<br>• 100 patients<br>• Inclusion Criteria: patients, scheduled either for coronary artery bypass grafting with the use of cardiopulmonary bypass (CPB) or aortic valve replacement or combined surgery (coronary artery bypass grafting and aortic valve replacement),	Exclusion: age less than 18 years, pregnancy, any contraindications for catheterization of the femoral artery, kidney injury requiring dialysis therapy, valve insufficiency of more than II and pre-existing atrial fibrillation.	GDFT Group: 67.3 (7.6)<br>Control Group: 65.5 (9.9)	NA	Cardiac Surgery	Control Group<br>GDFT Group:	duration of postoperative ICU therapy and time to fulfilment of predefined ICU discharge criteria	need for vasopressor and catecholamine support and predefined clusters of postoperative complications.
Gomez-Izquierdo 2017[90]	• Canada<br>• English<br>• 2017<br>• 135 Patients<br>• Inclusion criteria: Consecutive patients scheduled for elective laparoscopic colorectal resection able to give written consent	Excluded patients: younger than<br>18 years old, required emergency surgery, had undergone previous esophageal or gastric surgery, had esophageal varices or cancer, coarctation of the aorta, chronic atrial fibrillation,<br>severe aortic stenosis, preoperative bowel obstruction, coagulopathies, contraindications to epidural analgesia, if they<br>were chronically treated with opioids, and if they did not read or communicate in French or English	GDFT group: 63 (15)<br>Control group: 61 (15)	Cancer Inflam matory bowel disease Divertic ulitis	Colorecta l surgery	goal-directed fluid therapy based on near maximal stroke volume optimization.<br>fluid therapy based on traditional principles (control group).	primary postoperative ileus and GI function	Quality of Recovery score, 30-day complications, readiness to be discharged, length of hospital stay, and readmission rates.<br>Postoperative complications

Han 2016[91]	• China<br>• English<br>• 2016<br>• 40 patients<br>• Inclusion Criteria: (1) age 65 to 80 years; (2) unilateral hip replacement; (3) American Society of Anesthesiologists (ASA) physical status I-II; and (4) no contraindications for spinal anesthesia.	Exclusion Criteria: (1) cardiovascular diseases such as; hypotension, heart valve disease, arrhythmia, heart failure or a history of heart infarction; (2) severe complications including renal failure or severe hypovolemia; and (3) contraindications for central neural blockade such as; elevated intracranial pressure or coagulopathy.	normal fluid therapy group (group N): 73.9(61) LiDCO-Rapid guiding fluid therapy group (group L): 74.0(5.9)	Hypotension Arrythmias Acute coronary syndrome Pneumonia Pulmonary embolism Urinary infection Acute renal failure	Orthopaedic surgery	Normal Fluid Therapy group LiDCO-Rapid guiding fluid therapy group	Mean arterial pressure and heart rate	Inotropic support, fluid volume, blood consumption, haemodynamic parameters, MAP, Post-operative complications.

Harten 2008[92]	• Glasgow<br>• English<br>• 2008<br>• 30 patients<br>• Inclusion Criteria: patients over the age of 50 undergoing emergency abdominal surgery.	Exclusion Criteria: Patients who presented in a state of emergency following trauma, were to undergo vascular surgery, in whom surgery was expected to last less than 90 min or who were on lithium drug therapy	GDT Group: Lidco cardiovascular monitor: 66 (56–75)<br><br>Control group: standard care: 64 (51–76)	NA	GI Surgery	GDT Group: Lidco cardiovascular monitor<br>Control group: standard care:	Renal Function – serum urea, creatinine and cystatin C pre-operatively and throughout the postoperative period.	The Sequential Organ Failure Assessment (SOFA) score was calculated to assess organ function prior to and following surgery on day 1, day 3 and day 5.<br>The Systemic Inflammatory Response Syndrome (SIRS) score were assessed prior to and following surgery on day 1, day 3 and day 5.16 The Revised Cardiac Risk Index and POSSUM scores were documented immediately before emergency surgery.<br>Mortality at 30 days and length of hospital stay was confirmed from Greater Glasgow Health Board

Hasanin 2019[93]	• Egypt<br>• English<br>• 2019<br>• 120 Patients<br>• Inclusion Criteria: adult patients, aged between 18 years and 65 years, scheduled for major abdominal surgery with an anticipated duration of 180 minutes or more.	Exclusion criteria: Patients with cardiac arrhythmias, impaired cardiac contractility, patients with body mass index above 40 kg/m2, and patients with neck or chest lesions that impair the application of cardiometry electrodes	GDT Group: 49 (13)<br>Control Group: standard care: 50 (12)<br>Mean (SD)	Diabetes<br>Hypertension<br>Smoking	GI Surgery	GDT Group.<br>Control Group: standard care.	Lung ultrasound score (LUS)	Intraoperative fluids: total intraoperative fluid requirements, number of fluid boluses, number of patients requiring vasopressors, and urine output<br>Hemodynamic data: MAP, heart rate, and central venous pressure (evaluated every 5 minutes starting from the baseline preoperative reading till patient discharge from the post-anesthesia care unit)<br>Demographic data: age and gender.<br>Surgical data: surgical duration, blood loss, and type of operation.<br>Postoperative data: postoperative pH, HCO3, PCO2, P/F ratio (defined as PaO2 / Fraction of inspired oxygen), blood haemoglobin, length of ICU stay, and incidence of surgical complications.

Kapoor 2016[94]	• India<br>• English<br>• 2016<br>• 120 Patients<br>• Inclusion Criteria: either sex, with European system for cardiac operative risk evaluation ≥3 undergoing coronary artery bypass grafting on cardiopulmonary bypass.	Exclusion criteria: Patients with<br>cardiac dysrhythmias, contraindication to the central<br>venous cannulation, Patients requiring the initiation of intra-aortic balloon pump (IABP)<br>therapy	GDT Group: 61.17(5.09)<br>Control Group: 61.30(5.60)	N/A	Cardiac Surgery	Control group: Standardised care. GDT Group: cardiac index (CI) monitoring using FloTrac and and continuous central venous oxygen saturation (ScVO2) using PreSep™		heart rate (HR), mean arterial pressure (MAP), CVP, SpO2, ABG, CI, SVRI, DO2I, SVI, SVV, ScVO2, BNP, NGAL, lactate, Duration of inotropic support, duration of ventilation, hospital stay, ICU stay

Kaufmann 2017[95]	• Germany<br>• English<br>• 2017<br>• 100 patients<br>• Inclusion Criteria: indication for elective lung parenchyma resection (mostly for cancer or metastases) via thoracotomy or video-assisted thoracoscopy	Exclusion Criteria: Exclusion criteria were emergency surgery, age<18 years, New York Heart Association Functional Classification 4, morbid obesity (BMI>50 kgm2), cardiac pacemaker, automated implantable cardioverter defibrillator, oesophageal pathologies, pregnancy, intraoperative blood loss>1.5 L, intraoperative use of diuretics, recent history of pulmonary embolism, cardiac valve pathologies and intraoperative blood transfusion.	Goal directed therapy group: 65 (56–70) Standard care group: 65 (55–74)	Ischaemic heart disease, Hypertension, Diabetes mellitus, renal function,	Thoracic Surgery	standard haemodynamic management (control group) or goal-directed therapy (GDT group) guided by an oesophageal Doppler monitoring-based algorithm.	postoperative pulmonary complications (PPCs)	haemodynamic variables, renal, cardiac, and neurological complications, and length of hospital stay.

Kumar 2015[96]	• India<br>• English<br>• 2015<br>• 40 patients<br>• Inclusion criteria: Patients undergoing major abdominal surgeries (abdominal aortic aneurysm, bowel obstructions, mesenteric ischemia, bowel malignancy involving resection and anastomosis)	Exclusion Criteria: Those with renal dysfunction, liver dysfunction, poor cardiac reserve with ejection fraction ≤ 35%, those undergoing hepatic resections or any condition that contraindicated fluid resuscitation	Goal directed therapy: N/A<br>Control group: N/A	N/A	GI Surgery	Group A: CVP was targeted with boluses of crystalloids or colloids, MAP with fluids or Inotropes/ vasopressor (noradrenaline, dopamine) and ScvO2 measured hourly was maintained with fluids,<br>Group B: (enhanced GDT: above +advanced CO monitoring by arterial pressure analysis). CI was maintained within the desired range throughout surgery by administering boluses of crystalloid, 4% hydroxyethyl starch and blood transfusions when indicated	levels of lactate and base deficit intra-operatively and for 12 h postoperatively	(HR) blood pressure (BP), UO, ScvO2, CI and O2ER, ICU LOS, Hospital LOS, Fluid requirements, Post-operative ventilation requirement, post-operative complications.

Luo 2017[97]	• China<br>• English<br>• 2017<br>• 150 patients<br>• Inclusion Criteria: age >18, ASA score III or IV, and expected duration of surgery >2 h.	Exclusion Criteria: body weight <40 kg or >100 kg, patients with cardiac arrhythmia (well-known limitation to the use of the stroke volume variation as an indicator of fluid responsiveness)<br>[	Care group (control group): 62 (13)<br>GDFR group: 61 (13)	Coronary artery disease, Hypertension, Peripheral artery disease, COPD, Cerebrovascular disease, Diabetes, chronic kidney disease, malignancy	Neuro Surgery	Care group (control group)<br>GDFR group	ICU length of stay	lactates at the end of surgery, postoperative complications at day 30, postoperative morbidity (the proportion of patients who developed one or more complications) at day 30, mortality at day 30, hospital length of stay, and costs.

Marathias 2006[98]	• Greece<br>• English<br>• 2006<br>• 45 patients<br>• Inclusion criteria: men and women admitted for elective open heart surgery at Onassis Cardiac Surgery Center, who suffered from moderate-to-severe CKD (glomerular filtration rate calculated by the Cockroft-Gault equation less than 45 mL.min-1).	Exclusion Criteria: Not clearly stated	Control: fluid and food restriction: 64.2 (2.8)<br>Intervention: intravenous hydration with half-isotonic saline at a rate of 1 mL.kg-1.h-1 for 12 h before the operation: 64 (1.7)	Chronic kidney disease, stroke, congestive heart failure (assessed by the preoperative ejection fraction), diabetes mellitus, and obstructive lung disease	Cardiac Surgery	Control: fluid and food restriction.<br>Intervention: intravenous hydration with half-isotonic saline at a rate of 1 mL/ kg/h for 12 h before the operation.		cardiopulmonary bypass (CPB) duration and aorta clamp time, duration and type of operation,<br>number of aortocoronary grafts,<br>intra-aortic<br>balloon pump use, inotrope use, need for reoperation<br>and transfusion requirements, and durations of intubation, intensive care unit (ICU), and hospital stay.

Mikor 2015[99]	• Hungary<br>• English<br>• 2015<br>• 84 Patients<br>• Inclusion Criteria: all patients undergoing the following elective major abdominal surgeries, including oesophagectomy, total gastrectomy, radical cystectomy, aorto-bifemoral bypass or elective repair of abdominal aortic aneurysm,	Exclusion Criteria: Exclusion criteria were pre-existing chronic organ insufficiency as determined by the Acute Physiology and Chronic Health<br>Evaluation (APACHE) II scoring system, New York Heart Association Class IV, chronic hypoxia or hypercapnia,<br>chronic renal failure requiring renal replacement<br>therapy, biopsy proven cirrhosis or portal hypertension<br>and immunodeficiency [19]. Furthermore, in cases of preoperative anaemia (haemoglobin < 100 g.L-1), coagulation abnormality, and patients with chronic use of corticosteroids and non-steroid anti-inflammatory drugs<br>were also excluded. Patients requiring an operation due to malignant disease where the tumour then proved to be inoperable were also excluded.	$ScvO_2$ groups: 62 (8)<br>Control group: 62 (8)	NA	GI Surgery	$ScvO_2$ groups<br>Control group	incidence of postoperative complications	Baseline criteria, renal outcomes, difference in intraoperative<br>fluid and vasopressor requirements.

Moppett 2015[100]	• UK<br>• English<br>• 2015<br>• 130 Patients<br>• Inclusion Criteria: Patients admitted through the emergency department with primary fragility hip fracture, aged over 60 who were listed for surgical repair under spinal anaesthesia were enrolled. After ethical approval, patients unable to give their own consent were included [Abbreviated Mental State Score (AMTS) ≤6 out of 10].	Exclusion Criteria: planned general anaesthetic for surgery repair; severe valvular heart disease (as this could affect the accuracy of the LiDCO device); taking therapeutic lithium (as this can affect the calibration of the LiDCO device); multiple injuries; and revision hip surgery or requirement for total hip arthroplasty.	Intervention: targeted i.v. colloid boluses using invasive pulse contour analysis continuous cardiac output monitoring to optimize SV: 85 (78–90) (68–95) Control: Usual care - anaesthetist-directed fluid therapy: 85 (80–88) (63–95) median (IQR) (range)	N/A	Orthopaedic Surgery	Intervention: targeted i.v. colloid boluses using invasive pulse contour analysis continuous cardiac output monitoring to optimize SV. Control: Usual care - anaesthetist-directed fluid therapy	Time until medically fit for discharge	Postoperative complications, mobility, and mortality.

Osawa 2016[101]	• Brazil, Italy, UK.<br>• English<br>• 2016<br>• 126 patients<br>• Inclusion criteria: Patients undergoing coronary artery bypass grafting (CABG) or valvular surgery. European System for Cardiac Operative Risk Evaluation score equal to or greater than 6, left ventricular ejection fraction lower than 50%, recent acute myocardial infarction or high-risk unstable angina (< 14 d), or combined cardiac surgical procedure.	Exclusion Criteria: emergency surgery, heart transplantation, ascending and/or descending thoracic aortic procedure, enrolment in another study, pregnancy, neoplasm, endocarditis, pulmonary hypertension (systolic pulmonary artery pressure > 40 mm Hg), preoperative use of catecholamines, congenital heart disease, stage 5 chronic kidney disease, and refusal to consent. Patients with aortic regurgitation, preexisting atrial fibrillation, need for intra-aortic balloon pump during the intervention period, and need for a norepinephrine dose greater than 1 µg.kg-1.min-1.	cardiac output–guided hemodynamic therapy algorithm: 66 (9) usual care: 69 (9)	Heart failure, Previous myocardial infarction, Hypertension, Peripheral artery disease, COPD, Diabetes, Previous stroke, dyslipidaemia.	Cardiac Surgery	cardiac output–guided hemodynamic therapy algorithm Usual care	composite endpoint of 30-day mortality and major postoperative complications.	30-day incidence of delirium, seizure, acute kidney injury, venous thromboembolism, tachyarrhythmia, and bradyarrhythmia, and ICU and hospital length of stay. Measurements of Sequential Organ Failure Assessment (SOFA) score and hemodynamic variables, including HR, MAP, Svco2, lactate, and haemoglobin levels.

Parke 2015[102]	• New Zealnad<br>• English<br>• 2015<br>• 144 Patients<br>• Inclusion Criteria: age>16, if undergoing cardiac surgery, involving full median sternotomy and use of cardiopulmonary bypass	Exclusion Criteria: emergency procedure, had a preoperative intra-aortic balloon pump (IABP), pre-existing atrial fibrillation (AF), or end-stage renal failure.	Usual care: 61.2 (18–86)<br>Protocolised Strategy: 65.4 (38–86)	N/A	Cardiac Surgery	Usual care.<br>Protocolised Strategy.	difference in fluid administered to subjects in each group, from the time of admission to ICU, to 24 h, or de-sedation, whichever occurred first	Patient characteristics, co-morbid conditions and operative details were recorded, and length of stay and use of inotrope or vasopressor medications in the ICU. Continuous data from bedside haemodynamic monitors were downloaded into Excel (Microsoft, Redman WA USA) spreadsheets for analysis All patients were contacted 90 days after randomisation, to determine mortality and incidence of requirement for renal replacement therapy.
Peng 2014[103]	• China<br>• English<br>• 2014<br>• 80 Patients<br>• Inclusion Criteria: Patients scheduled for elective major orthopedic surgery, including total hip arthroplasty, spinal fusion surgery, femoral fracture surgery, and sacral tumor surgery under general anesthesia, with an anticipated blood loss >800 ml,	Exclusion Criteria: under 18 years old or had a BMI >40 or <15, coagulopathy, significant arrhythmia or cardiopulmonary dysfunction, or significant renal or liver diseases.	GDT Group: 55 (13)<br>Control/conventional standard of care: 53 (10)	Hypertension<br>Diabetes<br>Anaemia<br>COPD<br>Cerebral infarction	Orthopaedic Surgery	GDT Group<br>Control/conventional standard of care	Haemodynamic data<br>Laboratory parameters<br>Fluid Management (Blood loss, Volume infused, Urinary output)	Post-op Complications<br>Fluid management (Blood transfused, Drainage volume)<br>Length of stay<br>Mortality

Pestana 2014[104]	• Spain<br>• English<br>• 2014<br>• 170 Patients<br>• Inclusion criteria: Adult patients scheduled for open colorectal surgery, gastrectomy, or small bowel resection	Exclusion Criteria: not requiring ICU admission or in case of laparoscopic or emergency surgery, abdominal procedures not related to the above mentioned, intra-abdominal infection, life expectancy <60 days, and disseminated malignancy.	GDT Group: 73.5 (63.5 to 80)<br>Control/conventional standard of care: 74 (64 to 79)<br>median (interquartile range)	Cancer, COPD, Hypertension Ischaemic heart disease, Peripheral vascular disease, Congestive heart failure, Diabetes, Cirrhosis,	GI Surgery	GDT Group<br>Control/conventional standard of care	Hospital LOS<br>Morbidity<br>Renal failure<br>Pulmonary oedema<br>Circulatory failure<br>Infection	Secondary variables included the time to first flatus (considering time zero the end of surgery), the presence of wound infection or anastomotic leaks, and any cause mortality.

Phan 2014[105]	• Australia<br>• English<br>• 2014<br>• 100 patients<br>• Inclusion Criteria: suitability for an Enhanced Recovery After Surgery care pathway and patients with an American Society of Anesthesiologists Physical Status score of 1 to 3.	Exclusion Criteria: American Society of Anesthesiologists Physical Status 4, pregnancy, inability to give informed consent, emergency surgery, significant renal dysfunction (estimated glomerular filtration rate < 50 ml/minute), hepatic dysfunction, severe heart failure (New York Heart Association classification 3 or 4), age < 18 years and oesophageal pathology (such as varices),	RES Therapy: 65 (19.9)<br>GDT Therapy: 63.1 (23.8)	Smoker, Ischaemic heart disease, diabetes, congestive cardiac failure Renal impairment, COPD, Asthma, inflammatory bowel disease	GI Surgery	Restrictive fluid therapy<br>Doppler guided fluid therapy.	Hospital length of stay	any complication, number of patients suffering from major complications (Clavien-Dindo grade 3 or higher), intravenous fluid volumes administered to patients and change in patients' haemodynamic parameters.

Puckett 2017[106]	• New Zealand<br>• English<br>• 2017<br>• 41 Patients<br>• Inclusion Criteria: patients aged 18 to 85 years scheduled for elective colon resection.	Exclusion Criteria: estimated glomerular filtration rate (eGFR) <60 mL.min-1 per 1.73m-2, previous stage 2 or 3 AKI,14 proteinuria (>100 mg.dL-1 on random morning urine dipstick testing), a single functioning kidney, kidney transplantation, administration of potentially nephrotoxic substances (angiotensin-converting enzyme inhibitors, angiotensin receptor blockers, intravenous radiocontrast dyes, aminoglycosides, glycopeptides, and nonsteroidal anti-inflammatory drugs other than aspirin) 48 hours or less before surgery, pregnancy or breast feeding, ASA class IV, body mass index >35 kg.m-2, and pre-existing hepatic failure (Child-Pugh score >6). Subject with intraoperative ureteric injury or intraoperative hemorrhage >50% of blood volume	Low urine output target: 67(8.6)<br>Standard urine output target: 66(12.6)	NA	Gi Surgery	Low urine output target<br>Standard urine output target	concentration of neutrophil gelatinase-associated lipocalin in urine (uNGAL)	Serum cystatin C, serum creatinine, ERPF, and mGFR; intravenous and oral fluid administration; urine output; fluid balance; changes in body weight; and plasma concentrations of ADH, renin, aldosterone, and angiotensin II.
Pull Ter Gunne 1990 [107]	• Netherlands<br>• English<br>• 1990<br>• 26 Patients<br>• Inclusion Criteria: infrarenal aortic aneurysm	N/A	NA	Aortic Aneurysm	Vascular Surgery	Optimal Hydration<br>Conventional care	Renal Function	haemodynamic parameters

Schmid 2016[108]	• Germany<br>• English<br>• 2016<br>• 193 patients<br>• Inclusion Criteria: older than 18 years, American Society of Anaesthesiologists (ASA) physical status classification 1–3 undergoing major non-cardiac surgery planned to last at least 3 hours with an expected subsequent intensive care treatment of more than 3 days.	Exclusion criteria were need for dialysis and contraindication for an arterial line in the femoral artery.	Conventional Care: 65 (11)<br>Goal Directed therapy: 67 (12)	Diabetes, Hypertension, Chronic heart failure, coronary artery disease, heart failure, arrythmia, valvular disorder.	GI Surgery	Conventional Care. Goal Directed therapy.	Change in creatinine	postoperative complications, renal outcomes as well as 1-year mortality

Weinberg 2017[109]	• Australia<br>• English<br>• 2017<br>• 52 Patients<br>• Inclusion Criteria: adult patients undergoing elective .Pancreaticoduodenectomy	Exclusion criteria: age less than 18 years, pregnancy, pre-operative coagulopathy, renal impairment (creatinine >250umol.L-1), chronic liver disease (Child Pugh classification), American Society Anaesthesiology physical status > class IV, and patients undergoing distal, central or total pancreatectomy or pancreatic enucleation	Conventional Care: 68 (54-75)<br>Goal Directed fluid therapy: 61 (53-72)<br>median (interquartile range)	Not Clearly stated	HPB Surgery	Conventional Care<br>Goal Directed fluid therapy	length of hospital stay (defined as discharge from theatre to formal discharge from the acute hospital ward).	preoperative patient characteristics, body mass index, American Society of Anaesthesiologists (ASA) class, comorbidities and preoperative biochemical and haematological laboratory test results. Operative details collected included anaesthetic technique, fluid balances, intraoperative blood transfusion requirements, duration of surgery, and use of vasoactive medications (type and amount). Postoperative details included detailed fluid intervention for postoperative Days 1 and 2 (type and amount), detailed fluid balances for postoperative Days 1 and 2, blood transfusion requirements, daily body weight, routine biochemistry and haematology, and drain output.

Weinberg 2019[110]	• Australia<br>• English<br>• 2019<br>• 50 Patients<br>• Inclusion Criteria: adult patients (greater than 18 years) undergoing elective major liver resection. defined major resection as a resection of 3 or more liver segments.	Exclusion Criteria: preoperative coagulopathy (international normalized ratio>1.5), thrombocytopenia (platelet count<75×10^9L-1), renal impairment (creatinine>250 µmol.L-1), hepatic insufficiency (bilirubin>30 µmol.L-1, albumin<25 g.dL-1, alkaline phosphatase>300U.L-1, alanine transaminase>50U.L-1), American Society Anesthesiology class > III, impaired left ventricular function (ejection fraction<40%), atrial fibrillation, moderate or severe tricuspid regurgitation or any impairment of right ventricular function.	Conventional Care: 61 (52-73)<br>Restrictive therapy: 64 (57-71)<br>median (interquartile range)	Liver Cancer, Diabetes, Dyslipidaemia, COPD, Hypertension, Ischaemic Heat disease, peripheral vascular disease.	HPB Surgery	Conventional Care<br>Restrictive therapy	duration of hospital stay, which was measured from the time surgery was completed (last suture) to hospital discharge.	i) expected number of complications per patient ii) number (proportion) of participants with at least 1 complication iii) volume of intraoperative fluid use (expressed as a total volume, and as millilitres per kilogram per hour); iv) intraoperative fluid balances; v) use of intraoperative vasoactive medications.

Wu 2017 2[111]	• Taiwan<br>• English<br>• 2017<br>• 80 Patients<br>• Inclusion criteria: older than 20 years who had undergone elective craniotomy for supratentorial brain tumour resection during May	Exclusion Criteria: age >70 years; recurrent tumours; BMI <18.5 or >27.0 kg m–2; a history of cardiac dysfunction, such as coronary artery diseases, New York Heart Association Functional Classification (NYHA) class II or higher heart failure, and arrhythmia; renal insufficiency with an estimated glomerular filtration rate of <60 ml.min–1 per 1.73 m2; and chronic obstructive pulmonary disease and surgery in the prone position.	Low SVV: 55 (49–62)<br>High SVV: 58 (54–63)<br>median (inter-quartile range)	CNS Cancer, Asthma, Hypertension, Cardiac disease, Diabetes	Neurosurgery	SVV ≥10% group<br>SVV ≥18% group	Intra-operative physiology: Operation time, Transfusions, SVV, BNP, Cardiac Index, Heart rate, Blood pressure, Lactate.	ICU LOS, Hospital LOS, Post-op Complications, Post-op Neurological events
Wu 2017a 1[112]	• China<br>• English<br>• 2017<br>• 678 Patients<br>• Inclusion Criteria: 1) patients were 65–80 years old; 2) patients had American Society of Anesthesiologists (ASA) physical status grade I to III disease with a predicted surgery time N60 min; 3) no surgery for pre-existing renal disease; 4) current left ventricular ejection fraction N50%; and 5) no sign of cardiac dysfunction.	Exclusion Criteria: 1) patients used non-steroidal anti-inflammatory drugs during the past month; 2) patients had heart failure during the past 2 months; 3) patients had myocardial infarction during the past month (confirmed by blood-specific enzymes); 4) current severe pulmonary function insufficiency; 5) current intermediate to severe pulmonary hypertension; and 6) chronic<br>kidney diseases or renal dysfunction (confirmed by previous physician's diagnosis).	Level 1: 73 (7)<br>Level 2: 73 (6)<br>Level 3: 74 (5)	GI Cancer<br>Chronic Hypertension	GI Surgery	MAP, 65–79mmHg<br>MAP, 80–95mmHg;<br>MAP, 96–110mmH	incidence of AKI	incidence of surgical site infection, hospital-acquired pneumonia, stroke, admission to the intensive care unit (ICU), stay in the ICU, length of hospital stay, and 28-day mortality.

Xu 2017[113]	• China<br>• English<br>• 2017<br>• 172 patients<br>• Inclusion criteria: aged between 18 and 60 years; American Society of Anaesthesiologists physical (ASA) status I-II category; body mass index between 18.5 and 25kg.m-2	Exclusion Criteria: severe impairment of renal and cardiac function (New York Heart Association classes III-IV); Preoperative abnormal lung function (forced expiratory volume in 1s <50% of the predicted values); systemic or local active infections (clinically defined, leukocytosis, or the body temperature >38 ℃); Preoperative acid-base or electrolyte imbalance; Intraoperative frequent cardiac arrhythmias and OLV time <60 min.	Traditional Fluid Therapy: 49(5)<br>Goal Directed Fluid Therapy: 49(6)	non-small-cell lung cancer	Thoracic Surgery	Control – conventional fluid therapy<br>GDFT Programme	PaO_2/FiO_2	pulmonary variables and lung mechanics, inflammatory response, the incidence of postoperative pulmonary complications, and the length of hospital stay.
Yin 2018[114]	• China<br>• English<br>• 2018<br>• 45 Patients<br>• Inclusion Criteria: aged 65-90 years, with a body mass index (BMI) between 18-28 kg.m-2 and an American Society of Anesthesiology classification of II-III, normal liver and renal function.	Exclusion Criteria: Not clearly stated	Control: 68.3(5.8)<br>GDFT: 69.4(6.4)	Hypertension<br>Coronary heart disease<br>Diabetes mellitus<br>Cerebrovascular disease	GI Surgery	Control:Conventional fluid infusion<br>GDFT Programme	moderate or severe postoperative complications within 30 days.	Return of gastrointestinal function and the length of hospital stay after operation. The extubation time, intraoperative hemodynamic parameters and the use of vasoactive agents

Zakhaleva 2012[115]	• USA<br>• English<br>• 2012<br>• 91 Patients<br>• Inclusion Criteria: All patients over 18 years of age presenting to a single centre for elective bowel resection, defined as open or laparoscopic surgery with primary anastomosis, who were able to give their consent	Exclusion Criteria: admission as an emergency case; failure to sign consent; recent oesophageal or upper airway surgery; moderate or severe aortic valve disease; and congestive heart failure.	Control (Clinical indicators) - median (range).: 57 (27–79) Intervention (Stroke volume measurement) - median (range): 57 (22–80)	Not Clearly stated	GI Surgery	Control (Clinical indicators) Intervention (Stroke volume measurement).	30 day mortality	1. Operating time (min) from first skin incision to application of the dressing<br>2. Estimated blood loss (ml) recorded by the anaesthesiologist<br>3. Intra-operative fluid (l) administered and recorded by the anaesthesiologist<br>4. Postoperative fluid (l) administered and recorded by the colorectal resident<br>5. Postoperative day of out of bed and ambulation recorded by the physiotherapist<br>6. Postoperative day of the first passage of flatus reported by the patient<br>7. Removal of the epidural catheter (postoperative day) recorded by the anesthesiologist<br>8. Resumption of diet (postoperative day) recorded by the ward nurse<br>9. Hospital stay defined as the number of nights from surgery to discharge

Zhao 2018[116]	• China<br>• English<br>• 2018<br>• 88 Patients<br>• Inclusion Criteria: (1) aged over 60 years; (2) patients conformed to American Society of Anesthesiologist (ASA) physical status class II or III; and (3) those scheduled for radical operations of gastrointestinal cancers (stomach, colon or rectum).	Exclusion Criteria: (1) suffered from severe aortic regurgitation or rapid arrhythmia (atrial fibrillation, ventricular tachycardia); (2) patients conformed to American Society of Anesthesiologist (ASA) physical status class I or IV; and (3) those needed intra-aortic counterpulsation.	Control: 70.41 (8.99)<br>GDFT: 67.77 (7.99)	GI Cancer	GI Surgery	Control: Routine Fluid Infusion<br>GDFT	fluid intake, quantity of crystalloid and colloid, urine output, blood loss volume and dose of vasoactive agent	Postoperative complications, time to first bowel movement, postoperative hospitalization and total cost

Zheng 2013[117]	• China<br>• English<br>• 2013<br>• 65 patients<br>• Patients 60–80 years old with coronary artery disease (as per WHO diagnostic criteria) and New York Heart Association (NYHA) classification II-III with normal liver and renal function, body mass index (BMI) 18–24 kg.m^{-2} and anticipated intraoperative blood loss <600 ml undergoing moderate- to high-risk elective GI surgery.	• Scheduled for emergency or low-risk surgery<br>• American Society of Anesthesiologists (ASA) grade III<br>• Received prior fluid therapy (2,500 ml.-day over the 48 h before the surgery)<br>• Presence of congenital heart disease, cardiomyopathy, rheumatic heart disease, or pulmonary heart disease<br>• Preoperative use of vasoactive drugs (e.g. digoxin, nitroglycerine, nifedipine) for 3 months or more<br>• Preoperative or intraoperative administration of diuretics<br>• Preoperative acid–base imbalance or electrolyte (Na, K, Ca, Mg) imbalance<br>• Difficulty (or contraindication to) placing a central venous catheter<br>• Inability to cooperate (e.g. mental disorder, disturbance of consciousness, mental retardation<br>• Presence of blood-borne infectious disease (e.g. hepatitis B, hepatitis C, syphilis, acquired immunodeficiency	Control (median age (IQR)): 67 (63.75, 74)<br>GDT: 68 (64, 73.25)	Coronary artery disease	GI Surgery	Control: fluid infusion according to standard guidelines<br>Intervention: GDFT	Adverse cardiac events (myocardial ischaemia, acute myocardial infarction, serious arrythmias, congestive heart failure, cardiac death.	Postoperative GI dysfunction, time to extubation, length of intensive care unit (ICU) stay, GI recovery, and length of hospitalization.

		syndrome)<br>• Had undergone surgery twice after admission.							

Inodilators								
Baysal 2014[118]	• Turkey<br>• English<br>• 2013<br>• 128 patients<br>• Diagnosis of mitral valve insufficiency with or without coronary artery disease and an LVEF of 45% or less.	• Unstable angina<br>• Diabetes mellitus treated with insulin<br>• Clinical findings of acute or chronic renal failure (serum creatinine 41.5 mg.-dL)<br>• Severe hepatic disease (alanine aminotransferase or aspartate amino-transferase >100 U.-L), severe chronic obstructive pulmonary disease (FEV1 <50% of predicted or <2.0 litres)<br>• History of prior CABG surgery or myocardial infarction (MI) within the previous month<br>• Emergent operations<br>• Patients on inotropic support before surgery<br>• Aortic valvular disease<br>• Infective endocarditis	Intervention: 56.73(11.71)<br>Control: 58.41(9.83)	Mitral valve insufficiency, tricuspid valve insufficiency, coronary artery disease, preoperative, low preoperative LVEF, pulmonary hypertension.	Cardiac Surgery	Control: clinical indicators<br>Intervention: Levosimendan	Primary outcome: postoperative renal function measured by serum creatinine and eGFR	Secondary outcomes:<br>1- aortic cross-clamp time<br>2- cardio-pulmonary bypass time<br>3- inotropic support<br>4- intra-aortic balloon pump need<br>5- prolonged mechanical ventilation<br>6- pneumonia<br>7- perioperative myocardial infarction<br>8- cerebrovascular event (stroke, transient ischemic attack)<br>9- atrial fibrillation and other rhythm disturbances<br>10- need for early renal replacement therapy (RRT)<br>11- reoperation secondary to bleeding<br>12- ICU stay<br>13- hospital stay

Bragadottir 2013[119]	• Sweden<br>• English<br>• 2013<br>• 34 patients<br>• Preoperative serum creatinine < 10^5 µmol.-L in patients undergoing elective cardiac surgery with CPB.	Patients requiring inotropic or vasoactive support or with significant postoperative bleeding.	Placebo: 69.3 (2.71)<br>Levosimendan: 65.5 (3.27)	Hypertension, Coronary heart disease, valvular disease	Cardiac surgery	Placebo<br>Levosimendan	Primary outcome not defined	Secondary outcomes not defined
Cholley 2017[120]	• France<br>• English<br>• 2015<br>• 336 patients<br>• Patients scheduled for CABG surgery with CPB alone or combined with valve surgery if they had a left ventricular ejection fraction of 40% or lower	Younger than 18 years, pregnancy, renal failure (creatinine clearance <30 mL.min-1), liver failure (prothrombin ratio <50%), emergency surgery, hypotension (mean arterial pressure <60 mm Hg), or tachycardia (heart rate >120min-1).	Mean (SD)<br>Intervention 69 (10)<br>Control 67 (10)	Cancer, cirrhosis	Cardiac surgery	Control: placebo<br>Intervention: levosimendan	Composite of 3 elements reflecting low cardiac output syndrome: catecholamine (ie, dobutamine, epinephrine, norepinephrine, or milrinone) infusion persisting beyond 48 hours after the initiation of the study drug, the need for circulatory mechanical assist devices in the post-operative period or for renal replacement therapy at any time during intensive care unit stay.	In-hospital mortality, mortality at days 28 and 180, each component of the primary endpoint, number of days with circulatory mechanical assist de-vice, number of days with catecholamine infusion, number of days with renal replacement therapy and number of renal re-placement therapy kits that were used for each patient, and number of ventilator-free days, out-of-intensive care unit and out-of-hospital days at day 28,and total hospital and intensive care unit lengths of stay
Desai 2018[121]	• India<br>• English	Patients undergoing emergency/redo/combined CABG, those requiring	Mean (SD)<br>Control: 60.17(5.72)<br>Intervention 61.67(7.03)	Diabetes, hypert	Cardiac surgery	Control: conventional inotropes/vasopres	Haemodynamic parameters including serum	Postoperative AF, conversion to CPB, IABP requirement, serum

	• 2015<br>• 60 patients<br>• Patients between 35 and 75 years of age with severe LV dysfunction (LV ejection fraction <30% determined by preoperative transthoracic echocardiography)	preoperative pharmacological or mechanical support, renal, and hepatic dysfunction		ension, renal disease , coronar y artery disease		sor Intervention: Levosimendan + vasopressor	lactate	creatinine at day 2, AKI, LCOS, Noradrenaline requirement, ICU stay, hospital stay, mortality
Erb 2014[122]	• Germany<br>• English<br>• 2009<br>• 23 patients<br>• Patients aged18 years with ischaemic cardiomyopathy, scheduled for elective coronary artery bypass graft (CABG) surgery with or without valve surgery, with LVEF 30% (diagnosed by preoperative echocar-diography or fluoroscopy during coronary catheterization)	pregnancy and/or breast feeding, liver insufficiency Child–Pugh class B or C or Model for End-stage Liver Disease>17, disease or recent operation (<2 months prior) of the oesophagus or upper airway, neurological or psychiatric disorder, diabetes mellitus treated with sulphonyl urea drugs, HIV infection or hepatitis B or C infection, or alcohol abuse	Mean (SD)<br>Intervention 69.5(11.5)<br>Control 63.4(7.8)	NYHA class 4, stroke, lung disease , pulmon ary hypert ension, COPD, renal insuffici ency, chronic haemo dialysis, Diabete s, pacem aker,	Cardiac surgery	Control: placebo<br>Intervention: levosimendan	Haemodynamic variables, need for vasoactive medication, SOFA score.	Reintubation due to pneumonia and/or tracheobronchitis; reintubation due to lung oedema; sepsis; renal insufficiency requiring continuous renal replacement therapy; reoperation; readmission to hospital; persistent haemodynamic instability with the need of implantation of a VAD; cardiopulmonary resuscitation; death
Landoni 2017[123]	• Australia<br>• English<br>• 2016<br>• 506 patients<br>• Perioperative cardiovascular dysfunction, which was defined as the presence of at least one of the following criteria: a preoperative left ventricular ejection fraction of less than 25%, preoperative support with an intraaortic balloon pump, or	Previous adverse response to levosimendan, inclusion in another randomized trial, receipt of levosimendan in the previous 30 days, receipt of a kidney or liver trans-plant, liver cirrhosis, a decision to use extracorporeal membrane oxygenation, or the presence of a do-not-resuscitate order, emergency operation	Median (IQR)<br>Intervention 66 (58-74)<br>Control 66 (58-72)	Myocar dial infarcti on, AF, cardiog enic shock, COPD, stroke/ TIA, PVD, Diabete s,	Cardiac surgery	Control: placebo<br>Intervention: levosimendan	30-day mortality	Acute kidney injury, a need for renal-replacement therapy, a composite outcome of death and need for renal-replacement therapy, duration of mechanical ventilation, and durations of stay in the ICU and hospital, need for advanced mechanical circulatory support, myocardial

	the need for support with an intraaortic balloon pump or high-dose inotropic support in order to be weaned from cardiopulmonary by-pass or at any time within the first 24 hours after surgery							infarction, type 1 or type 2 neurologic damage, need for tracheostomy, sepsis, pneumonia, and mediastinitis.
Levin 2012[124]	• USA<br>• English<br>• 2008<br>• 252 patients<br>• Patients with CAD and SLVD with an ejection fraction <25% scheduled to undergo cardiac surgery with CPB	Not defined	Mean<br>Intervention 63.7<br>Control 62.9	Myocardial infarction, hypertension, diabetes,	Cardiac surgery	Control: placebo<br>Intervention: Levosimendan	postoperative LCOS and mortality	Difficult weaning from cardiopulmonary bypass (CPB) and the requirements for inotropes, vasopressors and IABP
Mehta 2017[125]	• USA<br>• English<br>• 2016<br>• 882 patients<br>• Patients 18 years of age or older, were scheduled to undergo cardiac surgery with the use of cardiopulmonary bypass, and had a left ventricular ejection fraction of 35% or less as assessed within 60 days before surgery	Not defined	Median (IQR)<br>Intervention 65 (59-73)<br>Control 65 (58-72)	Hypertension, diabetes mellitus, hypercholesteroloemia, chronic lung disease, CKD, myocardial infarction, stroke, PVD, cerebrovascular disease, heart failure	Cardiac surgery	Control: Control: placebo<br>Intervention: levosimendan	Two composite primary efficacy end points: The first was the four-component composite of death through day 30, renal-replacement therapy through day 30, perioperative myocardial infarction through day 5, or use of a mechanical cardiac assist device through day 5. The second was the two-component composite of death through day 30 or use of a mechanical cardiac assist	Incidence of low cardiac output syndrome, postoperative use of secondary inotropes at or beyond 24 hours after the start of the infusion of levosimendan or placebo, and postoperative duration of stay in an intensive care unit.

							device through day 5.	
Ristikankare 2012[126]	• Finland<br>• English<br>• Not stated<br>• 60 patients<br>• Coronary artery disease with an impaired left ventricular ejection fraction (0.50), signs of acute ischemic congestive heart failure, or both.	Patients with a previous administration of levosimendan within the preceding 30days and predialysis or end-stage chronic renal failure	Mean (SD)<br>Intervention 64 (10)<br>Control 64 (10)	Diabetes, hypertension	Cardiac surgery	Control: placebo<br>Intervention: levosimendan	Primary outcomes not defined, mean change in cystatin C, creatinine, urine NAG.	mean change in haemodynamic parameters
Shah 2014 [127]	• India<br>• English<br>• 2012<br>• 50 patients<br>• Patients with single vessel disease requiring graft to left anterior descending artery, double vessel disease, and triple vessel disease with LVEF less than 30% and myocardial perfusion scan showing viability of the affected territory	Patients undergoing urgent, emergent, congenital, valve, aortic, or combined surgeries or who were treated with levosimendan within 3 months or with other inotropes within the previous week and patients with a preoperative intraaortic balloon pump (IABP), significant pulmonary disease, renal dysfunction, liver dysfunction, redo-CABG, or arrhythmias with bundle branch block	Mean (SD)<br>Intervention 59.91 (8.8)<br>Control 61.32 (7.64)	Diabetes, myocadial infarction, myocardial infarction	Cardiac surgery	Control: placebo<br>Intervention: levosimendan	Primary outcomes not defined. Need for vasopressors, hypotension	Headache, nausea, vomiting.
Sharma 2014[128]	• India<br>• English<br>• 2012<br>• 40 patients<br>• Coronary artery disease, severe LV dysfunction (ejection fraction<30%), and severe MR, requiring on-pump CABG with mitral valve repair.	Previous cardiac surgery, any other valve pathology, diabetes mellitus treated with sulfonylurea drugs, renal failure (plasma creatinine>1.5 mg/dL), hepatic dysfunction (alanine aminotransferase or aspartate aminotransferase>100 U/dL), severe chronic obstructive pulmonary dis-ease (forced expiratory volume in 1 s<50% of predicted or<2.0 L), recent myocardial infarction	Intervention 53.95(12.06)<br>Control 54.5(57.87)	Hypertension, diabetes, unstable angina, myocardial infarction	Cardiac surgery	Control: placebo<br>Intervention: levosimendan	Primary outcomes not defined. Low cardiac output syndrome(LCOS), defined as cardiac index (2.2 L/min/m2); elevated pulmonary capillary wedge pressure (16 mm Hg),PaO2<60	Secondary outcomes not defined.

		(<30days), preoperative intubation, and emergency surgery					mm Hg; perioperative myocardial infarction; renal failure; stroke; neurologic change persisting<48 h was considered as a transient ischemic attack; sepsis ; adultrespiratory distress syndrome; hypoxemia;and postoperative mortality	
Zangrillo 2017[129]	• Multicenter (Italy)<br>• English<br>• 2016<br>• 506 patients<br>• Patients requiring perioperative hemodynamic support (defined as a preoperative left ventricular ejection fraction (LVEF)<25%, preoperative need for intra-aortic balloon pump (IABP), need for high-dose inotropic drugs or IABP during weaning from cardiopulmonary bypass or within 24 hours of surgery.	Previous adverse response to levosimendan, inclusion in another randomized trial, receipt of levosimendan in the previous 30 days, receipt of a kidney or liver transplantation, liver cirrhosis, emergency surgery, a decision to use extra-corporeal membrane oxygenation already made, or the presence of a do-not-resuscitate order	Mean (IQR)<br>Intervention 68 (60-76)<br>Placebo 68 (63-78)	COPD, stroke/ TIA, myocardial infarction, atrial fibrillation, PVD, diabetes, cardiogenic shock	Cardiac surgery	Control: placebo<br>Intervention: levosimendan	Acute kidney injury	Need for renal replacement therapy, need for renal replacement therapy or 30 day mortality, myocardial infarction, type 1 neurologic damage, type 2 neurologic damage, septic shock, pneumonia, mortality, serum creatinine changes.
Atalay 2016[130]	• Turkey<br>• English<br>• 2016<br>• 58 patients<br>• Inclusion Criteria: elective isolated CABG operations in end-stage renal disease patients	Exclusion criteria: NA	Control: 66.5<br>Levosimendan: 69.5	Coronary artery disease	Cardiac Surgery	Control – Placebo<br>Levosimendan 24-hour infusion of 0.03-0.05 µg/kg/kg/min	Cardiac output and cardiac index	Hemodynamic parameters, Pulmonary capillary wedge pressure, blood lactate and cTnI levels

**Loop Diuretics**								
Bebawy 2013[131]	• USA<br>• English<br>• N/A<br>• 23 patients<br>• All subjects were presenting for primary or metastatic brain tumor resection	Any patient with cardiovascular disease that might preclude the safe use of mannitol and/or furosemide	Mean (SD)<br>Intervention 51 (16.6)<br>Control 52.1 (15.6)	N/A	Neurosurgery	Control: placebo<br>Intervention: furosemide	Primary outcomes not defined. Urine output, plasma electrolytes, lactic acid and mean arterial pressure measured at different intervals.	Secondary outcomes not defined.
Danelich 2018[132]	• USA<br>• English<br>• N/A<br>• 23 patients<br>• All subjects were presenting for primary or metastatic brain tumor resection	Any patient with cardiovascular disease that might preclude the safe use of mannitol and/or furosemide	Mean (SD)<br>Intervention 51(16.6)<br>Control 52.1(15.6)	N/A	Neurosurgery	Control: placebo<br>Intervention: furosemide	Primary outcomes not defined. Urine output, plasma electrolytes, lactic acid and mean arterial pressure measured at different intervals.	Secondary outcomes not defined.
Lassnigg 2000 a[63]	• Austria<br>• English<br>• 2016<br>• 123 patients<br>• Patients undergoing elective cardiac surgery with normal renal function (baseline serum creatinine value: 2.0 mg.dl-1).	Patients with problems with urine collection or who required reoperation for bleeding complications	Mean (SD)<br>Intervention (Dopamine): 63 (10)<br>Intervention (Furosemide): 63 (10)<br>Control: 65 (10)	Diabetes, hypertension	Cardiac surgery	Control group: placebo<br>Intervention groups: dopamine and furosemide	Change in serum creatinine values over time	Acute renal injury (ARI), changes in creatinine clearance over time, urine output per hour, volume intake per hour, serum sodium, serum potassium, other parameters of renal function, necessity for hemodialysis or hemofiltration, and hospital mortality rate.

Luo 2013 a[133]	• China<br>• Chinese<br>• 2013<br>• 120 patients<br>• Patients undergoing elective gynecological open surgery under general anesthesia	Exclusion Criteria: na	Intervention: 39 (6)<br>Control: 41 (7)	Coronary heart disease	Cardiac Surgery	control group, furosemide 0. 05 mg/kg (F0.5) group and furosemide 0. 1 mg/kg (F1) group	Total time of surgery and intraoperative net fluid infusion volume	Urine Volume, Intraoperative oliguria
Luo 2013 b[133]	• China<br>• Chinese<br>• 2013<br>• 120 patients<br>• Patients undergoing elective gynecological open surgery under general anesthesia	Exclusion Criteria: na	Intervention: 40 (5)<br>Control: 41 (7)	Coronary Heart Disease	Cardiac surgery	control group, furosemide 0. 05 mg/kg (F0.5) group and furosemide 0. 1 mg.kg-1 (F1) group	Total time of surgery and intraoperative net fluid infusion volume	Urine Volume, Intraoperative oliguria
Mahesh 2008[134]	• United Kingdom<br>• English<br>• N/A<br>• 42 patients<br>• Patients at higher risk of postoperative renal dysfunction were selected, based on the presence of one or more of following preoperative criteria: renal insufficiency (serum creatinine>130mmol.l-1), LV ejection fraction (LVEF)<50%, congestive heart failure, diabetes (requiring oral hypoglycemic agents and/or insulin), procedures involving prolonged CPB such as coronary artery bypass grafts with valvular surgery and redo cardiac surgery.	Patients with end-stage dialysis-dependent renal failure	Mean (SD)<br>Intervention: 69.6 (8.3)<br>Control: 73.0 (9.1)	Diabetes	Cardiac surgery	Control group: placebo<br>Intervention: furosemide	No primary outcomes defined. Changes in urine and serum markers, renal dysfunction, need for IABP, dialysis and death.	No secondary outcomes defined.
**N-Acetylcysteine**								
Abd El Aal 2013[135]	• Egypt<br>• English<br>• N/A	Patients with pre-existing renal impairment (preoperative plasma	Mean (SD)<br>Intervention: 28.5 (7.62)<br>Control: 29.3 (7.52)	N/A	Cardiac surgery	Control: N-acetyl cysteine + 5% glucose	No primary outcomes defined.	No secondary outcomes defined.

	• 60 patients<br>• Adult patients undergoing elective open heart surgery for valve replacement	creatinine concentration >120mmol/l), those who had undergone redo cardiac surgery, those who had type 2diabetes and were on insulin therapy, those requiring emergency cardiac surgery, those who had chronic inflammatory disease and were on immunosuppressors, those who were on chronic moderate-to-high-dose corticosteroid therapy (Z10 mg/day prednisone or equivalent), and those who were 18 years of age or younger				Intervention: 5% glucose	Changes in postoperative levels of serum creatinine and urine output, duration of mechanical ventilation, length of ICU stay and hospital stay.	
Adabag 2008[136]	• USA<br>• English<br>• 2006<br>• 102 patients<br>• Patients with preexisting CKD undergoing elective cardiac surgery	Patients who were on dialysis before the surgery, those who were operated on urgently, patients with renal transplantation, and those who received an intravenous contrast agent within 4 days before surgery	Mean (SD)<br>Intervention 70 (9)<br>Control 72 (9)	Diabetes, hypertension, chronic lung disease, peripheral arterial disease, cerebrovascular disease, hyperlipidaemia, myocardial infarction	Cardiac surgery	Control: placebo<br>Intervention: N-acetyl cysteine	Maximal change in creatinine concentration from baseline anytime within 7 days after surgery	Development of AKI (defined as >0.5 mg/dL or ≥25% increase in creatinine from baseline) by postoperative days 5, 7, and 30; postoperative hemodialysis; operative mortality; and lengths of stay in the intensive care unit (ICU) and the hospital

Ali Karami 2016[137]	• Iran<br>• English<br>• 2014<br>• 84 patients<br>• Patients over 70 years old, confirmed renal failure, serum creatinine above 1.4 mg/dL, heart ejection fraction <35%, concomitant valve and bypass surgery, history of previous coronary artery surgeries and diabetes under treatment with oral agents or insulin.	Emergency or off-pump CABG, positive history of heart transplantation, acute renal failure, hypersensitivity to NAC or consumption of NAC in 5 days before the operation.	Mean (SD)<br>Intervention (NAC) 65.88 (9.35)<br>Intervention (NAC + vitamin C) 62.42 (10.22)<br>Intervention (placebo) 65.63 (11.36)	N/A	Cardiac surgery	Control: placebo<br>Intervention groups: NAC and NAC + vitamin C	Primary outcomes not defined. Mechanical ventilation, re-intubation, second surgery, MI, CVA, mediastinitis, sepsis, renal replacement therapy, length of ICU stay, changes in serum creatinine levels, adverse drug reactions.	Secondary outcomes not defined.
Amini 2018 c[138]	• Iran<br>• English<br>• 2018<br>• 272 patients<br>• adult patients with New York Heart Association (NYHA) class of I-III undergoing elective off-pump CABG at a teaching hospital were recruited for this clinical trial	change from off-pump to on-pump surgery, known drug allergy, history of chronic obstructive pulmonary disease (COPD), anemia, congestive heart failure (CHF), active sepsis, preoperative ejection fraction lower than 40%, preoperative creatinine above 1.3 mg/dL, use of any nephrotoxic drugs within the last week, coronary angiography within the last 2 days, intraoperative transfusion of more than 2 units of red blood cells (RBC), perioperative use of intra-aortic balloon counterpulsation (IABP), perioperative requirement for high-dose vasopressors and any intraoperative life-threatening events such as fatal arrhythmias, excessive	Mean (SD)<br>Intervention (selenium) 58.21(10.54)<br>Intervention (NAC) 60.03(10.37)<br>Intervention (vitamin C) 60.46(10.03)<br>Control (placebo) 58.72(8.57)	MI, hypertension, diabetes, drug abuse, hyperlipidaemia	Cardiac surgery	Control: placebo<br>Intervention groups: NAC, vitamin C, selenium	Primary outcomes not defined. AKI, mechanical ventilation duration, ICU length of stay, hospital length of stay, in-hospital mortality	Secondary outcomes not defined

		bleeding, or desaturation						
Ayhan 2012 a[139]	Turkey<br>English<br>N/A<br>60 patients<br>patients with normal renal function(Cr< 1.5 mg dl-1) scheduled for elective coronary artery bypass grafting surgery (CABG) with CPB	Patients with end-stage obstructive / restrictive pulmonary disease, renal failure, liver failure, sepsis, multiorgan failure, having previous cardiac surgery and emergency surgery, severe congestive heart failure (left ventricle fraction of ejection <35%), allergy to the drugs	Intervention (NAC in CPB): 58.6(9)<br>Intervention (NAC)<br>Control (placebo) 65(12.1)<br>59.9(9)	N/A	Cardiac Surgery	Control: placebo<br>Intervention groups: NAC and NAC during CPB	Primary outcomes not defined.<br>Haemodynamic data (MAP, heart rate, CVP), BUN, creatinine, blood and urine electrolytes and beta-2 microglobulin were measured, and albumin/creatinine ratio, creatinine clearance and fractional sodium excretion, side effects of NAC (rash, wheezing, superficial phlebitis, hemolysis and neutropenia) and the number of patients who required dialysis were recorded.	Secondary outcomes not defined.
Ayhan 2012 b[139]	Turkey<br>English<br>N/A<br>60 patients<br>patients with normal renal function(Cr< 1.5 mg dl-1) scheduled for elective coronary artery bypass grafting surgery (CABG) with CPB	Patients with end-stage obstructive / restrictive pulmonary disease, renal failure, liver failure, sepsis, multiorgan failure, having previous cardiac surgery and emergency surgery, severe congestive heart failure (left	Intervention (NAC in CPB): 58.6(9)<br>Control (placebo) 65(12.1)<br>59.9(9)	N/A	Cardiac Surgery	Control: placebo<br>Intervention groups: NAC and NAC during CPB	Primary outcomes not defined.<br>Haemodynamic data (MAP, heart rate, CVP), BUN, creatinine,	Secondary outcomes not defined.

		ventricle fraction of ejection <35%), allergy to the drugs					blood and urine electrolytes and beta-2 microglobulin were measured, and albumin/creatinine ratio, creatinine clearance and fractional sodium excretion, side effects of NAC (rash, wheezing, superficial phlebitis, hemolysis and neutropenia) and the number of patients who required dialysis were recorded.	
Barr 2008 b[51]	USA<br>English<br>2006<br>79 patients<br>patients who were >18 years old; who were undergoing elective, urgent, or emergency cardiac bypass and/or valve surgery; and who had a preoperative creatinine clearance of40 mL.min-1.	active hemodialysis, uncontrolled glaucoma (because of possible exacerbation by fenoldopam), pregnancy, nausea and vomiting, or sensitivity to metabisulfite (the preservative for fenoldopam). Patients with preoperative creatinine clearance<40 mL/min but with creatinine>1.1 (generally thin, elderly females)	Intervention (fenoldopam): 77.2 (1.2)<br>Intervention (NAC): 73.8(2.2)<br>Intervention (NAC + fenoldopam): 73.5(2.0)<br>Control (placebo): 72.4(2.0)	Diabetes, peripheral vascular disease	Cardiac surgery	Control: placebo<br>Intervention groups: NAC, NAC+ fenoldopam, fenoldopam	The primary outcome was the difference between the preoperative and postoperative day 3 creatinine clearances.	Change in weight between preoperative and postoperative day 3, the percent change in creatinine clearance postoperative day 14, the length of postoperative critical care unit stay, the length of postoperative total hospital stay, the need for renal replacement therapy any time during the postoperative hospitalization, mortality, and hospital costs.

Burns 2005 [140]	Canada<br>English<br>2004<br>295 patients<br>high-risk patients scheduled for elective or urgent CABG surgery	N/A	Intervention 68.9 (8.9)<br>Control 69.2 (9.7)	Diabetes, renal dysfunction, hypertension, recent MI, COPD, PVD, CVA, congestive heart failure,	Cardiac surgery	Intervention: NAC<br>Control: placebo	Proportion of patients developing postoperative renal dysfunction defined as an ab-solute increase in serum creatinine level of greater than 0.5 mg.dL-1 (44 µmol.L-1)or a 25% increase from baseline at any time within the first 5 postoperative days	Requirement for postoperative interventions (vasoactive medications, intra-aortic balloon pump insertion, renal dose dopamine, mechanical ventilation for at least 48 hours, or reintubation and re-operation within 24 hours) and post-operative complications (myocardial infarction, stroke, mediastinitis, and bloodstream infections). We recorded the requirement for renal replacement therapy (RRT) during hospitalization, hospital mortality, and ICU and hospital length of stay
El Hamamsy 2007 [141]	Canada<br>English<br>2004<br>100 patients<br>N/A	Emergency operations, acute myocardial infarction within fewer than 3 weeks, prior cardiac surgery, age older than 80 years, ejection fraction less than 20%, and concomitant procedures.	Intervention: 59.8 (7.8)<br>Control: 61.3 (7.4)	MI, angina, left ventricular dysfunction	Cardiac surgery	Intervention: NAC<br>Control: placebo	Mean postoperative release of cardiac troponin T levels between the two groups (1, 2, 4, 8, 12, and 24 hours postoperatively, then2, 3, and 4 days postoperatively) .	Rate of myocardial infarction (as defined by creatine kinase(CK-MB) level>50 and/or new Q wave on electrocardiogram in a given territory), renal function (creatinine), bleeding, low cardiac output syndromes, arrhythmias, and mean levels of CK-MB
Fischer 2005[142]	Germany<br>English<br>2015<br>Patients undergoing cardiac surgery	N/A	Intervention: NAC - NA<br>Control: placebo - NA	N/A	Cardiac surgery	NAC (100 mg/kg into the cardiopulmonary bypass prime	No primary outcomes defined.	serum creatinine, Creatinine clearance, Diuresis, heart rate, vascular pressures,

	with normal preoperative renal function					followed by infusion at 20 mg/kg/h; n = 20) or placebo		systemic and pulmonary vascular resistance, cardiac index, LV function
Haase 2007[143]	Canada<br>English<br>N/A<br>61 patients<br>Age of >70 years, preexisting renal impairment, New York Heart Association class III/IVb, valve surgery or complex cardiac surgery, redo cardiac surgery, insulin-dependent diabetes mellitus	Age of <18 years, known allergy or hypersensitivity to N-acetylcysteine, emergency cardiac surgery, planned off-pump cardiac surgery, enrolled in conflicting research study, known blood-borne infectious disease, chronic inflammatory disease on immunosuppression, chronic moderate to high-dose corticosteroid therapy, end-stage renal disease, patients receiving preoperative intravenous nitrates	Intervention 68.9 (9.7)<br>Control 68.3 (9.3)	Arterial hypertension, High cholesterol, atrial fibrillation, Recent myocardial infarction, on insulin-dependent diabetes mellitus, Chronic obstructive pulmonary disease, Peripheral vascular disease, Carotid disease, Stroke	Cardiac surgery	Intervention: NAC<br>Control: placebo	Increase in serum creatinine from baseline to peak	Changes in serum creatinine, cystatin C, urine output, chest tube drainage, postoperative AF, duration of mechanical ventilation, ICU and hospital stay.

Hynnien 2006 [144]	• Finland<br>• English<br>• 2006<br>• 70 Patients<br>• Inclusion Criteria: patients undergoing abdominal aortic surgery.	Exclusion Criteria: Patients with renal insufficiency (plasma creatinine >130 ug.L-1) or severe renal artery disease or planned suprarenal or renal artery clamp during the surgery	N-acetylcysteine: 66 (10)<br>Placebo: 67 (10)	Diabetes, Hypertension, Previous myocardial infarction, ischemic heart disease.	Vascular Surgery	N-acetylcysteine<br>Placebo	renal injury as measured by the increases in urinary N-acetyl-Beta-d-glucosaminidase (NAG) / creatinine ratio (indicator of renal tubular injury) and urinary albumin/ creatinine ratio (indicator of glomerular injury)	Renal function was assessed by measuring plasma creatinine and serum cystatin C concentrations
Prasad 2010[145]	• India<br>• English<br>• 2010<br>• 70 Patients<br>• Inclusion Criteria: patients admitted for elective OP-CABG surgery and deemed high risk: age older than 70 years,(b) diabetes mellitus, (c) hypertension, (d) baseline serum creatinine level higher than 133 mmol.l-1 (1.5mg.dl-1) and (e) ejection fraction less than 35%.	Exclusion criteria:<br>(a) known allergy or hypersensitivity to NAC,<br>(b) use of nephrotoxic drugs and nonsteroidal anti-inflammatory drugs,<br>(c) history of current or previous dialysis,<br>(d) prior renal transplant,<br>(e) conversion to cardiopulmonary bypass during surgery,<br>(f) patients needing intra-aortic balloon pump (IABP),<br>(g) patients on large doses of inotropes and<br>(h) pregnancy.	N-acetylcysteine: 55.60 (10.24)<br>Placebo: 57.77 (9.36)	Diabetes, Hypertension, COPD	Cardiac Surgery	N-acetylcysteine<br>Placebo	Serum creatinine level and glomerular filtration rate (GFR)	adverse reactions, duration of elective ventilation, duration of intensive care unit stay and duration of hospital stay.
Ristikankare 2006[146]	• Finland<br>• English<br>• 2006	Exclusion criteria: plasma creatinine level above 400 mmol litre^-1, chronic renal replacement therapy,	Mean (range)<br>N-acetylcysteine: 72 (44–87)	Diabetes, Hypertension,	Cardiac surgery	N-acetylcysteine<br>Placebo	Primary outcomes not defined.	urinary NAG/creatinine ratio, plasma creatinine and serum cystatin C. Risk of AKI, fluid

	• 80 Patients<br>• Inclusion Criteria: patients with mild to moderate renal failure undergoing elective heart surgery with cardiopulmonary bypass	kidney transplantation, and known or suspected allergy to NAC.	Placebo: 69 (51–81)	peripheral vascular disease				balance, vasoactive treatment
Sisillo 2008[147]	• Italy<br>• English<br>• 2008<br>• 254 Patients<br>• Inclusion Criteria: All consecutive patients scheduled for cardiac surgery and having at least moderate (stage 3 nephropathy) renal insufficiency were enrolled. Patients were included if their creatinine clearance, as calculated by the Cockcroft-Gault formula, was 60 mL.min-1.	Exclusion criteria: Patients in chronic peritoneal or hemodialytic treatment, those with known allergy to NAC, and those having received NAC for contrast-induced nephropathy prevention in the previous 7 days or contrast agents in the previous 72 hours (in elective cases). Patients undergoing emergency cardiac surgery were also excluded	N-acetylcysteine: 73 (6)<br>Placebo: 72 (6)	Diabetes, Hypertension, creatinine	Cardiac Surgery	N-acetylcysteine<br>Placebo	occurrence of ARF, defined as an increase in serum creatinine concentration >25% from baseline to the maximum value obtained within the 72-hr period following cardiac surgery	Maximal change in calculated creatinine clearance, doubling of serum creatinine according to the RIFLE definition, and the occurrence of major postoperative clinical events, including need for renal replacement therapy, acute myocardial infarction, prolonged (>48 hrs) mechanical ventilation, prolonged (>4 days) ICU stay, and death.
Song 2015[148]	• Korea<br>• English<br>• 2015<br>• 117 Patients<br>• Inclusion Criteria: Patients scheduled for elective multivessel OPCAB were included when they had one or more of the following: (i) preoperative serum creatinine higher than 1.4 mg/dL; (ii) left ventricular ejection fraction less than 35% or congestive heart failure (New York Heart Association functional class III or IV); (iii) age older than 70 years; (iv) diabetes; or (v) re-	Exclusion criteria: Exclusion criteria were: (i) history of renal replacement therapy; (ii) preoperative acute renal failure; (iii) administration of NAC within 5 days prior to surgery; and (iv) known hypersensitivity to NAC.	N-acetylcysteine: 68 (10)<br>Placebo: 69 (8)	Diabetes, Hypertension, COPD, Congestive heart failure, angina	Cardiac Surgery	N-acetylcysteine<br>Placebo	incidence of AKI	Serum creatinine, cystatin C, creatinine kinase-MB (CK-MB), troponin-T, high sensitivity C-reactive protein (hsCRP), white blood cell (WBC) count, percentage of neutrophil and Hct. duration of anaesthesia, and amounts of intravenous fluid, urine output, salvaged blood, pRBC transfusion and norepinephrine administered. During postoperative 48 h, amount of total intake, crystalloid and colloid

	operation							solutions administration, pRBC transfusion, urine output, drainage from the chest tube and dose of furosemide
Wijeysundera 2007[149]	• Canada<br>• English<br>• 2007<br>• 177 Patients<br>• Inclusion Criteria: 1) age ≥ 18 years; 2) pre-existing moderate renal insufficiency; and 3) elective coronary-artery-bypass-graft (CABG) and/or valve surgery with CPB. Moderate renal insufficiency was defined by an eGFR (Cockcroft-Gault equation) less than 60 mL·min-1	Exclusion criteria: 1) severe pre-existing renal insufficiency (creatinine concentration ≥ 300 µmol·L–1 or dependence on renal replacement therapy (RRT); 2) preoperative hemodynamic instability (intra-aortic balloon pump support or vasoactive medications); 3) N-acetylcysteine or angiographic contrast within 24 hr before surgery; 4) planned off-pump surgery; 5) planned deep-hypothermic-circulatory-arrest; or 6) prior adverse reaction to N-acetylcysteine.	N-acetylcysteine: 74 (8)<br>Placebo: 73 (9)	Hypertension, Diabetes, peripheral vascular disease, COPD	Cardiac Surgery	N-acetylcysteine<br>Placebo	72-hr % change in eGFR	renal replacement therapy, mortality, atrial fibrillation, vasoactive medications, and adverse effects.
Santana-Santos 2014[150]	• Brazil<br>• English<br>• 2014<br>• 70 Patients<br>• Inclusion Criteria: Patients with estimated glomerular filtration rate of 15 or greater and less than 60 mL.min.-1. Per 1.73 m-2, scheduled to undergo elective CABG	Exclusion criteria: allergy to NAC; participating in other studies and refusal to participate.	Control Group: 64.0 (9.0)<br>NAC Group: 65.0 (8.2)	Hypertension, Peripheral vascular disease, previous stroke, previous myocardial infarction,	Cardiac Surgery	Control Group<br>NAC Group	incidence of AKI	death by any cause, cardiovascular events (myocardial infarction, stroke, heart failure, life-threatening arrhythmia), and need of dialysis.

				diabetes, dyslipidaemia				
**Miscellaneous – Antioxidant**								
Amano 1994[151]	• Japan<br>• English<br>• 1994<br>• 19 Patients<br>• Adult patients undergoing coronary artery bypass surgery	Exclusion Criteria: NA	Intervention group: 58 (2.5)<br>Control group: 56 (2.3)	Coronary artery disease	Cardiac surgery	Intervention group<br>Control group	Primary outcomes not defined.	Mean arterial pressure, systemic vascular resistance, urine volume, creatinine clearance, renal excretory index, plasma renin level, plasma angiotensin II, Aldosterone concentration, fractional excretion of sodium
Amini 2018 a[138]	• Iran<br>• English<br>• 2018<br>• 272 patients<br>• adult patients with New York Heart Association (NYHA) class of I-III undergoing elective off-pump CABG at a teaching hospital were recruited for this clinical trial	change from off-pump to on-pump surgery, known drug allergy, history of chronic obstructive pulmonary disease (COPD), anemia, congestive heart failure (CHF), active sepsis, preoperative ejection fraction lower than 40%, preoperative creatinine above 1.3 mg/dL, use of any nephrotoxic drugs within the last week, coronary angiography within the last 2 days, intraoperative transfusion of more than 2 units of red blood cells (RBC), perioperative use of intra-aortic balloon counterpulsation (IABP), perioperative requirement for high-dose vasopressors	Intervention (selenium) 58.21(10.54)<br>Intervention (NAC) 60.03(10.37)<br>Intervention (vitamin C) 60.46(10.03)<br>Control (placebo) 58.72(8.57)	MI, hypertension, diabetes, drug abuse, hyperlipidaemia	Cardiac surgery	Control: placebo<br>Intervention groups: NAC, vitamin C, selenium	Primary outcomes not defined.<br>AKI, mechanical ventilation duration, ICU length of stay, hospital length of stay, in-hospital mortality	Secondary outcomes not defined

		and any intraoperative life-threatening events such as fatal arrhythmias, excessive bleeding, or desaturation						
Amini 2018 b[138]	• Iran<br>• English<br>• 2018<br>• 272 patients<br>• adult patients with New York Heart Association (NYHA) class of I-III undergoing elective off-pump CABG at a teaching hospital were recruited for this clinical trial	change from off-pump to on-pump surgery, known drug allergy, history of chronic obstructive pulmonary disease (COPD), anemia, congestive heart failure (CHF), active sepsis, preoperative ejection fraction lower than 40%, preoperative creatinine above 1.3 mg/dL, use of any nephrotoxic drugs within the last week, coronary angiography within the last 2 days, intraoperative transfusion of more than 2 units of red blood cells (RBC), perioperative use of intra-aortic balloon counterpulsation (IABP), perioperative requirement for high-dose vasopressors and any intraoperative life-threatening events such as fatal arrhythmias, excessive bleeding, or desaturation	Intervention (selenium) 58.21(10.54)<br>Intervention (NAC) 60.03(10.37)<br>Intervention (vitamin C) 60.46(10.03)<br>Control (placebo) 58.72(8.57)	MI, hypertension, diabetes, drug abuse, hyperlipidaemia	Cardiac surgery	Control: placebo<br>Intervention groups: NAC, vitamin C, selenium	Primary outcomes not defined. AKI, mechanical ventilation duration, ICU length of stay, hospital length of stay, in-hospital mortality	Secondary outcomes not defined
Billings 2015[152]	• Italy<br>• English<br>• 2015<br>• 60 Patients<br>• Inclusion Criteria: 18–80 years of age and undergoing elective cardiac surgery requiring CPB.	Exclusion criteria: 1) allergy to acetaminophen, 2) evidence of severe hepatic impairment (history of liver cirrhosis or total bilirubin >2.0mg/dL) 3) evidence of impaired renal function (serum creatinine >2.0mg/dL) or 4) pregnancy.	median (25th, 75th percentile).<br>Acetaminophen: 64.5 (49.0, 68.4)<br>Placebo: 61.0 (54, 67.8)	Hypertension, atrial fibrillation, diabetes, peripheral vascula	Cardiac Surgery	Acetaminophen<br><br>Placebo	oxidative stress response as measured by plasma and urine F2-isoprostanes and isofurans.	Blood loss (chest tube drainage), number of blood products transfused, need for surgical reexploration, mechanical ventilation hours, cardiac enzymes, aspartate aminotransferase (AST), urinary NGAL,

				r disease .				prevalence of postoperative atrial fibrillation and acute kidney injury (AKI) and hospital length of stay
Nouri Majalan 2009 [153]	• Iran<br>• English<br>• 2009<br>• 60 Patients<br>• Inclusion Criteria: included age >18 years, glomerular filtration rate (GFR) < 60 mL/min, and no prior use of allopurinol or vitamin E.	Exclusion Criteria: nonelective emergency surgery, history of dialysis, allergy to vitamin E or allopurinol, and use of radio contrast media within three months prior to surgery.	Treatment group: 65 (9.5) years<br><br>Control group: 61 (7.90) years	Diabetes, Hypertension,	Cardiac Surgery	100 units vitamin E four times per day and 100 mg allopurinol twice daily for three to five days prior to elective surgery, or to no treatment	Primary outcomes not defined	Serum potassium, creatinine, creatinine clearance, acute renal failure, dopamine infusion, length of stay
Wijnen 2002[154]	• Netherlands<br>• English<br>• 2002<br>• 42 Patients<br>• Inclusion Criteria: patients undergoing an elective open infrarenal abdominal aneurysm repair.	Exclusion Criteria: NA	Mean (Range)<br>Intervention: 67 (51-75)<br>Control: 70 (59-82)	NA	Vascular Surgery	Multi-anti-oxidant supplementation (vitamin E 200 mg orally for 5 days before surgery + vitamin C 200 mg orally on morning of surgery + allopurinol 300 mg orally 1 day before surgery and 300 mg at induction + N-acetyl cysteine 150 mg/kg bolus, followed by infusion of 200 mg/kg over 12 hours preoperatively +mannitol 10%, 500 ml over 12 hours from the time of surgery)<br><br>Usual care – no	Primary outcomes not defined	Peri-operative blood loss, Myocardial infarction, post-operative complications

						interventions		
**Miscellaneous Group**								
Amit 2018[155]	• Canada<br>• English<br>• 2018<br>• 606 Patients<br>• Inclusion Criteria: eligible patents were scheduled for elective repair of an abdominal aortic aneurysm, planned as either an open or an endovascular procedure. Patients scheduled for an endovascular procedure had to have at least 1 preoperative risk factor for surgical complications (diabetes mellitus, older than 70 years of age or elevated serum creatinine concentration, defined by value > 177 µmol.L-1 in men or > 146 µmol.L-1 in women).	Exclusion criteria: Elective abdominal AAA repair expected to occur in ≤ 3 days<br>Prior kidney transplant<br>Pregnant or breastfeeding<br>Current active gastrointestinal reflux disease, gastrointestinal ulcer, or hepatobiliary disease<br>Evidence of acute kidney injury in prior 30 days<br>Participating in another study that could conflict with the intervention or outcomes of this trial<br>Received ≥ 1 dialysis treatment (hemodialysis or peritoneal dialysis) in past week<br>Previous participation in this trial<br>A history of a major bleeding event in the prior 6 months<br>A bleeding disorder: a diagnosis of hemophilia, von Willibrand disease, platelets <70<br>An allergy to turmeric, ginger, curry, cumin, cardamom, yellow or red food coloring, gelatin or cellulose<br>A history of hypoglycemia in the past 6 months (<3.5 mmol/L or < 135.0 mg/dL)	Median (IQR)<br>Curcumin: 76 (71 to 80)<br>Placebo: 76 (70 to 81)	AAA, Diabetes, Congestive heart failure, Hypertension, Coronary artery disease, stroke, TIA.	Vascular Surgery	Oral curcumin (2000-mg doses 8 times over 4 d).	median concentrations of 4 biomarkers indicating injury and inflammation (postoperative urine interleukin-18 and perioperative rise in serum creatinine, plasma N-terminal pro–B-type natriuretic peptide and plasma high-sensitivity C-reactive protein).	Acute kidney injury, length of hospital stay, risk of clinical events

Balaguer 2013 a[156]	• USA<br>• English<br>• 2013<br>• 115 Patients<br>• Adult patients undergoing primary elective on-pump cardiac surgery including CABG or valvular surgery.	Exclusion Criteria: impaired renal function (creatinine >1.6 mg/dl), anemia (hematocrit <30%), evidence of coagulopathy (international normalized ratio >1.7), platelet count of <100 × 10^9 ml−1, and taking a glycoprotein IIb/IIIa antagonist within 48 h of surgery.	HOE 140 (bradykinin B2 receptor antagonist): 61.0 (2.0)<br><br>Aminocaproic acid: 58.5 (2.0)<br><br>Placebo: 60.1 (1.8)	Hypertension, Atrial fibrillation, Diabetes	Cardiac Surgery	HOE 140 (bradykinin B2 receptor antagonist)<br><br>Aminocaproic acid<br><br>Placebo	occurrence (yes or no) of any blood product transfusion	proportion as well as the number of blood product units transfused, blood loss as measured by 24-h chest tube output, re-exploration for bleeding, intraoperative MAP, prolonged ventilation (>24 h), new-onset postoperative atrial fibrillation, placement of permanent pacemaker, AKI, length of hospital stay, and 30-day readmission rate.
Balaguer 2013 b[156]	• USA<br>• English<br>• 2013<br>• 115 Patients<br>• Adult patients undergoing primary elective on-pump cardiac surgery including CABG or valvular surgery.	Exclusion Criteria: impaired renal function (creatinine >1.6 mg.dl-1), anemia (hematocrit <30%), evidence of coagulopathy (international normalized ratio >1.7), platelet count of <100 × 109 ml−1, and taking a glycoprotein IIb/IIIa antagonist within 48 h of surgery.	HOE 140 (bradykinin B2 receptor antagonist): 61.0 (2.0)<br><br>Aminocaproic acid: 58.5 (2.0)<br><br>Placebo: 60.1 (1.8)	Hypertension, Atrial fibrillation, Diabetes	Cardiac Surgery	HOE 140 (bradykinin B2 receptor antagonist)<br><br>Aminocaproic acid<br><br>Placebo	occurrence (yes or no) of any blood product transfusion	proportion as well as the number of blood product units transfused, blood loss as measured by 24-h chest tube output, re-exploration for bleeding, intraoperative MAP, prolonged ventilation (>24 h), new-onset postoperative atrial fibrillation, placement of permanent pacemaker, AKI, length of hospital stay, and 30-day readmission rate.
Billings 2012 b[1]	• USA<br>• English<br>• 2012<br>• 87 Patients<br>• Adult patients undergoing cardiac surgery with the use	left ventricular ejection fraction less than 30%, serum potassium greater than 5.0 mEq.L-1, serum creatinine greater than 1.6 mg.dL-1, and inability to discontinue current ACE inhibitor or ARB.	Candesartan: 67.0(1.7)<br>Ramipril: 64.4(2.1)<br>Placebo: 66.1(2.1)	CV disease Hypertension	Cardiac Surgery	Candesartan<br>Ramipril<br>Placebo	plasma t-PA and PAI-1 antigen concentrations	- IL-6, IL-8 and IL-10 concentrations.<br>- postoperative blood loss, transfusion requirements, re-exploration for bleeding, inotropic and

	of CPB							vasopressor use, new onset atrial fibrillation, and changes in serum creatinine
Blogowski 2012[157]	• Poland<br>• English<br>• 2012<br>• 68 Patients<br>• Adult patients undergoing abdominal aortic aneurysm repair	Exclusion Criteria: NA	Intervention: 66.3 (8.29)<br>Control: 68.95 (4.94)	AAA	Vascular Surgery	Prostaglandin E<br>Control	Superoxide dismutase, catalase, glutathione, glutathione peroxidase (GPx), and glutathione transferase (GST) levels	Urine Output, Hospital length of stay
Choi 2013[158]	• Korea<br>• English<br>• 2013<br>• 100 patients<br>• Inclusion criteria: patients(aged20-75years) who were scheduled for elective, isolated, multivessel OPCAB.	Exclusion Criteria: Patients with previous thyroid disease, abnormal baseline thyroid function, preoperative atrial fibrillation, previous cerebrovascular incident, left ventricular ejection fraction o35%, acute myocardial infarction (MI)within7days, mitral regurgitationgrademorethan2/4, chronic obstructive pulmonary disease, history of cardiac surgery, coronary angiography within24 hours before surgery, amiodarone medication, and serum creatinine (sCr) level Z1.5 mg.dL-1 were excluded	T3: 63 (9)<br>Placebo: 65 (8)	Daiabetes, Hypertension	Cardiac Surgery	receivedeither20 lg of oral triiodothyronine (T3) or placebo every12hours	Primary end point was to compare the perioperative thyroid hormone concentrations between the groups	Compare the postoperative CI and clinical outcome between the groups.
Dastan 2018[159]	• Iran<br>• English<br>• 2018<br>• 134 patients<br>• Inclusion criteria: Patients	Exclusion Criteria: history of any preoperative supraventricular arrhythmias, concomitant valve surgery, history of the use of antiarrhythmic drugs	L-Carnitine Group: 60.0 (9.2)<br><br>Control Group: 59.9 (8.0)	Diabetes, Hypertension, CVA, Myocar	Cardiac Surgery	L-Carnitine Group and Control Group	C-Reactive Protein	Renal function, Post-op Complications

	scheduled to scheduled to undergo CABG	except for beta-blockers and calcium-channel blockers, history of seizure or epilepsy, history of hypersensitivity to L-carnitine, chronic liver insufficiency (liver enzyme levels > 3 times the upper limit of normal), chronic kidney disease (stages IV and V), history of the use of anti-inflammatory medications except for aspirin for at least 2 weeks before admission, hypothyroidism, and consumption of magnesium before CABG.		dial infarction				
Demirjian 2017[160]	• USA<br>• English<br>• 2017<br>• 16 patients<br>• Inclusion criteria: nonemergent, on-pump cardiac surgery	Exclusion Criteria: postoperative Cleveland Clinic Foundation AKI cumulative risk score was >8, they had received an organ transplant, they were receiving immunosuppressive therapy, or they were women of childbearing potential.	QPI-1002(siRNA): 61.5 (10)<br>Placebo: 63 (20)	NA	Cardiac Surgery	QPI-1002(siRNA)<br><br>Placebo	Primary Outcomes not defined	Serum Creatinine, C3a, c5a, c4a, Complement Bb, Adverse events
Ederoth 2018[161]	• Sweden<br>• English<br>• 2018<br>• 224 patients<br>• Inclusion criteria: The study patient is scheduled for non-emergent (decision to operate more than 1 hour before start of surgery) CABG surgery. Preoperative cystatin C estimated glomerular	Exclusion Criteria: The patient has an uncontrolled hypertension.<br><br>Hypersensitivity to the active drug or vehicle, including egg protein, soya protein or peanut protein.<br><br>The patient is pregnant or is a fertile woman.<br><br>The patient has been treated	Placebo: 69 (8)<br>Cyclosporine: 69 (8)	Hypertension, Congestive Heart Failure, COPD, Diabetes, Peripheral vascular	Cardiac Surgery	Placebo<br><br>Cyclosporine	plasma cystatin C	plasma creatinine, troponin T, creatinine kinase-MB, or S100B, Risk of AKI, eGFR

	filtration rate (eGFR) or the Modification of Diet for Renal Disease (MDRD) eGFR is 15–90 mL.min-1 per 1.73m-2.	with ciclosporin within 4 weeks prior to the surgery.<br><br>The patient has a known ongoing malignancy.<br><br>The patient has ongoing immunosuppressive treatment.<br><br>The patient has severe hepatic dysfunction.<br><br>The patient is treated with dialysis.<br><br>The patient has preoperatively ongoing and/or increasing clinical infection with C reactive protein (CRP) levels of >50 mg/L. Clinical signs of infection may or may not be present. Increase in CRP due to signs of cardiac origin,41 according to the investigator, should not be considered as exclusion criteria.<br><br>The patient has a severe ongoing viral infection, including HIV, hepatitis C, current or history of hepatitis B.<br><br>For non-allowed and restricted ongoing and concomitant medications, see Protocol section 12.2.<br><br>The patient is planned for off-pump CABG surgery.<br><br>The patient is included in other ongoing clinical trials.		disease<br>,<br>Thyroid disease<br>, Atrial fibrillati<br>on					

Ellenberger 2018[162]	• Switzerland<br>• English<br>• 2018<br>• 224 patients<br>• Inclusion criteria: patients with severe aortic valve stenosis and/or coronary artery disease scheduled for elective aortic valve replacement (AVR) and/or coronary artery bypass surgery (CABG) were all screened and included if they had a Bernstein–Parsonnet score >7.	Exclusion Criteria: emergent or off-pump surgery, preoperative critical conditions, poorly controlled diabetes mellitus (glucose >12 or <3 mmol·L−1 ≥3 episodes per week), severe liver disease (Child– Pugh C stage), and dementia or significant cerebrovascular disease	GIK: 70.9 (10.7)<br>Placebo: 71.6 (10.7)	Aortic stenosis, coronary heart disease, hypertension, Diabetes, vascular disease, COPD, Hypercholestrolemia.	Cardiac Surgery	GIK (20 IU of insulin, 10 mEq of potassium chloride in 50 mL of glucose 40%)<br><br>saline infusion given	Post-cardiotomy ventricular dysfunction (PCVD),	Mortality, Cardiovascular complications, atrial fibrillation, respiratory complications, Troponin, Creatinine kinase, Creatinine kinase MB, ICU length of stay, Hospital Length of Stay.
Fayfman 2018[163]	• USA<br>• English<br>• 2018<br>• 80 patients<br>• Inclusion criteria: ages of 18 and 80 years old, had no prior history of DM [based on ICD-10 diagnoses and confirmed by hemoglobin A1c (HbA1c) of b6.5%] and without preoperative hyperglycemia (fasting BG b126 mg.dL-1 or random BG ≤140 mg.dL-1).	Exclusion Criteria: patients expected to require postoperative intensive care unit (ICU) admission, or those planned to be kept on strict NPO following surgery (unable to take study medication. Only subjects requiring general anesthesia for their surgery were enrolled in the study. Patients undergoing cardiac surgery, and/or those with severely impaired renal function (GFR b30 mL.min per 1.73 m2), clinically significant hepatic failure, pancreatic, or gallbladder disease, surgery for gastrointestinal obstruction, ileus or potential need for gastric suction, pre-operative treatment with glucocorticoids (equivalent to prednisone N5 mg/day), pregnancy or inability to	Sitagliptin: 51.1 (12.8)<br>Placebo: 45.9 (14.5)	NA	Mixed Surgery	Sitagliptin<br><br>Placebo	stress hyperglycemia, defined by blood glucose (BG) >140 mg/dL and >180 mg/dL after surgery	length-of-stay, ICU transfers, hypoglycemia, and hospital complications.

		consent for any reason.						
Golestaneh 2015[164]	• USA<br>• English<br>• 2015<br>• 40 patients<br>• Inclusion criteria: patients undergoing bypass surgery	Exclusion Criteria: NA	Minocycline: 64.1 (9.9)<br>Placebo: 60.8 (11.5)	Diabetes, COPD, Peripheral Vascular Disease, Chronic Kidney Disease, Hypertension, Congestive heart failure,	Cardiac Surgery	Minocycline<br>Placebo Capsules	Risk of AKI	death up to 30 days post-op, cardiovascular Events, infections, and length of stay and hemodynamic parameters (cardiac index, systemic vascular resistance, pulmonary artery diastolic and central venous pressures)
Himmelfarb 2018[165]	• USA<br>• English<br>• 2018<br>• 452 patients<br>• Inclusion criteria: men and women between 18 and 85 years of age who were scheduled for nonemergent cardiac surgery requiring cardiopulmonary bypass with additional recognized risk factors for AKI as determined by one or more of the following parameters: Egfr<60 ml.min-1 per 1.73 m2, age ≥75 years old, higher-risk surgery type (e.g.,	Exclusion criteria: had AKI at the time of screening, eGFR,20 ml.min-1 per 1.73 m2, prior organ transplantation, or dialysis dependence	Intervention: 74.0(11.1)<br>Control: 71.5(9.6)	CKD, AKI, Hypertension, Heart Failure, Ischemic heart disease, Cardiac arrythmias, diabetes	Cardiac Surgery	THR-184<br><br>0.9% sodium chloride	Development of AKI within 7 d of surgery according to KDIGO criteria	Development of AKI within 7 d of surgery according to serum creatinine–based KDIGO criteria<br>Severity of AKI per KDIGO criteria<br>Duration of AKI within the first 7 d<br>Death, dialysis, or eGFR decline .30% at day 30

	heart valve plus CABG), New York Heart Association class 3 or 4, left ventricular ejection fraction≤35%, history of diabetes mellitus with insulin use or proteinuria, or hemoglobin ,10 mg/dl							
Kishimoto 2018[166]	• Japan<br>• English<br>• 2002<br>• 280 patients<br>• Inclusion criteria: Adult patients who were undergoing cardiac surgery with cardiopulmonary bypass	Exclusion Criteria: (1) under dialysis treatment preoperatively; (2) presence of renal dysfunction (serum creatinine concentration ≥3.0 mg.dL-1); and (3) unable to take oral medication on postoperative day (POD)	tolvaptan group: 70.8(11.4) Conventional diuretic group (furosemide (20–40 mg/day): 69.5(12.2)	Coronary artery disease, Diabetes, Hyperlipidaemia, COPD, Cerebral infarction, Hypertension	Cardiac surgery	tolvaptan group<br><br>Conventional diuretic group (furosemide (20–40 mg/day)	incidence of worsening renal function (WRF)	Body fluid management: urine output, the time required to restore preoperative body weight, the total amount of furosemide administered, maximum serum creatinine concentrations, rate of increase in creatinine concentrations, changes in the serum sodium and potassium concentrations
Kramer 2002 [167]	• Germany<br>• English<br>• 2002<br>• 56 patients<br>• Inclusion criteria: Patients undergoing CABG	Exclusion criteria: allergic reaction to theophylline, asthma bronchiale related with theophylline, heart failure (ejection fraction <50%, baseline serum creatinine>1.3 mg/dl and impaired liver function	Theophylline (bolus of 4mg/kg followed by continuous infusion of 0.25mg.kg-1.h-1): 60.4(10.1) Isotonic saline placebo: 60.3(8.1)	Coronary artery disease	Cardiac Surgery	Theophylline (bolus of 4mg.kg-1 followed by continuous infusion of 0.25mg.kg-1.h-1)<br><br>Isotonic saline placebo	Serum creatinine and GFR	Fluid intake and output, Bloop pressure and heart rate, plasma theophylline concentrations,
Mansourian 2015[168]	• Germany<br>• English<br>• 2002<br>• 34 patients<br>• Inclusion criteria: isolated CABG candidates with EF	Exclusion Criteria: previous renal failure (RF), recent myocardial infarction (MI) (less than 4 weeks ago), uncontrolled diabetes, and use of anti-inflammatory drugs	median (interquartile range) oral pentoxifylline: 59.0 (53.0 to 69.0) Placebo Controlled: 59.0 (53.0 to 69.0)	NA	Cardiac Surgery	oral pentoxifylline Placebo Controlled	Primary outcomes not defined	Trop-T (ng/mL), TNF-a (pg/mL), IL-6 (pg/mL), WBC (×10/cm), Hgb (mg/dL), Hct (%), BUN (mg/dL), Creatinine, CPB Time, Length of stay, Post op Complications,

	≤30%.							Mortality
Morgera 2002[169]	• Germany<br>• English<br>• 2002<br>• 34 patients<br>• Inclusion criteria: normal renal function before surgery and a cardiac ejection fraction <40%.	Exclusion criteria: concomitant valve or left ventricular reducing operations (Batista operation) as well as cardiac redo operations	Prostacyclin: 62 (5.5)<br>Control: 61 (7.0)	Hypertension, Myocardial infarction, Diabetes, Peripheral vascular disease, coronary artery disease	Cardiac surgery	Prostacyclin<br>Control	Creatinine clearance, Urine output	MAP, mean arterial pressure; CVP, central venous pressure; Hct, Hematocrit; Fractional excretion rate of sodium (FENa,) and N-acetyl--D-glucosaminidase (B-NAG)
Schmidt 2017[170]	• Switzerland<br>• English<br>• 2017<br>• 411 patients<br>• Inclusion criteria: Patients >18 years of age and scheduled for elective coronary artery bypass graft (CABG) and/or valve surgery were	Exclusion Criteria: emergency operations, pregnancy, chronic renal failure, signs of an acute infection, age <18 years, failure to obtain informed consent, or participation in another clinical trial.	Selenium: 66 (11)<br>Placebo: 68 (10)	Hypertension, Coronary artery disease, Atrial fibrillation, COPD, Diabetes,	Cardiac Surgery	4000 mg selenium (in the form of sodium selenite) or placebo	SOFA score on study day 3	SOFA score sub-variables, inflammatory markers, cardiac markers
Shahbazi 2017[171]	• Iran<br>• English<br>• 2017<br>• 144 patients<br>• Inclusion criteria: Patients undergoing cardiac surgery without arrhythmia, tachycardia, non-oliguric kidney injury,	Exclusion Criteria: NA	Aminophylline: 61.3(10.09)<br>Placebo: 65.6(10.10)	NA	Cardiac Surgery	5 mg/kg aminophylline bolus<br><br>normal saline Placebo	glomerular filtration rate (GFR) and Cr and its relationship with the Cleveland score	severe acute kidney injury, time between intensive care unit (ICU) admission and first successful extubation, CPB time, blood transfusion volume, urine output(UO), ICU and

	anuria, obstructive, uropathy, and congenital anomalies							post intensive care unit (PICU), and length of hospital stay.
Soliman 2019[172]	• Saudi Arabia<br>• English<br>• 2019<br>• 122 patients<br>• Inclusion criteria: adult patients with diabetes mellitus, ejection fraction >40%).	Exclusion Criteria: patients with congestive heart failure, acute myocardial infarction, emergency, redo cases, malfunctioning artificial heart valve, obstructive cardiomyopathy, heart rate <50 bpm, pericardial disease, and renal or hepatic impairment.	Magnesium Sulphate: 55.70(12.62)<br>Placebo: 56.15(13.25)	Diabetes, Hypertension, Ischemic heart disease, valvular disease, atrial fibrillation	Cardiac Surgery	Magnesium sulphate (15 mg.kg-1.h-1)<br><br>Equal volume of placebo	efficacy of magnesium infusion in reducing the blood glucose levels.	requirement for insulin infusion in addition to the safety of the study medication, which was assessed by the occurrence of any adverse events.
Song 2013 1[173]	• Korea<br>• English<br>• 2013<br>• 23 patients<br>• Inclusion Criteria: patients undergoing elective cardiac surgery requiring CPB	Exclusion criteria: urgent/ emergency surgery, previous heart surgery, diabetes, ischemic heart disease, combined surgery with a coronary artery bypass graft procedure, age >75 years, left ventricular ejection fraction <45%, diabetes, active gastropathic disorder, chronic obstructive pulmonary disease, preoperative administration of furosemide, and renal failure requiring renal replacement therapy	control (group C): 59 (15)<br>Ulinastatin pretreatment (group U) - 5,000 U/kg: 58 (17)	NA	Cardiac Surgery	control (group C)<br>Ulinastatin pretreatment (group U) - 5,000 U/kg	PaO2/FiO2 ratio	CK-MB (ng/ml, TnI (ng/ml) Serum creatinine (mg/dl)<br>GFR (ml.min -1 per 1.73 m2)<br>Creatinine clearance (ml/min)<br>Extubation time (min)<br>Urine output in the first 2 h in the ICU (ml)<br>Bleeding during the first 2 h in the ICU (chest tube, ml)
Stoppe 2013[174]	• Belgium<br>• English<br>• 2013	Exclusion criteria: women with childbearing potential or pregnancy, cardiac, respiratory, liver, or	median (range)<br>Sevoflurane: 68 (51–79)<br>Xenon: 66 (48–81)	Acute kidney injury, Hyperb	Cardiac Surgery	Sevoflurane<br>xenon	Adverse events (AE)	feasibility and safety criteria (evaluation of anaesthetic depth,

	• 30 patients<br>• Inclusion Criteria: isolated CABG surgery with the use of CPB, ASA physical status II–IV, preserved cardiac function (left ventricular ejection fraction .50%), and EuroSCORE ≤8.	renal failure (creatinine .1.5 mg dl), acute coronary syndrome within 24 h before surgery, haemodynamic instability, emergency operations, lack of informed consent, severe neurological dysfunction, depression, a geriatric depression score (GDS) .5, and Mini-Mental State Examination (MMSE) <24. Furthermore, patients with predisposition to malignant hyperthermia and/or hypersensitivity to the study drugs		ilirubin aemia, Hypote nsion, Throm bocyto penia				haemodynamic and respiratory profile, regional cerebral tissue oxygenation), assessment of organ dysfunction, time on mechanical ventilation, postoperative inflammation, perioperative values of TropT and NT-Pro-BNP, ICU and hospital length of stay, and incidence of SIRS, sepsis, severe sepsis, and septic shock.
Thompson 1986[175]	• London<br>• English<br>• 1986<br>• 40 patients<br>• Inclusion Criteria: obstructive jaundice (bilirubin > 100 pmol.l-1) undergoing surgery	Exclusion Criteria: Patients who had undergone surgery within 4 weeks or had duodenal obstruction	Median (Range)<br><br>Oral UDCA (Destolit, Merrell Pharmaceuticals Ltd): 57.0 (18-72)<br><br>no additional treatment (control patients): 56.5 (45-78)	Obstru ctive Jaundic e	HPB Surgery	Oral UDCA (Destolit, Merrell Pharmaceuticals Ltd)900 mg 8-hourly for 48 h immediately preoperatively<br><br>no additional treatment (control patients)	Primary outcomes not defined	Bilirubin (umol.l-1), Alkaline phosphatase, Albumin, Haematocrit, White cell count, Creatinine.
Wang 2013[176]	• China<br>• English<br>• 2014<br>• 44 patients<br>• Inclusion Criteria: ASA physical status I/II/III adult patients were enrolled in the current study when they were scheduled for hepatectomy with inflow occlusion.	Exclusion criteria: (i) age <18 or >70 yearsr, (ii) operation history of cryosurgery or radiofrequency ablation, (iii) scheduled resection not requiring hepatic portal occlusion, (iv) cardiac ejection fraction ,40%, myocardial infarction within 3 months, or any angina pain within 48 h, (v) pulmonary dysfunction (PaO2,60 mm Hg) or chronic obstructive pulmonary disease, (vi) hypertension,	range<br>Dexmedetomidine: 27–68<br>saline placebo: 30–69	Conges tive heart failure, Myocar dial infarcti on, Renal failure, Death, HPB Malign ancy	HPB Surgery	Dexmedetomidine<br>saline placebo	serum diamine oxidase (DAO) activity	variables reflecting intestinal, hepatic, kidney, and cardiopulmonary function, and biomarkers of oxidative stress and systemic inflammatory response

		(vii) diabetes mellitus, and (viii) history of inflammatory bowel disease or diarrhoea (≥ 2 liquid stools per day for ≥2 days) within 1 week of surgery.						
Yu 2018[177]	• China<br>• English<br>• 2018<br>• 80 patients<br>• Inclusion Criteria: Patients who met the diagnostic criteria for abdominal aortic dissection; patients who were classified into American Society of Anesthesiologists (ASA) Grade II-III; patients aged 54-75 years old.	Exclusion Criteria: Patients with severe mental diseases; patients with severe cardiovascular and cerebrovascular diseases; patients with liver or kidney diseases; patients who were forbidden to receive general anesthesia or local anesthesia.	Group A: General Anesthesia: 65.3(6.2)<br><br>Group B: Local Anesthesia: 66.5(7.3)	Hypertension, Diabetes, Hyperlipidemia, Coronary artery disease, AAA.	Vascular Surgery	Group A received the interventional surgery for abdominal aortic dissection under general anesthesia and those in Group B received the interventional surgery under local anesthesia.	Primary outcomes not defined	The duration of the surgery, intraoperative bleeding volume, intra-operative urine amount, adverse reactions of anesthesia, blood pressure and heart rate right at the beginning of the surgery (T0), before stent release (T1), during stent release (T2) and at the end of surgery (T3) of the two groups of patients were recorded. Hospitalization duration and hospitalization cost.
Demir 2015[178]	• Turkey<br>• English<br>• 2015<br>• 37 patients<br>• Inclusion Criteria: patients scheduled for elective CABG with cardiopulmonary bypass	Exclusion criteria: patients undergoing emergency operation, combined operation, off-pump surgery, repeat surgery or valve surgery, patients presenting with chronic kidney disease or renal impairment, patients younger than 18 years old and neurological and/or psychiatric disturbances, patients who underwent angiography more than a week shortly before	Remifentanil (RPM) group: 61(6.9)<br>Ketamine (KPM) group: 59(8.7)	Diabetes, Hypertension, COPD.	Cardiac Surgery	Remifentanil (RPM) group<br>Ketamine (KPM) group	Primary outcomes not defined	Urea levels (mg.dL-1)<br><br>Creatinine levels (mg.dL-1)<br><br>Cystatin-C levels (mg. L-1)<br><br>Hs-troponin T levels (ng/L)
**Nitric Oxide Donor**								

Kaya 2007[179]	• Turkey<br>• English<br>• 2007<br>• 240 patients<br>• Inclusion Criteria: patients with multi-vessel coronary artery disease undergoing elective, primary CABG. (1) left ventricular ejection fraction (LVEF) 0.50; (2) estimated glomerular filtration rate (eGFR) >30ml.min-1; and (3) with at least two de novo lesions located in different major epicardial coronary arteries amenable to bypass surgery.	Exclusion Criteria: (1) signs of congestive heart failure class IV, or cardiogenic shock; (2) unstable angina pectoris; (3) myocardial infarction (MI) within the week preceding randomization, (4) dialysis dependent renal failure, (5) microalbuminuria, (6) hepatic dysfunction; (7) sickle cell anemia, (8) serum creatinine (SCr) level >3 mg/dl pre-operatively; (9) morbid obesity or cachexia; (10) skeletal muscle disorders or paraplegia and (11) concomitant major cardiac procedures.	Saline: 61.3 (9.7)<br>Sodium Nitroprusside: 60.8 (10.8)	Diabetes, Hypertension, Pulmonary disease, Peripheral vascular disease	Cardiac Surgery	Saline (NaCl) or SNP (Nipruss, Adeka, Turkey) infusion from initiation of rewarming period during CPB until weaning from CPB	Serum Creatinine, Estimated GFR	changes in cardiac enzymes, urine output per hour, daily fluid balance, changes in creatinine clearance over time, and major adverse events.
Lei 2018[180]	• China<br>• English<br>• 2018<br>• 244 patients<br>• Inclusion Criteria: adult patients undergoing multiple valve cardiac surgery	Exclusion criteria: patients who received hydroxyethyl starch in the cardiopulmonary bypass priming solution	nitric oxide (treatment): 48.7(9.5)<br>nitrogen (control): 48.4(8.6)	Rheumatic disease, Congenital valve disease, Infectious valve disease	Cardiac Surgery	nitric oxide (treatment)<br>nitrogen (control)	AKI within 7 days of surgery	Plasma Biomarkers of Hemolysis<br><br>Urinary creatinine<br><br>MAKE index<br><br>Urinary kidney injury molecule-1 to urinary creatinine.<br><br>ratio of urinary neutrophil gelatinase-associated lipocalin to urinary creatinine (NGALu/creau)
**Osmotic Diuretics**								

Carcoana 2003 a[54]	• USA<br>• English<br>• 2003<br>• 100 patients<br>• Inclusion Criteria: Male and non-pregnant female patients aged 21 to 79 years, with a preoperative serum creatinine level of ≤ 1.5 mg.dL-1, who were scheduled for elective, primary coronary artery bypass graft surgery requiring CPB,	Exclusion Criteria: Patients who had cardiac catheterization within 5 days of surgery; preoperative hypotension, defined as a systolic blood pressure <90 mm Hg, at any time; use of an intra-aortic balloon pump at any time during the current hospitalisation; the administration of dopaminergic or antidopaminergic drugs; a contraindication to the use of DA; and chronic inflammatory disease states, lymphoproliferative disorders, or carcinoma, which are associated with increased serum levels of 2M. Because of the diurnal variation in 2M excretion, patients undergoing surgery in the late afternoon or evening were also excluded.	Placebo: 63.3 (8.8)<br>Mannitol: 64.3 (8.9)<br>Dopamine: 63.8 (9.4)<br>Mannitol and dopamine: 63.4 (7.8)	Hypertension, Myocardial infarction, Pulmonary disease ,Cerebrovascular disease , NIDDM ,IDDM.	Cardiac Surgery	Placebo<br>Mannitol<br>Dopamine<br>Mannitol and dopamine	Beta-2 M excretion rate at one/hour post-CPB	Beta-2 M excretion rate at 6 and 24 h post-CPB; urinary flow rate and creatinine clearance at 1, 6, and 24 h post-CPB; and the highest postoperative serum creatinine level. Length of intensive care stay and hospitalization, as well as adverse events.
Dural 2000 b[60]	• Turkey<br>• English<br>• 2000<br>• 36 patients<br>• Inclusion Criteria: elective coronary artery surgery	Exclusion Criteria: (i) serum creatinine levels greater than 1.3 mg.dl-1, (ii) BUN greater than 60 mg.dl-1, (iii) severe hypertension, (iv) presence of any carotid or peripheral artery disease, (v) left ventricular ejection fraction lower than 50%, and (vi) contact with any radiocontrast agent (as in a diagnostic procedure) within 72 hours of surgical procedure.	Control: 53.7 (8.3)<br>Mannitol: 55.4(8.4)<br>Dopamine: 53.2 (10.9)	NA	Cardiac Surgery	Patients received a continuous infusion of dopamine, 3 µg.kg-1.min-1 (Group I), mannitol, 1 Mg.kg-1.h-1 (Group II), no medication (Group III) before the induction of anaesthesia.	(Primary outcomes not defined)	Serum BUN (mg.dl-1) and creatinine (CRE), Urine output (mg.kg-1.h-1), urine log (NAG) activity

Gubern 1988[181]	• Spain<br>• English<br>• 1996<br>• 31 patients<br>• Inclusion Criteria: patients with obstructive jaundice (bilirubin, 3 mg.dl-1 or higher)	Exclusion Criteria: NA	Mannitol: NA<br>Usual Care: NA		Vascular surgery	Mannitol<br><br>Usual care	Primary outcomes not defined	Post-operative complications, creatinine clearance, fibrin degradation products, serum sodium, urinary sodium,
Nicholson 1996[182]	• UK<br>• English<br>• 1996<br>• 28 patients<br>• Inclusion Criteria: patients undergoing elective aortic aneurysm repair	Exclusion Criteria: NA	Mannitol: 68 (65-72)<br>Placebo (Saline): 71 (68-75)<br>Mean 95% CI	AAA, Hypertension, COPD, Diabetes, Thromboembolic disease	Vascular surgery	IV Mannitol 0.3 g.kg-1<br><br>Equivalent volume of IV normal saline	Primary outcomes not defined	Post-operative complications, Mean urine output, blood urea, serum creatinine, creatinine clearance, urinary albumin creatinine ratio. urinary N-acetyl glucosaminidase : creatinine ratio
Raghava 2015[183]	• India<br>• English<br>• 2015<br>• 50 patients<br>• Inclusion Criteria: aged >18, Glasgow Coma Scale (GCS) >13 with ASA physical status 1,2, and 3 scheduled to undergo craniotomy for supratentorial tumors	Exclusion Criteria: Patients with the presence of raised ICP, electrolyte imbalance, with severe cardiac, respiratory, or renal disease	3% hypertonic saline: 41.6(12.9)<br>20% mannitol: 38.8(11.9)	Neuro - Malignancy	Neuro surgery	3% hypertonic saline<br>20% mannitol	Primary outcomes not defined	Hemodynamic variables (heart rate [HR], SBP, DBP, MBP, and central venous pressure [CVP]), serum electrolytes, serum osmolality, urine output, and fluid balance were measured. Surgeon assessed the brain condition on four point scale (1 = perfectly relaxed,<br>2 = satisfactorily relaxed, 3 = firm brain, and 4 = bulging brain),
Shim 2007[184]	• Korea<br>• English<br>• 2007<br>• 50 patients<br>• Inclusion Criteria: patients scheduled for elective	Exclusion Criteria: Patients with pre-existing pulmonary and/or renal disease, serum creatinine level greater than 1.3 mg.dL-1, left ventricular ejection fraction	Control group: 63 (8)<br>Mannitol group: 63 (8)	Diabetes, Hypertension	Cardiac Surgery	Control group<br>Mannitol group	Primary Outcomes not defined	Pulmonary variables (Dynamic lung compliance, Static lung compliance) and CK-MB level. Fluid balance and serum sodium

	multivessel OPCAB	less than<br>40%, unstable angina, and recent myocardial infarction within 1<br>month						level. Hemodynamic data (HR, heart rate; MAP, mean arterial pressure; MPAP, mean pulmonary arterial pressure; CVP, central venous pressure; PCWP, pulmonary capillary wedge pressure; SvO2, mixed venous oxygen saturation; CI, cardiac index.)
**Other Diuretics**								
Barba Navarro 2017[185]	• Mexico<br>• English<br>• 2017<br>• 233 patients<br>• Inclusion Criteria: Adult patients (aged ≥18 years) were eligible for the study if they were undergoing elective or emergency cardiac surgery requiring CPB, for whom the surgery was not an isolated correction of atrial septal defect.	Exclusion criteria: preoperative CKD (defined as creatinine . 1.6 mg.dL-1), kidney transplantation or dialysis, pregnancy, hyperkalemia (potassium. >5.0 mEq.L-1),	Spironolactone: 52.4 (14.3)<br>Placebo: 54.0 (615.8)	Hypertension, diabetes, COPD, Peripheral vascualr disease, cerebrovascular disease, myocardial infarction.	Cardiac Surgery	Spironolactone<br>Placebo	AKI incidence defined by KDIGO	requirement of renal replacement therapy, ICU length of stay, and ICU mortality
Pretorius 2012 b[5]	• USA<br>• English<br>• 2012<br>• 14 patients<br>• Inclusion Criteria: Patients undergoing elective cardiac surgery including coronary	Exclusion Criteria: exclusion criteria were chronic AF or paroxysmal AF within 6 months, an ejection fraction less than 30 per cent, evidence of coagulopathy, emergency	Placebo: 60.0(12.0)<br>Ramipril: 58.7(12.3)<br>Spironolactone: 59.2(12.3)	Hypertension, Diabetes,	Cardiac Surgery	Placebo<br><br>ramipril (2.5 mg the first three days followed by 5mg.day-1, with	occurrence of electrocardiographically confirmed postoperative atrial fibrillation	acute renal failure, hyperkalemia, the incidence of hypotension, length of hospital stay, stroke, and death.

	artery bypass graft or valvular surgery	surgery, serum creatinine greater than 1.6 mg.dl-1, and hyperkalemia with potassium greater than 5.0 mEq.L-1.				the dose reduced to 2.5mg.day-1 on the first postoperative day only),<br><br>spironolactone (25 mg/day).		
**Restrictive vs Liberal**								
Nan Hai 2013[186]	• Country: NA<br>• English<br>• 2013<br>• 174 patients<br>• Inclusion Criteria: Patients undergoing general surgery	Exclusion Criteria: NA	Restricted fluid therapy: NA<br>Standard fluid Therapy: NA	NA	GI Surgery	Restricted fluid therapy<br>Standard fluid Therapy	perioperative intravenous fluid volume	Post-operative complications.
Belavic 2018 1a[187]	• Croatia<br>• English<br>• 2018<br>• 120 Patients<br>• Inclusion Criteria: American Society of Anesthesiology (ASA) classes I and II who were scheduled for laparoscopic cholecystectomy.	Exclusion Criteria: Patients younger than 18 or older than 75 years of age, those with local and systemic inflammatory or immune diseases, and those with severe functional organ impairment or hematologic or malignant diseases were excluded.	median (25–75 interquartile range).<br>Restrictive group: 50 (40.3–62.5)<br>Low liberal group: 58 (44.5–69)<br>High liberal group: 56.5 (42.3–66.5)	NA	GI Surgery	Restrictive group<br>Low liberal group<br>High liberal group	hemodynamic and laboratory evaluating global hypoperfusion (SAP, systolic blood pressure; DAP, diastolic blood pressure; MAP, mean arterial pressure; HR, heart rate)	duration of surgery and anesthesia, onsumption of crystalloids, volatile anesthetics, and opioids. 1-year morbidity.
Choi 2015 2[188]	• Korea<br>• English<br>• 2015<br>• 78 Patients<br>• Inclusion Criteria: individuals scheduled for elective living-donor right lobectomy	Exclusion criteria: Donors whose remnant liver volume was < 35% of the whole liver volume, as determined by computed tomography volumetry, and those with major medical diseases. donors < 20 years of age or with cardiac arrhythmia from the study. Donors	Median (IQR)<br>High Stroke volume variation group: 28.5 (23.5–33.5)<br>Control group : 26.5 (24.0–34.0)	NA	HPB Surgery	High Stroke volume variation group<br><br>Control group	intraoperative blood loss	intra-operative haemodynamic variables, peri-operative laboratory data and postoperative complications in both groups.

		with history of previous abdominal operation.						
Matot 2012[189]	• Israel<br>• English<br>• 2012<br>• 107 Patients<br>• Inclusion Criteria: adult patients with an American Society of Anesthesiologists. physical status of I through III who were presenting for laparoscopic bariatric surgery. Patients were considered eligible if they had a BMI greater than 40 or had a BMI greater than 35 and at least 1 comorbid condition and were scheduled to undergo one of the following laparoscopic operations: Roux-en-Y gastric bypass, biliopancreatic diversion with duodenal switch, or sleeve gastrectomy.	Exclusion criteria: younger than 18 years, patients with renal dysfunction (creatinine level >50% of the upper limit of the reference range) or congestive heart failure, and patients receiving diuretics were excluded from the study.	high-volume group (HVG)<br>low-volume group (LVG)	Type 2 diabetes, ischemic heart disease, obstructive sleep apnoea, Hyperlipidaemia.	GI Surgery	high-volume group (HVG)<br>low-volume group (LVG)	intraoperative urine output	serum creatinine concentrations in the first 3 postoperative days. number of patients who died and those who developed complications during the perioperative period.
Matot 2013[190]	• Israel<br>• English<br>• 2013<br>• 102 Patients<br>• Inclusion Criteria: adult patients with an American Society of Anesthesiologists (ASA) physical status of 1 through 3 who were undergoing elective VATS	Exclusion criteria: patients younger than 18 years, patients with renal dysfunction (creatinine >50% upper limit of reference range, which is 1.3 mg.dL-1 for men and 1.1 mg.dL-1 for women), and patients with congestive heart failure (previous history of pulmonary edema as defined by clinical and radiologic signs that required a change in medication involving at minimum treatment with	high volume : 64 (10)<br>low volume : 65 (11)	Hyperlipidaemia, Hypernsion, ischamic heart disease, diabetes, oulmonary disease	Thoracic surgery	high (8 mL.kg-1.h-1) volume of Ringer's lactate solution.<br><br>low (2 mL.kg-1.h-1) volume of Ringer's lactate solution.	intraoperative urinary output.	creatinine serum levels and postoperative complication rate.

		diuretic drugs).						
Abraham Nordling 2012[191]	• Sweden<br>• English<br>• 2018<br>• 161 Patients<br>• Inclusion Criteria: elective colorectal resection with or without stoma, adult patients and American Society of Anesthesiologists grade I–III (no life-threatening systemic diseases).	Exclusion Criteria: disseminated or secondary cancer, inflammatory bowel disease, diabetes mellitus, renal insufficiency (serum creatinine level more than 180 µmol.l-1), alcohol overconsumption, pregnancy, lactation, mental disorder and contraindications to epidural analgesia.	median (interquartile range).<br>Restricted fluid: 68·0 (59·0–77·0)<br>Standard fluid: 69·0 (62·0–79·0)	NA	General Surgery	Restricted fluid therapy<br><br>Standard fluid therapy	Length of postoperative hospital stay	complications within 30 days.
Bhaskaran 2018[192]	• India<br>• English<br>• 2018<br>• 600 Patients<br>• Inclusion Criteria: Adult patients of either gender aged between 40 and 70 years with estimated glomerular filtration rate (eGFR) of more than 60 mL/min/1.73 m2, patients with triple vessel coronary heart disease, and the American Society of Anesthesiologist – II - III physical status	Exclusion criteria: serum creatinine higher than 1.20 mg/dl in males and higher than 0.8 in females, emergency surgery, preoperative resting hypoxia (room air oxygen saturation [SpO2] <90%), preoperative mechanical ventilation, body mass index (BMI) <18 or >30, age <40 or >70 years, ejection fraction < 45%, comorbid disease other than hypertension (HTN) and diabetes mellitus (DM), cardiovascular instability requiring cardiac support to maintain hemodynamic parameters within 20% of baseline perioperatively, intraoperative or postoperative respiratory dysfunction leading to partial pressure of carbon dioxide (PaCO2) >45 mmg or requiring noninvasive	Group A: 59.40 (7.017)<br>Group B: 58.18 (6.30)	Hypertension, Diabetes	Cardiac Surgery	Liberal Fluid Therapy – Group A<br><br>Restricted fluid therapy – Group B	Risk of AKI	serum creatinine, mean estimated glomerular filtration rate, mean pH, mean PCO2, mean bicarbonate, mean base excess,<br>mean serum chloride, mean serum sodium, mean serum potassium, mean lactate,

		ventilation, not able to maintaine intraoperative or postoperative haemodynamic parameters within 20% of baseline, intraoperative or postoperative demise of patient due to other than renal function deterioration, preexisting known kidney disease or previous nephrectomy or previous renal transplantation were excluded from the study.						
Gao 2012[193]	• China<br>• English<br>• 2012<br>• 179 Patients<br>• Inclusion Criteria: patients aged 65 years or older who were admitted for gastrointestinal cancer surgeries	Exclusion criteria: life-threatening systemic disease, according to the American society of anesthesiologists (ASA) physical scoring system (ASA scores C4); currently lactating; language problems; smoking within 2 weeks or diagnosis of mental disorder, diabetes mellitus, renal insufficiency, disseminated cancer, secondary cancers, inflammatory bowel disease, or diseases hindering epidural analgesia.	Median (Range)<br>Restricted group: 72 (65–89)<br>Standard group: 73 (65–87)	Cardiovascular disease, pulmonary disease, GI Cancer	General surgery	Restricted group<br>Standard group	Complication recorded within 30 days after surgery	death and other adverse effects, including ischemia and impairment of renal function.
Hubner 2012[194]	• Switzerland<br>• English<br>• 2012<br>• 197 Patients<br>• Inclusion Criteria: patients undergoing elective open colonic surgery	Exclusion criteria: NA	Median(range)<br>Fast track program: 62 (27–91)<br>Standard care: 59 (39–89)	NA	General surgery	Fast track program: fluid restriction and epidural Analgesia<br><br>Standard care	perioperative vasopressor requirements and/or need for additional fluid administration indicating clinically relevant hemodynamic	plasma concentrations of sodium, potassium, creatinine, and hematocrit values pre- and postoperatively that served as surrogate parameters for perioperative fluid shifts

							instability	
Jie 2014[195]	• China<br>• English<br>• 2014<br>• 197 Patients<br>• Inclusion Criteria: Adult patients admitted for elective colorectal resection were considered eligible if they had no life-threatening systemic diseases [American Society of Anesthesiologist (ASA) Ⅰ-Ⅲ]	Exclusion criteria: current lactation, pregnancy, language problems, smoking within 2 wk, diabetes mellitus, renal insufficiency, disseminated or secondary cancer, inflammatory bowel disease, alcohol overconsumption, mental disorder and contraindications to epidural analgesia	Restricted fluid: 64.7 (16.8)<br>Standard fluid: 65.4 (17.6)	Cardiovascular disease, pulmonary disease, colorectal cancer	GI Cancer	Restricted fluid<br>Standard fluid	Any complication recorded within 30 d as well as death	other adverse effects, including ischemia and impairment of renal function after surgery
Mazer 2018[196]	• Canada<br>• English<br>• 2018<br>• 5243 Patients<br>• Inclusion Criteria: adults undergoing cardiac surgery with CPB and who had a preoperative additive EuroSCORE I score of 6 or higher.	Exclusion criteria: patients who were unable to receive blood products, declined blood products, were participating in a preoperative autologous blood donation program, were undergoing heart transplantation, were having surgery solely for the insertion of a ventricular assist device, or were pregnant or lactating.	Restrictive Threshold: 72(10)<br>Liberal Threshold: 72(10)	Diabetes, Hypertension, previous cardiac surgery.	Cardiac surgery	Restrictive Threshold<br>Liberal Threshold	Composite-outcome event	Death from any cause<br>Myocardial infarction<br>Stroke<br>New-onset renal failure with dialysis<br>Expanded secondary composite-outcome event<br>Coronary revascularization<br>Hospital readmission or emergency department visit<br>Hospital readmission<br>Emergency department visit
Myles 2018[197]	• New Zealand<br>• English<br>• 2018<br>• 2983 Patients<br>• Inclusion Criteria: adults who had an increased risk of complications while undergoing major abdominal surgery that included a skin	Exclusion criteria: undergoing urgent or time-critical surgery, liver resection, or less extensive surgery (e.g., laparoscopic cholecystectomy) or if they had end-stage kidney failure requiring dialysis.	Restrictive Fluid: 66(13)<br>Liberal Fluid: 66(13)	Hypertension, coronary artery disease, heart failure, peripheral	GI Surgery	Restrictive Fluid<br>Liberal Fluid	disability-free survival at 1 year	AKI, renal replacement therapy (RRT), Complications, death

	incision, an expected operative duration of at least 2 hours, and an expected hospital stay of at least 3 days.			vascular disease, stroke, TIA, Renal disease				
Peng 2013[198]	• County: NA<br>• English<br>• 2013<br>• 174 Patients<br>• Inclusion Criteria: patients undergoing gastrointestinal surgery for malignancy	Exclusion Criteria: NA	restricted fluid regimen (R group): NA<br>standard fluid regimen (S group): NA	Abdominal Malignancy	GI Surgery	restricted fluid regimen (R group)<br>standard fluid regimen (S group)	Weight gained	Post-operative complications<br>Fluid distribution.
Piljic 2015[199]	• Bosnia<br>• English<br>• 2015<br>• 60 Patients<br>• Inclusion Criteria: isolated stable infrarenal AAA exceeding 5.5 cm with normal dimension of supra- and juxta-renal aorta and iliac arteries.	Exclusion Criteria: emergency operations due to AAA rupture, previous abdominal surgery with laparotomy being performed for any pathology, liver, thyroid, or renal failure on dialysis, preoperative anemia, and any systemic or malignant disease.	standard fluid administration: 69.34 (8.94)<br><br>reduced fluid administration: 68.64 (7.06)	AAA, Diabetes, Hypertension.	Vascular surgery	standard fluid administration<br><br>reduced fluid administration	30-days mortality, length of ICU stay, length of hospital stay.	1) cardiovascular (myocardial infarction, arrhythmia, cardiac failure); (2) respiratory (pneumonia, pulmonary edema, respiratory failure); (3) thrombotic (thrombosis, embolism);<br>(4) hemorrhagic (re-exploration for bleeding); (5) renal (renal failure); (6) infections (wound dehiscence, other infections); (7) neurologic (depression, acute delirium); (8) gastrointestinal (obstipation, vomiting, ileus).
Shehata 2012[200]	• Canada<br>• English<br>• 2012<br>• 50 Patients	Exclusion criteria: NA	Restrictive therapy: 67.2 (11.2)<br>Liberal therapy: 68.8 (9.2)	Angina, Unstable angina,	Cardiac surgery	"restrictive" transfusion strategy received RBC transfusions if	enrollment rate and overall adherence to the	RBC transfusions, clinical outcomes, and physiologic indicators of hypoxemia

	• Inclusion Criteria: elective cardiac surgery			Myocardial infarction, valvular heart disease, hypertension, COPD, CVA, Congestive heart failure		their Hb was 70 g.L-1 or less during cardiopulmonary bypass (CPB) and 75 g.L-1 or less postoperatively after bypass. Patients allocated to a "liberal" transfusion strategy received RBC transfusions if their Hb concentration was 95 g.L-1 or less during and less than 100 g.L-1 after bypass.	transfusion strategies	(mixed venous oxygen saturation). Clinical outcomes were defined as 1) in-hospital all-cause mortality 2) a composite score of morbidity consisting of a) neurologic events defined as a new focal neurologic deficit lasting more than 24 hours or irreversible encephalopathy, b) dialysis-dependent renal failure or greater than 50% increase in creatinine, c) prolonged low cardiac output state (i.e., need for two or more inotropes for 24 hours or more, intra-aortic balloon pump or ventricular assist device for greater than 48 h), and/or myocardial infarction, defined as troponin I level greater than 2.5 mg/L and new Q waves on electrocardiogram or a clinical diagnosis; and 3) hospital lengths of stay
Van Samkar 2015[201]	• Netherlands<br>• English<br>• 2015<br>• 66 Patients<br>• Inclusion Criteria: Patients scheduled to undergo	Exclusion criteria: diabetes mellitus, renal failure, drug or alcohol abuse, clinical signs of preoperative gastroparesis, unfit to participate due to language or psychiatric	standard (S:10ml.kg-1.hr-1) fluid therapy: NA restricted (R:5ml.kg-1.hr-1) fluid therapy: NA	NA	HPB Surgery	standard (S:10ml.kg-1.hr-1) fluid therapy: restricted (R:5ml.kg-1.hr-1) fluid therapy:	The primary outcome measure was gastric emptying time (i.e. reduction of minutes	Secondary outcome parameters included postoperative surgical complications, including the incidence of delayed gastric emptying

	pancreatoduodenectomy (PD) for a suspected pancreatic head or periampullary tumor, age >18 years and American Society of Anesthesiologists (ASA) classification I-IV.	problems, contraindications for epidural (such as severe coagulation disorder), blood loss of more than 50% of circulating volume and/or postoperative ICU, or participation in another clinical trial.					needed to achieve a 50% emptying of the stomach), as measured by scintigraphy with a solid test meal on the seventh postoperative day.	(DGE) according to the definition of the International Study Group of Pancreatic Surgery (ISGPS), relaparotomy rate, non-surgical complications, mortality, duration of hospital stay, values of urea, creatinine, albumin, remaining length of duodenum and body weight
**Sodium Bicarbonate**								
Brulotte 2013[202]	• Canada<br>• English<br>• 2013<br>• 34 Patients<br>• Inclusion Criteria: patients ranging from 18 to 85 years of age, and presenting for elective endovascular aortic repair	Exclusion Criteria: patient refusal, exposure to contrast media within 14 days prior to surgery, acute renal failure on the day of surgery, defined as serum creatinine 50% above normal patient values, patients who were renal transplant recipients, and patients on dialysis for chronic renal failure.	Sodium Bicarbonate: 71.9 (6.1)<br>Sodium Chloride: 75.6 (6.0)	Aortic Aneurysm, Diabetes, Hypotension, Chronic renal failure, chronic heart failure	Vascular Surgery	Sodium Bicarbonate<br>Sodium Chloride	Incidence of AKI	Creatinine, cystatin C, serum, IL-18: interleukin-18; KIM-1: kidney injury molecule-1; NAG: N-acetyl-β-D-glucosaminidase; NGAL: neutrophil gelatinase-associated lipocalin.
Cho 2017[203]	• Korea<br>• English<br>• 2017<br>• 70 Patients<br>• Inclusion Criteria: patients between 20 and 80 years old, who were scheduled for valvular heart surgery for IE.	Exclusion Criteria: (1) estimated glomerular filtration rate (eGFR) <15 ml.min-1 per 1.73 m2; (2) receiving renal replacement therapy; (3) receiving intra-aortic balloon pump support; (4) preexisting hypernatremia, alkalosis, or severe pulmonary edema; or (5) chronic moderate dose to	Sodium bicarbonate Group: 53.9 (14.1)<br>Control Group (Sodium Chloride): 55.1 (15.7)	Hypertension, diabetes mellitus, congestive heart failure, chronic	Cardiac Surgery	Sodium bicarbonate<br>Sodium chloride	peak serum creatinine (SCr)	incidence of postoperative AKI, electrolyte abnormalities, major morbidity endpoints, ICU and hospital length of stay, and in-hospital mortality.

		high dose corticosteroid therapy (>10 mg/day prednisolone or equivalent).		kidney disease , Cerebrovascular disease , COPD				
Haase 2009[143]	• USA<br>• English<br>• 2009<br>• 100 Patients<br>• Inclusion Criteria: Age above 70 years. Preexisting renal impairment (preoperative plasma creatinine concentration >120 mol.L-1. New York Heart Association class III/IV or impaired left ventricular function (left ventricular ejection fraction <35%). Valvular surgery or concomitant valvular and coronary artery bypass graft surgery. Redo cardiac surgery. Insulin-dependent Type 2 diabetes mellitus	Exclusion criteria: End stage renal disease (plasma creatinine concentration >300 umol.L-1). Emergency cardiac surgery. Planned off-pump cardiac surgery. Known blood-bourne infectious disease. Chronic inflammatory disease on immunosuppression. Chronic moderate to high dose corticosteroid therapy (>10 mg per day prednisone or equivalent). Enrolled in conflicting research study. Age <18 years	Sodium Bicarbonate: 71.5(9.2)<br>Sodium Chloride: 70.6(9.5)	Arterial hypertension, Hypercholestrolaemia, Atrial fibrillation, COPD, Vascular disease.	Cardiac Surgery	Sodium Bicarbonate<br>Sodium Chloride	the proportion of patients developing acute renal dysfunction defined as a postoperative increase in plasma creatinine concentration >25% of baseline within the first five postoperative days.	changes in plasma creatinine, plasma urea, urinary neutrophil gelatinase-associated lipocalin, and urinary neutrophil gelatinase-associated lipocalin/urinary creatinine ratio
Haase 2013[204]	• Germany<br>• English<br>• 2013<br>• 350 Patients<br>• Inclusion Criteria: Age above 70 y. Pre-existing renal impairment (preoperative plasma. creatinine concentration >120 mmol.l-1. New York Heart Association class III/IV or impaired left. ventricular function (left	Exclusion Criteria: End stage renal disease (serum creatinine concentration >300 mmol/l). Emergency cardiac surgery. Planned off-pump cardiac surgery. Known blood-borne infectious disease. Chronic inflammatory disease on immunosuppression. Chronic moderate to high dose corticosteroid therapy (10 mg/d prednisone or	Sodium Bicarbonate: 66.4(12.1)<br>Sodium Chloride: 64.6(13.5)	Arterial hypertension, Pulmonary hypertension, Hypercholestrolaemia, Atrial fibrillation,	Cardiac Surgery	sodium bicarbonate (5.1 mmol.kg-1) or sodium chloride (5.1 mmol.kg-1).	proportion of patients developing acute kidney injury	Secondary endpoints included the magnitude of acute tubular damage as measured by urinary neutrophil gelatinase-associated lipocalin (NGAL), initiation of acute renal replacement therapy, and mortality.

	ventricular ejection fraction <35%). Valvular surgery or concomitant valvular and coronary artery bypass graft surgery. Redo cardiac surgery. Insulin-dependent Type 2 diabetes mellitus	equivalent) Enrolled in conflicting research study		Myocardial infarction, COPD, Peripheral vascular disease				
Kristeller 2013[205]	• USA<br>• English<br>• 2012<br>• 48 Patients<br>• Inclusion Criteria: patients undergoing cardiac surgery using CPB and had A baseline estimated GFR of less than 60 ml.minutes-1 per 1.73 m2 using the Modification of Diet in Renal Disease equation.	Exclusion Criteria: cardiogenic shock (defined as a cardiac index < 2 L.min-1.m-2 despite high-dose inotrope administration or the use of an intra-aortic balloon pump), had end-stage kidney disease requiring renal replacement therapy, or received a radiocontrast agent on the same day as cardiac surgery.	Sodium Bicarbonate: 72 (11)<br>Isotonic Saline: 73 (11)	Chronic lung disease, Diabetes, Hypertension, dyslipidaemia. Atrial fibrillation, atrial flutter, previous myocardial infarction, Heart Failure	Cardiac Surgery	Sodium Bicarbonate Saline	development of any stage of acute kidney injury within 5 days after surgery as defined by the Acute Kidney Injury Network criteria	increase in plasma creatinine concentration at least 25% above baseline or the development of alkalosis (pH > 7.5), hypokalemia (potassium level < 3.5 mEq.L-1), or hypernatremia (sodium level > 150 mEq.L-1). We also assessed blood transfusions, new-onset atrial fibrillation, duration of mechanical ventilation, and length of hospital stay.
McGuiness 2013[206]	• New Zealand<br>• English<br>• 2013<br>• 427 Patients<br>• Inclusion Criteria: Age > 70 years. Preexisting renal impairment (preoperative plasma creatinine concentration > 1.4 mg.dL-1.	Exclusion Criteria: End-stage renal disease (plasma creatinine concentration > 3.4 mg.dL-1). Emergency cardiac surgery<br>Planned off-pump cardiac surgery. Known blood-borne infectious disease. Chronic inflammatory disease on immunosuppression. Chronic	Sodium Bicarbonate: 66.3 (14)<br>Sodium Chloride: 67.8 (12.8)	Arterial hypertension, Hypercholesterolaemia, atrial fibrillation, recent	Cardiac surgery	Sodium Bicarbonate Saline	the number of patients who had postoperative AKI development	the number of patients who received renal replacement therapy (RRT) during their hospital stay. The number of patients with development of acute kidney dysfunction according to the Risk, Injury,

	New York Heart Association class III/IV or impaired left ventricular function (left ventricular ejection fraction < 35%). Valvular surgery or concomitant valvular and coronary artery bypass graft surgery. Redo cardiac surgery. Insulin-dependent diabetes mellitus	moderate to high dose corticosteroid therapy (>10 mg/d prednisone or equivalent). Enrolled in conflicting research study Age < 18 years		myocardial infarction, COPD, Peripheral vascular disease.				Failure, Loss, End-stage (RIFLE) and Acute Kidney Injury Network classifications (AKIN total, AKIN stage 1, AKIN stage 2, and AKIN stage 3) (26). Urine output data and any episodes of urine output less than 0.5 mL/kg/ hr in any 6- or 12-hour period or less than 0.3 mL/kg/hr in any 24-hour period was recorded.
Muralidhar 2018[207]	• India<br>• English<br>• 2019<br>• 60 Patients<br>• Inclusion Criteria: adult (aged 18 years or above) consecutive stable chronic kidney disease (CKD) patients not on dialysis who were scheduled for elective off-pump CABG (OP-CABG)	Exclusion Criteria: on-pump CABG, emergency surgery or redo operations, and those who had end-stage renal disease, chronic inflammatory disease or immune-suppression, those enrolled in a conflicting research study, and patients on corticosteroid therapy, renal replacement therapy (RRT) and renal-transplanted patients.	Sodium Bicarbonate: 60.81 (9.2)<br>Sodium Chloride: 60.87 (7.1)<br>Normal control: 57.11 (9.5)	NA	Cardiac surgery	NaHCO3 at a rate of 0.5 mmol.kg-1.h-1 during the first hour of surgery followed by 0.2 mmol.kg-1.h-1 + standard care<br><br>0.9% NaCl infusion + standard care	(Primary outcomes not defined)	Creatinine, urine output and troponin
Saratzis 2018[208]	• UK<br>• English<br>• 2018<br>• 58 Patients<br>• Inclusion Criteria: Patients scheduled to undergo elective EVAR for an infrarenal AAA	Exclusion Criteria: emergency EVAR; established severe cardiac failure defined as functional status New York Heart Association stage 4; allergy to NaHCO3; juxta-renal or suprarenal aneurysm; solitary kidney; administration of intravenous or intra-arterial contrast <2 days prior to EVAR;<br>previous open AAA or iliac aneurysm repair; surgery or	Median (range)<br>Standard hydration: 75.5 (70-80)<br>Standard hydration + bolus Bicarbonate: 74.5 (73-80)	AAA<br>Previous stroke, previous myocardial infarction, Previous TIA, Atrial fibrillati	Vascular surgery	Standard hydration<br>Standard hydration + bolus Bicarbonate	proportion of eligible patients recruited into the study	(i) incidence of AKI; (ii) tolerability of the intervention by the patients (number of patients withdrawing from the study); (iii) adequacy of the standardised hydration regimen in the two arms if the proposed fluid regimen failed to maintain an intra-operative central aortic pressure of at

		trauma within 1 month before EVAR; metabolic or respiratory alkalosis; chemotherapy, radiotherapy, or steroid therapy; patient undergoing renal dialysis for established renal failure; patient receiving nephrotoxic medication (non-steroidal anti-inflammatory drugs, angiotensin converting enzyme inhibitors, angiotensin II receptor blockers) for 48 h prior to EVAR; pulmonary oedema; systolic blood pressure > 200 mm Hg at baseline.		on, Angina, CKD				least 90% of baseline and bioimpedance indicated that post-operative hydration levels were <90% of baseline; (iv) levels of serum bicarbonate during and after the EVAR; (v) adverse events and complications 30 days after EVAR (all clinical events between recruitment and completion of follow-up were recorded during inpatient hospital stay and outpatient visits).
Soh 2016[209]	• Korea<br>• English<br>• 2016<br>• 162 Patients<br>• Inclusion Criteria: Patients scheduled to undergo elective EVAR for an infrarenal	Exclusion criteria: (i) pre-existing acute renal failure; history of renal replacement therapy, or both; (ii) steroid therapy (>10 mg per day prednisone or equivalent); (iii) pre-existing hypernatremia, alkalosis, or severe pulmonary oedema; or (iv) preexisting anaemia (haemoglobin <10 mg.dl−1).	Median (interquartile range)<br>Control: 68 (62–73)<br>Sodium Bicarbonate: 70 (64–74)	Diabetes, Hypertension, COPD, Congestive heart failure, previous myocardial infarction	Cardiac surgery	sodium bicarbonate (0.5 mmol kg−1 for 1 h upon induction of anaesthesia followed by 0.15 mmol kg−1 h−1 for 23 h)<br>0.9% saline	incidence of AKI	requirement for renal replacement therapy during first 2 days after surgery and the calculated number of patients with AKI at the various stages of the Acute Kidney Injury Network classifications (AKIN stages 1, 2, and 3),16 and the incidence of major postoperative morbidity end points (permanent stroke, mechanical ventilation >24 h, myocardial infarction, reoperation, deep sternal wound infection, and atrial fibrillation).

Turner 2014[210]	• USA<br>• English<br>• 2014<br>• 123 Patients<br>• Inclusion Criteria: Inclusion criteria: planned receipt of cardiovascular. Surgery as well as increased risk factors for AKI post-operatively. Including either an estimated GFR of <60 ml.min-1 per 1.73meters.	Exclusion criteria: Age < 18. Pre-existing ESRD (dialysis patients). Pre-op GFR ≤ 15ml.min-1.m-2. Pre-op bicarbonate level ≥ 30 mEq.L-1. Emergency surgery (unable to effectively consent). Pregnancy. Heart transplant (OHT). Distal Aortic surgery (i.e., descending aortic aneurysm). Procedure does not require central venous access	Sodium Chloride: 69.7 (13.5)<br>Sodium Bicarbonate: 70.2 (12.6)	Kidney Disease, Hypertension, Diabetes, COPD, Myocardial infection.	Cardiac surgery	Sodium chloride<br>Sodium bicarbonate	Risk of AKI	The maximum change In estimated serum creatinine and the glomerular filtration rate (GFR) during the first 72h post-operatively, LOS, incidence of dialysis (HD) and mortality.
**Statin**								
Almansob 2012 [211]	• China<br>• English<br>• 2012<br>• 151 Patients<br>• Inclusion Criteria: elective noncoronary artery cardiac surgery	Exclusion Criteria: Patients <10 years, with coronary artery disease, contraindications to statins treatment, and women who were gestating or lactating	Statin group: 45.5 (14.5)<br>Control group: 41.5 (18.7)	Chronic AF, Diabetes, Dyslipidemia, Hypertension,	Cardiac surgery	Statin group<br>Control Group	(primary outcomes not defined)	(secondary outcomes not defined)<br><br>Plasma TnT, Plasma CKMB, Plasma CRP, Blood Urea Nitrogen, Serum Creatinine, Plasma IL-6, and IL-8, NO Generation, Inotropic Requirement and Left Ventricular Ejection Fraction, Akt-eNOS, p38 MAPK, Caveolin-1, Hsp90 Expression
Billings 2016 [212]	• USA<br>• English<br>• 2012<br>• 151 Patients<br>• Inclusion Criteria: Adult patients undergoing elective coronary artery bypass grafting, valvular heart surgery, or ascending aortic surgery at Vanderbilt	Exclusion Criteria: (1) prior statin intolerance;<br>(2) acute coronary syndrome (defined as ST or non-ST elevation myocardial infarction with elevated serum troponin concentrations); (3) liver dysfunction (defined as serum transaminase concentrations >3 times the	median (10th-90th percentile)<br>Atorvastatin: 66 (49-81)<br>Placebo: 67 (51-81)	Congestive heart failure, Myocardial infarction, Atrial fibrillation,	Cardiac Surgery	Atorvastatin<br>Placebo	diagnosis of AKI according to criteria from the Acute Kidney Injury Network (AKIN),	maximum increase in creatinine concentration from baseline to 48 hours following surgery (postoperative day 2), the incidence and duration of delirium while in the intensive care unit (ICU) (incorrectly

	University Medical Center	upper limit of normal [120 U.L-1], a bilirubin concentration >3 mg.dL-1, or a diagnosis of cirrhosis); (4) current use of potent CYP3A4 inhibitors, including azole antifungals, protease inhibitors, and macrolide antibiotics; (5) current use of cyclosporine; (6) current use of renal replacement therapy; (7) a history of kidney transplant; (8) required emergency or urgent surgery; or (9) were pregnant.		Prior cardiac surgery , diabete s, COPD, OSA, CVA, TIA				noted as a co-primary end point during the editing of the study registration), the degree of myocardial injury, and the incidence of postoperative atrial fibrillation, pneumonia, or str
Park 2016[213]	• USA<br>• English<br>• 2012<br>• 151 Patients<br>• Inclusion Criteria: statin-naïve patient (older than 20 years) scheduled for elective valvular heart surgery	Exclusion criteria: pre-existing renal dysfunction (estimated glomerular filtration rate less than 15 ml.min-1 per 1.73 m2; calculated from the Modification of Diet in Renal Disease Study equation), left ventricular ejection fraction less than 30 %, severe coronary artery occlusive disease, active liver disease or cirrhosis, preoperative unexplained elevation of serum transaminases, history of rhabdomyolysis, preoperative unexplained elevation of creatinine kinase, hemodynamically unstable arrhythmia, preoperative cardiogenic shock, or mechanical support.	Statin: 58 (12)<br>Control: 58 (14)	Hypert ension, Diabete s, Hyperli pidemi a, history of stroke	Cardiac Surgery	Atorvastatin<br>Placebo	incidence of postoperative AKI as defined by AKIN	The secondary end points were changes in serum biomarkers including serum creatinine, plasma NGAL, and IL-18 during the perioperative period

Prowle 2012[214]	• Australia<br>• English<br>• 2012<br>• 100 Patients<br>• Inclusion Criteria: Elective cardiac surgery. Planned cardiopulmonary bypass. >1 risk factors for postoperative renal dysfunction:<br>• Age >70 years<br>• Congestive heart failure (New York Heart Association (NYHA) symptom severity class 3 or 4)<br>• Left ventricular ejection fraction <35%<br>• Insulin-requiring diabetes mellitus<br>• Prior cardiac surgery<br>• Valvular surgery +/- coronary artery bypass<br>• Preoperative creatinine >106.1 mmol.L-1 (1.2 mg.dL-1)	Exclusion Criteria:<br>Emergent cardiac surgery, cardiac transplant or insertion of device<br>Planned off-pump cardiac surgery Hypersensitivity, allergy or known intolerance to statins. Premorbid end-stage kidney disease or renal transplant. Preoperative acute kidney injury, defined as an increase in plasma creatinine 88.4 mmol.L-1 (1.0 mg.dL-1) from preadmission to operation. Active liver disease or cirrhosis. Preoperative unexplained elevation of serum transaminases. Enrolled in a conflicting study. Age < 18. Pregnancy	Atorvastatin: 69.0 (11.1)<br>Placebo: 67.3 (10.8)	Arterial Hypertension, hypercholesterolaemia, atrial fibrillation, previous myocardial infarction, COPD, Vascular disease.	Cardiac Surgery	Atorvastatin<br>Placebo	plasma creatinine	AKI defined by the creatinine criteria of RIFLE consensus classification (RIFLE R, I or F), change in urinary neutrophil gelatinase-associated lipocalin (NGAL) concentration, requirement for renal replacement therapy, length of stay in intensive care, length of stay in<br>hospital and hospital mortality.
Zheng 2016[215]	• China<br>• English<br>• 2012<br>• 1922 Patients<br>• Inclusion Criteria: Men and women who were 18 years of age or older and were scheduled to undergo elective coronary-artery bypass grafting (CABG), surgical aortic-valve replacement, or both were eligible if they were in sinus rhythm and were not taking antiarrhythmic medication	Exclusion Criteria: moderate or severe mitral-valve disease or known renal dysfunction (creatinine level, >2.3 mg per deciliter [200 µmol per liter]) or had contraindications<br>to statin therapy.	Rosuvastatin: 59.3(9.4)<br>Placebo: 59.5(9.5)	Hypertension, Myocardial infarction, Stroke, peripheral arterial disease, heart failure, COPD, Diabetes mellitu	Cardiac surgery	Rosuvastatin<br>Placebo	postoperative atrial fibrillation within 5 days after surgery, as assessed by Holter electrocardiographic monitoring, and myocardial injury within 120 hours after surgery, as assessed by serial measurements of the<br>cardiac troponin	major in-hospital adverse events, duration of stay in the hospital and intensive care unit, left ventricular<br>and renal function, and blood biomarkers

	(other than betablockers).			s, Chronic kidney disease .			I concentration	
**Steroid Therapy**								
Dieleman 2012[216]	• Netherlands<br>• English<br>• 2012<br>• 4494 Patients<br>• Inclusion Criteria: Patients aged 18 years or older who were scheduled for any type of elective or urgent cardiac surgical procedure requiring CPB.	Exclusion Criteria: emergent or off-pump procedure and a life expectancy of less than 6 months.	Dexamethasone: 66.2 (11.0)<br>Placebo: 66.1 (10.7)	Hypertension, Diabetes, previous stroke, previous TIA, Peripheral vascular disease, chronic renal dysfunction,	Cardiac Surgery	Dexamethasone(1 mg/kg of body weight,<br>With a 100mg maximum) or placebo	Major adverse events - a composite of death, myocardial infarction (MI), stroke, renal failure, or respiratory failure, occurring within 30 days of randomization.	death, MI, stroke, renal failure, or respiratory failure, within<br>the first 30 days; postoperative infections; postoperative atrial fibrillation;<br>highest serum glucose concentration in<br>the ICU; highest body temperature in the ICU; postoperative delirium (defined as the postoperative indication for treatment with neuroleptic drugs); time to<br>weaning from postoperative mechanical ventilation; and time to discharge<br>from the ICU and from the hospital.
Garg 2019[217]	• London<br>• English<br>• 2019<br>• 7286 Patients<br>• Inclusion Criteria: Patients aged 18 years or older who were scheduled for any type of elective or urgent cardiac	Exclusion Criteria: those with Pre-randomization end-stage kidney disease (i.e., patients with an<br>estimated glomerular filtration rate of < 15 mL.min-1. Per 1.73 m2 [calculated using the Chronic Kidney	Methylprednisolone: 68 (14)<br>Placebo: 68 (14)	Hypertension, Congestive heart failure, diabetes, atrial	Cardiac Surgery	Methylprednisolone<br>Placebo	postoperative acute kidney injury, defined as an increase in the<br>serum creatinine concentration	Percentage change and absolute change in serum creatinine concentration

	surgical procedure requiring CPB.	Disease Epidemiology Collaboration equation] or patients receiving dialysis), those missing a pre-randomization serum creatinine measurement (which is needed to define acute kidney injury) and those who did not undergo surgery.		fibrillation, peripheral artery disease.			(from the preoperative value) of 0.3 mg.dL-1 or greater (≥ 26.5 µmol.L-1) or 50% or greater in the 14-day period after surgery, or use of dialysis within 30 days after surgery.	
Loef 2004 [218]	• Netherlands<br>• English<br>• 2004<br>• 20 Patients<br>• Inclusion Criteria: patients with normal renal function as assessed by a serum creatinine of less than 120 mmol per litre and normal urinalysis. All patients had coronary artery disease but normal cardiac (ejection fraction more than 45%), cerebral and hepatic function.	Exclusion criteria: Patients with diabetes, recent myocardial infarction, hypertension, unstable angina, or recent use of radiocontrast media.	Median (Range)<br>Dexamethasone: 67.7 (58–76)<br>Placebo: 59.6 (47–76)	NA	Cardiac Surgery	Dexamethasone<br>Placebo	Primary outcomes not defined	Secondary outcomes not defined.<br><br>Urine output, urinary glucose, Changes in (A) fractional excretion of sodium, (B) free water clearance (Cl water), and (C) urinary N-acetyl-b-D-glucosaminidase (NAG)
Morariu 2005[219]	• Netherlands<br>• English<br>• 2004<br>• 20 Patients<br>• Inclusion Criteria: first-time coronary artery revascularization. normal renal function (as assessed by a serum creatinine level < 120 mol/L and normal urinalysis results) and normal hepatic, cerebral, and cardiac function	Exclusion criteria: diabetes, recent myocardial infarction, unstable angina, or recent use of radiocontrast agents or corticosteroids were excluded	Mean(95% CI)<br>Placebo Group: 59.5 (53.4–65.5)<br>Dexamethasone Group: 67.8 (63.4–72.1)	NA	Cardiac surgery	Placebo Group<br>Dexamethasone Group	Primary outcomes not defined	Secondary Outcomes not defined<br><br>Inflammatory markers: (1) IL-6, IL-8, IL-10: solid-phase, enzyme-labeled, chemiluminescent sequential immunometric assay (Immulite; Euro/DPC; Los Angeles, CA); (2) C-reactive

	(ejection fraction > 45%).								protein (CRP)<br><br>Myocardial injury markers<br>(1) plasma heart-type fatty acid binding protein (H-FABP; cytosolic protein released from injured myocytes). (2) cardiac troponin I (cTnI; myofibrillar protein released<br>from injured myocytes).<br>(3) creatine kinase MB (CK-MB) activity:<br><br>Kidney injury markers<br>N-acetyl-glucosaminidase<br>(NAG; enzyme released from injured proximal renal tubules)<br><br>Hepatic injury biomarkers<br>-Glutathione S-transferase (-<br>GST; enzyme released from centrilobular and periportal damaged Hepatocytes<br><br>Intestinal Injury Biomarkers: Urinary I-FABP and L-FABP
Turner 2008[220]	• UK<br>• English<br>• 2008<br>• 20 Patients<br>• Inclusion Criteria: Adult patients aged over 60 years	Exclusion Criteria: diabetes mellitus, current steroid medication,<br>active infection or inflammatory aneurysm (diagnosed by<br>computed tomography or	methylprednisolone 10 mg.kg-1: 71·9 (6·0)<br>dextrose (control): 69·1(5·4)	AAA	Vascular surgery	methylprednisolone 10 mg.kg-1<br>dextrose (control)	difference in urinary β-NAG/creatinine ratio.	cytokine levels, length of hospital stay and 30-day mortality.

	undergoing repair of an infrarenal aortic aneurysm were included	clinically at the time of surgery),<br>and chronic renal dysfunction (serum creatinine level above 150 μmol.l-1).						
**Vasopressor / Vasoconstrictor**								
Abbas 2019[221]	• Saudi Arabia<br>• English<br>• 2019<br>• 50 Patients<br>• Inclusion Criteria: adult patients aged > 18 years old with American Society of Anesthesiologists (ASA) Classification (Class I-II) undergoing major elective hepatobiliary surgery	Exclusion criteria included patients<br>with preoperative renal failure, severe liver dysfunction (Child-Turcotte-Pugh grade C), hyponatremia (Na+ < 132 mmol.l-1), severe valvular heart disease, heart failure, symptomatic coronary heart disease, bradycardic arrhythmia (heart rate < 60 per min), peripheral artery occlusive disease (clinical stadium II-IV), uncontrolled arterial hypertension (Blood pressure > 160/100 mmHg despite intensive treatment), pregnancy and intraoperative need for Pringle maneuver.	Terlipressin: 58.7 (5.9)<br>Control: 55.5 (8.4)	NA	HPB Surgery	Terlipressin (bolus dose of (1 mg over 30 min) followed by a continuous infusion of 2 μg.kg-1.h-1)<br>Control	hepatic hemodynamics (portal venous pressure).	Secondary goals included the effects of terlipressin infusion upon intraoperative systemic hemodynamics, estimated blood loss during open hepatobiliary surgery, and the early postoperative renal function
Futier 2017[222]	• France<br>• English<br>• 2017<br>• 298 Patients<br>• Inclusion Criteria: aged 50 years or older, were scheduled to undergo surgery under general anesthesia with an expected duration of 2 hours or longer, had an American Society of Anesthesiologists physical status of class II or higher, had	Exclusion Criteria: severe uncontrolled hypertension (SBP ≥180 mm Hg or diastolic blood pressure ≥110 mm Hg); had chronic kidney disease (glomerular filtration rate <30 mL.min-1 per 1.73 m2 or requiring<br>renal replacement therapy for end-stage renal disease); had acute or decompensated heart failure or acute coronary syndrome;	individualized treatment strategy: 69.7 (7.1)<br>Standard: 70.0 (7.5)	Chronic arterial hypert ension, Chronic heart failure, Ischae mic heart disease , renal impair	Mixed Surgery	standard<br><br>individualized treatment strategy.	composite of systemic inflammatory response syndrome and dysfunction of at least 1 organ system of the renal, respiratory, cardiovascular, coagulation, and neurologic	individual components of the primary outcome, durations of ICU and hospital stay, adverse events, and all-cause mortality at 30 days after surgery.

	a preoperative acute kidney injury risk index of class III or higher	had preoperative sepsis or were already receiving norepinephrine infusion; required renal vascular surgery; or were enrolled in another study.		ment, diabetes mellitus.			systems by day 7 after surgery	
Urzua 1992[223]	• Chile<br>• English<br>• 1992<br>• 21 Patients<br>• Inclusion Criteria: normal renal function (plasma creatinine cl.2 mg.dL-1, creatinine clearance >70 mL.min-1), aged 50 to 70 years, without associated pathology	Exclusion Criteria: Patients with unstable angina, and those receiving inotropes or exposed to radiologic contrast medium in the previous 72 hours,	Phenylephrine: 55 (7)<br>No Treatment: 54 (7)	NA	Cardiac Surgery	phenylephrine to maintain their mean arterial pressure (MAP) > 70 mm Hg<br><br>No treatment	Primary outcome measures not defined	Secondary outcome measures not defined<br><br>Creatinine clearance, perfusion pressure, post-operative renal function, urinary output, plasma sodium concentration, osmolal clearance
Wu 2017b 1[112]	• China<br>• English<br>• 2017<br>• 678 Patients<br>• Inclusion Criteria: 1) patients were 65–80 years old; 2) patients had American Society of Anesthesiologists (ASA) physical status grade I to III disease with a predicted surgery time N60 min; 3) no surgery for pre-existing renal disease; 4) current left ventricular ejection fraction N50%; and 5) no sign of cardiac dysfunction.	Exclusion Criteria: 1) patients used non-steroidal anti-inflammatory drugs during the past month; 2) patients had heart failure during the past 2 months; 3) patients had myocardial infarction during the past month (confirmed by blood-specific enzymes); 4) current severe pulmonary function insufficiency; 5) current intermediate to severe pulmonary hypertension; and 6) chronic kidney diseases or renal dysfunction (confirmed by previous physician's diagnosis).	Level 1: 73 (7)<br>Level 2: 73 (6)<br>Level 3: 74 (5)	GI Cancer Chronic Hypertension	GI Surgery	• MAP, 65–79mmHg<br>• MAP, 80–95mmHg;<br>• MAP, 96–110mmH	incidence of AKI	incidence of surgical site infection, hospital-acquired pneumonia, stroke, admission to the intensive care unit (ICU), stay in the ICU, length of hospital stay, and 28-day mortality.
**Volatile vs Propofol Anaesthesia**								

Zhao 2017[224]	• China<br>• English<br>• 2017<br>• 80 Patients<br>• Inclusion Criteria: all patients had normal renal and hepatic functions, had cardiac surgical indications, were informed of and signed the informed written consents.	Exclusion criteria: infection, renal and hepatic dysfunction, allergy to anesthetic agents, psychological disorders and mental diseases or failed to compliance with physicians' orders due to the lack of a clear awareness	sevoflurane anesthesia (SA) group: 56.7(7.6)<br>Total intravenous anesthesia (TIVA) group: 54.3(7.4)	NA	Cardiac Surgery	sevoflurane anesthesia (SA) group<br><br>Total intravenous anesthesia (TIVA) group	first, the time to pain-free, loss of eyelash reflex and intubation, as well as the total dosage of dopamine and of nitroprusside; second, the mean arterial pressure (MAP), heart rate and arterial oxygen saturation (SpO2) before and after induction, immediately and 3min after intubation.	CPB time, anesthesia time, operation time, urine volume and length of hospital stay, adverse reactions, complications and return of spontaneous heartbeat.
Song 2013 2[225]	• China<br>• English<br>• 2013<br>• 200 Patients<br>• Inclusion Criteria: ASA I-III patients undergoing liver resection for a variety of hepatic tumours. Aged 18–75 years of age and weighed 45–90 kg.	Exclusion criteria: patients who had developed ascites, renal dysfunction (Serum creatinine > 1.5 mg.dl-1), heart failure (ejection fraction < 40%) and/or severe respiratory disease (vital capacity or/and forced expiratory volume < 50%)	Sevoflurane group: 47.6 (8.6)<br>Propofol group: 49.9 (8.7)	Cirrhosis	HPB Surgery	Sevoflurane group<br>Propofol group	Primary endpoints not defined<br><br>difference between the preoperative baseline and the highest values of serum creatinine and blood urea nitrogen measured at day 1, 3 and 6 postoperatively.	Secondary endpoints not defined<br><br>Blood loss, transfusion requirement, hospital length of stay, fluid management, urine output, furosemide administration,
Yoo 2014[226]	• Korea<br>• English	Exclusion Criteria: pre-existing renal insufficiency, serum creatinine level	Sevoflurane: 58.8(12.3)<br>Propofol: 58.1(12.2)	Diabetes, Hypert	Cardiac Surgery	Sevoflurane<br>Propofol	incidence of AKI	changes in serum biomarkers of renal injury and

	• 2014<br>• 112 Patients<br>• inclusion Criteria: Patients undergoing valvular heart surgery	(1.5mg.dl-1 in men or 1.3mg.dl-1 in women),36 older than 80 years, coronary artery occlusive disease, hepatic or pulmonary disease, active infective endocarditis, left ventricular ejection fraction of 30%, myocardial infarction within 4 weeks, or with a history of hypersensitivity to propofol. In addition, patients undergoing surgery requiring hypothermic circulatory arrest were also excluded.		ension, CVA, Congestive heart failure, COPD				inflammatory mediators, and occurrence of postoperative complications. To evaluate the degree of inflammation, IL-1, IL-6, and tumor necrosis factor-a were assessed at 15 min after anesthetic induction and after 1, 6, and 24 h after declamping of the ACC. CRP was measured 1 day before the operation and POD 1. Neutrophil counts were determined 1 day before the operation, upon ICU arrival, and POD 1, 2, and 3. CKMB was measured 1 day before the operation, and at 24 and 48 h after the operation.
Landoni 2019[227]	• Italy<br>• English<br>• 2019<br>• 5400 Patients<br>• inclusion Criteria: 18 years of age or older and if they were scheduled to undergo elective, isolated CABG.	Exclusion Criteria: planned concomitant valve surgery or aortic surgery; unstable angina; myocardial infarction in the previous 30 days; current use of a sulfonylurea, allopurinol, or theophylline; participation in other randomized, controlled trials in the previous 30 days; general anesthesia in the previous 30 days; non-elective CABG; previous kidney or liver transplantation; cirrhosis; and a previous adverse response to any of the trial anesthetic agents.	volatile anesthetics group: 62.2(8.3)<br>total intravenous anesthesia group: 62.3(8.41)	Diabetes, Hypertension, COPD, Atrial fibrillation, Previous stroke, previous TIA.	Cardiac Surgery	volatile anesthetics group<br>total intravenous anesthesia group	death from any cause at 1 year	death from any cause at 30 days, a composite of nonfatal myocardial infarction at 30 days or death at 30 days, death from cardiac causes at 30 days and at 1 year, hospital readmission during follow-up, and duration of stay in an intensive care unit and in the hospital.

Wasowicz 2018[228]	• Canada<br>• English<br>• 2018<br>• 157 Patients<br>• inclusion Criteria: patients scheduled for elective or urgent CABG with preserved ventricular function (ejection fraction > 40%).	Exclusion Criteria: history of malignant hyperthermia or propofol infusion syndrome, emergency surgery (patients in cardiogenic shock or ongoing ischaemia), history of severe kidney disease (glomerular filtration rate below 30 mL min-1) or severe liver disease (bilurubin > 2 mg dL-1) and poorly controlled diabetes (glycosylated haemoglobin > 9%).	Volatile anaesthesia: 65 (9)<br>Propofol-based anaesthesia: 63 (10)	COPD, Cardiovascular disease, Diabetes, congestive heart failure,	Cardiac surgery	Volatile anaesthesia propofol-based anaesthesia	myocardial injury measured by serial troponin measurement	secondary outcome was cardiac performance expressed as cardiac index (CI) and the need for inotropic and vasopressor drug support

eTable 1: Characteristics of included studies

	Number of studies	Total population	Individual interventions
**ACE Inhibitor**	6	621	Ramipril (2), Captopril (1), Enalapril (3)
**Alpha 2 agonist**	17	9167	Dexmedetomidine (15), Clonidine (2)
**Atrial Natriuretic Peptide**	14	2207	Nesiritide (3), Carperitide (1), ANP (10)
**Calcium Channel Blocker**	13	544	Diltiazem (4), Nifedipine (2), Nicardipine (5), Felodipine (1), Verapimil (1), Nimodpine (1).
**Dopamine Agonist**	23	1864	Fenoldapam (4), Dopexamine (3), Dopamine (16)
**Erythropoeitin**	5	376	EPO (5)
**Glycaemic Control**	3	240	Tight glycaemic control (2), Insulin infusion (1)
**Goal Directed Therapy**	37	4338	GDT (37)
**Inodilators**	13	2941	Levosimendan (13)
**Loop Diuretics**	5	448	Furosemide (5)
**Miscellaneous-Anti-oxidant**	5	382	Glutathione (1), Selenium (1), Vitamin C (1), Acetaminophen(1), Vitamin E (1), Antioxidants (1)
**Miscellaneous**	25	3502	Curcumin (1), E-aminocaproic acid(1), HOE 140 (bradykinin B2 receptor antagonist) (1), Candesartan(1), Prostaglandin E(1), Triiodothyronine(1), Carnitine(1), SiRNA(1), Cyclosporine(1), Glucose-Insuline-Potassium(1), Sitagliptin(1), Minocycline(1) , THR 184(1), Tolvaptan(1), Theophylline(1), Pentoxifylline(1), Prostacyclin(1), Sodium Selenite(1), Aminophylline(1), Magnesium Sulphate(1), Ulinastatin(1), Xenon(1), ursodeoxycholic Acid(1), Epidural + general anesthesia(1), General Anaesthesia(1), Ketamine (1).
**N-Acetylcysteine**	17	1934	NAC (17)
**Nitric Oxide Donor**	2	484	Nitric Oxide (1), Sodium Nitroprisside (1)
**Osmotic Diuretics**	6	295	Mannitol (6)
**Other Diuretics**	2	706	spironolactone(2)
**Restrictive vs Liberal**	16	10430	Restrictive (16)
**Sodium Bicarbonate**	10	1482	Sodium Bicarbinate (10)
**Statin**	5	2961	Statin (1), Atorvastatin(2), simvastatin (1), Resouvastatin (1)
**Steroid Therapy**	5	11840	Dexamethasone (3), Methylprednisolone (2)
**Vasopressor / Vasoconstrictor**	4	1047	Terlipressin (1), Noradrenalin (1), Phenylephrine (1), Medium MAP Target (1)
**Volatile vs Propofol Anaesthesia**	4	5869	Sevoflurane (4)

eTable2: Summary of Included Studies.

All Studies	30 Day Mortality	Risk of AKI	Renal Replacement Therapy	Urine output 24 hours	Creatinine Clearance (24H)	Creatinine Clearance (2-4 days)	Creatinine Clearance (5-7 days)	Perioperative - Blood Loss	Adverse Reaction	Myocardial Infarction	Low Cardiac Output	Acute Brain Injury/Stroke	sepsis / infection	ICU LOS	Hospital LOS
**Atrial Natriuretic Peptide**	RR 0.63<br>(0.41, 0.97)<br>I2 0%	RR 0.43<br>(0.33, 0.56)<br>I2 0%	RR 0.26<br>(0.15, 0.47)<br>I2 0%	MD 0.47<br>[-0.37, 1.32]<br>I2 99%	MD 15.22<br>[-20.49, 50.94]<br>I2 100%	MD 19.70<br>[-5.85, 45.24]<br>I2 97%	MD 11.29<br>[-8.35, 30.93]<br>I2 95%	MD 162.00<br>[-454.97, 778.97]<br>I2 NA	RR 0.95<br>(0.61, 1.48)<br>I2 30%	RR 0.98<br>(0.31, 3.10)<br>I2 0%	RR 0.93<br>(0.80, 1.09)<br>I2 18%	RR 0.59<br>(0.18, 1.93)<br>I2 0%	RR 3.36<br>(0.81, 13.88)<br>I2 0%	MD -0.28<br>[-0.98, 0.41]<br>I2 47%	MD -2.60<br>[-3.69, -1.50]<br>I2 56%
**Alpha 2 agonist**	RR 0.49<br>(0.22, 1.11)<br>I2 0%	RR 0.68<br>(0.47, 0.99)<br>I2 59%	RR 0.76<br>[0.46, 1.27]<br>I2 35%	MD 0.12<br>[-0.04, 0.28]<br>I2 91%	MD 10.67<br>[2.57, 18.77]<br>I2 71%	MD 4.50<br>[1.78, 7.22]<br>I2 28%	MD -25.75<br>[-51.54, 0.04]<br>I2 96%	MD -10.17<br>[-57.70, 37.35]<br>I2 9%	N/A	RR 0.52<br>(0.09, 2.93)<br>I2 0%	RR 0.76<br>(0.53, 1.10)<br>I2 58%	RR 0.44<br>(0.21, 0.93)<br>I2 0%	RR 0.44<br>(0.21, 0.94)<br>I2 0%	MD -0.02<br>[-0.40, 0.36]<br>I2 95%	MD -0.62<br>[-1.13, -0.12]<br>I2 52%
**Inodilators**	RR 0.71<br>(0.53, 0.94)<br>I2 0%	RR 0.65<br>(0.50, 0.85)<br>I2 0%	RR 0.63<br>(0.46, 0.85)<br>I2 0%	MD -0.21<br>[-0.56, 0.14]<br>I2 NA	N/A	N/A	N/A	N/A	RR 5.00<br>(0.59, 42.34)<br>I2 N/A	RR 0.90<br>(0.60, 1.35)<br>I2 20%	RR 0.99<br>(0.80, 1.21)<br>I2 61%	RR 0.91<br>(0.64, 1.30)<br>I2 0%	RR 0.77<br>(0.45, 1.31)<br>I2 34%	MD -0.32<br>[-1.20, 0.56]<br>I2 92%	MD 0.68<br>[-1.37, 2.74]<br>I2 93%
**Vasopressor**	RR 1.05<br>(0.50, 2.21)<br>I2 0%	RR 0.56<br>(0.36, 0.86)<br>I2 0%	RR 1.00<br>(0.41, 2.45)<br>N/A	MD -0.35<br>[-0.80, 0.10]<br>I2 NA	MD -36.20<br>[-84.25, 11.85]<br>I2 NA	N/A	MD 1.40<br>[-22.68, 25.48]<br>I2 NA	MD -113.97<br>[-333.16, 105.21]<br>I2 88%	N/A	P 0.50<br>RR 0.33 (0.01,<br>8.12) N/A	RR 0.33<br>(0.04, 2.99)<br>I2 N/A	RR 0.89<br>(0.11, 7.17)<br>I2 0%	RR 0.63<br>(0.38, 1.06)<br>I2 0%	MD -0.53<br>[-1.51, 0.45]<br>I2 59%	MD -2.00<br>[-4.37, 0.37]<br>I2 NA
**Calcium Channel Blocker**	N/A	RR 0.22<br>(0.07, 0.76)<br>I2 0%	RR 0.19<br>(0.05, 0.75)<br>I2 0%	MD -0.01<br>[-0.36, 0.34]<br>I2 74%	MD -3.55<br>[-20.40, 13.30]<br>I2 93%	MD 45.16<br>[4.09, 86.23]<br>I2 0%	MD 22.30<br>[-16.37, 60.97]<br>I2 NA	MD -24.83<br>[-193.42, 143.76]<br>I2 30%	N/A	RR 0.33<br>(0.04, 2.98)<br>I2 0%	RR 0.70<br>(0.25, 1.96)<br>I2 0%	RR 1.00<br>(0.07, 14,21)<br>I2 N/A	N/A	N/A	MD 0.00<br>[-1.96, 1.96]<br>I2 92%
**Nitric Oxide Donor**	RR 0.38<br>(0.10, 1.49)<br>I2 0%	RR 0.73<br>(0.58, 0.92)<br>I2 N/A	RR 0.68<br>(0.20, 2.34)<br>I2 N/A	MD 0.16<br>[-0.01, 0.33]<br>I2 NA	MD 6.60<br>[2.41, 10.79]<br>I2 NA	MD 5.90<br>[2.04, 9.76]<br>I2 NA	MD 3.10<br>[-0.81, 7.01]<br>I2 NA	N/A	N/A	RR 0.70<br>(0.16, 3.07)<br>I2 N/A	N/A	RR 0.31<br>(0.01, 7.58)<br>I2 N/A	RR 0.94<br>(0.06, 14.78)<br>I2 N/A	MD 0.00<br>[-0.32, 0.32]<br>I2 NA	MD 0.00<br>[-0.64, 0.64]<br>I2 NA
**Statin**	RR 3.63<br>(1.01, 13.03)<br>I2 0%	RR 1.19<br>(0.96, 1.48)<br>I2 42%	RR 1.22<br>(0.68, 2.18)<br>I2 0%	MD -0.01<br>[-0.32, 0.30]<br>I2 NA	N/A	N/A	N/A	N/A	RR 1.28<br>(0.60, 2.72)<br>I2 0%	RR 0.90<br>(0.59, 1.40)<br>I2 N/A	RR 0.93<br>(0.71, 1.22)<br>I2 0%	RR 1.19<br>(0.61, 2.30)<br>I2 0%	RR 0.70<br>(0.37, 1.34)<br>I2 59%	MD 0.09<br>[-0.11, 0.29]<br>I2 34%	MD 0.03<br>[-0.90, 0.96]<br>I2 49%
**Other Diuretics (Spironolactone)**	RR 0.83<br>(0.15, 4.75)<br>I2 34%	RR 1.49<br>(1.05, 2.14)<br>I2 N/A	RR 1.14<br>(0.46, 2.87)<br>I2 N/A	N/A	N/A	N/A	N/A	MD -18.00<br>[-90.93, 54.93]<br>I2 NA	RR 0.62<br>(0.17, 2.25)<br>N/A	RR 0.99<br>(0.19, 5.30)<br>I2 N/A	RR 1.86<br>(0.64,5.42)<br>I2 N/A	N/A	RR 0.33<br>(0.06, 1.94)<br>I2 N/A	MD -1.00<br>[-1.55, -0.45]<br>I2 NA	MD -1.00<br>[-2.92, 0.92]<br>I2 NA
**Restrictive vs Liberal**	RR 0.95<br>(0.80, 1.13)<br>I2 0%	RR 1.64<br>(1.29, 2.09)<br>I2 0%	RR 1.22<br>(0.70, 2.11)<br>I2 19%	MD -0.65<br>[-0.78, -0.52]<br>I2 NA	N/A	N/A	N/A	MD 5.26<br>[-4.53, 15.05]<br>I2 4%	RR 0.69<br>[0.12, 3.98]<br>I2 NA	RR 1.00<br>(0.81, 1.23)<br>I2 0%	RR 1.15<br>(0.61, 2.15)<br>I2 16%	RR 1.18<br>(0.88, 1.58)<br>I2 0%	RR 1.01<br>(0.60, 1.70)<br>I2 33%	MD 0.40<br>[0.30, 0.50]<br>I2 NA	MD 0.64<br>[-0.10, 1.38]<br>I2 32%
**ACE Inhibitor**	RR 1.91<br>(0.31, 11.63)<br>I2 0%	RR 0.74<br>(0.23 , 2.36)<br>I2 N/A	RR 0.95<br>[0.04, 22.78]<br>I2 N/A	MD 0.48<br>[-0.37, 1.32]<br>I2 77%	MD 24.00<br>[5.15, 42.85]<br>I2 NA	N/A	N/A	MD 33.00<br>[4.90, 61.10]<br>I2 NA	RR 0.75<br>(0.22, 2.56)<br>I2 N/A	RR 0.50<br>(0.03, 7.83)<br>I2 N/A	RR 1.12<br>(0.72, 1.74)<br>I2 26%	RR 0.59<br>(0.04, 8.83)<br>I2 N/A	RR 0.66<br>(0.15, 2.88)<br>I2 N/A	N/A	MD -1.39<br>[-1.72, -1.06]<br>I2 0%
**Dopamine Agonist**	RR 1.06<br>(0.80, 1.39)<br>I2 0%	RR 0.81<br>(0.54, 1.22)<br>I2 31%	RR 0.91<br>[0.61, 1.35]<br>I2 22%	MD 0.21<br>[-0.12, 0.55]<br>I2 87%	MD 20.90<br>[13.44, 28.35]<br>I2 73%	MD 7.26<br>[-6.24, 20.77]<br>I2 93%	MD -5.24<br>[-23.74, 13.25]<br>I2 78%	MD 70.00<br>[-172.18, 312.18]<br>I2 NA	RR 3.09<br>(0.64, 14.95)<br>I2 N/A	RR 0.98<br>(0.11, 8.64)<br>I2 0%	RR 1.07<br>(0.93, 1.23)<br>I2 15%	RR 0.58<br>(0.16, 2.08)<br>I2 0%	RR 0.82<br>(0.25, 2.70)<br>I2 22%	MD -1.03<br>[-3.17, 1.11]<br>I2 0%	MD 0.33<br>[-2.19, 2.85]<br>I2 0%
**Erythropoeitin**	RR 0.53<br>(0.16, 1.80)<br>I2 0%	RR 0.75<br>(0.50, 1.13)<br>I2 40%	RR 0.42<br>(0.16, 1.08)<br>I2 0%	MD 0.06<br>[-0.37, 0.49]<br>I2 0%	N/A	N/A	N/A	MD 200.00<br>[40.20, 359.80]<br>I2 NA	N/A	RR 3.87<br>(0.44, 34.13)<br>I2 0%	RR 0.85<br>(0.03, 23.06)<br>I2 79%	RR 3.00<br>(0.13, 71.22)<br>I2 N/A	RR 0.62<br>(0.08, 4.90)<br>I2 0%	MD -0.33<br>[-0.76, 0.11]<br>I2 0%	MD 0.05<br>[-1.24, 1.34]<br>I2 0%
**Glycaemic Control**	RR 0.61<br>(0.15, 2.51)<br>I2 0%	RR 0.34<br>(0.11, 1.04)<br>I2 0%	RR 0.49<br>(0.09, 2.50)<br>I2 N/A	N/A	N/A	N/A	N/A	MD 41.80<br>[-182.07, 265.67]<br>I2 NA	N/A	RR 0.76<br>(0.18, 3.27)<br>I2 N/A	RR 0.62<br>(0.45, 0.86)<br>I2 0%	RR 0.72<br>(0.24, 2.21)<br>I2 0%	RR 0.44<br>(0.24, 0.81)<br>I2 23%	N/A	MD -16.80<br>[-40.74, 7.14]<br>I2 NA

**Goal Directed Therapy**	RR 0.85<br>(0.61, 1.18)<br>I2 0%	RR 0.91<br>(0.75, 1.10)<br>I2 21%	RR 0.79<br>(0.51, 1.22)<br>I2 6%	MD -0.07<br>[-0.27, 0.13]<br>I2 85%	MD -10.00<br>[-38.06, 18.06]<br>I2 NA	N/A	MD -25.00<br>[-48.87, -1.13]<br>I2 NA	MD -24.23<br>[-56.84, 8.39]<br>I2 72%	RR 1.00<br>(0.74, 1.35)<br>I2 N/A	RR 0.51<br>(0.26, 1.0)<br>I2 0%	RR 0.90<br>(0.74, 1.10)<br>I2 55%	RR 0.58<br>(0.33, 1.02)<br>I2 2%	RR 0.66<br>(0.53, 0.82)<br>I2 1%	MD -0.75<br>[-1.16, -0.34]<br>I2 83%	MD -0.87<br>[-1.38, -0.36]<br>I2 77%
**Loop Diuretics**	RR 1.28<br>(0.15, 11.28)<br>I2 29%	RR 1.20<br>(0.60, 2.41)<br>I2 0%	RR 4.61<br>(0.56, 37.82)<br>I2 0%	MD 1.88<br>[-0.08, 3.84]<br>I2 100%	MD -22.10<br>[-42.92, -1.28]<br>I2 0%	MD -28.00<br>[-56.55, 0.55]<br>I2 NA	NA	N/A	N/A	RR 2.73<br>(0.31, 23.88)<br>I2 0%	RR 0.70<br>(0.32, 1.54)<br>I2 N/A	RR 0.20<br>(0.01, 3.97)<br>I2 N/A	RR 1.06<br>(0.16, 7.05)<br>I2 0%	MD -0.70<br>[-3.40, 2.00]<br>I2 NA	MD 0.82<br>[0.11, 1.52]<br>I2 0%
**N-Acetylcysteine**	RR 0.85<br>(0.46, 1.59)<br>I2 0%	RR 0.89<br>(0.74, 1.08)<br>I2 8%	RR 0.68<br>(0.52, 0.89)<br>I2 0%	MD -0.15<br>[-0.57, 0.26]<br>I2 94%	MD -1.43<br>[-16.87, 14.01]<br>I2 0%	MD 5.58<br>[-2.19, 13.34]<br>I2 19%	N/A	MD 93.77<br>[31.75, 155.79]<br>I2 0%	RR 1.48<br>(0.91, 2.40)<br>I2 2%	RR 0.75<br>(0.31, 1.82)<br>I2 5%	RR 0.96<br>(0.89, 1.04)<br>I2 0%	RR 0.70<br>(0.27, 1.84)<br>I2 0%	RR 1.23<br>(0.71, 2.13)<br>I2 0%	MD -0.37<br>[-1.15, 0.41]<br>I2 91%	MD 0.35<br>[-0.16, 0.87]<br>I2 0%
**Corticoteroid Therapy**	RR 0.92<br>(0.57, 1.49)<br>N/A	RR 0.92<br>(0.65, 1.30)<br>I2 59%	RR 0.90<br>(0.72, 1.12)<br>I2 N/A	N/A	N/A	MD -4.78<br>[-12.08, 2.52]<br>I2 NA	N/A	N/A	N/A	RR 0.90<br>(0.57, 1.42)<br>I2 N/A	RR 3.50<br>(1.39, 8.80)<br>I2 0%	RR 0.91<br>(0.55, 1.50)<br>I2 N/A	RR 0.64<br>(0.54, 0.75)<br>I2 0%	MD 0.00<br>[-0.01, 0.01]<br>I2 0%	MD -0.07<br>[-3.27, 3.14]<br>I2 49%
**Osmotic Diuretics**	RR 2.81<br>(0.62, 12.80)<br>I2 0%	RR 0.90<br>(0.14, 5.69)<br>I2 0%	RR 0.90<br>(0.14, 5.69)<br>I2 0%	MD -0.02<br>[-0.25, 0.22]<br>I2 43%	MD -5.98<br>[-13.42, 1.47]<br>I2 0%	MD 1.00<br>[-6.17, 8.17]<br>I2 NA	MD 6.30<br>[-0.14, 12.74]<br>I2 NA	MD 92.73<br>[-11.26, 196.73]<br>I2 0%	N/A	RR 4.38<br>(0.23, 83.62)<br>I2 N/A	RR 2.62<br>(0.12, 59.40)<br>I2 N/A	N/A	RR 0.80<br>(0.13, 5.04)<br>I2 0%	MD -0.20<br>[-1.76, 1.37]<br>I2 98%	MD 0.30<br>[-0.08, 0.68]<br>I2 NA
**Sodium Bicarbonate**	RR 1.15<br>(0.62, 2.17)<br>I2 18%	RR 0.91<br>(0.72, 1.16)<br>I2 54%	RR 0.76<br>(0.39, 1.49)<br>I2 0%	MD -0.02<br>[-0.26, 0.21]<br>I2 75%	MD 0.00<br>[-29.80, 29.80]<br>I2 NA	MD -18.00<br>[-46.00, 10.00]<br>I2 NA	MD -15.80<br>[-47.63, 16.03]<br>I2 NA	MD -5.65<br>[-112.08, 100.78]<br>I2 50%	N/A	RR 1.30<br>(0.08, 20.11)<br>I2 36%	RR 1.07<br>(0.88, 1.31)<br>I2 0%	RR 0.14<br>(0.01, 2.67)<br>I2 N/A	RR 0.61<br>(0.08, 4.80)<br>I2 0%	MD 0.11<br>[-0.02, 0.23]<br>I2 0%	MD 0.23<br>[-0.29, 0.74]<br>I2 0%
**Volatile vs Propofol Anaesthesia**	RR 1.11<br>(0.70, 1.76)<br>N/A	RR 1.68<br>(0.45, 6.24)<br>I2 89%	RR 1.19<br>(0.56, 2.53)<br>I2 0%	MD 0.08<br>[-0.08, 0.24]<br>I2 46%	N/A	N/A	N/A	MD 66.00<br>[-25.97, 157.97]<br>I2 NA	RR 1.16<br>[0.59, 2.26]<br>I2 0%	RR 0.98<br>(0.69, 1.39)<br>I2 0%	RR 1.0<br>(0.71, 1.39)<br>I2 0%	RR 1.39<br>(0.72, 2.69)<br>I2 N/A	RR 1.00<br>(0.14, 6.96)<br>I2 N/A	MD 0.19<br>[-0.28, 0.67]<br>I2 77%	MD 0.10<br>[-2.20, 2.40]<br>I2 98%
**Miscellaneous Interventions**	RR 0.82<br>(0.47, 1.44)<br>I2 0%	RR 0.97<br>(0.74, 1.26)<br>I2 26%	RR 1.01<br>(0.55, 1.83)<br>I2 0%	MD 0.04<br>[-0.09, 0.18]<br>I2 57%	MD 15.26<br>[0.33, 30.18]<br>I2 46%	MD 29.21<br>[12.12, 46.30]<br>I2 0%	N/A	MD -17.22<br>[-47.99, 13.55]<br>I2 92%	RR 1.12<br>[0.76, 1.67]<br>I2 0%	RR 0.89<br>(0.51, 1.54)<br>I2 19%	RR 0.88<br>(0.73, 1.06)<br>I2 45%	RR 0.43<br>(0.18, 1.02)<br>I2 0%	RR 0.74<br>(0.33, 1.66)<br>I2 0%	MD -0.38<br>[-0.83, 0.06]<br>I2 90%	MD -0.36<br>[-0.95, 0.23]<br>I2 87%
**Miscellaneous Anti-oxidant**	RR 0.88<br>(0.15, 5.26)<br>I2 0%	RR 1.68<br>(0.86, 3.28)<br>I2 0%	RR 1.14<br>(0.46, 2.82)<br>I2 0%	MD 0.60<br>[0.33, 0.87]<br>I2 NA	MD 12.39<br>[-13.77, 38.54]<br>I2 88%	MD 34.00<br>[18.59, 49.41]<br>I2 NA	N/A	MD 17.00<br>[-77.25, 111.25]<br>I2 NA	N/A	N/A	RR 0.21<br>(0.04, 1.18)<br>I2 0%	N/A	N/A	MD -1.27<br>[-1.85, -0.68]<br>I2 0%	MD -0.07<br>[-0.64, 0.50]<br>I2 0%

eTable3: Summary of results for all included studies. Colour codes indicate direction of treatment effect on that outcome. Red indicating a harmful effect and green indicating a beneficial effect.

(green)	Statistically significant beneficial effect in intervention group.
(red)	Statistically significant harmful effect in intervention group.
(grey)	Statistically significant effect, however only reportedby one study.
(white)	No statistically significant effect.

Cardiac Surgery Only	30 Day Mortality	Risk of AKI	Renal Replacement Therapy	Urine output 24 hours	Creatinine Clearance (24H)	Creatinine Clearance (2-4 days)	Creatinine Clearance (5-7 days)	Perioperative Blood Loss	Adverse Reaction	Myocardial Infarction	Low Cardiac Output	Acute Brain Injury/Stroke	sepsis / infection	ICU LOS	Hospital LOS
**Atrial Natriuretic Peptide**	RR 0.64 (0.41, 0.99) I2 0%	RR 0.43 (0.33, 0.58) I2 0%	RR 0.26 [0.15, 0.47] I2 0%	MD 0.41 [-0.55, 1.37] I2 99%	MD 12.08 [-26.64, 50.81] I2 100%	MD 12.89 [-11.92, 37.71] I2 100%	MD 11.29 [-8.35, 30.93] I2 95%	MD 162.00 [-454.97, 778.97] I2 NA	RR 0.95 (0.61, 1.48) I2 30%	RR 0.98 [0.31, 3.10] I2 0%	RR 1.00 [0.85, 1.16] I2 3%	RR 0.59 [0.20, 1.80] I2 0%	RR 3.36 [0.81, 13.88] I2 0%	MD -0.47 [-1.31, 0.37] I2 50%	MD -2.66 [-3.83, -1.50] I2 61%
**Alpha 2 agonist**	RR 0.47 (0.17, 1.31) I2 0%	RR 0.61 (0.37, 1.01) I2 48%	RR 0.60 [0.35, 1.06] I2 13%	MD 0.09 [-0.07, 0.25] I2 92%	MD 10.67 [2.57, 18.77] I2 71%	MD 3.37 [-1.00, 7.73] I2 33%	MD -25.75 [-51.54, 0.04] I2 96%	MD -14.32 [-57.64, 28.99] I2 7%	N/A	RR 1.00 [0.07, 15.12] I2 NA	RR 0.87 [0.61, 1.25] I2 57%	RR 0.54 [0.21, 1.36] I2 5%	RR 0.42 [0.12, 1.44] I2 0%	MD 0.01 [-0.19, 0.21] I2 88%	MD -0.55 [-1.08, -0.02] I2 51%
**Inodilators**	RR 0.71 (0.53, 0.94) I2 0%	RR 0.65 (0.50, 0.85) I2 0%	RR 0.63 [0.46, 0.85] I2 0%	MD -0.21 [-0.56, 0.14] I2 NA	N/A	N/A	N/A	N/A	RR 5 (0.59, 42.34) N/A	RR 0.85 [0.52, 1.40] I2 40%	RR 0.99 [0.80, 1.21] I2 61%	RR 1.11 [0.76, 1.62] I2 0%	RR 0.77 [0.45, 1.31] I2 34%	MD -0.32 [-1.20, 0.56] I2 92%	MD 0.69 [-1.38, 2.75] I2 93%
**Vasopressor**	N/A	N/A	NA	MD -0.35 [-0.80, 0.10] I2 NA	MD -36.20 [-84.25, 11.85] I2 NA	N/A	MD 1.40 [-22.68, 25.48] I2 NA	N/A	N/A	NA	NA	NA	NA	NA	NA
**Calcium Channel Blocker**	N/A	N/A	RR 0.14 [0.01, 2.45] I2 NA	MD -0.36 [-1.69, 0.97] I2 90%	MD -5.41 [-27.39, 16.57] I2 93%	MD 41.70 [-12.09, 95.49] I2 NA	MD 22.30 [-16.37, 60.97] I2 NA	MD -193.31 [-412.53, 25.91] I2 0%	N/A	RR 0.33 [0.04, 2.98] I2 0%	RR 0.51 [0.20, 1.32] I2 0%	RR 1.00 [0.07, 14.21] I2 NA	NA	NA	NA
**Nitric Oxide Donor**	RR 0.38 (0.10, 1.49) I2 0%	RR 0.73 (0.58, 0.92) I2 N/A	RR 0.68 [0.20, 2.34] I2 NA	MD 0.16 [-0.01, 0.33] I2 NA	MD 6.60 [2.41, 10.79] I2 NA	MD 5.90 [2.04, 9.76] I2 NA	MD 3.10 [-0.81, 7.01] I2 NA	N/A	N/A	NA	NA	RR 0.31 [0.01, 7.58] I2 NA	RR 0.94 [0.06, 14.78] I2 NA	MD 0.00 [-0.32, 0.32] I2 NA	MD 0.00 [-0.64, 0.64] I2 NA
**Statin**	RR 3.63 (1.01, 13.03) I2 0%	RR 1.19 (0.96, 1.48) I2 42%	RR 1.22 [0.68, 2.18] I2 0%	MD -0.01 [-0.32, 0.30] I2 NA	N/A	N/A	N/A	N/A	RR 1.28 (0.60, 2.72) I2 0%	NA	RR 0.93 [0.71, 1.22] I2 0%	RR 1.19 [0.61, 2.30] I2 0%	RR 0.70 [0.37, 1.34] I2 59%	MD 0.09 [-0.11, 0.29] I2 34%	MD 0.03 [-0.90, 0.96] I2 49%
**Other Diuretics (Spironolactone)**	RR 0.83 (0.15, 4.75) I2 34%	RR 1.49 (1.05, 2.14) I2 N/A	RR 1.14 [0.46, 2.87] I2 NA	N/A	N/A	N/A	N/A	MD -18.00 [-90.93, 54.93] I2 NA	RR 0.62 (0.17, 2.25) I2 N/A	NA	RR 1.86 [0.64, 5.42] I2 NA	NA	RR 0.33 [0.06, 1.94] I2 NA	MD -1.00 [-1.55, -0.45] I2 NA	MD -1.00 [-2.92, 0.92] I2 NA
**Restrictive vs Liberal**	RR 1.30 (0.40, 4.22) I2 43%	RR 1.56 (0.90, 2.69) I2 0%	RR 0.92 [0.69, 1.22] I2 0%	N/A	N/A	N/A	N/A	MD 64.30 [-62.49, 191.09] I2 NA	N/A	NA	RR 2.00 [0.40, 9.95] I2 NA	RR 1.56 [0.44, 5.50] I2 29%	RR 15.00 [0.90, 249.30] I2 NA	NA	MD 2.00 [-2.96, 6.96] I2 NA
**ACE Inhibitor**	RR 1.91 (0.31, 11.63) I2 0%	RR 0.74 (0.23, 2.36) N/A	RR 1.82 [0.08, 42.22] I2 NA	MD 0.97 [0.24, 1.70] I2 NA	NA	N/A	N/A	MD 33.00 [4.90, 61.10] I2 NA	RR 0.75 (0.22, 2.56) i2 N/A	RR 0.50 [0.03, 7.83] I2 NA	RR 1.12 [0.72, 1.74] I2 26%	RR 0.59 [0.04, 8.83] I2 NA	RR 0.66 [0.15, 2.88] I2 NA	NA	MD -1.39 [-1.72, -1.06] I2 0%
**Dopamine Agonist**	RR 1.08 (0.82, 1.42) I2 0%	RR 0.81 (0.54, 1.21) I2 29%	RR 0.88 [0.66, 1.18] I2 0%	MD 0.16 [-0.25, 0.57] I2 85%	MD 24.54 [15.94, 33.14] I2 74%	MD 11.26 [-6.99, 29.51] I2 96%	MD -2.17 [-31.42, 27.08] I2 43%	MD 70.00 [-172.18, 312.18] I2 NA	N/A	RR 2.44 [0.12, 48.62] I2 NA	RR 1.07 [0.93, 1.23] I2 15%	RR 0.56 [0.09, 3.47] I2 29%	RR 0.82 [0.25, 2.70] I2 22%	MD -0.06 [-0.55, 0.44] I2 0%	MD 0.09 [-1.21, 1.39] I2 0%
**Erythropoeitin**	RR 0.35 (0.07, 1.71) I2 0%	RR 0.64 (0.38, 1.08) I2 33%	RR 0.49 [0.15, 1.60] I2 10%	MD 0.06 [-0.37, 0.49] I2 0%	NA	N/A	N/A	MD 200.00 [40.20, 359.80] I2 NA	N/A	RR 5.00 [0.25, 100.53] I2 NA	RR 2.18 [0.02, 241.99] I2 81%	RR 3.00 [0.13, 71.22] I2 NA	RR 0.62 [0.08, 4.90] I2 0%	MD -0.32 [-0.75, 0.12] I2 0%	MD 0.05 [-1.24, 1.34] I2 0%

**Glycaemic Control**	RR 0.61<br>(0.15, 2.51)<br>I2 0%	RR 0.34<br>(0.11, 1.04)<br>I2 0%	RR 0.49<br>[0.09, 2.50]<br>I2 NA	N/A	N/A	N/A	N/A	N/A	N/A	RR 0.76<br>[0.18, 3.27]<br>I2 NA	RR 0.63<br>[0.45, 0.88]<br>I2 NA	RR 0.72<br>[0.24, 2.21]<br>I2 0%	RR 0.39<br>[0.16, 0.98]<br>I2 61%	NA	NA
**Goal Directed Therapy**	RR 0.42<br>(0.16, 1.11)<br>I2 0%	RR 0.84<br>(0.55, 1.28)<br>I2 58%	RR 1.08<br>[0.28, 4.21]<br>I2 39%	MD -0.14<br>[-0.40, 0.12]<br>I2 57%	N/A	N/A	N/A	MD -58.90<br>[-147.08, 29.29]<br>I2 0%	N/A	RR 0.45<br>[0.18, 1.12]<br>I2 0%	RR 0.72<br>[0.18, 2.92]<br>I2 80%	RR 0.27<br>[0.09, 0.85]<br>I2 0%	RR 0.41<br>[0.21, 0.81]<br>I2 0%	MD -1.14<br>[-2.16, -0.12]<br>I2 93%	MD -1.25<br>[-2.13, -0.36]<br>I2 76%
**Loop Diuretics**	RR 1.05<br>(0.22, 5.07)<br>I2 0%	RR 1.24<br>(0.59, 2.61)<br>I2 0%	RR 4.61<br>[0.56, 37.82]<br>I2 0%	MD 1.18<br>[-1.40, 3.76]<br>I2 98%	MD -22.10<br>[-42.92, -1.28]<br>I2 0%	MD -28.00<br>[-56.55, 0.55]<br>I2 NA	MD 1.00<br>[-30.92, 32.92]<br>I2 NA	N/A	N/A	NA	RR 0.70<br>[0.32, 1.54]<br>I2 NA	RR 0.20<br>[0.01, 3.97]<br>I2 NA	RR 0.33<br>[0.01, 7.81]<br>I2 NA	MD -0.70<br>[-3.40, 2.00]<br>I2 NA	MD 4.90<br>[-5.95, 15.75]<br>I2 NA
**N-Acetylcysteine**	RR 0.81<br>(0.43, 1.53)<br>I2 0%	RR 0.89<br>(0.74, 1.08)<br>I2 8%	RR 0.68<br>[0.52, 0.89]<br>I2 0%	MD -0.04<br>[-0.31, 0.22]<br>I2 76%	MD -1.43<br>[-16.87, 14.01]<br>I2 0%	MD 5.58<br>[-2.19, 13.34]<br>I2 19%	N/A	MD 93.77<br>[31.75, 155.79]<br>I2 0%	RR 1.48<br>(0.91, 2.40)<br>I2 2%	NA	RR 0.96<br>[0.89, 1.04]<br>I2 0%	RR 0.70<br>[0.27, 1.84]<br>I2 0%	RR 1.23<br>[0.71, 2.13]<br>I2 0%	MD -0.48<br>[-1.22, 0.26]<br>I2 89%	MD 0.10<br>[-0.43, 0.62]<br>I2 28%
**Corticosteroid Therapy**	RR 0.92<br>(0.57, 1.49)<br>I2 N/A	RR 0.92<br>(0.65, 1.30)<br>I2 59%	RR 0.90<br>[0.72, 1.12]<br>I2 NA	N/A	N/A	N/A	N/A	N/A	N/A	NA	RR 3.50<br>[1.39, 8.80]<br>I2 0%	RR 0.91<br>[0.55, 1.50]<br>I2 NA	RR 0.64<br>[0.54, 0.75]<br>I2 0%	MD 0.00<br>[-0.01, 0.01]<br>I2 0%	MD -1.00<br>[-1.26, -0.74]<br>I2 NA
**Osmotic Diuretics**	N/A	N/A	NA	MD -0.12<br>[-0.27, 0.03]<br>I2 0%	MD -5.70<br>[-32.52, 21.12]<br>I2 NA	N/A	N/A	MD 110.00<br>[0.43, 219.57]<br>I2 NA	N/A	NA	NA	NA	NA	MD -1.00<br>[-1.34, -0.66]<br>I2 NA	NA
**Sodium Bicarbonate**	RR 1.09<br>(0.54, 2.18)<br>I2 28%	RR 0.97<br>(0.79, 1.19)<br>I2 40%	RR 0.71<br>[0.36, 1.42]<br>I2 0%	MD -0.02<br>[-0.26, 0.21]<br>I2 75%	MD 0.00<br>[-29.80, 29.80]<br>I2 NA	MD -18.00<br>[-46.00, 10.00]<br>I2 NA	MD -15.80<br>[-47.63, 16.03]<br>I2 NA	MD -5.65<br>[-112.08, 100.78]<br>I2 50%	N/A	NA	RR 1.08<br>[0.88, 1.31]<br>I2 0%	RR 0.14<br>[0.01, 2.67]<br>I2 NA	RR 1.00<br>[0.07, 15.36]<br>I2 NA	MD 0.11<br>[-0.02, 0.23]<br>I2 0%	MD 0.55<br>[-0.26, 1.36]<br>I2 0%
**Volatile vs Propofol Anaesthesia**	RR 1.11<br>(0.70, 1.76)<br>I2 N/A	RR 1.68<br>(0.45, 6.24)<br>I2 89%	RR 1.19<br>[0.56, 2.53]<br>I2 0%	MD -0.02<br>[-0.22, 0.18]<br>I2 NA	N/A	N/A	N/A	N/A	RR 1.16<br>(0.59, 2.26)<br>I2 0%	RR 0.98<br>(0.69, 1.39)<br>I2 0%	RR 0.66<br>[0.19, 2.34]<br>I2 NA	RR 1.39<br>[0.72, 2.69]<br>I2 NA	NA	MD 0.19<br>[-0.28, 0.67]<br>I2 77%	MD -1.69<br>[-5.02, 1.64]<br>I2 99%
**Miscellaneous Interventions**	RR 0.73<br>(0.38, 1.39)<br>I2 0%	RR 0.85<br>(0.68, 1.05)<br>I2 0%	RR 0.97<br>[0.53, 1.78]<br>I2 0%	MD 0.01<br>[-0.13, 0.16]<br>I2 60%	MD 15.26<br>[0.33, 30.18]<br>I2 46%	MD 29.21<br>[12.12, 46.30]<br>I2 0%	N/A	MD -20.90<br>[-98.80, 57.00]<br>I2 91%	RR 0.84<br>(0.43, 1.65)<br>I2 0%	NA	RR 0.88<br>[0.73, 1.06]<br>I2 45%	RR 0.42<br>[0.18, 0.97]<br>I2 0%	RR 0.87<br>[0.35, 2.17]<br>I2 0%	MD -0.48<br>[-0.84, -0.12]<br>I2 85%	MD -1.17<br>[-2.02, -0.32]<br>I2 92%
**Miscellaneous Anti-oxidant**	RR 1.34<br>(0.15, 11.81)<br>I2 0%	RR 1.68<br>(0.86, 3.28)<br>I2 0%	RR 1.14<br>[0.46, 2.82]<br>I2 0%	MD 0.60<br>[0.33, 0.87]<br>I2 NA	MD 12.39<br>[-13.77, 38.54]<br>I2 88%	N/A	N/A	MD 17.00<br>[-77.25, 111.25]<br>I2 NA	N/A	NA	RR 0.17<br>[0.02, 1.30]<br>I2 NA	NA	NA	MD -1.27<br>[-1.85, -0.68]<br>I2 0%	MD -0.07<br>[-0.64, 0.50]<br>I2 0%

eTable4: Summary of results for all studies in the cardiac surgery subgroup. Colour codes indicate direction of treatment effect on that outcome. Red indicating a harmful effect and green indicating a beneficial effect.

(green)	Statistically significant beneficial effect in intervention group.
(red)	Statistically significant harmful effect in intervention group.
(grey)	Statistically significant effect, however only reportedby one study.
(white)	No statistically significant effect.

General Surgery Only	30 Day Mortality	Risk of AKI	Renal Replacement Therapy	Urine output 24 Hr	Creatinine Clearance (24H)	Creatinine Clearance (2-4 days)	Creatinine Clearance (5-7 days)	Perioperative Blood Loss	Adverse Reaction	Myocardial Infarction	Low Cardiac Output	Acute Brain Injury/Stroke	sepsis / infection	ICU LOS	Hospital LOS
**Atrial Natriuretic Peptide**	N/A	N/A	N/A	N/A	N/A	N/A	N/A	N/A	N/A	N/A	N/A	N/A	N/A	N/A	N/A
**Alpha 2 agonist**	RR 0.59 (0.14, 2.46) I2 N/A	RR 0.40 (0.09, 1.81) I2 N/A	N/A	N/A	N/A	N/A	N/A	MD -92.74 [-407.58, 222.10] I2 0%	N/A	RR 0.49 [0.05, 5.42] I2 NA	RR 0.57 [0.13, 2.52] I2 0%	RR 0.22 [0.05, 1.01] I2 NA	RR 0.46 [0.18, 1.18] I2 NA	MD -1.00 [-1.45, -0.55] I2 NA	MD -0.93 [-1.70, -0.16] I2 20%
**Inodilators**	N/A	N/A	N/A	N/A	N/A	N/A	N/A	N/A	N/A	N/A	N/A	N/A	N/A	N/A	N/A
**Vasopressor**	RR 0.92 (0.26,3.23) I2 0%	RR 0.49 (0.25, 0.96) I2 0%	N/A	N/A	N/A	N/A	N/A	MD -223.70 [-321.29, -126.11] I2 NA	N/A	N/A	RR 0.33 [0.04, 2.99] I2 NA	RR 1.68 [0.07, 40.94] I2 NA	RR 0.98 [0.29, 3.27] I2 NA	MD -1.00 [-1.83, -0.17] I2 NA	N/A
**Calcium Channel Blocker**	N/A	N/A	N/A	N/A	N/A	N/A	N/A	N/A	N/A	N/A	N/A	N/A	N/A	N/A	N/A
**Nitric Oxide Donor**	N/A	N/A	N/A	N/A	N/A	N/A	N/A	N/A	N/A	N/A	N/A	N/A	N/A	N/A	N/A
**Statin**	N/A	N/A	N/A	N/A	N/A	N/A	N/A	N/A	N/A	N/A	N/A	N/A	N/A	N/A	N/A
**Other Diuretics (Spironolactone)**	N/A	N/A	N/A	N/A	N/A	N/A	N/A	N/A	N/A	N/A	N/A	N/A	N/A	N/A	N/A
**Restrictive vs Liberal**	RR 0.96 (0.73, 1.25) I2 0%	RR 1.66 (1.27, 2.18) I2 0%	RR 2.23 [0.97, 5.14] I2 0%	MD -0.65 [-0.78, -0.52] I2 NA	N/A	N/A	N/A	MD -0.04 [-11.06, 10.99] I2 29%	RR 0.68 [0.12, 3.93] I2 NA	RR 3.21 [0.34, 30.58] I2 0%	RR 1.03 [0.45, 2.38] I2 31%	RR 0.85 [0.21, 3.54] I2 0%	RR 1.16 [1.03, 1.29] I2 0%	MD 0.40 [0.30, 0.50] I2 NA	MD 0.59 [-0.28, 1.46] I2 49%
**ACE Inhibitor**	N/A	N/A	N/A	N/A	N/A	N/A	N/A	N/A	N/A	N/A	N/A	N/A	N/A	N/A	N/A
**Dopamine Agonist**	N/A	N/A	N/A	MD 0.50 [-0.18, 1.18] I2 58%	MD 15.01 [5.38, 24.64] I2 0%	MD 10.23 [-2.75, 23.22] I2 0%	MD 10.04 [7.18, 12.90] I2 0%	N/A	N/A	N/A	N/A	N/A	N/A	N/A	N/A
**Erythropoeitin**	N/A	N/A	N/A	N/A	N/A	N/A	N/A	N/A	N/A	N/A	N/A	N/A	N/A	N/A	N/A
**Glycaemic Control**	N/A	N/A	N/A	N/A	N/A	N/A	N/A	N/A	N/A	N/A	N/A	N/A	N/A	N/A	N/A
**Goal Directed Therapy**	RR 1.01 (0.68, 1.50) I2 0%	RR 1.14 (0.92, 1.41) I2 0%	RR 1.19 [0.57, 2.48] I2 0%	MD -0.30 [-1.54, 0.94] I2 94%	N/A	N/A	N/A	MD -37.79 [-82.03, 6.46] I2 53%	RR 1.00 [0.74, 1.35] I2 NA	RR 0.83 [0.19, 3.68] I2 18%	RR 0.97 [0.65, 1.46] I2 67%	RR 1.52 [0.39, 5.90] I2 0%	RR 0.78 [0.58, 1.05] I2 0%	MD -0.70 [-1.24, -0.17] I2 68%	MD -0.95 [-2.05, 0.15] I2 86%

**Loop Diuretics**	N/A	RR 0.98 (0.14, 6.76) I2 N/A	N/A	N/A	N/A	N/A	N/A	N/A	N/A	N/A	N/A	N/A	RR 1.97 [0.18, 21.14] I2 NA	N/A	MD 0.80 [0.08, 1.52] I2 NA
**N-Acetylcysteine**	N/A	N/A	N/A	N/A	N/A	N/A	N/A	N/A	N/A	N/A	N/A	N/A	N/A	N/A	N/A
**Corticosteroid Therapy**	N/A	N/A	N/A	N/A	N/A	N/A	N/A	N/A	N/A	N/A	N/A	N/A	N/A	N/A	N/A
**Osmotic Diuretics**	RR 4.12 (0.54, 31.26) I2N/A	RR 1.65 (0.17, 16.33) I2 N/A	RR 1.65 [0.17, 16.33] I2 NA	N/A	N/A	N/A	N/A	N/A	N/A	N/A	N/A	N/A	N/A	N/A	N/A
**Sodium Bicarbonate**	N/A	N/A	N/A	N/A	N/A	N/A	N/A	N/A	N/A	N/A	N/A	N/A	N/A	N/A	N/A
**Volatile vs Propofol Anaesthesia**	N/A	N/A	N/A	MD 0.15 [0.01, 0.29] I2 NA	N/A	N/A	N/A	MD 66.00 [-25.97, 157.97] I2 NA	N/A	N/A	RR 1.03 [0.73, 1.45] I2 NA	N/A	RR 1.00 [0.14, 6.96] I2 NA	N/A	MD 0.80 [-0.42, 2.02] I2 NA
**Miscellaneous Interventions**	RR 2.00 (0.20, 20.33) I2 N/A	N/A	RR 3.00 [0.13, 69.52] I2 NA	N/A	N/A	N/A	N/A	MD 3.82 [-1.41, 9.05] I2 NA	N/A	N/A	N/A	N/A	RR 0.40 [0.09, 1.83] I2 NA	N/A	N/A
**Miscellaneous Anti-oxidant**	N/A	N/A	N/A	N/A	N/A	N/A	N/A	N/A	N/A	N/A	N/A	N/A	N/A	N/A	N/A

eTable5: Summary of results for all studies in the general surgery subgroup. Colour codes indicate direction of treatment effect on that outcome. Red indicating a harmful effect and green indicating a beneficial effect.

(green)	Statistically significant beneficial effect in intervention group.
(red)	Statistically significant harmful effect in intervention group.
(grey)	Statistically significant effect, however only reportedby one study.
(white)	No statistically significant effect.

Vascular Surgery	30 Day Mortality	Risk of AKI	Renal Replacement Therapy	Urine output 24 hours	Creatinine Clearance (24H)	Creatinine Clearance (2-4 days)	Creatinine Clearance (5-7 days)	Perioperative Blood Loss	Adverse Reaction	Myocardial Infarction	Low Cardiac Output	Acute Brain Injury/Stroke	sepsis / infection	ICU LOS	Hospital LOS
**Atrial Natriuretic Peptide**	RR 0.33<br>[0.01, 7.81]<br>I2 NA	RR 0.38<br>[0.18, 0.80]<br>I2 NA	N/A	MD 0.63<br>[-0.48, 1.74]<br>I2 85%	MD 33.55<br>[-3.41, 70.51]<br>I2 NA	MD 47.47<br>[9.88, 85.05]<br>I2 NA	N/A	N/A	N/A	N/A	RR 0.83<br>[0.67, 1.03]<br>I2 NA	N/A	N/A	MD 1.30<br>[0.41, 2.19]<br>I2 NA	RR -1.80<br>[-6.00, 2.40]<br>I2 NA
**Alpha 2 agonist**	RR 0.33<br>[0.01, 8.05]<br>I2 NA	RR 0.67<br>[0.20, 2.27]<br>I2 NA	N/A	N/A	N/A	N/A	N/A	N/A	N/A	RR 0.33<br>[0.04, 3.13]<br>I2 NA	RR 0.24<br>[0.09, 0.60]<br>I2 NA	N/A	N/A	N/A	NA
**Inodilators**	N/A	N/A	N/A	N/A	N/A	N/A	N/A	N/A	N/A	N/A	N/A	N/A	N/A	N/A	NA
**Vasopressor**	N/A	N/A	N/A	N/A	N/A	N/A	N/A	N/A	N/A	N/A	N/A	N/A	N/A	N/A	NA
**Calcium Channel Blocker**	N/A	N/A	N/A	MD -0.12<br>[-1.08, 0.84]<br>I2 NA	MD 14.25<br>[7.48, 21.02]<br>I2 NA	N/A	N/A	N/A	N/A	N/A	N/A	N/A	N/A	N/A	NA
**Nitric Oxide Donor**	N/A	N/A	N/A	N/A	N/A	N/A	N/A	N/A	N/A	N/A	N/A	N/A	N/A	N/A	NA
**Statin**	N/A	N/A	N/A	N/A	N/A	N/A	N/A	N/A	N/A	N/A	N/A	N/A	N/A	N/A	NA
**Other Diuretics (Spironolactone)**	N/A	N/A	N/A	N/A	N/A	N/A	N/A	N/A	N/A	N/A	N/A	N/A	N/A	N/A	NA
**Restrictive vs Liberal**	RR 0.33<br>[0.01, 7.87]<br>I2 NA	N/A	N/A	N/A	N/A	N/A	N/A	N/A	N/A	N/A	N/A	N/A	RR 0.14<br>[0.01, 2.65]<br>I2 NA	N/A	NA
**ACE Inhibitor**	N/A	N/A	N/A	MD 0.10<br>[-0.29, 0.49]<br>I2 NA	MD 24.00<br>[5.15, 42.85]<br>I2 NA	N/A	N/A	N/A	N/A	N/A	N/A	N/A	N/A	N/A	NA
**Dopamine Agonist**	RR 0.33<br>[0.01, 7.45]<br>I2 NA	N/A	RR 0.28<br>[0.07, 1.13]<br>I2 NA	MD 0.00<br>[-0.42, 0.42]<br>I2 NA	MD 4.39<br>[-11.16, 19.94]<br>I2 0%	MD -5.84<br>[-44.72, 33.04]<br>I2 83%	MD -24.49<br>[-39.45, -9.54]<br>I2 0%	N/A	RR 3.00<br>[0.75, 12.00]<br>I2 NA	RR 0.33<br>[0.01, 7.45]<br>I2 NA	N/A	RR 0.33<br>[0.01, 7.45]<br>I2 NA	N/A	N/A	NA
**Erythropoeitin**	RR 0.97<br>[0.15, 6.46]<br>I2 NA	RR 0.97<br>[0.63, 1.49]<br>I2 NA	RR 0.32<br>[0.04, 2.94]<br>I2 NA	N/A	N/A	N/A	N/A	N/A	N/A	RR 2.91<br>[0.12, 68.81]<br>I2 NA	RR 0.16<br>[0.02, 1.26]<br>I2 NA	N/A	N/A	N/A	NA
**Glycaemic Control**	N/A	N/A	N/A	N/A	N/A	N/A	N/A	N/A	N/A	N/A	N/A	N/A	N/A	N/A	NA
**Goal Directed Therapy**	RR 0.99<br>[0.11, 9.23]<br>I2 0%	RR 1.00<br>[0.35, 2.83]<br>I2 0%	RR 1.00<br>[0.35, 2.83]<br>I2 0%	N/A	MD -10.00<br>[-38.06, 18.06]<br>I2 NA	N/A	MD -25.00<br>[-48.87, -1.13]<br>I2 NA	MD 151.16<br>[-185.50, 487.83]<br>I2 0%	N/A	RR 0.28<br>[0.05, 1.63]<br>I2 0%	N/A	RR 3.00<br>[0.13, 71.22]<br>I2 NA	RR 1.10<br>[0.53, 2.27]<br>I2 0%	MD 0.03<br>[-0.69, 0.75]<br>I2 0%	RR 0.34<br>[-0.96, 1.64]<br>I2 0%

**Loop Diuretics**	N/A	N/A	N/A	N/A	N/A	N/A	N/A	N/A	N/A	N/A	N/A	N/A	N/A	N/A	NA
**N-Acetylcysteine**	RR 3.09 [0.13, 73.21] I2 NA	N/A	N/A	MD 14.40 [14.20, 14.60] I2 NA	N/A	N/A	N/A	N/A	N/A	N/A	N/A	N/A	N/A	N/A	NA
**Corticosteroid Therapy**	N/A	N/A	N/A	N/A	N/A	N/A	N/A	N/A	N/A	N/A	N/A	N/A	N/A	MD 0.20 [-0.66, 1.06] I2 NA	RR 2.80 [-2.52, 8.12] I2 NA
**Osmotic Diuretics**	RR 1.73 [0.18, 16.99] I2 NA	RR 0.29 [0.01, 6.60] I2 NA	RR 0.29 [0.01, 6.60] I2 NA	MD 0.32 [-0.10, 0.73] I2 NA	MD -6.00 [-13.75, 1.75] I2 NA	MD 1.00 [-6.17, 8.17] I2 NA	MD 6.00 [-0.44, 12.44] I2 NA	N/A	N/A	RR 4.38 [0.23, 83.62] I2 NA	RR 2.62 [0.12, 59.40] I2 NA	N/A	RR 0.43 [0.04, 4.25] I2 NA	MD 0.60 [0.37, 0.83] I2 NA	RR 0.30 [-0.08, 0.68] I2 NA
**Sodium Bicarbonate**	RR 3.00 [0.13, 68.84] I2 NA	RR 0.19 [0.04, 0.78] I2 NA	RR 3.00 [0.13, 68.84] I2 NA	N/A	N/A	N/A	N/A	N/A	N/A	RR 0.31 [0.01, 7.35] I2 NA	RR 0.93 [0.21, 4.25] I2 NA	N/A	RR 0.31 [0.01, 7.35] I2 NA	N/A	RR 0.00 [-0.67, 0.67] I2 NA
**Volatile vs Propofol Anaesthesia**	N/A	N/A	N/A	N/A	N/A	N/A	N/A	N/A	N/A	N/A	N/A	N/A	N/A	N/A	NA
**Miscellaneous Interventions**	RR 0.99 [0.29, 3.40] I2 NA	RR 1.69 [1.11, 2.58] I2 NA	N/A	MD 0.21 [-0.08, 0.50] I2 NA	N/A	N/A	N/A	MD 5.80 [-5.34, 16.94] I2 NA	RR 1.38 [0.70, 2.70] I2 9%	N/A	N/A	N/A	N/A	N/A	RR 0.88 [-0.74, 2.50] I2 93%
**Miscellaneous Anti-oxidant**	RR 0.37 [0.02, 8.48] I2 NA	N/A	N/A	N/A	N/A	N/A	N/A	N/A	N/A	N/A	RR 0.37 [0.02, 8.48] I2 NA	N/A	N/A	N/A	NA

eTable6: Summary of results for all studies in the Vascular surgery subgroup. Colour codes indicate direction of treatment effect on that outcome. Red indicating a harmful effect and green indicating a beneficial effect.

(green)	Statistically significant beneficial effect in intervention group.
(red)	Statistically significant harmful effect in intervention group.
(grey)	Statistically significant effect, however only reportedby one study.
(white)	No statistically significant effect.

	Egger's Test	Co-Primary Outcomes	
Interventions	Intervention	30 day mortality	Risk of AKI
	Calcium Channel Blocker	No Outcomes	<10 studies
	Atrial Natriuretic Peptide	0.8819	<10 studies
	Inodilator	0.001112	<10 studies
	NO Donor	<10 studies	<10 studies
	Alpha 2 Agonist	<10 studies	0.05626
	Vasopressors	<10 studies	<10 studies

eTable7: Summary of results for Egger's test.

All Studies - Low risk of Allocation Concealment	30 Day Mortality	Risk of AKI	Renal Replacement Therapy	Urine output 24 hours	Creatinine Clearance (24H)	Creatinine Clearance (2-4 days)	Creatinine Clearance (5-7 days)	Perioperative Blood Loss	Adverse Reaction	Myocardial Infarction	Low Cardiac Output	Acute Brain Injury/Stroke	sepsis / infection	ICU LOS	Hospital LOS
**Atrial Natriuretic Peptide**	RR 0.59<br>(0.21, 1.64)<br>I2 0%	RR 0.40<br>(0.30, 0.54)<br>I2 0%	RR 0.28<br>(0.15, 0.52)<br>I2 0%	MD 1.12<br>(-0.28, 2.52)<br>I2 99%	MD 54.36<br>(27.83, 80.88)<br>I2 59%	MD 45.11<br>(44.39, 45.83)<br>I2 0%	MD 28.57<br>(27.88, 29.26)<br>I2 N/A	N/A	N/A	RR 3.06<br>(0.13, 74, 57)<br>I2 N/A	RR 0.82<br>(0.67, 1.01)<br>I2 0%	RR 0.58<br>(0.12, 2.72)<br>I2 0%	RR 2.35<br>(0.35, 15.93)<br>I2 0%	MD 0.30<br>(-0.59, 1.19)<br>I2 N/A	MD -2.33<br>(-3.95, -0.71)<br>I2 77%
**Alpha 2 agonist**	RR 0.45<br>(0.17, 1.18)<br>I2 0%	RR 0.71<br>(0.46, 1.11)<br>I2 71%	RR 0.95<br>(0.54, 1.65)<br>I2 53%	MD 0.17<br>(0.02, 0.32)<br>I2 54%	MD 15.61<br>(1.05, 30.17)<br>I2 86%	MD 1.34<br>(-3.15, 5.83)<br>I2 4%	MD -2.00<br>(-10.63, 6.63)<br>I2 N/A	MD-64.96<br>(-133.04, 3.11)<br>I2 0%	N/A	RR 1.00<br>(0.07, 15.12)<br>I2 N/A	RR 0.52<br>(0.23, 1.16)<br>I2 0%	RR 0.29<br>(0.11, 0.74)<br>I2 0%	RR 0.45<br>(0.21, 0.97)<br>I2 0%	MD -0.13<br>(-1.11, 0.85)<br>I2 97%	MD -0.87<br>(-2.19, 0.44)<br>I2 71%
**Inodilators**	RR 0.83<br>(0.61, 1.13)<br>I2 0%	RR 0.73<br>(0.53, 1.01)<br>I2 0%	RR 0.64<br>(0.45, 0.91)<br>I2 0%	N/A	N/A	N/A	N/A	N/A	RR 5.00<br>(0.59, 42.34)<br>I2 N/A	RR 1.02<br>(0.77, 1.35)<br>I2 0%	RR 1.02<br>(0.84, 1.24)<br>I2 56%	RR 0.93<br>(0.60, 1.46)<br>I2 23%	RR 0.90<br>(0.61, 1.34)<br>I2 0%	MD -0.23<br>(-0.49, 0.02)<br>I2 0%	MD -0.03<br>(-0.74, 0.68)<br>I2 0%
**Vasopressor**	RR 0.33<br>(0.01, 7.81)<br>N/A	RR 0.50<br>(0.05, 5.17)<br>I2 N/A	N/A	N/A	N/A	N/A	N/A	MD -223.70<br>(-321.29, -126.11)<br>I2 N/A	N/A	N/A	RR 0.33<br>(0.04, 2.99)<br>I2 N/A	N/A	N/A	N/A	N/A
**Calcium Channel Blocker**	N/A	N/A	N/A	N/A	N/A	N/A	N/A	N/A	N/A	N/A	N/A	N/A	N/A	N/A	N/A
**Nitric Oxide Donor**	RR 0.94<br>(0.06, 14.78)<br>I2 N/A	N/A	N/A	MD 0.16<br>(-0.01, 0.33)<br>I2 N/A	MD 6.60<br>(2.41, 10.79)<br>I2 N/A	MD 5.90<br>(2.04, 9.76)<br>I2 N/A	MD 3.10<br>(-0.81, 7.01)<br>I2 N/A	N/A	N/A	RR 0.70<br>(0.16, 3.07)<br>I2 N/A	N/A	RR 0.31<br>(0.01, 7.58)<br>N/A	RR 0.94<br>(0.06, 14.78)<br>I2 N/A	N/A	N/A
**Statin**	RR 5.00<br>(0.25, 101.58)<br>I2 N/A	RR 1.65<br>(1.04, 2.60)<br>I2 N/A	RR 0.75<br>(0.28, 2.00)<br>I2 N/A	N/A	N/A	N/A	N/A	N/A	RR 1.33<br>(0.50, 3.56)<br>N/A	N/A	RR 0.91<br>(0.66, 1.24)<br>I2 N/A	N/A	N/A	MD 0.59<br>(-0.02, 1.20)<br>I2 N/A	MD 1.50<br>(-0.43, 3.43)<br>I2 N/A
**Other Diuretics (Spironolactone)**	RR 1.36<br>(0.48, 3.79)<br>I2 N/A	RR 1.49<br>(1.05, 2.14)<br>I2 N/A	RR 1.14<br>(0.46, 2.87)<br>I2 N/A	N/A	N/A	N/A	N/A	MD -18.00<br>(-90.93, 54.93)<br>I2 N/A	N/A	N/A	N/A	N/A	N/A	MD -1.00<br>(-1.55, -0.45)<br>I2 N/A	N/A
**Restrictive vs Liberal**	RR 0.54<br>(0.16, 1.75)<br>I2 0%	RR 1.30<br>(0.50, 3.42)<br>I2 0%	RR 1.40<br>(0.40, 4.88)<br>I2 0%	MD -0.65<br>(-0.78, -0.52)<br>I2 N/A	N/A	N/A	N/A	MD 13.30<br>(-8.80, 34.86)<br>I2 35%	N/A	RR 3.21<br>(0.34, 30.58)<br>I2 0%	RR 1.29<br>(0.34, 4.90)<br>I2 44%	RR 0.83<br>(0.20, 3.44)<br>I2 0%	RR 0.64<br>(0.32, 1.27)<br>I2 0%	N/A	MD 0.00<br>(-0.99, 0.99)<br>I2 N/A
**ACE Inhibitor**	N/A	N/A	N/A	MD 0.10<br>(-0.29, 0.49)<br>I2 N/A	MD 24.00<br>(5.15, 42.85)<br>I2 N/A	N/A	N/A	N/A	N/A	N/A	N/A	N/A	N/A	N/A	N/A
**Dopamine Agonist**	RR 1.07<br>(0.81, 1.41)<br>I2 0%	RR 0.74<br>(0.36, 1.53)<br>I2 47%	RR 1.13<br>(1.02, 1.26)<br>I2 0%	MD 0.30<br>(-0.37, 0.97)<br>I2 87%	MD 11.05<br>(-25.43, 47.54)<br>I2 77%	MD 12.36<br>(-8.30, 33.20)<br>I2 97%	MD -5.58<br>(-50.82, 39.65)<br>I2 57%	MD 70.00<br>(-172.18, 312.18)<br>I2 N/A	N/A	RR 2.44<br>(0.12, 48.62)<br>I2 N/A	RR 1.03<br>(0.82, 1.30)<br>I2 70%	RR 1.03<br>(0.21, 5.05)<br>I2 N/A	RR 0.82<br>(0.25, 2.70)<br>I2 22%	MD -1.03<br>(-3.17, 1.11)<br>I2 0%	MD 0.33<br>(-2.19, 2.85)<br>I2 0%

**Erythropoeitin**	RR 0.64 (0.17, 2.45) I2 0%	RR 0.90 (0.65, 1.25) I2 0%	RR 0.42 (0.16, 1.08) I2 0%	MD 0.06 (-0.37, 0.49) I2 0%	N/A	N/A	N/A	MD 200.00 (40.20, 359, 80) I2 N/A	N/A	RR 3.87 (0.44, 34.13) I2 0%	RR 0.85 (0.03, 23.06) I2 79%	RR 3.00 (0.13, 71.22) I2 N/A	RR 0.62 (0.08, 4.90) I2 0%	MD -0.33 (-0.76, 0.11) I2 0%	MD 0.05 (-1.24, 1.34) I2 0%
**Glycaemic Control**	N/A	N/A	N/A	N/A	N/A	N/A	N/A	MD 41.80 (-182.07, 265,67) I2 N/A	N/A	N/A	RR 0.33 (0.05, 2.41) I2 N/A	N/A	RR 0.64 (0.03, 14.36) N/A	N/A	MD 16.80 (-40.74, 7.14) I2 N/A
**Goal Directed Therapy**	RR 0.48 (0.28, 0.81) I2 0%	RR 0.82 (0.63, 1.06) I2 25%	RR 0.83 (0.37, 1.86) I2 22%	MD -0.05 (-0.59, 0.49) I2 89%	N/A	N/A	N/A	MD -38.30 (-75.37, - 1.23) I2 77%	N/A	RR 0.54 (0.25, 1.16) I2 0%	RR 0.92 (0.76, 1.13) I2 57%	RR 0.74 (0.27, 2.05) I2 23%	RR 0.65 (0.51, 0.84) I2 0%	MD -1.04 (-1.69, -0.39) I2 91%	MD -0.90 (-1.43, -0.36) I2 76%
**Loop Diuretics**	RR 4.50 (0.25, 79.72) I2 N/A	RR 4.50 (0.25, 79,72) I2 N/A	RR 6.50 (0.38, 109.96) I2 N/A	MD -0.12 (-0.47, 0.23) I2 N/A	MD -30.00 (-60.73, 0.73) I2 N/A	MD -28.00 (-56.55, 0.55) I2 N/A	N/A	N/A	N/A	RR 2.50 (0.13, 49.76) I2 N/A	N/A	N/A	N/A	MD -0.70 (-3.40, 2.00) I2 N/A	MD 4.90 (-5.95, 15.75) I2 N/A
**N-Acetylcysteine**	RR 0.80 (0.24, 2.67) I2 30%	RR 1.02 (0.76, 1.36) I2 0%	RR 0.62 (0.46, 0.82) I2 0%	MD -0.33 (-0.82, 0.16) I2 95%	MD -1.43 (-16.87, 14.01) I2 N/A	MD 5.58 (-2.19, 13.34) I2 19%	N/A	MD 75.91 (9.65, 142.17) I2 0%	RR 1.71 (1.07, 2.76) I2 0%	RR 1.05 (0.30, 3.68) I2 22%	RR 0.87 (0.70, 1.09) I2 0%	RR 0.70 (0.27, 1.84) I2 0%	RR 1.99 (0.37, 10.68) I2 N/A	MD -0.39 (-1.49, 0.71) I2 94%	MD 0.36 (-0.22, 0.94) I2 0%
**Corticosteroid Therapy**	N/A	RR 1.03 (0.98, 1.09) I2 N/A	RR 0.90 (0.72, 1.12) I2 N/A	N/A	N/A	N/A	N/A	N/A	N/A	N/A	N/A	N/A	N/A	MD 0.20 (-0.66, 1.06) I2 N/A	MD 2.80 (-2.52, 8.12) I2 N/A
**Osmotic Diuretics**	N/A	N/A	N/A	N/A	N/A	N/A	N/A	N/A	N/A	N/A	N/A	N/A	N/A	N/A	N/A
**Sodium Bicarbonate**	RR 1.71 (0.86, 3.41) I2 0%	RR 1.01 (0.78, 1.30) I2 54%	RR 0.77 (0.35, 1.71) I2 0%	MD -0.11 (-0.22, -0.00) I2 0%	MD 0.00 (-29.80, 29.80) I2 N/A	MD -18.00 (-46.00, 10.00) I2 N/A	MD -15.80 (-47.63, 16.03) I2 N/A	MD -5.65 (-112.08, 100.78) I2 50%	N/A	RR 5.06 (0.24, 104.58) I2 N/A	RR 1.08 (0.88, 1.31) I2 0%	RR 0.14 (0.01, 2.67) I2 N/A	RR 1.00 (0.07, 15.36) I2 N/A	MD 0.08 (-0.05, 0.22) I2 0%	MD 0.13 (-0.42, 0.68) I2 0%
**Volatile vs Propofol Anaesthesia**	RR 1.11 (0.70, 1.76) I2 N/A	RR 1.68 (0.45, 6.24) I2 89%	RR 1.19 (0.56, 2.53) I2 0%	MD -0.02 (- 0.22, 0.18) I2 N/A	N/A	N/A	N/A	N/A	RR 1.79 (0.60, 5.33) I2 N/A	RR 0.96 (0.67, 1.37) I2 N/A	RR 0.93 (0.64, 1.37) I2 0%	RR 1.39 (0.72, 2.69) I2 N/A	N/A	MD 0.07 (-0.20, 0.34) I2 57%	MD 0.98 (-0.53, 2.48) I2 90%
**Miscellaneous Interventions**	RR 0.54 (0.22, 1.29) I2 0%	RR 1.43 (1.04, 1.96) I2 0%	RR 0.84 (0.39, 1.79) I2 0%	MD -0.21 (-0.46, 0.04) I2 N/A	N/A	N/A	N/A	MD -57.18 (-147.48, 33.11) I2 91%	N/A	RR 0.88 (0.34, 2.25) I2 31%	RR 0.83 (0.50, 1.39) I2 42%	RR 0.47 (0.17, 1.29) I2 0%	RR 0.57 (0.13, 2.52) I2 0%	MD -0.08 (-0.67,0.52) I2 46%	MD -0.91 (-1.51, -0.31) I2 68%
**Miscellaneous Anti-oxidant**	N/A	N/A	N/A	N/A	N/A	N/A	N/A	MD 17.00 (-77.25, 111.25) I2 N/A	N/A	N/A	N/A	N/A	N/A	N/A	MD 0.00 (-0.59, 0.59) I2 N/A

eTable8: Summary of results for sensitivity analysis including only those studies at low risk of allocation concealment. Colour codes indicate direction of treatment effect on that outcome. Red indicating a harmful effect and green indicating a beneficial effect.

Colour	Meaning
Green	Statistically significant beneficial effect in intervention group.
Red	Statistically significant harmful effect in intervention group.
Grey	Statistically significant effect, however only reportedby one study.
White	No statistically significant effect.

All Low risk of Bias studies	30 Day Mortality	Risk of AKI	Renal Replacement Therapy	Urine output 24 H	Creatinine Clearance (24H)	Creatinine Clearance (2-4 days)	Creatinine Clearance (5-7 days)	Perioperative Blood Loss	Adverse Reaction	Myocardial Infarction	Low Cardiac Output	Acute Brain Injury/Stroke	sepsis / infection	ICU LOS	Hospital LOS
**Atrial Natriuretic Peptide**	RR 3.30<br>[0.14, 79.16]<br>I2 N/A	RR 0.24<br>[0.07, 0.77]<br>I2 N/A	RR 0.73<br>[0.13, 4.21]<br>I2 N/A	N/A	N/A	N/A	N/A	N/A	N/A	N/A	N/A	N/A	N/A	N/A	MD -0.53<br>[-1.90, 0.84]<br>I2 N/A
**Alpha 2 agonist**	RR 0.42<br>(0.13, 1.38)<br>I2 0%	RR 0.62<br>(0.31, 1.25)<br>I2 76%	RR 0.73<br>[0.10, 5.16]<br>I2 70%	MD 0.02<br>(-0.17, 0.21)<br>I2 N/A	MD 21<br>(8.14, 33.86)<br>I2 N/A	MD -2<br>(-9.78, 5.78)<br>I2 N/A)	MD -2<br>(-10.63, 6.63)<br>I2 N/A	MD -126.84<br>(-240.96, -12.72)<br>I2 0%	N/A	RR 0.67<br>[0.11, 4.05]<br>I2 0%	RR 0.57<br>[0.13, 2.52]<br>I2 0%	RR 0.19<br>[0.05, 0.73]<br>I2 0%	RR 0.46<br>[0.18, 1.18]<br>I2 N/A	MD -0.51<br>[-1.16, 0.13]<br>I2 85%	MD -1.47<br>[-3.16, 0.22]<br>I2 51%
**Inodilators**	N/A	N/A	N/A	N/A	N/A	N/A	N/A	N/A	N/A	N/A	N/A	N/A	N/A	N/A	N/A
**Vasopressor**	N/A	N/A	N/A	N/A	N/A	N/A	N/A	N/A	N/A	N/A	N/A	N/A	N/A	N/A	N/A
**Calcium Channel Blocker**	N/A	N/A	N/A	N/A	N/A	N/A	N/A	N/A	N/A	N/A	N/A	N/A	N/A	N/A	N/A
**Nitric Oxide Donor**	RR 0.94<br>[0.06, 14.78]<br>I2 N/A	N/A	N/A	MD 0.16<br>[-0.01, 0.33]<br>I2 N/A	MD 6.60<br>[2.41, 10.79]<br>I2 N/A	MD 5.90<br>[2.04, 9.76]<br>I2 N/A	MD 3.10<br>[-0.81, 7.01]<br>I2 N/A	N/A	N/A	RR 0.70<br>[0.16, 3.07]<br>I2 N/A	N/A	RR 0.31<br>[0.01, 7.58]<br>I2 N/A	RR 0.94<br>[0.06, 14.78]<br>I2 N/A	N/A	N/A
**Statin**	N/A	N/A	N/A	N/A	N/A	N/A	N/A	N/A	N/A	N/A	N/A	N/A	N/A	N/A	N/A
**Other Diuretics (Spironolactone)**	N/A	N/A	N/A	N/A	N/A	N/A	N/A	N/A	N/A	N/A	N/A	N/A	N/A	N/A	N/A
**Restrictive vs Liberal**	N/A	N/A	N/A	N/A	N/A	N/A	N/A	N/A	N/A	N/A	N/A	N/A	N/A	N/A	N/A
**ACE Inhibitor**	N/A	N/A	N/A	N/A	N/A	N/A	N/A	N/A	N/A	N/A	N/A	N/A	N/A	N/A	N/A
**Dopamine Agonist**	RR 1.08<br>[0.82, 1.43]<br>I2 N/A	RR 0.74<br>[0.29, 1.87]<br>I2 79%	RR 0.93<br>[0.67, 1.29]<br>I2 N/A	MD 0.80<br>[0.56, 1.04]<br>I2 N/A	MD 29.50<br>[26.10, 32.90]<br>I2 N/A	MD 31.70<br>[27.39, 36.01]<br>I2 N/A	N/A	MD 70.00<br>[-172.18, 312.18]<br>I2 N/A	N/A	N/A	RR 1.03<br>[0.82, 1.30]<br>I2 70%	RR 1.03<br>[0.21, 5.05]<br>I2 N/A	RR 1.03<br>[0.67, 1.58]<br>I2 N/A	N/A	N/A
**Erythropoeitin**	RR 0.75<br>[0.17, 3.27]<br>I2 0%	RR 0.90<br>[0.65, 1.25]<br>I2 0%	RR 0.76<br>[0.17, 3.43]<br>I2 0%	MD 0.18<br>[-0.32, 0.68]<br>I2 N/A	N/A	N/A	N/A	N/A	N/A	RR 3.87<br>[0.44, 34.13]<br>I2 0%	RR 0.17<br>[0.03, 0.94]<br>I2 0%	RR 3.00<br>[0.13, 71.22]<br>I2 N/A	RR 1.00<br>[0.07, 15.36]<br>I2 N/A	MD -0.30<br>[-0.75, 0.15]<br>I2 N/A	MD 0.30<br>[-1.14, 1.74]<br>I2 N/A
**Glycaemic Control**	N/A	N/A	N/A	N/A	N/A	N/A	N/A	N/A	N/A	N/A	N/A	N/A	N/A	N/A	N/A
**Goal Directed Therapy**	N/A	RR 0.17<br>[0.05, 0.56]<br>I2 N/A	RR 0.17<br>[0.05, 0.56]<br>I2 N/A	N/A	N/A	N/A	N/A	MD -100.00<br>[-138.05, -61.95]<br>I2 N/A	N/A	RR 0.50<br>[0.05, 5.52]<br>I2 N/A	N/A	N/A	RR 0.34<br>[0.01, 8.21]<br>I2 N/A	MD -0.40<br>[-0.60, -0.20]<br>I2 N/A	MD -2.00<br>[-2.92, -1.08]<br>I2 N/A
**Loop Diuretics**	N/A	N/A	N/A	N/A	N/A	N/A	N/A	N/A	N/A	N/A	N/A	N/A	N/A	N/A	N/A
**N-Acetylcysteine**	RR 0.41<br>[0.06, 2.88]<br>I2 51%	RR 1.03<br>[0.77, 1.38]<br>I2 0%	RR 0.56<br>[0.19, 1.67]<br>I2 0%	MD -0.39<br>[-0.90, 0.12]<br>I2 69%	N/A	N/A	N/A	MD 73.84<br>[-40.00,	RR 1.69<br>[1.04, 2.73]<br>I2 0%	RR 0.71<br>[0.23, 2.18]<br>i2 N/A	RR 0.88<br>[0.70, 1.11]<br>I2 0%	RR 0.70<br>[0.27, 1.84]<br>I2 0%	RR 1.99<br>[0.37, 10.68]<br>I2 N/A	MD 0.02<br>[-0.20, 0.24]<br>I2 0%	MD 0.40<br>[-0.36, 1.16]<br>I2 0%

								187.69]<br>I2 0%							
**Corticosteroid Therapy**	N/A	N/A	N/A	N/A	N/A	N/A	N/A	N/A	N/A	N/A	N/A	N/A	N/A	N/A	N/A
**Osmotic Diuretics**	N/A	N/A	N/A	N/A	N/A	N/A	N/A	N/A	N/A	N/A	N/A	N/A	N/A	N/A	N/A
**Sodium Bicarbonate**	RR 2.39<br>[0.97, 5.93]<br>I2 0%	RR 0.93<br>[0.44, 1.94]<br>I2 87%	RR 1.35<br>[0.26, 6.94]<br>I2 0%	MD -0.03<br>[-0.42, 0.36]<br>I2 N/A	N/A	N/A	N/A	MD 150.00<br>[-35.40,<br>335.40]<br>I2 N/A	N/A	RR 5.06<br>[0.24, 104.58]<br>I2 N/A	RR 1.11<br>[0.90, 1.38]<br>I2 N/A	N/A	N/A	MD 0.19<br>[-0.19, 0.58]<br>I2 0%	MD -0.09<br>[-0.73, 0.55]<br>I2 0%
**Volatile vs Propofol Anaesthesia**	N/A	N/A	N/A	N/A	N/A	N/A	N/A	N/A	N/A	N/A	N/A	N/A	N/A	N/A	N/A
**Miscellaneous Interventions**	RR 0.35<br>[0.01, 8.48]<br>I2 N/A	RR 2.63<br>[0.53, 13.16]<br>I2 N/A	RR 1.58<br>[0.27, 9.19]<br>I2 N/A	MD -0.21<br>[-0.46, 0.04]<br>I2 N/A	N/A	N/A	N/A	MD 59.00<br>[-29.41,<br>147.41]<br>I2 N/A	N/A	RR 5.26<br>[0.26, 107.86]<br>I2 N/A	RR 1.10<br>[0.51, 2.40]<br>I2 0%	RR 0.26<br>[0.03, 2.34]<br>I2 0%	N/A	N/A	N/A
**Miscellaneous Anti-oxidant**	N/A	N/A	N/A	N/A	N/A	N/A	N/A	N/A	N/A	N/A	N/A	N/A	N/A	N/A	N/A

eTable9: Summary of results for sensitivity analysis including only those studies at low risk of bias. Colour codes indicate direction of treatment effect on that outcome. Red indicating a harmful effect and green indicating a beneficial effect.

(green)	Statistically significant beneficial effect in intervention group.
(red)	Statistically significant harmful effect in intervention group.
(grey)	Statistically significant effect, however only reportedby one study.
(white)	No statistically significant effect.

Question: Atrial Natriuretic Peptide compared to placebo for the Prevention of Renal Injury in the Perioperative Period.

Certainty assessment							№ of patients		Effect		Certainty	Importance
**№ of studies**	**Study design**	**Risk of bias**	**Inconsistency**	**Indirectness**	**Imprecision**	**Other considerations**	**Atrial Natriuretic Peptide**	**placebo**	**Relative (95% CI)**	**Absolute (95% CI)**		
**30-day mortality**												
10	randomised trials	not serious	not serious	serious [a]	serious	none	30/961 (3.1%)	52/976 (5.3%)	**RR 0.63** (0.41 to 0.97)	**20 fewer per 1,000** (from 31 fewer to 2 fewer)	⊕⊕◯◯ LOW	CRITICAL
**Risk of AKI**												
8	randomised trials	not serious	not serious	serious [a]	not serious	none	61/772 (7.9%)	150/783 (19.2%)	**RR 0.43** (0.33 to 0.56)	**109 fewer per 1,000** (from 128 fewer to 84 fewer)	⊕⊕⊕◯ MODERATE	CRITICAL
**Myocardial Infarction**												
3	randomised trials	serious [b]	not serious	not serious	serious [c]	none	5/387 (1.3%)	5/389 (1.3%)	**RR 0.98** (0.31 to 3.10)	**0 fewer per 1,000** (from 9 fewer to 27 more)	⊕⊕◯◯ LOW	IMPORTANT
**AKI / Dialysis**												
6	randomised trials	not serious	not serious	not serious	not serious	none	13/724 (1.8%)	58/736 (7.9%)	**RR 0.26** (0.15 to 0.47)	**58 fewer per 1,000** (from 67 fewer to 42 fewer)	⊕⊕⊕⊕ HIGH	CRITICAL
**Acute Brain Injury / Stroke**												
6	randomised trials	not serious	not serious	serious [d]	not serious	none	3/565 (0.5%)	7/522 (1.3%)	**RR 0.59** (0.18 to 1.93)	**5 fewer per 1,000** (from 11 fewer to 12 more)	⊕⊕⊕◯ MODERATE	IMPORTANT
**Sepsis and Infection**												
4	randomised trials	not serious	not serious	not serious	serious [c]	none	7/472 (1.5%)	1/477 (0.2%)	**RR 3.36** (0.81 to 13.88)	**5 more per 1,000** (from 0 fewer to 27 more)	⊕⊕⊕◯ MODERATE	IMPORTANT
**Hospital LOS**												
9	randomised trials	not serious	not serious	not serious	not serious	none	910	921	-	MD **2.6 lower** (3.69 lower to 1.5 lower)	⊕⊕⊕⊕ HIGH	NOT IMPORTANT

CI: Confidence interval; **RR:** Risk ratio; **MD:** Mean difference

Explanations

a. Chen 2007 and Ejaz 2009 evaluated nesiritide instead of ANP.
b. Unclear risk of performance bias across all studies.
c. Low number of events.
d. Chen 2007 evaluated nesiritide effect instead of ANP.

eTable10: GRADE Assessment for Atrial Natriuretic Peptide.

Author(s):
Question: Alpha 2 Agonist compared to placebo for the Prevention of Renal Injury in the Perioperative Period.
Setting:
Bibliography: . Interventions for the Prevention of Renal Injury in the Perioperative Period.. Cochrane Database of Systematic Reviews [Year], Issue [Issue].

Certainty assessment							№ of patients		Effect		Certainty	Importance
№ of studies	Study design	Risk of bias	Inconsistency	Indirectness	Imprecision	Other considerations	Alpha 2 Agonist	placebo	Relative (95% CI)	Absolute (95% CI)		
**30-day mortality**												
7	randomised trials	not serious	not serious	not serious	not serious	none	8/797 (1.0%)	18/796 (2.3%)	**RR 0.49** (0.22 to 1.11)	**12 fewer per 1,000** (from 18 fewer to 2 more)	⨁⨁⨁⨁ HIGH	CRITICAL
**Risk of AKI**												
10	randomised trials	not serious	not serious	not serious	not serious	none	538/4281 (12.6%)	333/2560 (13.0%)	**RR 0.69** (0.48 to 0.99)	**40 fewer per 1,000** (from 68 fewer to 1 fewer)	⨁⨁⨁⨁ HIGH	CRITICAL
**Myocardial Infarction**												
3	randomised trials	not serious	not serious	not serious	serious [a]	none	3/369 (0.8%)	6/366 (1.6%)	**RR 0.51** (0.13 to 2.08)	**8 fewer per 1,000** (from 14 fewer to 18 more)	⨁⨁⨁◯ MODERATE	IMPORTANT
**AKI / Dialysis**												
7	randomised trials	not serious	not serious	not serious	serious [b]	none	90/3921 (2.3%)	78/2164 (3.6%)	**RR 0.75** (0.43 to 1.33)	**9 fewer per 1,000** (from 21 fewer to 12 more)	⨁⨁⨁◯ MODERATE	CRITICAL
**Acute Brain Injury / Stroke**												
6	randomised trials	serious [c]	not serious	not serious	not serious	none	10/875 (1.1%)	26/791 (3.3%)	**RR 0.44** (0.21 to 0.93)	**18 fewer per 1,000** (from 26 fewer to 2 fewer)	⨁⨁⨁◯ MODERATE	IMPORTANT
**Sepsis and Infection**												
3	randomised trials	not serious	not serious	not serious	not serious	none	9/478 (1.9%)	21/484 (4.3%)	**RR 0.44** (0.21 to 0.94)	**24 fewer per 1,000** (from 34 fewer to 3 fewer)	⨁⨁⨁⨁ HIGH	IMPORTANT
**Hospital LOS**												
12	randomised trials	not serious	not serious	not serious	not serious	publication bias strongly suspected	616	594	-	MD **0.62 lower** (1.13 lower to 0.12 lower)	⨁⨁⨁◯ MODERATE	NOT IMPORTANT

CI: Confidence interval; **RR:** Risk ratio; **MD:** Mean difference

Explanations

a. Low number of events.
b. Several small studies with low number of events.
c. Risk of detection bias.

eTable11: GRADE Assessment for Alpha 2 Agonist.

Author(s):
Question: Inodilators compared to placebo for the Prevention of Renal Injury in the Perioperative Period.
Setting:
Bibliography: . Interventions for the Prevention of Renal Injury in the Perioperative Period.. Cochrane Database of Systematic Reviews [Year], Issue [Issue].

Certainty assessment							№ of patients		Effect		Certainty	Importance
№ of studies	Study design	Risk of bias	Inconsistency	Indirectness	Imprecision	Other considerations	Inodilators	placebo	Relative (95% CI)	Absolute (95% CI)		
**30-day mortality**												
12	randomised trials	not serious	not serious	not serious	not serious	publication bias strongly suspected	75/1242 (6.0%)	113/1253 (9.0%)	**RR 0.71** (0.53 to 0.94)	**26 fewer per 1,000** (from 42 fewer to 5 fewer)	⨁⨁⨁◯ MODERATE	CRITICAL
**Risk of AKI**												
9	randomised trials	not serious	not serious	not serious	not serious	publication bias strongly suspected	73/607 (12.0%)	112/612 (18.3%)	**RR 0.65** (0.50 to 0.85)	**64 fewer per 1,000** (from 92 fewer to 27 fewer)	⨁⨁⨁◯ MODERATE	CRITICAL
**Myocardial Infarction**												
5	randomised trials	not serious	not serious	not serious	not serious	publication bias strongly suspected	89/901 (9.9%)	95/907 (10.5%)	**RR 0.90** (0.60 to 1.35)	**10 fewer per 1,000** (from 42 fewer to 37 more)	⨁⨁⨁◯ MODERATE	IMPORTANT
**AKI / Dialysis**												
9	randomised trials	not serious	not serious	not serious	not serious	publication bias strongly suspected	61/1019 (6.0%)	98/1022 (9.6%)	**RR 0.63** (0.46 to 0.85)	**35 fewer per 1,000** (from 52 fewer to 14 fewer)	⨁⨁⨁◯ MODERATE	CRITICAL
**Acute Brain Injury / Stroke**												
7	randomised trials	not serious	not serious	not serious	not serious	none	53/972 (5.5%)	57/976 (5.8%)	**RR 0.91** (0.64 to 1.30)	**5 fewer per 1,000** (from 21 fewer to 18 more)	⨁⨁⨁⨁ HIGH	IMPORTANT
**Sepsis and Infection**												
5	randomised trials	not serious	not serious	not serious	not serious	none	47/927 (5.1%)	61/931 (6.6%)	**RR 0.77** (0.45 to 1.31)	**15 fewer per 1,000** (from 36 fewer to 20 more)	⨁⨁⨁⨁ HIGH	IMPORTANT
**Hospital LOS**												
8	randomised trials	not serious	very serious [a]	not serious	not serious	publication bias strongly suspected	597	614	-	MD **0.68 higher** (1.37 lower to 2.74 higher)	⨁◯◯◯ VERY LOW	NOT IMPORTANT

CI: Confidence interval; **RR:** Risk ratio; **MD:** Mean difference

Explanations

a. Extreme inconsistency of results across the studies.

eTable 12: GRADE Assessments for Inodilators.

Author(s):
Question: Vasopressor/Vasoconstrictor compared to placebo for the Prevention of Renal Injury in the Perioperative Period.
Setting:
Bibliography: . Interventions for the Prevention of Renal Injury in the Perioperative Period.. Cochrane Database of Systematic Reviews [Year], Issue [Issue].

Certainty assessment							№ of patients		Effect		Certainty	Importance
№ of studies	Study design	Risk of bias	Inconsistency	Indirectness	Imprecision	Other considerations	Vasopressor/Vasoconstrictor	placebo	Relative (95% CI)	Absolute (95% CI)		
**30-day mortality**												
3	randomised trials	serious [a]	not serious	not serious	not serious	none	15/380 (3.9%)	12/289 (4.2%)	**RR 1.05** (0.50 to 2.21)	**2 more per 1,000** (from 21 fewer to 50 more)	⨁⨁⨁◯ MODERATE	CRITICAL
**Risk of AKI**												
3	randomised trials	serious [a]	not serious	not serious	not serious	none	30/380 (7.9%)	43/289 (14.9%)	**RR 0.56** (0.36 to 0.86)	**65 fewer per 1,000** (from 95 fewer to 21 fewer)	⨁⨁⨁◯ MODERATE	CRITICAL
**Myocardial Infarction**												
1	randomised trials	serious [b]	not serious	not serious	serious [c]	none	0/149 (0.0%)	1/149 (0.7%)	**RR 0.33** (0.01 to 8.12)	**4 fewer per 1,000** (from 7 fewer to 48 more)	⨁⨁◯◯ LOW	IMPORTANT
**AKI / Dialysis**												
1	randomised trials	serious [d]	not serious	not serious	serious [e]	none	9/149 (6.0%)	9/149 (6.0%)	**RR 1.00** (0.41 to 2.45)	**0 fewer per 1,000** (from 36 fewer to 88 more)	⨁⨁◯◯ LOW	CRITICAL
**Acute Brain injury / Stroke**												
1	randomised trials	serious [a]	not serious	not serious	serious [e]	none	1/206 (0.5%)	0/115 (0.0%)	**RR 1.68** (0.07 to 40.94)	**0 fewer per 1,000** (from 0 fewer to 0 fewer)	⨁⨁◯◯ LOW	IMPORTANT
**Sepsis and infection**												
2	randomised trials	serious [a]	not serious	not serious	serious [f]	none	19/355 (5.4%)	27/264 (10.2%)	**RR 0.60** (0.34 to 1.08)	**41 fewer per 1,000** (from 67 fewer to 8 more)	⨁⨁◯◯ LOW	IMPORTANT
**Hospital LOS**												
1	randomised trials	serious [a]	not serious	not serious	serious [e]	none	149	149	-	MD **2 lower** (4.37 lower to 0.37 higher)	⨁⨁◯◯ LOW	NOT IMPORTANT

CI: Confidence interval; **RR:** Risk ratio; **MD:** Mean difference

Explanations

a. Risk of selection, performance and detection bias.
b. Risk of selection bias.
c. Results related to a single trial, low number of events.
d. Risk of reporting bias.
e. Results related to a single trial.
f. Results limited to two trials.

eTable13: GRADE Assessments for Vasopressors

Author(s):
Question: Calcium Channel Blockers compared to placebo for the Prevention of Renal Injury in the Perioperative Period.
Setting:
Bibliography: . Interventions for the Prevention of Renal Injury in the Perioperative Period.. Cochrane Database of Systematic Reviews [Year], Issue [Issue].

Certainty assessment							№ of patients		Effect		Certainty	Importance
№ of studies	Study design	Risk of bias	Inconsistency	Indirectness	Imprecision	Other considerations	Calcium Channel Blockers	placebo	Relative (95% CI)	Absolute (95% CI)		
**Risk of AKI**												
2	randomised trials	serious [a]	not serious	not serious	serious [b]	none	3/82 (3.7%)	15/82 (18.3%)	**RR 0.22** (0.07 to 0.76)	**143 fewer per 1,000** (from 170 fewer to 44 fewer)	⨁⨁◯◯ LOW	CRITICAL
**Myocardial Infarction**												
2	randomised trials	serious [c]	not serious	not serious	serious [d]	none	0/22 (0.0%)	2/22 (9.1%)	**RR 0.33** (0.04 to 2.98)	**61 fewer per 1,000** (from 87 fewer to 180 more)	⨁⨁◯◯ LOW	IMPORTANT
**AKI / Dialysis**												
3	randomised trials	serious [a]	not serious	not serious	not serious	none	2/92 (2.2%)	14/92 (15.2%)	**RR 0.19** (0.05 to 0.75)	**123 fewer per 1,000** (from 145 fewer to 38 fewer)	⨁⨁⨁◯ MODERATE	CRITICAL
**Acute Brain Injury / Stroke**												
1	randomised trials	very serious [c]	not serious	not serious	very serious [d]	none	1/12 (8.3%)	1/12 (8.3%)	**RR 1.00** (0.07 to 14.21)	**0 fewer per 1,000** (from 77 fewer to 1,000 more)	⨁◯◯◯ VERY LOW	IMPORTANT
**Hospital LOS**												
2	randomised trials	not serious	not serious	not serious	serious [b]	none	100	100	-	MD **0** (1.96 lower to 1.96 higher)	⨁⨁⨁◯ MODERATE	NOT IMPORTANT

CI: Confidence interval; **RR:** Risk ratio; **MD:** Mean difference

Explanations

a. Unclear risk of selection and performance bias.
b. Data obtained from two trials.
c. Risk of selection, detection and performance bias.
d. Extremely low number of participants and events.

eTable 14: GRADE Assessment for calcium channel blockers.

Author(s):
Question: Nitric Oxide Donor compared to placebo for the Prevention of Renal Injury in the Perioperative Period.
Setting:
Bibliography: . Interventions for the Prevention of Renal Injury in the Perioperative Period.. Cochrane Database of Systematic Reviews [Year], Issue [Issue].

Certainty assessment							№ of patients		Effect		Certainty	Importance
№ of studies	Study design	Risk of bias	Inconsistency	Indirectness	Imprecision	Other considerations	Nitric Oxide Donor	placebo	Relative (95% CI)	Absolute (95% CI)		
**30-day mortality**												
2	randomised trials	not serious	not serious	not serious	serious [a]	none	3/253 (1.2%)	8/247 (3.2%)	**RR 0.38** (0.10 to 1.49)	**20 fewer per 1,000** (from 29 fewer to 16 more)	⨁⨁⨁◯ MODERATE	CRITICAL
**Risk of AKI**												
1	randomised trials	serious [b]	not serious	not serious	serious [c]	none	58/129 (45.0%)	81/131 (61.8%)	**RR 0.73** (0.58 to 0.92)	**167 fewer per 1,000** (from 260 fewer to 49 fewer)	⨁⨁◯◯ LOW	CRITICAL
**Myocardial Infarction**												
1	randomised trials	not serious	not serious	not serious	serious [c]	none	3/124 (2.4%)	4/116 (3.4%)	**RR 0.70** (0.16 to 3.07)	**10 fewer per 1,000** (from 29 fewer to 71 more)	⨁⨁⨁◯ MODERATE	IMPORTANT
**AKI / Dialysis**												
1	randomised trials	serious [b]	not serious	not serious	serious [c]	none	4/129 (3.1%)	6/131 (4.6%)	**RR 0.68** (0.20 to 2.34)	**15 fewer per 1,000** (from 37 fewer to 61 more)	⨁⨁◯◯ LOW	CRITICAL
**Acute Brain Injury / Stroke**												
1	randomised trials	not serious	not serious	not serious	serious [c]	none	0/124 (0.0%)	1/116 (0.9%)	**RR 0.31** (0.01 to 7.58)	**6 fewer per 1,000** (from 9 fewer to 57 more)	⨁⨁⨁◯ MODERATE	IMPORTANT
**Sepsis and Infection**												
1	randomised trials	not serious	not serious	not serious	serious [c]	none	1/124 (0.8%)	1/116 (0.9%)	**RR 0.94** (0.06 to 14.78)	**1 fewer per 1,000** (from 8 fewer to 119 more)	⨁⨁⨁◯ MODERATE	IMPORTANT
**Hospital LOS**												
1	randomised trials	serious [b]	not serious	not serious	serious [c]	none	129	131	-	MD **0** (0.64 lower to 0.64 higher)	⨁⨁◯◯ LOW	NOT IMPORTANT

CI: Confidence interval; **RR:** Risk ratio; **MD:** Mean difference

Explanations

a. Two trials included, low number of events.
b. Risk of selection bias.
c. Results related to a single trial.

eTable 15: GRADE Assessments for Nitric Oxide Donors.

Author(s):
Question: Other Diuretics compared to placebo for the Prevention of Renal Injury in the Perioperative Period.
Setting:
Bibliography: . Interventions for the Prevention of Renal Injury in the Perioperative Period.. Cochrane Database of Systematic Reviews [Year], Issue [Issue].

Certainty assessment							№ of patients		Effect		Certainty	Importance
№ of studies	Study design	Risk of bias	Inconsistency	Indirectness	Imprecision	Other considerations	Other Diuretics	placebo	Relative (95% CI)	Absolute (95% CI)		
**30-day mortality**												
2	randomised trials	not serious	not serious	not serious	very serious [a]	none	8/276 (2.9%)	7/201 (3.5%)	**RR 0.83** (0.15 to 4.75)	**6 fewer per 1,000** (from 30 fewer to 131 more)	⨁⨁◯◯ LOW	CRITICAL
**Risk of AKI**												
1	randomised trials	serious [b]	not serious	not serious	very serious [c]	none	50/123 (40.7%)	34/125 (27.2%)	**RR 1.49** (1.05 to 2.14)	**133 more per 1,000** (from 14 more to 310 more)	⨁◯◯◯ VERY LOW	CRITICAL
**Myocardial Infarction**												
1	randomised trials	serious [d]	not serious	not serious	very serious [c]	none	4/153 (2.6%)	2/76 (2.6%)	**RR 0.99** (0.19 to 5.30)	**0 fewer per 1,000** (from 21 fewer to 113 more)	⨁◯◯◯ VERY LOW	IMPORTANT
**AKI / Dialysis**												
1	randomised trials	serious [b]	not serious	not serious	very serious [c]	none	9/123 (7.3%)	8/125 (6.4%)	**RR 1.14** (0.46 to 2.87)	**9 more per 1,000** (from 35 fewer to 120 more)	⨁◯◯◯ VERY LOW	CRITICAL
**Acute Brain injury / Stroke**												
0	randomised trials						0/0	0/0	not pooled	see comment	-	IMPORTANT
**Sepsis and infection**												
1	randomised trials	serious [d]	not serious	not serious	very serious [c]	none	2/153 (1.3%)	3/76 (3.9%)	**RR 0.33** (0.06 to 1.94)	**26 fewer per 1,000** (from 37 fewer to 37 more)	⨁◯◯◯ VERY LOW	IMPORTANT
**Hospital LOS**												
1	randomised trials	serious [d]	not serious	not serious	very serious [c]	none	153	76	-	MD **1 lower** (2.92 lower to 0.92 higher)	⨁◯◯◯ VERY LOW	NOT IMPORTANT

CI: Confidence interval; **RR:** Risk ratio; **MD:** Mean difference

Explanations

a. 2 trials included, low number of events and participants.
b. Unclear risk of attrition bias.
c. Single trial, low number of participants.
d. Unclear risk of selection and detection bias.

eTable 16: GRADE Assessments for Other Diuretics (Spironolactone)

Author(s):
Question: Restrictive compared to Liberal intervention for the Prevention of Renal Injury in the Perioperative Period.
Setting:
Bibliography: . Interventions for the Prevention of Renal Injury in the Perioperative Period.. Cochrane Database of Systematic Reviews [Year], Issue [Issue].

Certainty assessment							№ of patients		Effect		Certainty	Importance
№ of studies	Study design	Risk of bias	Inconsistency	Indirectness	Imprecision	Other considerations	Restrictive	Liberal intervention	Relative (95% CI)	Absolute (95% CI)		
**30-day mortality**												
8	randomised trials	serious [a]	not serious	not serious	serious	none	245/4249 (5.8%)	257/4258 (6.0%)	**RR 0.95** (0.80 to 1.13)	**3 fewer per 1,000** (from 12 fewer to 8 more)	⨁⨁◯◯ LOW	CRITICAL
**Risk of AKI**												
9	randomised trials	serious [b]	not serious	not serious	not serious	none	165/2296 (7.2%)	99/2306 (4.3%)	**RR 1.64** (1.29 to 2.09)	**27 more per 1,000** (from 12 more to 47 more)	⨁⨁⨁◯ MODERATE	CRITICAL
**Myocardial Infarction**												
4	randomised trials	serious [a]	not serious	not serious	not serious	none	165/2623 (6.3%)	164/2635 (6.2%)	**RR 1.00** (0.81 to 1.23)	**0 fewer per 1,000** (from 12 fewer to 14 more)	⨁⨁⨁◯ MODERATE	IMPORTANT
**AKI / Dialysis**												
6	randomised trials	serious [b]	not serious	not serious	not serious	none	106/4203 (2.5%)	103/4216 (2.4%)	**RR 1.22** (0.70 to 2.11)	**5 more per 1,000** (from 7 fewer to 27 more)	⨁⨁⨁◯ MODERATE	CRITICAL
**Acute Brain Injury / Stroke**												
7	randomised trials	serious [a]	not serious	not serious	not serious	none	94/2827 (3.3%)	79/2838 (2.8%)	**RR 1.18** (0.88 to 1.58)	**5 more per 1,000** (from 3 fewer to 16 more)	⨁⨁⨁◯ MODERATE	IMPORTANT
**Sepsis and Infection**												
11	randomised trials	serious [a]	not serious	not serious	not serious	none	485/2121 (22.9%)	420/2130 (19.7%)	**RR 1.00** (0.65 to 1.55)	**0 fewer per 1,000** (from 69 fewer to 108 more)	⨁⨁⨁◯ MODERATE	IMPORTANT
**Hospital LOS**												
6	randomised trials	serious [b]	not serious	not serious	not serious	none	1739	1740	-	MD **0.64 higher** (0.1 lower to 1.38 higher)	⨁⨁⨁◯ MODERATE	NOT IMPORTANT

CI: Confidence interval; **RR:** Risk ratio; **MD:** Mean difference

Explanations

a. Risk of performance, detection and attrition bias.
b. Risk of performance and detection bias.

eTable17: GRADE Assessment for Restrictivs vs Liberal fluid therapy.

References:

1. Billings F, Balaguer J, Yu C, Wright P, Petracek M, Byrne J, et al. Comparative Effects of Angiotensin Receptor Blockade and ACE Inhibition on the Fibrinolytic and Inflammatory Responses to Cardiopulmonary Bypass. Clin Pharmacol Ther. 2012;91(6):1065-73.
2. Colson PMD, Ribstein JMD, Mimran AMD, Grolleau DMD, Chaptal PAMD, Roquefeuil BMD. Effect of Angiotensin Converting Enzyme Inhibition on Blood Pressure and Renal Function during Open Heart Surgery. Anesthesiology: The Journal of the American Society of Anesthesiologists. 1990;72(1):23-7.
3. Colson P, Saussine M, Séguin JR, Cuchet D, ChaptalG P-A, Roquefeuil B. Hemodynamic Effects of Anesthesia in Patients Chronically Treated With Angiotensin-Converting Enzyme Inhibitors. Anesthesia & Analgesia. 1992;74(6).
4. Licker MMD, Neidhart PMD, Lustenberger SMD, Valloton MBMD, Kalonji TMD, Fathi MP, et al. Long-term Angiotensin-converting Enzyme Inhibitor Treatment Attenuates Adrenergic Responsiveness without Altering Hemodynamic Control in Patients Undergoing Cardiac Surgery. Anesthesiology: The Journal of the American Society of Anesthesiologists. 1996;84(4):789-800.
5. Pretorius M, Murray KT, Yu C, Byrne JG, Billings FTt, Petracek MR, et al. Angiotensin-converting enzyme inhibition or mineralocorticoid receptor blockade do not affect prevalence of atrial fibrillation in patients undergoing cardiac surgery. Critical care medicine. 2012;40(10):2805-12.
6. Ryckwaert F, Colson P, Ribstein J, Boccara G, Guillon G. Haemodynamic and renal effects of intravenous enalaprilat during coronary artery bypass graft surgery in patients with ischaemic heart dysfunction†. British Journal of Anaesthesia. 2001;86(2):169-75.
7. Ammar A, Mahmoud K, Kasemy Z, Helwa M. Cardiac and renal protective effects of dexmedetomidine in cardiac surgeries: A randomized controlled trial. Saudi Journal of Anaesthesia. 2016;10(4):395-401.
8. Balkanay OO, Goksedef D, Omeroglu SN, Ipek G. The dose-related effects of Dexmedetomidine on renal functions and serum neutrophil gelatinase-associated lipocalin values after coronary artery bypass grafting: a randomized, triple-blind, placebo-controlled study. Interactive CardioVascular and Thoracic Surgery. 2014;20(2):209-14.
9. Cheng XQ, Mei B, Zuo YM, Wu H, Peng XH, Zhao Q, et al. A multicentre randomised controlled trial of the effect of intra-operative dexmedetomidine on cognitive decline after surgery. Anaesthesia. 2019;74(6):741-50.
10. Chi X, Liao M, Chen X, Zhao Y, Yang L, Luo A, et al. Dexmedetomidine Attenuates Myocardial Injury in Off-Pump Coronary Artery Bypass Graft Surgery. Journal of Cardiothoracic and Vascular Anesthesia. 2016;30(1):44-50.
11. Cho JS, Shim J-K, Soh S, Kim MK, Kwak Y-L. Perioperative dexmedetomidine reduces the incidence and severity of acute kidney injury following valvular heart surgery. Kidney International. 2016;89(3):693-700.
12. Djaiani G, Silverton N, Fedorko L, Carroll J, Styra R, Rao V, et al. Dexmedetomidine versus Propofol Sedation Reduces Delirium after Cardiac Surgery: A Randomized Controlled Trial. Anesthesiology: The Journal of the American Society of Anesthesiologists. 2016;124(2):362-8.
13. Garg AX, Kurz A, Sessler DI, Cuerden M, Robinson A, Mrkobrada M, et al. Perioperative aspirin and clonidine and risk of acute kidney injury: A randomized clinical trial. JAMA - Journal of the American Medical Association. 2014;312(21):2254-64.
14. Kulka PJ, Tryba M, Zenz M. Preoperative alpha sub 2-adrenergic receptor agonists prevent the deterioration of renal function after cardiac surgery: Results of a randomized, controlled trial. Critical Care Medicine. 1996;24(6).
15. Li X, Yang J, Nie X-L, Zhang Y, Li X-Y, Li L-H, et al. Impact of dexmedetomidine on the incidence of delirium in elderly patients after cardiac surgery: A randomized controlled trial. PloS one. 2017;12(2):e0170757-e.
16. Liu X, Zhang K, Wang W, Xie G, Fang X. Dexmedetomidine sedation reduces atrial fibrillation after cardiac surgery compared to propofol: a randomized controlled trial. Critical care (London, England). 2016;20(1):298-.
17. Park JB, Bang SH, Chee HK, Kim JS, Lee SA, Shin JK. Efficacy and safety of dexmedetomidine for postoperative delirium in adult cardiac surgery on cardiopulmonary bypass. Korean J Thorac Cardiovasc Surg. 2014;47(3):249-54.

18. Soliman R, Zohry G. The myocardial protective effect of dexmedetomidine in high-risk patients undergoing aortic vascular surgery. Annals of Cardiac Anaesthesia. 2016;19(4):606-13
19. Soliman R, Hussien M. Comparison of the renoprotective effect of dexmedetomidine and dopamine in high-risk renal patients undergoing cardiac surgery: A double-blind randomized study. Annals of Cardiac Anaesthesia. 2017;20(4):408-15.
20. Song Y, Kim DH, Kwon TD, Han DW, Baik SH, Jung HH, et al. Effect of intraoperative dexmedetomidine on renal function after cytoreductive surgery and hyperthermic intraperitoneal chemotherapy: a randomized, placebo-controlled trial. International journal of hyperthermia. 2018.
21. Wang Z, Huang C, Hua Y, Huang W, Deng L, Liu K. Dexmedetomidine reduces intestinal and hepatic injury after hepatectomy with inflow occlusion under general anaesthesia: a randomized controlled trial. BR J ANAESTH. 2014;112(6):1055-64.
22. Zhai M, Kang F, Han M, Huang X, Li J. The effect of dexmedetomidine on renal function in patients undergoing cardiac valve replacement under cardiopulmonary bypass: A double-blind randomized controlled trial. Journal of Clinical Anesthesia. 2017;40:33-8.
23. Salah M, El-Tawil T, Nasr S, Nosser T. Does dexmedetomidine affect renal outcome in patients with renal impairment undergoing CABG? http://wwwejcaegnet/articleasp?issn=1687-9090;year=2013;volume=7;issue=1;spage=7;epage=12;aulast=Salah. 2013;7(1):7-12.
24. Chen Horng H, Sundt Thoralf M, Cook David J, Heublein Denise M, Burnett John C. Low Dose Nesiritide and the Preservation of Renal Function in Patients With Renal Dysfunction Undergoing Cardiopulmonary-Bypass Surgery. Circulation. 2007;116(11_supplement):I-134-I-8.
25. Ejaz AA, Martin TD, Johnson RJ, Winterstein AG, Klodell CT, Hess PJ, et al. Prophylactic nesiritide does not prevent dialysis or all-cause mortality in patients undergoing high-risk cardiac surgery. The Journal of Thoracic and Cardiovascular Surgery. 2009;138(4):959-64.
26. Hisatomi K, Eishi K. Multicenter trial of carperitide in patients with renal dysfunction undergoing cardiovascular surgery. General Thoracic and Cardiovascular Surgery. 2012;60(1):21-30.
27. Izumi K, Eishi K, Hashizume K, Tada S, Yamane K, Takai H, et al. The efficacy of human atrial natriuretic peptide in patients with renal dysfunction undergoing cardiac surgery. Annals of Thoracic and Cardiovascular Surgery. 2008;14(5):1341-098.
28. Mentzer RM, Oz MC, Sladen RN, Graeve AH, Hebeler RF, Luber JM, et al. Effects of Perioperative Nesiritide in Patients With Left Ventricular Dysfunction Undergoing Cardiac Surgery: The NAPA Trial. Journal of the American College of Cardiology. 2007;49(6):716-26.
29. Mitaka C, Kudo T, Jibiki M, Sugano N, Inoue Y, Makita K, et al. Effects of human atrial natriuretic peptide on renal function in patients undergoing abdominal aortic aneurysm repair*. Critical Care Medicine. 2008;36(3).
30. Mitaka C, Ohnuma T, Murayama T, Kunimoto F, Nagashima M, Takei T, et al. Effects of low-dose atrial natriuretic peptide infusion on cardiac surgery-associated acute kidney injury: A multicenter randomized controlled trial. Journal of critical care. 2017;38:253-8.
31. Mori Y, Kamada T, Ochiai R. Reduction in the incidence of acute kidney injury after aortic arch surgery with low-dose atrial natriuretic peptide: A randomised controlled trial. Eur J Anaesthesiol. 2014;31(7):381-7.
32. Sezai A, Shiono M, Orime Y, Hata H, Hata M, Negishi N, et al. Low-dose continuous infusion of human atrial natriuretic peptide during and after cardiac surgery. The Annals of Thoracic Surgery. 2000;69(3):732-8.
33. Sezai A, Hata M, Wakui S, Shiono M, Negishi N, Kasamaki Y, et al. Efficacy of Low-Dose Continuous Infusion of α-Human Atrial Natriuretic Peptide (hANP) During Cardiac Surgery. Possibility of Postoperative Left Ventricular Remodeling Effect. Circulation Journal. 2006;70(11):1426-31.
34. Sezai A, Hata M, Wakui S, Niino T, Takayama T, Hirayama A, et al. Efficacy of Continuous Low-Dose hANP Administration in Patients Undergoing Emergent Coronary Artery Bypass Grafting for Acute Coronary Syndrome. Circulation Journal. 2007;71(9):1401-7.

35. Sezai A, Hata M, Niino T, Yoshitake I, Unosawa S, Wakui S, et al. Influence of Continuous Infusion of Low-Dose Human Atrial Natriuretic Peptide on Renal Function During Cardiac Surgery: A Randomized Controlled Study. Journal of the American College of Cardiology. 2009;54(12):1058-64.
36. Sezai A, Hata M, Niino T, Yoshitake I, Unosawa S, Wakui S, et al. Results of Low-Dose Human Atrial Natriuretic Peptide Infusion in Nondialysis Patients With Chronic Kidney Disease Undergoing Coronary Artery Bypass Grafting: The NU-HIT (Nihon University Working Group Study of Low-Dose hANP Infusion Therapy During Cardiac Surgery) Trial for CKD. Journal of the American College of Cardiology. 2011;58(9):897-903.
37. Sezai A, Nakata K-i, Iida M, Yoshitake I, Wakui S, Hata H, et al. Results of low-dose carperitide infusion in high-risk patients undergoing coronary artery bypass grafting. The Annals of thoracic surgery. 2013;96(1):119-26.
38. Amano J, Suzuki A, Sunamori M, Tofukuji M. Effect of Calcium Antagonist Diltiazem on Renal Function in Open Heart Surgery. Chest. 1995;107(5):1260-5.
39. Bergman ASF, Odar-Cederlöf I, Westman L, Bjellerup P, Höglund P, Öhqvist G. Diltiazem infusion for renal protection in cardiac surgical patients with preexisting renal dysfunction. Journal of Cardiothoracic and Vascular Anesthesia. 2002;16(3):294-9.
40. Bertolissi M, Antonucci F, De Monte A, Padovani R, Giordano F. Effects on renal function of a continuous infusion of nifedipine during cardiopulmonary bypass. Journal of Cardiothoracic and Vascular Anesthesia. 1996;10(2):238-42.
41. Cho JE, Shim JK, Chang JH, Oh YJ, Kil HK, Rha KH, et al. Effect of Nicardipine on Renal Function After Robot-assisted Laparoscopic Radical Prostatectomy. 2009;73(5):1056-60
42. Colson P, Ribstein J, Séguin JR, Marty-Ane C, Roquefeuil B. Mechanisms of Renal Hemodynamic Impairment During Infrarenal Aortic Cross-Clamping. Anesthesia & Analgesia. 1992;75(1).
43. de Lasson L, Hansen HE, Juhl B, Paaske WP, Pedersen EB. Effect of felodipine on renal function and vasoactive hormones in infrarenal aortic surgery. British Journal of Anaesthesia. 1997;79(6):719-25.
44. Dönmez A, Ergün F, Kayhan Z, Taşdelen A, Doğan S. Verapamil and nimodipine do not improve renal function during cardiopulmonary bypass. Acta Anaesthesiologica Italica. 1998;49:173,7.
45. Huh H, Kim NY, Park SJ, Cho JE. Effect of nicardipine on renal function following robot-assisted laparoscopic radical prostatectomy in patients with pre-existing renal insufficiency. Journal of International Medical Research. 2014;42(2):427-35.
46. Kim JE, Lee JS, Kim MK, Kim SH, Kim JY. Nicardipine infusion for hypotensive anesthesia during orthognathic surgery has protective effect on renal function. Journal of Oral and Maxillofacial Surgery. 2014;72(1):41-6.
47. Park C, Kim JY, Kim C, Chang CH. Nicardipine Effects on Renal Function During Spine Surgery. Clinical spine surgery. 2017;30(7):E954-E8.
48. Witczak BJ, Hartmann A, Geiran OR, Bugge JF. Renal function after cardiopulmonary bypass surgery in patients with impaired renal function. A randomized study of the effect of nifedipine. European Journal of Anaesthesiology (EJA). 2008;25(4).
49. Yavuz S, Ayabakan N, Goncu M, Ozdemir I. Effect of combined dopamine and diltiazem on renal function after cardiac surgery. Medical science monitor : international medical journal of experimental and clinical research. 2002;8:PI45-50.
50. Zanardo G, Michielon P, Rosi P, Teodori T, Antonucci F, Caenaro G, et al. Effects of a continuous diltiazem infusion on renal function during cardiac surgery. Journal of Cardiothoracic and Vascular Anesthesia. 1993;7(6):711-6.
51. Barr LF, Kolodner K. N-acetylcysteine and fenoldopam protect the renal function of patients with chronic renal insufficiency undergoing cardiac surgery. Critical Care Medicine. 2008;36(5).
52. Berendes E, Möllhoff T, Van Aken H, Schmidt C, Erren M, Deng MC, et al. Effects of dopexamine on creatinine clearance, systemic inflammation, and splanchnic oxygenation in patients undergoing coronary artery bypass

grafting. Anesthesia and analgesia. 1997;84(5):950-7.
53. Bove T, Zangrillo A, Guarracino F, Alvaro G, Persi B, Maglioni E, et al. Effect of Fenoldopam on Use of Renal Replacement Therapy Among Patients With Acute Kidney Injury After Cardiac Surgery: A Randomized Clinical Trial. JAMA. 2014;312(21):2244-53.
54. Carcoana OV, Mathew JP, Davis E, Byrne DW, Hayslett JP, Hines RL, et al. Mannitol and Dopamine in Patients Undergoing Cardiopulmonary Bypass: A Randomized Clinical Trial. Anesthesia & Analgesia. 2003;97(5).
55. Cogliati AA, Vellutini R, Nardini A, Urovi S, Hamdan M, Landoni G, et al. Fenoldopam Infusion for Renal Protection in High-Risk Cardiac Surgery Patients: A Randomized Clinical Study. Journal of Cardiothoracic and Vascular Anesthesia. 2007;21(6):847-50.
56. Costa P, Ottino GM, Matani A, Pansini S, Canavese C, Passerini G, et al. Low-dose dopamine during cardiopulmonary bypass in patients with renal dysfunction. Journal of Cardiothoracic Anesthesia. 1990;4(4):469-73.
57. Cregg N, Mannion D, Casey W. Oliguria during corrective spinal surgery for idiopathic scoliosis: the role of antidiuretic hormone. Pediatric Anesthesia. 1999;9(6):505-14.
58. de Lasson L, Hansen HE, Juhl B, Paaske WP, Pedersen EB. A randomised, clinical study of the effect of low-dose dopamine on central and renal haemodynamics in infrarenal aortic surgery. European Journal of Vascular and Endovascular Surgery. 1995;10(1):82-90.
59. Dehne MG, Dehne MG, Klein TF, Mühling J, Sablotzki A, Osmer C, et al. impairment of renal function after cardiopulmonary bypass is not influenced by dopexamine. Renal Failure. 2001;23(2):217-30.
60. Dural O, Ozkara A, Celebioglu B, Kanbak N, Ciliv G, Aypar U. Comparative study of dopamine and mannitol effects on renal function during cardiopulmonary bypass by using N-acetyl-β-D-glucosaminidase assay. Turkish Journal of Medical Sciences. 2000;30:453-7.
61. Halpenny M, Rushe C, Breen P, Cunningham AJ, Boucher-Hayes D, Shorten GD. The effects of fenoldopam on renal function in patients undergoing elective aortic surgery. European Journal of Anaesthesiology (EJA). 2002;19(1).
62. Kanchi M, Manjunath R, Massen J, Vincent L, Belani K. Neutrophil gelatinase-associated lipocalin as a biomarker for predicting acute kidney injury during off-pump coronary artery bypass grafting. Annals of Cardiac Anaesthesia. 2017;20(3):297-302.
63. Lassnigg A, Donner EVA, Grubhofer G, Presterl E, Druml W, Hiesmayr M. Lack of Renoprotective Effects of Dopamine and Furosemide during Cardiac Surgery. Journal of the American Society of Nephrology. 2000;11(1):97.
64. Myles PS, Buckland MR, Schenk NJ, Cannon GB, Langley M, Davis BB, et al. Effect of "Renal-Dose" Dopamine on Renal Function following Cardiac Surgery. Anaesthesia and Intensive Care. 1993;21(1):56-60.
65. Parks RW, Diamond T, McCrory DC, Johnston GW, Rowlands BJ. Prospective study of postoperative renal function in obstructive jaundice and the effect of perioperative dopamine. BJS (British Journal of Surgery). 1994;81(3):437-9.
66. Pérez J, Taurá P, Rueda J, Balust J, Anglada T, Beltran J, et al. Role of dopamine in renal dysfunction during laparoscopic surgery. Surgical Endoscopy And Other Interventional Techniques. 2002;16(9):1297-301.
67. Russo A, Bevilacqua F, Scagliusi A, Scarano A, Di Stasio E, Marana R, et al. Dopamine infusion and fluid administration improve renal function during laparoscopic surgery. Minerva Anestesiologica. 2014;80(4):452-60.
68. Tang ATM, El-Gamel A, Keevil B, Yonan N, Deiraniya AK. The effect of 'renal-dose' dopamine on renal tubular function following cardiac surgery: assessed by measuring retinol binding protein (RBP)1. European Journal of Cardio-Thoracic Surgery. 1999;15(5):717-22.
69. Wahbah A, el-Hefny M, Wafa EMW, el-Kharbotly W, El-Enin A, Zaglol A, et al. Perioperative renal protection in patients with obstructive jaundice using drug combinations. Hepato-gastroenterology. 2000;47:1691-4.
70. Welch M, Newstead CG, Smyth JV, Dodd PD, Walker MG. Evaluation of dopexamine hydrochloride as a renoprotective agent during

aortic surgery. Annals of Vascular Surgery. 1995;9(5):488-92.
71. Woo EBC, Tang ATM, El Gamel A, Keevil B, Greenhalgh D, Patrick M, et al. Dopamine therapy for patients at risk of renal dysfunction following cardiac surgery: science or fiction? European Journal of Cardio-Thoracic Surgery. 2002;22(1):106-11.
72. Yavuz S, Ayabakan N, Dilek K, Ozdemir A. Renal dose dopamine in open heart surgery. Does it protect renal tubular function? The Journal of cardiovascular surgery. 2002;43(1):25-30.
73. Ahmed F. Abdel-latif1 SMS, Hatem S. Mohammed1, Mohamed Abdel-Bary2, Mona M. Abdelmegid3, Abdelkader Ahmed Hashim4, Mohammed H. Hassan5*, Ahmed Farouk6 and Hany A. Ibrahim7. Does erythropoietin have a reno-protective impact in patients undergoing Coronary Artery Bypass Grafting? A randomized, double-blind, placebo-controlled trial. Http://wwwwhoint/trialsearch/trial2aspx? Trialid=irct2015031617419n2. 2015.
74. Dardashti A, Ederoth P, Algotsson L, Bronden B, Grins E, Larsson M, et al. Erythropoietin and Protection of Renal Function in Cardiac Surgery (The EPRICS Trial). Surv anesthesiol. 2015;59(4):163-4.
75. Kim JE, Song SW, Kim JY, Lee HJ, Chung KH, Shim YH. Effect of a Single Bolus of Erythropoietin on Renoprotection in Patients Undergoing Thoracic Aortic Surgery With Moderate Hypothermic Circulatory Arrest. Annals of Thoracic Surgery. 2016;101(2):690-6.
76. Song YR, Lee T, You SJ, Chin HJ, Chae DW, Lim C, et al. Prevention of Acute Kidney Injury by Erythropoietin in Patients Undergoing Coronary Artery Bypass Grafting: A Pilot Study. American Journal of Nephrology. 2009;30(3):253-60.
77. Tasanarong A, Duangchana S, Sumransurp S, Homvises B, Satdhabudha O. Prophylaxis with erythropoietin versus placebo reduces acute kidney injury and neutrophil gelatinase-associated lipocalin in patients undergoing cardiac surgery: a randomized, double-blind controlled trial. BMC Nephrology. 2013;14(1):136.
78. Wahby EA, Abo Elnasr MM, Eissa MI, Mahmoud SM. Perioperative glycemic control in diabetic patients undergoing coronary artery bypass graft surgery. Journal of the Egyptian Society of Cardio-Thoracic Surgery. 2016;24(2):143-9.
79. Nour MG, Zadeh FJf. A study on the Outcomes of Modified Tight Glucose Control for the Management of
Glycemic Control in Diabetic Patients Undergoing Cardiac Surgery. Journal of Pharmacy Research 2016:764-70.
80. Tohya A, Kohjitani A, Ohno S, Yamashita K, Manabe Y, Sugimura M. Effects of glucose-insulin infusion during major oral and maxillofacial surgery on postoperative complications and outcomes. JA clinical reports. 2018;4(1):9.
81. Bartha E, Arfwedson C, Imnell A, Fernlund ME, Andersson LE, Kalman S. Randomized controlled trial of goal-directed haemodynamic treatment in patients with proximal femoral fracture. Br J Anaesth. 2013;110(4):545-53.
82. Bisgaard J, Gilsaa T, Rnholm E, Toft P. Optimising stroke volume and oxygen delivery in abdominal aortic surgery: a randomised controlled trial. Acta Anaesthesiol Scand. 2013;57(2):178-88.
83. Calvo-Vecino J, Ripolles-Melchor J, Mythen M, Casans-Frances R, Balik A, Artacho J, et al. Effect of goal-directed haemodynamic therapy on postoperative complications in low-moderate risk surgical patients: a multicentre randomised controlled trial (FEDORA trial). BR J ANAESTH. 2018;120(4):734-44.
84. Cesur S, Cardakozu T, Kus A, Turkyilmaz N, Yavuz O. Comparison of conventional fluid management with PVI-based goal-directed fluid management in elective colorectal surgery. Journal of clinical monitoring and computing. 2019;33(2):249-57.
85. Challand C, Struthers R, Sneyd J, Erasmus P, Mellor N, Hosie K, et al. Randomized controlled trial of intraoperative goal-directed fluid therapy in aerobically fit and unfit patients having major colorectal surgery. BR J ANAESTH. 2012;108(1):53-62.
86. Demirel I, Bolat E, Altun AY, Ozdemir M, Bestas A. Efficacy of Goal-Directed Fluid Therapy via Pleth Variability Index During Laparoscopic Roux-en-Y Gastric Bypass Surgery in Morbidly Obese Patients. Obesity Surgery. 2018;28(2):358-63.
87. Elgendy MA, Esmat IM, Kassim DY. Outcome of intraoperative goal-directed therapy

using Vigileo/FloTrac in high-risk patients scheduled for major abdominal surgeries: A prospective randomized trial. Egyptian Journal of Anaesthesia. 2017;33(3):263-9.
88. Funk DJ, HayGlass KT, Koulack J, Harding G, Boyd A, Brinkman R. A randomized controlled trial on the effects of goal-directed therapy on the inflammatory response open abdominal aortic aneurysm repair. Critical Care. 2015;19(1):247.
89. Goepfert M, Richter H, zu Eulenburg C, Gruetzmacher J, Rafflenbeul E, Roeher K, et al. Individually Optimized Hemodynamic Therapy Reduces Complications and Length of Stay in the Intensive Care Unit: A Prospective, Randomized Controlled Trial. Anesthesiology. 2013;119(4):824-36.
90. Gomez-Izquierdo J, Trainito A, Mirzakandov D, Stein B, Liberman S, Charlebois P, et al. Goal-directed Fluid Therapy Does Not Reduce Primary Postoperative Ileus after Elective Laparoscopic Colorectal Surgery: A Randomized Controlled Trial. Anesthesiology. 2017;127(1):36-49.
91. Han G, Liu K, Xue H, Zhao P. Application of LiDCO-Rapid in peri-operative fluid therapy for aged patients undergoing total hip replacement. International Journal of Clinical and Experimental Medicine. 2016;9(2):4473-8.
92. Harten J, Crozier JE, McCreath B, Hay A, McMillan DC, McArdle CS, et al. Effect of intraoperative fluid optimisation on renal function in patients undergoing emergency abdominal surgery: a randomised controlled pilot study (ISRCTN 11799696). Int J Surg. 2008;6(3):197-204.
93. Hasanin A, Mourad KH, Farouk I, Refaat S, Nabih A, Raouf SA, et al. The impact of goal-directed fluid therapy in prolonged major abdominal surgery on extravascular lung water and oxygenation: A randomized controlled trial. Open Access Macedonian Journal of Medical Sciences. 2019;7(8):1276-81.
94. Kapoor PM, Magoon R, Rawat R, Mehta Y. Perioperative utility of goal-directed therapy in high-risk cardiac patients undergoing coronary artery bypass grafting: A clinical outcome and biomarker-based study. Annals of Cardiac Anaesthesia. 2016;19(4):638-45.
95. Kaufmann KB, Stein L, Bogatyreva L, Ulbrich F, Kaifi JT, Hauschke D, et al. Oesophageal Doppler guided goal-directed haemodynamic therapy in thoracic surgery - a single centre randomized parallel-arm trial. Br J Anaesth. 2017;118(6):852-61.
96. Kumar L, Kanneganti YS, Rajan S. Outcomes of implementation of enhanced goal directed therapy in high-risk patients undergoing abdominal surgery. Indian journal of anaesthesia. 2015;59(4):228-33.
97. Luo J, Xue J, Liu J, Liu B, Liu L, Chen G. Goal-directed fluid restriction during brain surgery: a prospective randomized controlled trial. Annals of Intensive Care. 2017;7(1):16.
98. Marathias KP, Vassili M, Robola A, Alivizatos PA, Palatianos GM, Geroulanos S, et al. Preoperative Intravenous Hydration Confers Renoprotection in Patients With Chronic Kidney Disease Undergoing Cardiac Surgery. Artificial Organs. 2006;30(8):615-21.
99. Mikor A, Trasy D, Nemeth MF, Osztroluczki A, Kocsi S, Kovacs I, et al. Continuous central venous oxygen saturation assisted intraoperative hemodynamic management during major abdominal surgery: A randomized, controlled trial. BMC Anesthesiology. 2015;15(1):82.
100. Moppett IK, Rowlands M, Mannings A, Moran CG, Wiles MD. LiDCO-based fluid management in patients undergoing hip fracture surgery under spinal anaesthesia: a randomized trial and systematic review. Br J Anaesth. 2015;114(3):444-59.
101. Osawa EA, Rhodes A, Landoni G, Galas FR, Fukushima JT, Park CH, et al. Effect of Perioperative Goal-Directed Hemodynamic Resuscitation Therapy on Outcomes Following Cardiac Surgery: A Randomized Clinical Trial and Systematic Review. Crit Care Med. 2016;44(4):724-33.
102. Parke RL, McGuinness SP, Gilder E, McCarthy LW, Cowdrey KAL. A randomised feasibility study to assess a novel strategy to rationalise fluid in patients after cardiac surgery. British journal of anaesthesia. 2015;115(1):45-52.
103. Peng K, Li J, Cheng H, Ji FH. Goal-directed fluid therapy based on stroke volume variations improves fluid management and gastrointestinal perfusion in patients undergoing major

orthopedic surgery. Medical Principles and Practice. 2014;23(5):413-20.
104. Pestana D, Espinosa E, Eden A, Najera D, Collar L, Aldecoa C, et al. Perioperative Goal-Directed Hemodynamic Optimization Using Noninvasive Cardiac Output Monitoring in Major Abdominal Surgery: A Prospective, Randomized, Multicenter, Pragmatic Trial: POEMAS Study (PeriOperative goal-directed thErapy in Major Abdominal Surgery). Anesth Analg. 2014;119(3):579-87.
105. Phan T, D'Souza B, Rattray M, Johnston M, Cowie B. A Randomised Controlled Trial of Fluid Restriction Compared to Oesophageal Doppler-Guided Goal-Directed Fluid Therapy in Elective Major Colorectal Surgery within an Enhanced Recovery after Surgery Program. Anaesthesia and intensive care. 2014;42:752-60.
106. Puckett JR, Pickering JW, Palmer SC, McCall JL, Kluger MT, De Zoysa J, et al. Low Versus Standard Urine Output Targets in Patients Undergoing Major Abdominal Surgery. Annals of Surgery. 2017;265(5):874-81.
107. Pull ter Gunne AJ, Bruining HA, Obertop H. Haemodynamics and 'optimal' hydration in aortic cross clamping. The Netherlands journal of surgery. 1990;42(4):113-7.
108. Schmid S, Kapfer B, Heim M, Bogdanski R, Anetsberger A, Blobner M, et al. Algorithm-guided goal-directed haemodynamic therapy does not improve renal function after major abdominal surgery compared to good standard clinical care: a prospective randomised trial. Critical Care. 2016;20(1):50.
109. Weinberg L, Harris L, Bellomo R, Ierino FL, Story D, Eastwood G, et al. Effects of intraoperative and early postoperative normal saline or Plasma-Lyte 148® on hyperkalaemia in deceased donor renal transplantation: a double-blind randomized trial. British journal of anaesthesia. 2017;119(4):606-15.
110. Weinberg L, Ianno D, Churilov L, McGuigan S, Mackley L, Banting J, et al. Goal directed fluid therapy for major liver resection: A multicentre randomized controlled trial. Annals of Medicine and Surgery. 2019;45:45-53.
111. Wu CY, Lin YS, Tseng HM, Cheng HL, Lee TS, Lin PL, et al. Comparison of two stroke volume variation-based goal-directed fluid therapies for supratentorial brain tumour resection: A randomized controlled trial. British Journal of Anaesthesia. 2017;119(5):934-42.
112. Wu X, Jiang Z, Ying J, Han Y, Chen Z. Optimal blood pressure decreases acute kidney injury after gastrointestinal surgery in elderly hypertensive patients: A randomized study: Optimal blood pressure reduces acute kidney injury. Journal of Clinical Anesthesia. 2017;43:77-83.
113. Xu H, Shu SH, Wang D, Chai XQ, Xie YH, Zhou WD. Goal-directed fluid restriction using stroke volume variation and cardiac index during one-lung ventilation: A randomized controlled trial. Journal of Thoracic Disease. 2017;9(9):2992-3004.
114. Yin kD, J. Wu, Y. Randomized clinical trail assessing the effect of goal-directed fluid therapy based on NICOM on prognosis in elderly patients undergoing resection of gastrointestinal tumor. Http://wwwwhoint/trialsearch/trial2aspx? Trialid=chictr1800014388. 2018.
115. Zakhaleva J, Tam J, Denoya PI, Bishawi M, Bergamaschi R. The impact of intravenous fluid administration on complication rates in bowel surgery within an enhanced recovery protocol: A randomized controlled trial. Colorectal Disease. 2013;15(7):892-9.
116. Zhao G, Peng P, Zhou Y, Li J, Jiang H, Shao J. The accuracy and effectiveness of goal directed fluid therapy in plateau-elderly gastrointestinal cancer patients: a prospective randomized controlled trial. International Journal of Clinical and Experimental Medicine. 2018;11(8):8516-22.
117. Zheng H, Guo H, Ye J-R, Chen L, Ma H-P. Goal-directed fluid therapy in gastrointestinal surgery in older coronary heart disease patients: randomized trial. World journal of surgery. 2013;37(12):2820-9.
118. Baysal A, Yanartas M, Dogukan M, Gundogus N, Kocak T, Koksal C. Levosimendan improves renal outcome in cardiac surgery: A randomized trial. Journal of Cardiothoracic and Vascular Anesthesia. 2014;28(3):586-94.
119. Bragadottir G, Redfors B, Ricksten SE. Effects of levosimendan on glomerular filtration rate, renal blood flow, and renal oxygenation after cardiac surgery with cardiopulmonary bypass: a randomized placebo-controlled study. Crit Care Med. 2013;41(10):2328-35.

120. Cholley B, Caruba T, Grosjean S, Amour J, Ouattara A, Villacorta J, et al. Effect of Levosimendan on Low Cardiac Output Syndrome in Patients With Low Ejection Fraction Undergoing Coronary Artery Bypass Grafting With Cardiopulmonary Bypass: The LICORN Randomized Clinical Trial. Jama. 2017;318(6):548-56.
121. Desai PM, Sarkar MS, Umbarkar SR. Prophylactic preoperative levosimendan for off-pump coronary artery bypass grafting in patients with left ventricular dysfunction: Single-centered randomized prospective study. Annals of Cardiac Anaesthesia. 2018;21(2):123-8.
122. Erb J, Beutlhauser T, Feldheiser A, Schuster B, Treskatsch S, Grubitzsch H, et al. Influence of levosimendan on organ dysfunction in patients with severely reduced left ventricular function undergoing cardiac surgery. J Int Med Res. 2014;42(3):750-64.
123. Landoni G, Lomivorotov VV, Alvaro G, Lobreglio R, Pisano A, Guarracino F, et al. Levosimendan for Hemodynamic Support after Cardiac Surgery. New England Journal of Medicine. 2017;376(21):2021-31.
124. Levin R, Degrange M, Del Mazo C, Tanus E, Porcile R. Preoperative levosimendan decreases mortality and the development of low cardiac output in high-risk patients with severe left ventricular dysfunction undergoing coronary artery bypass grafting with cardiopulmonary bypass. Experimental and clinical cardiology. 2012;17(3):125-30.
125. Mehta RH, Leimberger JD, van Diepen S, Meza J, Wang A, Jankowich R, et al. Levosimendan in Patients with Left Ventricular Dysfunction Undergoing Cardiac Surgery. New England Journal of Medicine. 2017;376(21):2032-42.
126. Ristikankare A, Poyhia R, Eriksson H, Valtonen M, Leino K, Salmenpera M. Effects of levosimendan on renal function in patients undergoing coronary artery surgery. J Cardiothorac Vasc Anesth. 2012;26(4):591-5.
127. Shah B, Sharma P, Brahmbhatt A, Shah R, Rathod B, Shastri N, et al. Study of levosimendan during off-pump coronary artery bypass grafting in patients with LV dysfunction: a double-blind randomized study. Indian J Pharmacol. 2014;46(1):29-34.
128. Sharma P, Malhotra A, Gandhi S, Garg P, Bishnoi A, Gandhi H. Preoperative levosimendan in ischemic mitral valve repair. Asian Cardiovascular and Thoracic Annals. 2013;22(5):539-45.
129. Zangrillo A, Alvaro G, Belletti A, Pisano A, Brazzi L, Calabro MG, et al. Effect of Levosimendan on Renal Outcome in Cardiac Surgery Patients With Chronic Kidney Disease and Perioperative Cardiovascular Dysfunction: A Substudy of a Multicenter Randomized Trial. Journal of Cardiothoracic and Vascular Anesthesia. 2018;32(5):2152-9.
130. Atalay H, Temizturk Z, Altinsoy HB, Azboy D, Colak S, Atalay A, et al. Levosimendan use increases cardiac performance after coronary artery bypass grafting in end-stage renal disease patients. Heart Surgery Forum. 2016;19(5):E230-E6.
131. Bebawy J, Ramaiah V, Mbbs MD, Zeeni C, Hemmer L, Koht A, et al. The Effect of Furosemide on Intravascular Volume Status and Electrolytes in Patients Receiving Mannitol: An Intraoperative Safety Analysis. J Neurosurg Anesthesiol. 2013;25(1):51-4.
132. Danelich IM, Bergquist JR, Bergquist WJ, Osborn JL, Wright SS, Tefft BJ, et al. Early diuresis after colon and rectal surgery does not reduce length of hospital stay: Results of a randomized trial. Diseases of the Colon and Rectum. 2018;61(10):1187-95.
133. Luo LL, Ni J, Luo D, Gao XR, Huang W, Lin X. [Low-dose of furosemide to correct oliguria in gynecological surgery]. Sichuan da xue xue bao Yi xue ban = Journal of Sichuan University Medical science edition. 2013;44(5):783-6.
134. Mahesh B, Yim B, Robson D, Pillai R, Ratnatunga C, Pigott D. Does furosemide prevent renal dysfunction in high-risk cardiac surgical patients? Results of a double-blinded prospective randomised trial. Eur J Cardiothorac Surg. 2008;33(3):370-6.
135. Abd El Aal F, Abbas M. N-acetylcysteine for renal protection in patients with rheumatic heart disease undergoing valve replacement. 2013;7(1):19-26.
136. Adabag AS, Ishani A, Koneswaran S, Johnson DJ, Kelly RF, Ward HB, et al. Utility of N-acetylcysteine to prevent acute kidney injury

after cardiac surgery: a randomized controlled trial. Am Heart J. 2008;155(6):1143-9.
137. Ali Karami SRS, Pooya Vatankhah , Seyd Hedayatallah Akhlagh , Mansoor Masjedi and Masih Shafa. Evaluation of Protective Effects of N-Acetyl Cysteine and Vitamin C on Perioperative Renal Function in High-Risk Patients Undergoing Coronary Artery Surgeries, a Randomized Clinical Trial. Research Journal of Medical Sciences. 2016;10(6):711-5.
138. Amini S, Robabi HN, Tashnizi MA, Vakili V. Selenium, Vitamin C and N-Acetylcysteine do not Reduce the Risk of Acute Kidney Injury after Off-Pump CABG: a Randomized Clinical Trial. Brazilian journal of cardiovascular surgery. 2018;33(2):129-34.
139. Banu AYHAN AGP, Baflak KANTAR, Meral KANBAK, Bilge ÇELEB‹O⁄LU, Ülkü AYPAR. renal functional effects of using n-acetyl-cysteine (nac) in cardiac surgery. Journal of Anesthesia. 2012;20(3):159-67.
140. Burns KE, Chu MW, Novick RJ, Fox SA, Gallo K, Martin CM, et al. Perioperative N-acetylcysteine to prevent renal dysfunction in high-risk patients undergoing cabg surgery: a randomized controlled trial. Jama. 2005;294(3):342-50.
141. El-Hamamsy I, Stevens LM, Carrier M, Pellerin M, Bouchard D, Demers P, et al. Effect of intravenous N-acetylcysteine on outcomes after coronary artery bypass surgery: a randomized, double-blind, placebo-controlled clinical trial. J Thorac Cardiovasc Surg. 2007;133(1):7-12.
142. Fischer UM, Tossios P, Mehlhorn U. Renal protection by radical scavenging in cardiac surgery patients. Current Medical Research and Opinion. 2005;21(8):1161-4.
143. Haase M, Haase-Fielitz A, Bagshaw SM, Reade MC, Morgera S, Seevenayagam S, et al. Phase II, randomized, controlled trial of high-dose N-acetylcysteine in high-risk cardiac surgery patients. Crit Care Med. 2007;35(5):1324-31.
144. Hynninen MS, Niemi TT, Poyhia R, Raininko EI, Salmenpera MT, Lepantalo MJ, et al. N-acetylcysteine for the prevention of kidney injury in abdominal aortic surgery: a randomized, double-blind, placebo-controlled trial. Anesth Analg. 2006;102(6):1638-45.
145. Prasad A, Banakal S, Muralidhar K. N-acetylcysteine does not prevent renal dysfunction after off-pump coronary artery bypass surgery. Eur J Anaesthesiol. 2010;27(11):973-7.
146. Ristikankare A, Kuitunen T, Kuitunen A, Uotila L, Vento A, Suojaranta-Ylinen R, et al. Lack of renoprotective effect of i.v. N-acetylcysteine in patients with chronic renal failure undergoing cardiac surgery. Br J Anaesth. 2006;97(5):611-6.
147. Sisillo E, Ceriani R, Bortone F, Juliano G, Salvi L, Veglia F, et al. N-acetylcysteine for prevention of acute renal failure in patients with chronic renal insufficiency undergoing cardiac surgery: a prospective, randomized, clinical trial. Crit Care Med. 2008;36(1):81-6.
148. Song JW, Shim JK, Soh S, Jang J, Kwak YL. Double-blinded, randomized controlled trial of N-acetylcysteine for prevention of acute kidney injury in high risk patients undergoing off-pump coronary artery bypass. Nephrology (Carlton, Vic). 2015;20(2):96-102.
149. Wijeysundera DN, Beattie WS, Rao V, Granton JT, Chan CT. N-acetylcysteine for preventing acute kidney injury in cardiac surgery patients with pre-existing moderate renal insufficiency. Canadian journal of anaesthesia = Journal canadien d'anesthesie. 2007;54(11):872-81.
150. Santana-Santos E, Gowdak L, Md P, Gaiotto F, Md P, Puig L, et al. High Dose of N-Acetylcystein Prevents Acute Kidney Injury in Chronic Kidney Disease Patients Undergoing Myocardial Revascularization. Ann Thorac Surg. 2014;97(5):1617-23.
151. Amano J, Suzuki A, Sunamori M. Salutary effect of reduced glutathione on renal function in coronary artery bypass operation. Journal of the American College of Surgeons. 1994;179(6):714-20.
152. Billings FTt, Petracek MR, Roberts LJ, 2nd, Pretorius M. Perioperative intravenous acetaminophen attenuates lipid peroxidation in adults undergoing cardiopulmonary bypass: a randomized clinical trial. PloS one. 2015;10(2):e0117625.
153. Nouri-Majalan N, Ardakani EF, Forouzannia K, Moshtaghian H. Effects of allopurinol and vitamin E on renal function in patients with cardiac coronary artery bypass grafts. Vasc Health Risk Manag. 2009;5(2):489-94.
154. Wijnen MH, Vader HL, Van Den Wall Bake AW, Roumen RM. Can renal dysfunction after

infra-renal aortic aneurysm repair be modified by multi-antioxidant supplementation? The Journal of cardiovascular surgery. 2002;43(4):483-8.
155. Amit X. Garg MD PJDM, Andrew Hill MD, Manish Sood MD, Bharat Aggarwal PhD, Luc Dubois MD,, Swapnil Hiremath MD RGM, Vikram Iyer MD, Matthew James MD, Eric McArthur MSc,, Louise Moist MD GOM, Chirag R. Parikh MD, Virginia Schumann, Sumit Sharan MD,, Heather Thiessen-Philbrook MMath STM, Ron Wald MD, Michael Walsh MD, Matthew Weir MD,, Investigators NPMCAA. Oral curcumin in elective abdominal aortic aneurysm repair: a multicentre randomized controlled trial. CMAJ : canadian medical association journal. 2018;190(43):E1273-E80.
156. Balaguer J, Yu C, Byrne J, Ball S, Petracek M, Brown N, et al. Contribution of Endogenous Bradykinin to Fibrinolysis, Inflammation, and Blood Product Transfusion Following Cardiac Surgery: A Randomized Clinical Trial. Clin Pharmacol Ther. 2013;93(4):326-34.
157. Blogowski W, Dolegowska B, Pikula E, Gutowski P, Starzynska T. The effect of PGE administration on the activity of oxidative system in erythrocytes and platelets during ischemia reperfusion injury and on postoperative renal function in patients undergoing open abdominal aortic aneurysm reconstruction. Journal of Biological Regulators and Homeostatic Agents. 2012;26(3):429-38.
158. Choi YS, Shim JK, Song JW, Song Y, Yang SY, Kwak YL. Efficacy of perioperative oral triiodothyronine replacement therapy in patients undergoing off-pump coronary artery bypass grafting. Journal of Cardiothoracic and Vascular Anesthesia. 2013;27(6):1218-23.
159. Dastan F, Talasaz AH, Mojtahedzadeh M, Karimi A, Salehiomran A, Bina P, et al. Randomized Trial of Carnitine for the Prevention of Perioperative Atrial Fibrillation. Seminars in thoracic and cardiovascular surgery. 2018;30(1):7-13.
160. Demirjian S, Ailawadi G, Polinsky M, Bitran D, Silberman S, Shernan SK, et al. Safety and tolerability study of an intravenously administered small interfering ribonucleic acid (siRNA) post on-pump cardiothoracic surgery in patients at risk of acute kidney injury. KI reports. 2017;2(5):836-43.
161. Ederoth P, Dardashti A, Grins E, Bronden B, Metzsch C, Erdling A, et al. Cyclosporine before Coronary Artery Bypass Grafting Does Not Prevent Postoperative Decreases in Renal Function: A Randomized Clinical Trial. Anesthesiology. 2018;128(4):710-7.
162. Ellenberger C, Md M, Sologashvili T, Kreienbuhl L, Cikirikcioglu M, Diaper J, et al. Myocardial Protection by Glucose-Insulin-Potassium in Moderate- to High-Risk Patients Undergoing Elective On-Pump Cardiac Surgery: A Randomized Controlled Trial. Anesth Analg. 2018;126(4):1133-41.
163. Fayfman M, Davis G, Duggan EW, Urrutia M, Chachkhiani D, Schindler J, et al. Sitagliptin for prevention of stress hyperglycemia in patients without diabetes undergoing general surgery: a pilot randomized study. Journal of diabetes and its complications. 2018;32(12):1091-6.
164. Golestaneh L, Lindsey K, Malhotra P, Kargoli F, Farkas E, Barner H, et al. Acute kidney injury after cardiac surgery: is minocycline protective? J Nephrol. 2015;28(2):193-9.
165. Himmelfarb J, Chertow GM, McCullough PA, Mesana T, Shaw AD, Sundt TM, et al. Perioperative THR-184 and AKI after Cardiac Surgery. Journal of the American Society of Nephrology : JASN. 2018;29(2):670-9.
166. Kishimoto Y, Nakamura Y, Harada S, Onohara T, Kishimoto S, Kurashiki T, et al. Can tolvaptan protect renal function in the early postoperative period of cardiac surgery?: - Results of a single-center randomized controlled study. Circulation Journal. 2018;82(4):999-1007.
167. Kramer BK, Preuner J, Ebenburger A, Kaiser M, Bergner U, Eilles C, et al. Lack of renoprotective effect of theophylline during aortocoronary bypass surgery. Nephrol Dial Transplant. 2002;17(5):910-5.
168. Mansourian S, Bina P, Fehri A, Karimi AA, Boroumand MA, Abbasi K. Preoperative oral pentoxifylline in case of coronary artery bypass grafting with left ventricular dysfunction (ejection fraction equal to/less than 30%). Anatolian journal of cardiology. 2015;15(12):1014-9.
169. Morgera S, Woydt R, Kern H, Schmutzler M, DeJonge K, Lun A, et al. Low-dose prostacyclin preserves renal function in high-risk patients after coronary bypass surgery. Crit Care Med. 2002;30(1):107-12.

170. Schmidt T, Pargger H, Seeberger E, Eckhart F, von Felten S, Haberthur C. Effect of high-dose sodium selenite in cardiac surgery patients: A randomized controlled bi-center trial. Clinical nutrition (Edinburgh, Scotland). 2018;37(4):1172-80.
171. Shahbazi S, Alishahi P, Asadpour E. Evaluation of the effect of aminophylline in reducing the incidence of acute kidney injury after cardiac surgery. Anesthesiology and Pain Medicine. 2017;7(4):e21740.
172. Soliman R, Nofal H. The effect of perioperative magnesium sulfate on blood sugar in patients with diabetes mellitus undergoing cardiac surgery: a double-blinded randomized study. Annals of cardiac anaesthesia. 2019;22(2):151-7.
173. Song J, Park J, Kim JY, Kim JD, Kang WS, Muhammad HB, et al. Effect of ulinastatin on perioperative organ function and systemic inflammatory reaction during cardiac surgery: A randomized double-blinded study. Korean Journal of Anesthesiology. 2013;64(4):334-40.
174. Stoppe C, Fahlenkamp A, Rex S, Veeck N, Gozdowsky S, Schalte G, et al. Feasibility and safety of xenon compared with sevoflurane anaesthesia in coronary surgical patients: a randomized controlled pilot study+. BR J ANAESTH. 2013;111(3):406-16.
175. Thompson JN, Cohen J, Blenkharn JI, McConnell JS, Barr J, Blumgart LH. A randomized clinical trial of oral ursodeoxycholic acid in obstructive jaundice. Br J Surg. 1986;73(8):634-6.
176. Wang P, Wang HW, Zhong TD. Influence of different anesthesia on liver and renal function in elderly patients undergoing laparoscopic colon or rectal resection. Hepato-Gastroenterology. 2013;60(122):79-82.
177. Yu Q, Li Q, Yang X, Liu Q, Deng J, Zhao Y, et al. A comparison of the efficacy between general anesthsia and local anesthesia in the interventional surgery for abdominal aortic dissection. International Journal of Clinical and Experimental Medicine. 2018;11(4):3954-60.
178. Demir A, Yılmaz FM, Ceylan C, Doluoglu OG, Uçar P, Züngün C, et al. A comparison of the effects of ketamine and remifentanil on renal functions in coronary artery bypass graft surgery. Renal Failure. 2015;37(5):819-26.
179. Kaya K, Oguz M, Akar AR, Durdu S, Aslan A, Erturk S, et al. The effect of sodium nitroprusside infusion on renal function during reperfusion period in patients undergoing coronary artery bypass grafting: a prospective randomized clinical trial. Eur J Cardiothorac Surg. 2007;31(2):290-7.
180. Lei C, Berra L, Rezoagli E, Yu B, Dong H, Yu S, et al. Nitric Oxide Decreases Acute Kidney Injury and Stage 3 Chronic Kidney Disease after Cardiac Surgery. Am J Respir Crit Care Med. 2018;198(10):1279-87.
181. Gubern JM, Sancho JJ, Simo J, Sitges-Serra A. A randomized trial on the effect of mannitol on postoperative renal function in patients with obstructive jaundice. Surgery. 1988;103(1):39-44.
182. Nicholson ML, Baker DM, Hopkinson BR, Wenham PW. Randomized controlled trial of the effect of mannitol on renal reperfusion injury during aortic aneurysm surgery. Br J Surg. 1996;83(9):1230-3.
183. Raghava A, Bidkar PU, Prakash MVSS, Hemavathy B. Comparison of equiosmolar concentrations of hypertonic saline and mannitol for intraoperative lax brain in patients undergoing craniotomy. Surgical neurology international. 2015;6:73.
184. Shim JK, Choi SH, Oh YJ, Kim CS, Yoo KJ, Kwak YL. The effect of mannitol on oxygenation and creatine kinase MB release in patients undergoing multivessel off-pump coronary artery bypass surgery. J Thorac Cardiovasc Surg. 2007;133(3):704-9.
185. Barba-Navarro R, Tapia-Silva M, Garza-Garcia C, Lopez-Giacoman S, Melgoza-Toral I, Vazquez-Rangel A, et al. The Effect of Spironolactone on Acute Kidney Injury After Cardiac Surgery: A Randomized, Placebo-Controlled Trial. American Journal of Kidney Diseases. 2017;69(2):192-9.
186. Peng NH, Gao T, Chen YY, Xi FC, Zhang JJ, Li N, et al. Restricted intravenous fluid regimen reduces fluid redistribution of patients operated for abdominal malignancy. Hepatogastroenterology. 2013;60(127):1653-9.
187. Belavic M. A restrictive dose of crystalloids in patients during laparoscopic cholecystectomy is safe and cost-effective: prospective, two-arm parallel, randomized controlled trial.

Http://wwwwhoint/trialsearch/trial2aspx? Trialid=drks00007904 https://wwwdovepresscom/a-restrictive-dose-of-crystalloids-in-patients-during-laparoscopic-cho-peer-reviewed-fulltext-article-TCRM. 2018.
188. Choi SS, Jun IG, Cho SS, Kim SK, Hwang GS, Kim YK. Effect of stroke volume variation-directed fluid management on blood loss during living-donor right hepatectomy: a randomised controlled study. Anaesthesia. 2015;70(11):1250-8.
189. Matot I, Paskaleva R, Eid L, Cohen K, Khalaileh A, Elazary R, et al. Effect of the Volume of Fluids Administered on Intraoperative Oliguria in Laparoscopic Bariatric Surgery: A Randomized Controlled Trial. Arch Surg. 2012;147(3):228-34.
190. Matot I, Dery E, Bulgov Y, Cohen B, Paz J, Nesher N. Fluid management during video-assisted thoracoscopic surgery for lung resection: A randomized, controlled trial of effects on urinary output and postoperative renal function. J Thorac Cardiovasc Surg. 2013;146(2):461-6.
191. Abraham-Nordling M, Hjern F, Pollack J, Prytz M, Borg T, Kressner U. Randomized clinical trial of fluid restriction in colorectal surgery. Br J Surg. 2012;99(2):186-91.
192. Bhaskaran K, Arumugam G, Vinay Kumar PV. A prospective, randomized, comparison study on effect of perioperative use of chloride liberal intravenous fluids versus chloride restricted intravenous fluids on postoperative acute kidney injury in patients undergoing off-pump coronary artery bypass grafting surgeries. Annals of Cardiac Anaesthesia. 2018;21(4):413-8.
193. Gao T, Li N, Zhang JJ, Xi FC, Chen QY, Zhu WM, et al. Restricted intravenous fluid regimen reduces the rate of postoperative complications and alters immunological activity of elderly patients operated for abdominal cancer: a randomized prospective clinical trail. World J Surg. 2012;36(5):993-1002.
194. Hubner M, Schafer M, Demartines N, Muller S, Maurer K, Baulig W, et al. Impact of restrictive intravenous fluid replacement and combined epidural analgesia on perioperative volume balance and renal function within a Fast Track program. The Journal of surgical research. 2012;173(1):68-74.
195. Jie HY, Ye JL, Zhou HH, Li YX. Perioperative restricted fluid therapy preserves immunological function in patients with colorectal cancer. World Journal of Gastroenterology. 2014;20(42):15852-9.
196. Mazer CD, Whitlock RP, Fergusson DA, Belley-Cote E, Connolly K, Khanykin B, et al. Six-Month Outcomes after Restrictive or Liberal Transfusion for Cardiac Surgery. New England journal of medicine. 2018;379(13):1224-33.
197. Myles PS, Bellomo R, Corcoran T, Forbes A, Peyton P, Story D, et al. Restrictive versus Liberal Fluid Therapy for Major Abdominal Surgery. The New England journal of medicine. 2018;378(24):2263-74.
198. Peng NH, Gao T, Chen YY, Xi FC, Zhang JJ, Li N, et al. Restricted intravenous fluid regimen reduces fluid redistribution of patients operated for abdominal malignancy. Hepato-Gastroenterology. 2013;60(127):1653-9.
199. Piljic D, Petricevic M, Piljic D, Ksela J, Robic B, Klokocovnik T. Restrictive versus Standard Fluid Regimen in Elective Minilaparotomy Abdominal Aortic Repair-Prospective Randomized Controlled Trial. The Thoracic and cardiovascular surgeon. 2016;64(4):296-303.
200. Shehata N, Burns L, Nathan H, Hebert P, Hare G, Fergusson D, et al. A randomized controlled pilot study of adherence to transfusion strategies in cardiac surgery. Transfusion. 2012;52(1):91-9.
201. van Samkar G, Eshuis WJ, Bennink RJ, van Gulik TM, Dijkgraaf MGW, Preckel B, et al. Intraoperative Fluid Restriction in Pancreatic Surgery: A Double Blinded Randomised Controlled Trial. PLOS ONE. 2015;10(10):e0140294.
202. Brulotte V, Leblond FA, Elkouri S, Therasse E, Pichette V, Beaulieu P. Bicarbonates for the prevention of postoperative renal failure in endovascular aortic aneurysm repair: a randomized pilot trial. Anesthesiology research and practice. 2013;2013.
203. Cho JS, Soh S, Shim J-K, Kang S, Choi H, Kwak Y-L. Effect of perioperative sodium bicarbonate administration on renal function following cardiac surgery for infective endocarditis: a randomized, placebo-controlled trial. Critical Care. 2017;21(1):3.
204. Haase-Fielitz A, Haase M, Plass M, Murray P, Bailey M, Bellomo R, et al. Perioperative sodium bicarbonate to prevent acute kidney

injury after cardiac surgery: A multicenter double-blind randomized controlled trial. Critical Care. 2013;17(SUPPL. 2):S154-S5.
205. Kristeller J, Zavorsky G, Prior J, Keating D, Brady M, Romaldini T, et al. Lack of Effectiveness of Sodium Bicarbonate in Preventing Kidney Injury in Patients Undergoing Cardiac Surgery: A Randomized Controlled Trial. Pharmacotherapy. 2013;33(7):710-7.
206. McGuinness S, Mb C, Frca F, Parke R, Msc RN, Bellomo R, et al. Sodium Bicarbonate Infusion to Reduce Cardiac Surgery-Associated Acute Kidney Injury: A Phase II Multicenter Double-Blind Randomized Controlled Trial*. Crit Care Med. 2013;41(7):1599-607.
207. Muralidhar Kanchi1 RM, Jos Maessen2, Lloyd Vincent3, Kumar Belani4. Effect of sodium bicarbonate infusion in off-pump coronary artery bypass grafting in patients with renal dysfunction. Journal of anaesthesiology, clinical pharmacology. 2018;34(3):301-6.
208. Saratzis A, Chiocchia V, Jiffry A, Hassanali N, Singh S, Imray CH, et al. HYDration and Bicarbonate to Prevent Acute Renal Injury After Endovascular Aneurysm Repair With Suprarenal Fixation: Pilot/Feasibility Randomised Controlled Study (HYDRA Pilot Trial). European journal of vascular and endovascular surgery : the official journal of the European Society for Vascular Surgery. 2018;55(5):648-56.
209. Soh S, Song JW, Shim JK, Kim JH, Kwak YL. Sodium bicarbonate does not prevent postoperative acute kidney injury after off-pump coronary revascularization: A double-blinded randomized controlled trial. British Journal of Anaesthesia. 2016;117(4):450-7.
210. Turner KR, Fisher EC, Hade EM, Houle TT, Rocco MV. The role of perioperative sodium bicarbonate infusion affecting renal function after cardiothoracic surgery. Frontiers in pharmacology. 2014;5:127-.
211. Almansob MAS, Xu B, Zhou L, Hu XX, Chen W, Chang FJ, et al. Simvastatin reduces myocardial injury undergoing noncoronary artery cardiac surgery: A randomized controlled trial. Arteriosclerosis, Thrombosis, and Vascular Biology. 2012;32(9):2304-13.
212. Billings F, Hendricks P, Schildcrout J, Shi Y, Petracek M, Byrne J, et al. High-Dose Perioperative Atorvastatin and Acute Kidney Injury Following Cardiac Surgery: A Randomized Clinical Trial. JAMA. 2016;315(9):877-88.
213. Park JH, Shim J-K, Song J-W, Soh S, Kwak Y-L. Effect of atorvastatin on the incidence of acute kidney injury following valvular heart surgery: a randomized, placebo-controlled trial. Intensive Care Medicine. 2016;42(9):1398-407.
214. Prowle J, Calzavacca P, Licari E, Ligabo E, Echeverri J, Haase M, et al. Pilot double-blind, randomized controlled trial of short-term atorvastatin for prevention of acute kidney injury after cardiac surgery. Nephrology. 2012;17(3):215-24.
215. Zheng Z, Jayaram R, Jiang L, Emberson J, Zhao Y, Li Q, et al. Perioperative Rosuvastatin in Cardiac Surgery. New England Journal of Medicine. 2016;374(18):1744-53.
216. Dieleman JM, Nierich AP, Rosseel PM, van der Maaten JM, Hofland J, Diephuis JC, et al. Intraoperative high-dose dexamethasone for cardiac surgery: a randomized controlled trial. JAMA. 2012;308(17):1761-7.
217. Garg A, Chan M, Cuerden M, Devereaux PJ, Abbasi S, Hildebrand A, et al. Effect of methylprednisolone on acute kidney injury in patients undergoing cardiac surgery with a cardiopulmonary bypass pump: a randomized controlled trial. CMAJ. 2019;191(9):E247-E56.
218. Loef BG, Henning RH, Epema AH, Rietman GW, van Oeveren W, Navis GJ, et al. Effect of dexamethasone on perioperative renal function impairment during cardiac surgery with cardiopulmonary bypass. British Journal of Anaesthesia. 2004;93(6):793-8.
219. Morariu AM, Loef BG, Aarts LPHJ, Rietman GW, Rakhorst G, van Oeveren W, et al. Dexamethasone: benefit and prejudice for patients undergoing on-pump coronary artery bypass grafting: a study on myocardial, pulmonary, renal, intestinal, and hepatic injury. Chest. 2005;128(4):2677-87.
220. Turner S, Derham C, Orsi NM, Bosomworth M, Bellamy MC, Howell SJ. Randomized clinical trial of the effects of methylprednisolone on renal function after major vascular surgery. The British journal of surgery. 2008;95(1):50-6.
221. Abbas MS, Ibraheim OA, Ahmed GA, Mahdy M. Terlipressin infusion during hepatobiliary surgery could improve systemic and

splanchnic hemodynamics and renal function: a double-blind randomized clinical trial. Anesthesia and analgesia. 2018;126(4):305-6.
222. Futier E, Lefrant J-Y, Guinot P-G, Godet T, Lorne E, Cuvillon P, et al. Effect of Individualized vs Standard Blood Pressure Management Strategies on Postoperative Organ Dysfunction Among High-Risk Patients Undergoing Major Surgery: A Randomized Clinical Trial. JAMA. 2017;318(14):1346-57.
223. Urzua J, Troncoso S, Bugedo G, Canessa R, Muñoz H, Lema G, et al. Renal function and cardiopulmonary bypass: Effect of perfusion pressure. Journal of Cardiothoracic and Vascular Anesthesia. 1992;6(3):299-303.
224. Zhao X, Wu X, Xu G. Sevoflurane versus total intravenous anesthesia for cardiac surgery. International Journal of Clinical and Experimental Medicine. 2017;10(9):13739-45.
225. Song JC, Zhang MZ, Wu QC, Lu ZJ, Sun YM, Yang LQ, et al. Sevoflurane has no adverse effects on renal function in cirrhotic patients: A comparison with propofol. Acta Anaesthesiologica Scandinavica. 2013;57(7):896-902.
226. Yoo YC, Shim JK, Song Y, Yang SY, Kwak YL. Anesthetics influence the incidence of acute kidney injury following valvular heart surgery. Kidney International. 2014;86(2):414-22.
227. Landoni G, Lomivorotov VV, Nigro Neto C, Monaco F, Pasyuga VV, Bradic N, et al. Volatile Anesthetics versus Total Intravenous Anesthesia for Cardiac Surgery. New England Journal of Medicine. 2019;380(13):1214-25.
228. Wasowicz M, Jerath A, Luksun W, Sharma V, Mitsakakis N, Meineri M, et al. Comparison of propofol-based versus volatile-based anaesthesia and postoperative sedation in cardiac surgical patients: a prospective, randomized, study. Anaesthesiology intensive therapy. 2018;50(3):200-9.
